# Executive summary: Italian guidelines for diagnosis, risk stratification, and care continuity of fragility fractures 2021

**DOI:** 10.3389/fendo.2023.1137671

**Published:** 2023-04-18

**Authors:** Giovanni Corrao, Annalisa Biffi, Gloria Porcu, Raffaella Ronco, Giovanni Adami, Rosaria Alvaro, Riccardo Bogini, Achille Patrizio Caputi, Luisella Cianferotti, Bruno Frediani, Davide Gatti, Stefano Gonnelli, Giovanni Iolascon, Andrea Lenzi, Salvatore Leone, Raffaella Michieli, Silvia Migliaccio, Tiziana Nicoletti, Marco Paoletta, Annalisa Pennini, Eleonora Piccirilli, Maurizio Rossini, Umberto Tarantino, Maria Luisa Brandi

**Affiliations:** ^1^ National Centre for Healthcare Research and Pharmacoepidemiology, Laboratory of the University of Milano-Bicocca, Milan, Italy; ^2^ Department of Statistics and Quantitative Methods, Unit of Biostatistics, Epidemiology, and Public Health, University of Milano-Bicocca, Milan, Italy; ^3^ Rheumatology Unit, University of Verona, Verona, Italy; ^4^ Department of Biomedicine and Prevention, University of Rome Tor Vergata, Rome, Italy; ^5^ Local Health Unit (USL) Umbria, Perugia, Italy; ^6^ Department of Pharmacology, School of Medicine, University of Messina, Messina, Italy; ^7^ Italian Bone Disease Research Foundation, Fondazione Italiana Ricerca sulle Malattie dell’Osso (FIRMO), Florence, Italy; ^8^ Department of Medicine, Surgery and Neurosciences, Rheumatology Unit, University of Siena, Azienda Ospedaliero-Universitaria Senese, Siena, Italy; ^9^ Department of Medicine, Surgery and Neuroscience, Policlinico Le Scotte, University of Siena, Siena, Italy; ^10^ Department of Medical and Surgical Specialties and Dentistry, University of Campania “Luigi Vanvitelli”, Naples, Italy; ^11^ Department of Experimental Medicine, Sapienza University of Rome, Viale del Policlinico, Rome, Italy; ^12^ AMICI Onlus, Associazione Nazionale per le Malattie Infiammatorie Croniche dell’Intestino, Milan, Italy; ^13^ Italian Society of General Medicine and Primary Care Società Italiana di Medicina Generale e delle cure primarie (SIMG), Florence, Italy; ^14^ Department of Movement, Human and Health Sciences, Foro Italico University, Rome, Italy; ^15^ CnAMC, Coordinamento nazionale delle Associazioni dei Malati Cronici e rari di Cittadinanzattiva, Rome, Italy; ^16^ Department of Clinical Sciences and Translational Medicine, University of Rome “Tor Vergata”, Rome, Italy; ^17^ Department of Orthopedics and Traumatology, “Policlinico Tor Vergata” Foundation, Rome, Italy

**Keywords:** evidence-based guideline, fragility fracture, secondary prevention, systematic review, grade

## Abstract

**Background:**

Fragility fractures are a major public health concern owing to their worrying and growing burden and their onerous burden upon health systems. There is now a substantial body of evidence that individuals who have already suffered a fragility fracture are at a greater risk for further fractures, thus suggesting the potential for secondary prevention in this field.

**Purpose:**

This guideline aims to provide evidence-based recommendations for recognizing, stratifying the risk, treating, and managing patients with fragility fracture. This is a summary version of the full Italian guideline.

**Methods:**

The Italian Fragility Fracture Team appointed by the Italian National Health Institute was employed from January 2020 to February 2021 to (i) identify previously published systematic reviews and guidelines on the field, (ii) formulate relevant clinical questions, (iii) systematically review literature and summarize evidence, (iv) draft the Evidence to Decision Framework, and (v) formulate recommendations.

**Results:**

Overall, 351 original papers were included in our systematic review to answer six clinical questions. Recommendations were categorized into issues concerning (i) frailty recognition as the cause of bone fracture, (ii) (re)fracture risk assessment, for prioritizing interventions, and (iii) treatment and management of patients experiencing fragility fractures. Six recommendations were overall developed, of which one, four, and one were of high, moderate, and low quality, respectively.

**Conclusions:**

The current guidelines provide guidance to support individualized management of patients experiencing non-traumatic bone fracture to benefit from secondary prevention of (re)fracture. Although our recommendations are based on the best available evidence, questionable quality evidence is still available for some relevant clinical questions, so future research has the potential to reduce uncertainty about the effects of intervention and the reasons for doing so at a reasonable cost.

## Background

Fragility fractures result from mechanical forces that would not ordinarily result in fracture, known as low-level (or “low energy”) trauma (1). The World Health Organization (WHO) has quantified this as forces equivalent to a fall from a standing height or less (2).

Fragility fractures have garnered great attention as a public health concern. Worldwide, approximately 200 million women have osteoporosis and an increased risk of fragility fracture (3). It was estimated that 2.7 million new fragility fractures occurred in 2017 in the five largest EU countries (France, Germany, Italy, Spain, and UK) plus Sweden (overall referred to as EU6) (4).

The worrying burden of fragility fractures on individuals attributable to the high number of fracture-related annual losses of quality-adjusted (QALYs) and disability-adjusted life years (5–7), of sick days (8, 9), and of healthcare costs totalled an estimated €37.5 billion across the EU6 countries (10, 11). It should be emphasized that with aging populations, EU6 countries should expect increases in the number of fragility fractures (+23%), QALY loss (+26%), and fracture-related costs (+27%) from 2017 to 2030 (6).

There is now a substantial body of evidence that individuals who have already suffered a fragility fracture are at greater risk for further fractures (12–21), particularly in the 2 years following an initial fracture (22). This suggests that there is a potential for optimizing the benefits of secondary fracture prevention by recognition that it is due to fragility, rather than other causes, and treating patients as soon as possible after occurrence of a fracture (6). Nevertheless, the treatment gap (i.e., the proportion of patients who did not receive appropriate drug therapy), in EU6 in 2017 is estimated to be 73% for women and 63% for men (6). Compared with analysis from the year 2010, this indicates a marked increase from 56% in women and 47% in men (23, 24).

Given these premises, that is by considering that secondary prevention of fragility fracture is a huge concern for public health which needs to be addressed as a priority, the Italian National Health Institute, in accordance with the recently founded (2020) Italian Fragility Fracture Observatory (monitoring centre of the epidemiology of fragility fractures in Italy), encouraged the establishment of a working group to draft guidelines in this field (i.e., the Italian Guidelines for “Diagnosis, risk stratification and continuity of care of fragility fractures” (25)). The primary objective was to provide support so that healthcare professionals from several disciplines, including non-specialist physicians, nurses, and patients’ organizations, could make appropriate decisions to improve the outcomes of secondary fragility fractures in adherence with standards for trustworthy guidelines and the GRADE (Grading of Recommendations Assessment, Development and Evaluation) system (26, 27). The current guidelines cover a wide range of areas, including recognition of fragility as the cause of bone fracture, assessment of the risk (including the imminent risk) of secondary fractures, the choice, sequence and timing of drug therapy, and the management of clinical pathway.

The current manuscript is a translated summary of the full version of the Italian Guidelines for “Diagnosis, risk stratification and continuity of care of fragility fractures” (25). We hope that the worldwide audience of healthcare professionals and policymakers takes advantage of the Italian experience.

## Guideline development process

### Who contributed to guideline accomplishment: the Fragility Fracture Team

The Fragility Fracture Team (FFT) was made up of professionals appointed by speciality and primary care scientific societies and the National College of Nursing Professions, representatives of patients’ associations, in addition to a team of clinical epidemiologists and biostatisticians directly appointed by the Italian National Health Institute (please see Supplementary material, **Table S1**, for the complete list of experts involved).

FFT took office in January 2020 for establishing the team arrangement by assigning each member to one or more panels including (i) the executive committee (MLB, GC, SL, MR, UT) for leading the FFT, and for convocation, and coordination of plenary meetings; (ii) the evidence review team (AB, LC, DG, SM, EP, GP, MP, RR) for defining the clinical questions, developing the literature search strategies, querying the bibliographic databases, and assessing the quality of the evidence; (iii) the skilled/stakeholder panel whose members (GA, RA, RB, MLB, LC, DG, SG, GI, AL, SL, RM, SM, TN, MP, AP, EP, MR, UT) consulted the preliminary versions of the guidelines and expressed opinions, comments and viewpoints according to their own experience, and made recommendations for subsequent versions; and (iv) the quality assurance team (MLB, GC, SL, MR, UT) responsible for ensuring that the Guideline Development Process complied with methodological standards. FFT members met *via* webinar and corresponded through e-mail. Once the Guidelines were definitively drafted, a peer review was requested from two external experts (APC, BF). The final document was signed by all FFT members, submitted for its endorsement to the National Centre for Clinical Excellence, Healthcare Quality and Safety, and approved by the Italian National Health Institute in October 2021.

### Identifying previously published systematic reviews and guidelines

The GRADE-ADOLOPMENT approach (based on the GRADE EtD frameworks) was used to determine whether to develop a new guideline or adopt existing recommendations (28). Through databases developed by international health agencies (29–32), guidelines published in the last 10 years were preliminarily searched. Experts in the sector were also asked to report any other documents of interest. Only evidence-based guidelines ensuring editorial independence and reporting the adopted methods were included. Guidelines developed by regional, peripheral, or local agencies or bodies or by a single author and guidelines containing recommendations limited to a single intervention were excluded. In addition, systematic reviews on the issues of interest were also identified from those cited in guidelines, through the more widespread biomedical research databases (33, 34), and those reporting systematic reviews (35–37), as well as by means of hand-checking to identify additional relevant publications. Only systematic reviews published in the last 10 years were included. When data were published more than once, we considered the most recent and complete publication. Supplementary material, **Figure S1**, describes the results of the guideline/systematic review selection process. Overall, eight documents were selected (four guidelines and four systematic reviews) (38–45). Their critical analysis in terms of quality, topicality, and content was presented to the entire FFT. Because no document addressed the full spectrum of recommendations for secondary prevention of fragility fractures, the FFT opted to develop new recommendations, i.e., of developing the current guidelines.

### Formulating clinical questions

Topics to be considered in the current guidelines were established in a plenary session by the entire FFT. They covered three clinical issues, namely, (i) recognition of frailty as the cause of bone fracture, (ii) the (re)fracture risk assessment for prioritizing interventions, and (iii) the treatment and management of patients experiencing a fragility fracture. Clinical Questions (CQ) covering the abovementioned clinical issues were organized according to the PICO model against which we issued the recommendations (46). PICO stands for patient/population, intervention, comparison, outcome. The PICO questions were formulated by the skilled/stakeholder panel and the evidence review team.

### Systematically reviewing literature and building evidence synthesis

For each CQ, a literature search was conducted using PubMed/Medline, Embase, and the Cochrane Library (33–35), as well as original articles reported through guidelines and systematic reviews. All databases were queried, and specific search strategies were adopted for each CQ. A two-step procedure (i.e., article screening by title and abstract followed by review of entire main text) was performed in a double-blind fashion by the evidence review team. Discrepancies between readers were resolved in conference. The quality of each individual study included was evaluated using validated tools, such as the revised Cochrane ROB (risk of bias) for RCTs (randomized controlled trials) (47), the NOS (Newcastle–Ottawa Scale) for observational studies (48), and the QUADAS-2 (Quality Assessment of Diagnostic Accuracy Studies) for accuracy diagnostic studies (49).

After making a final decision regarding the quality of evidence and conducting the corresponding meta-analytic syntheses, the SoF (summary of finding) table was developed for each combination of CQ and outcome. The GRADE evidence profile table was consistently built (50). The GRADE quality assessment labels (i.e., high, moderate, low, and very low) were assigned to each outcome through five dimensions (risk of bias, consistency of effect, imprecision, indirectness, and publication bias).

### Evidence-to-Decision (EtD) Framework achievement

The process of moving from evidence to recommendations represents a cornerstone of guideline development (51, 52). Among the broad variety of criteria for consideration suggested by international organizations for reaching a decision (53, 54), those included in the most popular framework known as GRADE-EtD (55, 56) were adopted for building the current recommendations. Details about the development process of the GRADE-EtD framework are available elsewhere (57). Briefly, the GRADE-EtD framework aims to help panel members use evidence in a structured and transparent way to inform healthcare decisions and help guideline development teams consider the most relevant criteria influencing decisions by shaping discussions (58).

### Formulating recommendations

When a new systematic review was conducted, or when existing systematic reviews were evaluated and their results adapted, the FFT collaborated ahead of the recommendation decision according with the GRADE-EtD framework, developing drafts of the evidence for a decision table and recommendations’ text. Ratings for recommendation type and strength (i.e., 1 recommended/recommended against, 2 suggested/suggested against) together with GRADE quality assessment labels (i.e., A = high, B = moderate, C = low, and D = very low) were assigned. The balance of effects, values and acceptability, and feasibility were also considered. The manual from the Italian National System for Guidelines (National Health Institute 2019) (58) was referenced in developing the recommendations.

## Results

**Figure 1:**
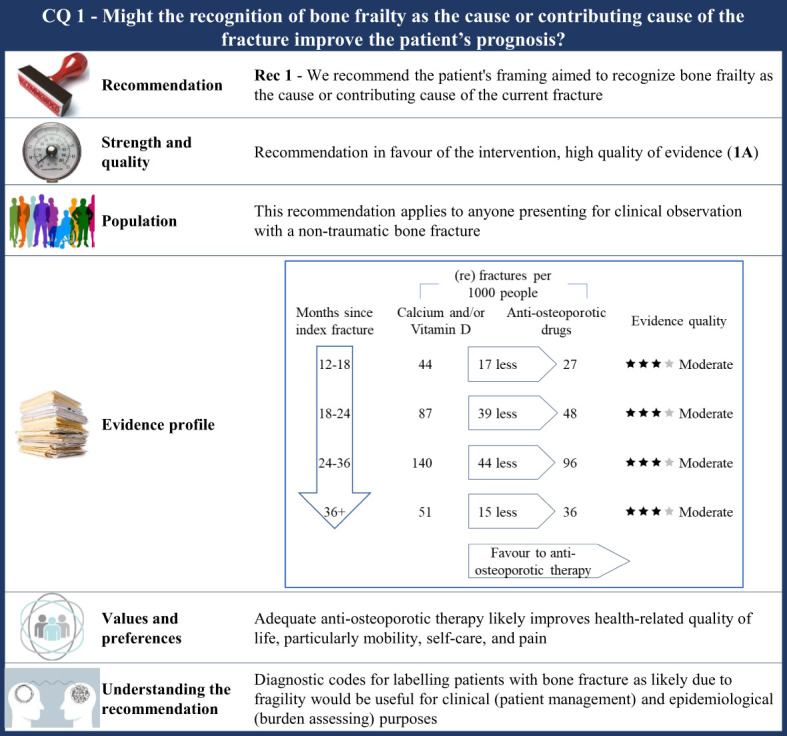
Visual summary for CQ1 (Might the recognition of frailty as the cause or contributing cause of the fracture improve the patient’s prognosis)?. Rationale. As ethical concerns hinder carrying out clinical studies by randomizing patients to obtain an adequate comparator (i.e., patients who have no tools to recognize bone frailty were included), the CQ was indirectly investigated. RCTs comparing outcome occurrence (refracture) among patients who received any anti-osteoporotic drug therapy and those who received calcium and/or vitamin D were included. The underlying assumption is that all the included patients were indicated for anti-osteoporotic drug therapy (i.e., the bone frailty was the cause or contributing cause of the current fracture), but some of them did not receive effective drug therapy (so surrogating those patients for whom no tools recognizing bone frailty are used, i.e., the comparator of interest). Through the updating of the most recently published systematic review on this issue (42), our systematic review included 46 RCTs (59–74, 89–104). Critical outcomes of interest pertained the rate of refracture at 12–18 months, 18–24 months, 24–36 months, and 3 years or more from the index fracture. Clinical benefits. Although the quality of evidence was moderate within each time category, a clear advantage favouring anti-osteoporotic drug therapy was observed. Between-rate absolute difference (RD) ranged from 15 to 44 (re)fractures avoided with therapy every 1,000 fractured patients, respectively, 36 months or more and 24–36 months after the index fracture. Values and preferences. Osteoporotic fractures have a negative impact on Health-related Quality of Life (HRQoL), particularly for mobility, self-care, and pain. Patients over 50 years of age treated with anti-osteoporotic therapy showed a significant improvement in HRQoL at 24 months (105). Increased quality of life as detected by the QUALIOST questionnaire was obtained through treatment of postmenopausal women (90), although no significant differences were found for the Short-Form or SF-36. At last, a higher Osteoporosis Quality of Life Scale score was reached after 12 months of drug therapy (99). Understanding the recommendation. The FFT noted that there were strong clinical benefits associated with anti-osteoporotic therapy and, consequently, agreed to upgrade up to high evidence quality despite the moderate certainty evidence. A combination of the evidence, values, and preferences also contributed to the strong recommendation in favour of the anti-osteoporotic treatment in patients with fragility fractures. Diagnostic codes for label patients who have bone fracture is likely due to fragility would be useful for clinic (patients’ managing) and epidemiologic (burden assessing) purposes.

**Figure 2:**
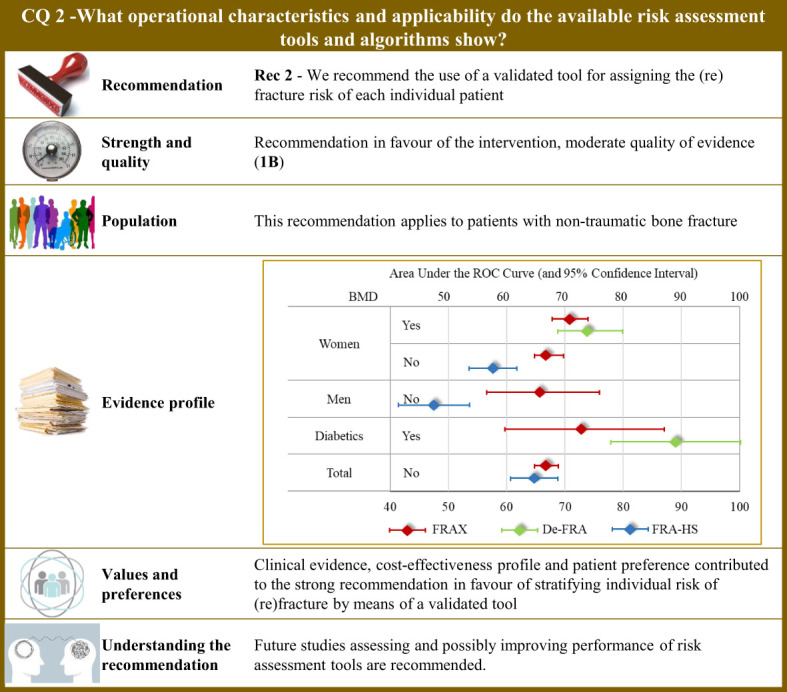
Visual summary for CQ2 (What operational characteristics and applicability do the available risk assessment tools and algorithms show)?. Rationale. The most common tool used worldwide for assessing the fracture risk is the so-called FRAX^®^, which was developed at the University of Sheffield, United Kingdom, and is based on individual patient models that integrate the risk associated with several individual features (i.e., gender, age, body mass index, personal history of fragility fracture; parental history of proximal femur fracture; current smoking status; prolonged use of glucocorticoids; rheumatoid arthritis; secondary causes of osteoporosis; and alcohol consumption ≥ 3 units per day), with or without including bone mineral density at the femoral neck (106). The model was externally validated (107) and calibrated from country-specific fracture data covering more than 80% of the world population (108). The FRAX^®^ algorithm give the 10-year probability of fracture (106). Although the FRAX^®^ tool is the most popular predictive tool, it nevertheless presents some application concerns, and above all access problems for regulatory use. For this reason, national versions have been developed such as the QFracture algorithm to predict risk of osteoporotic fracture in primary care in the UK (109). In Italy, three algorithms have been developed, nominally: (i) the DeFRA developed by the Italian Society for Osteoporosis, Mineral Metabolism and Bone Diseases in collaboration with the Italian Society of Rheumatology (110), and made available online (111); (ii) its updated version (DeFRAcalc79) defined according to drug reimbursement rules from the Italian Drug Agency (112); (iii) and the FRActure Health Search (FRA-HS) developed by the Italian Society of General Medicine and Primary Care (113). As studies directly comparing reliability and applicability of available tools are lacking, a systematic revision of literature was carried out for obtaining and comparing meta-analytic estimates of discriminatory powers through the AUC (area under the receiver operating curve) (114). Through the updating of the NICE guidelines (115), our systematic review included 47 original papers investigating operative characteristics of FRAX^®^ (116–162) and added three papers pertaining Italian instruments (two for DeFRA (163, 164) and one for FRA-HS (165)). Operative characteristics pertain the 10-year predicted and observed fracture risk (major osteoporotic or proximal femur) in all the included papers. Tools performance. Meta-analytic AUC estimates (and 95% confidence intervals) for FRAX were 0.66 (0.57 to 0.76) and 0.67 (0.65 to 0.70) in women and men, respectively. By including body mineral density among the considered items, the AUC of FRAX^®^ improved to 0.71 (0.68 to 0.74) and 0.73 (0.60 to 0.87) in women and diabetics, respectively. DeFRA had better performance than FRAX^®^ for both women (0.74, 0.69 to 0.80) and diabetics (0.89, 0.78 to 1.00). Conversely, FRA-HS discriminated worse than other tools with the AUC estimates 0.58 (0.54 to 0.62) and 0.48 (0.42 to 0.54) in women and men, respectively. Clinical and value issues. Ten-year fracture risk perceived by patients and that estimated by the predictive tool (specifically by FRAX^®^) was found to be highly disagreeing among patients at high fracture risk, women, elderly, and patients treated with anti-osteoporotic medications or calcium/vitamin D (166), thus making implementation of fracture prediction tools in clinical practice highly to be hoped for. Efficient screening strategies may support fragility fracture prevention as shown by a Sweden study that administered the FRAX^®^ to postmenopausal women *via* e-mail, online or screening mammography (167). Patients at high risk of fracture should be earlier identified to reduce mortality, comorbidities, and costs (177). Suitable cost-effectiveness profiles for fracture risk screening (169), and for consequent therapy of high-risk patients with any anti-osteoporotic treatment (170–176), and other drugs (176) were consistently reported from several European countries. Understanding the recommendation. Although clinical evidence, cost-effectiveness profile, and patient’s preference contributed to the strong recommendation in favours of stratifying the individual risk of (re)fracture, concerns persist about quality of evidence of studies investigating predictivity of available tools, including FRAX^®^. Although FRAX^®^ might be used in any healthcare settings (168, 177), cautions should be taken in its use in specific countries by adopting tools built and validated in the target population. Future studies assessing and possibly improving performance of risk assessment tools are recommended.

**Figure 3:**
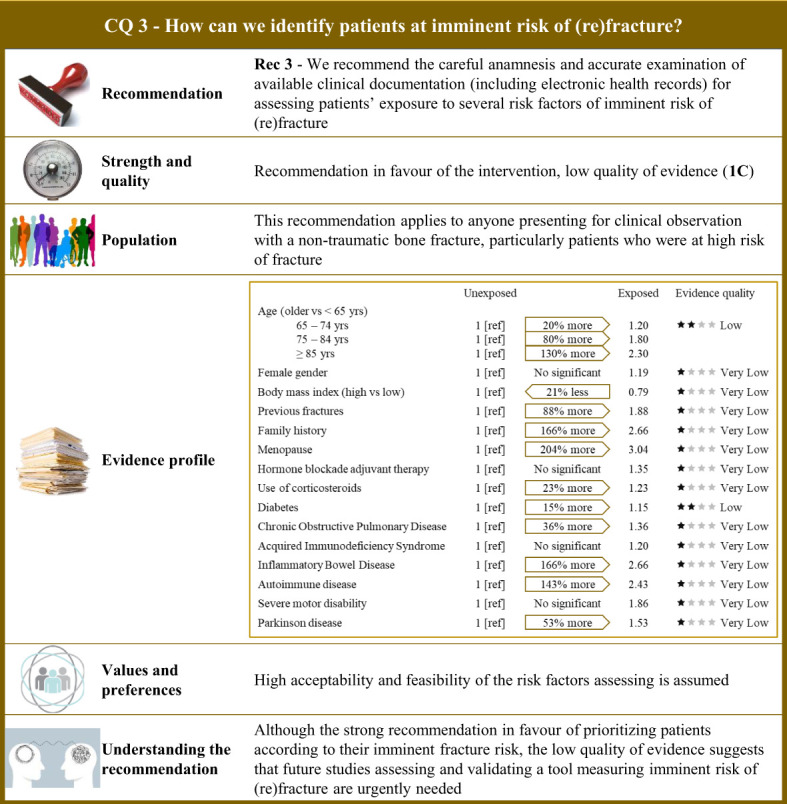
Visual summary for CQ3 (How can we identify patients at imminent risk of (re)fracture)?. Rationale. As the risk of bone fracture after experiencing a fracture is on average doubled in the 2 years that follow (11, 13, 16, 178–185), a period which has been defined “imminent,” particular attention should be placed on identifying patients at higher risk of imminent (re)fractures (10, 186–188). Our systematic review included 46 observational studies comparing the risk of imminent (re)fracture of patients who were exposed and not exposed to a series of potential risk factors (18, 181, 186, 187, 189–230). Risk factor profile. Among the 15 factors included, 11 showed evidence of increasing the imminent risk of fracture, with relative risk excess ranging from 20% (acquired immunodeficiency syndrome) to 204% (menopausal status). Values and preferences. High acceptability and feasibility of the assessment risk factors is assumed. Understanding the recommendation. Despite the strong recommendation in favour of prioritizing patients according to their imminent fracture risk, uncertainty due to serious/very serious risk of bias of observational designs and imprecision and inconsistency of estimates strongly affected the quality of evidence. Future studies assessing and validating a tool measuring imminent risk of (re)fracture are therefore urgently needed.

**Figure 4:**
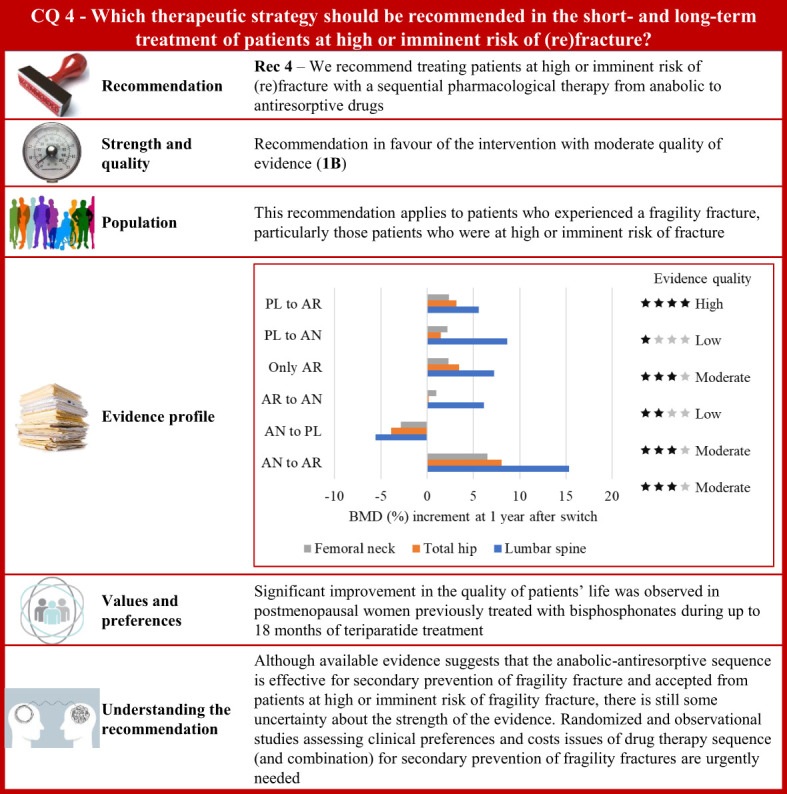
Visual summary for CQ4 (Which therapeutic strategy should be recommended in the short- and long-term treatment of patients at high or imminent risk of (re)fracture)?. Rationale. Nowadays, pharmacological options have been developed for the treatment of osteoporosis and fragility fractures. Bisphosphonates and denosumab are potent antiresorptive drugs (AR). In particular, denosumab is a fully monoclonal antibody, directed against the receptor activator of nuclear factor-κB ligand that inhibits the differentiation, activation, and survival of osteoclasts (231). Anabolic drugs (AN) are teriparatide and abaloparatide (the latter not yet available in Italy). These treatments, typically taken intermittently, act through the parathyroid hormone receptor and stimulate osteoblast activity for bone formation. Romosozumab is the newer anabolic drug made available (also in Italy) (232), acting with a dual mechanism of action since it stimulates bone formation and inhibits bone resorption (231). Anabolic treatment is time-limited (12 to 24 months) and consequently its beneficial effects against bone loss, which could increase the fracture risk. For this reason, treatment that includes antiresorptive agents should be considered (231). However, the optimal treatment strategy for fracture needs to be identified based on sequential or combined therapies whose clinical efficacy should be assessed according with their antifracture potential and harm profile (231–233). Our systematic review included 17 RCTs (234–250). Between-arm comparison of bone mineral density changes and the fracture risk during follow-up were the critical outcomes of interest. Clinical benefits. Only one study compared bone mineral density changes among patients randomized to the anabolic–antiresorptive sequence or vice versa (241). Increasing values of bone mineral density were obtained for the entire observation time-window (that is, from baseline to 24 months after the drug switch was scheduled, and 24 months after the start of the second sequential drug) for all the considered bone sites. Conversely, among patients who started with an antiresorptive medication, decreasing values of bone mineral density were observed once the switch was made to an anabolic medication, suggesting that anabolic drugs in the second phase can compromise the effect of antiresorptive taken initially. Several other comparisons are available, but the corresponding findings (available in the **Supplementary material**) do not offer clear evidence at favour of a specific sequence. The risk of fracture was found to be lower 12 and 24 months after switching from anabolic to antiresorptive compared with both placebo (PL)–antiresorptive sequence (235, 242), or treatment with alendronate only (245, 250). Twelve months after switching from placebo to anabolic (teriparatide) the risk of fracture was found to be lower than switching from antiresorptive (bisphosphonate) to anabolic teriparatide (240). The certainty for fracture risk was moderate because of risk of bias and imprecision. Values and preferences. A prospective, multicentre observational study conducted in eight European countries (Austria, Denmark, France, Germany, Greece, Ireland, The Netherlands, and Sweden) after teriparatide was approved by regulatory agencies reported that postmenopausal women previously treated with bisphosphonates had significant improvement in HRQoL during up to 18 months of teriparatide treatment (251). Understanding the recommendation. Although available evidence suggests that the anabolic–antiresorptive sequence is effective for secondary prevention of fragility fracture and accepted from patients at high or imminent risk of fragility fracture, there is still some uncertainty about the strength of the evidence. Randomized and observational studies assessing clinical preferences and cost issues of drug therapy sequence (and combination) for secondary prevention of fragility fractures are however urgently need.

**Figure 5:**
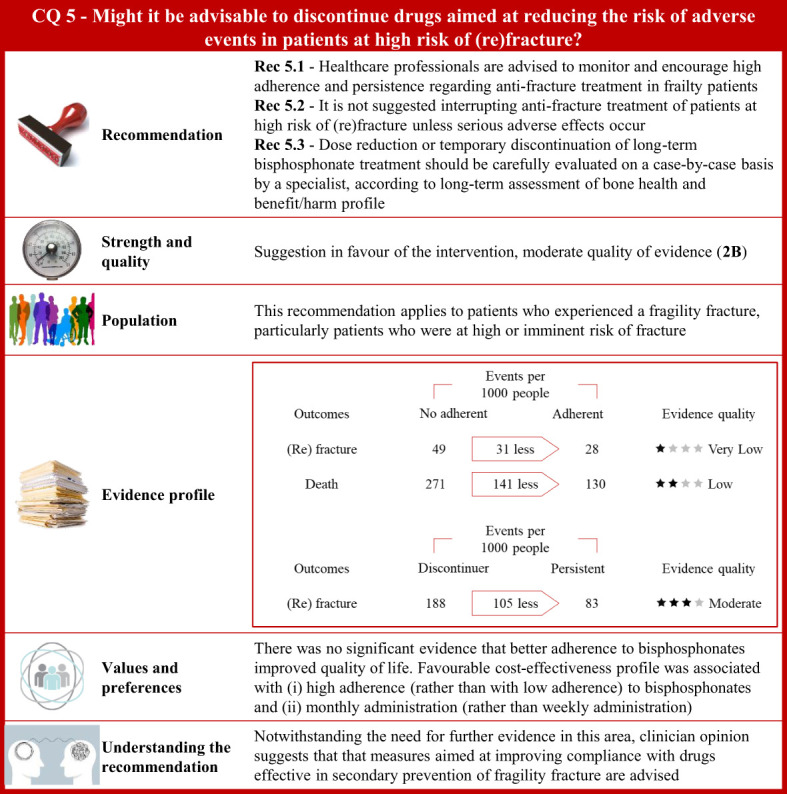
Visual summary for CQ5 (Might it be advisable to discontinue drugs aimed at reducing the risk of adverse events in patients at high risk of (re)fracture)?. Rationale. Secondary fragility fractures may be prevented by improving long-term adherence to anti-osteoporotic drugs (252–254). However, low adherence could be induced by adverse events (255–257), inadequate drug dosage regimens (255, 257), or asymptomatic disease (255, 256). Less frequent or intermittent dosing schedules may promote medications adherence to long-term therapies and improve health outcomes in postmenopausal women (74, 258). Through updates of the most recently published systematic reviews on this issue (259–261), and specific manual research, our systematic review included 15 publications investigating the association between continuity of treatment (persistence and adherence) and several outcomes (changes in bone mineral density and fracture risk) in patients with fragility fracture (62, 74, 252, 258, 262–272). Clinical benefits. Three comparisons evaluated the medication vacation in patients with osteoporosis, nominal adherence vs. no adherence, persistence vs. discontinuity, and continuous vs. cyclical treatment. Adherence was defined by the number of doses dispensed with respect to the observation time and calculated by the medication possession ratio (MPR). A reduced risk of vertebral (risk ratio 0.74; 95% confidence interval 0.60 to 0.91), non-vertebral (0.42; 0.20 to 0.87), or any fracture (0.82; 0.72 to 0.93) was detected among patients with MPR greater than 80% compared to non-adherent subjects. Adherence was associated with lower mortality (0.47; 0.35 to 0.64). Reduced vertebral fracture risk (0.81; 0.66 to 0.99) was found among patients who adhered to therapy for more than 12 months with respect to those whose adherence was less than 12 months. Persistence was defined by at least 30 days of drug therapy interruption. Reduced vertebral fracture risk was found in persistent patients compared to discontinuers (0.85; 0.75 to 0.96). No significant decreased risk of vertebral, non-vertebral, or any fracture was detected among patients who persisted with therapy for more than 12 months compared to patients who persisted for less than 12 months. Consistently, there was no evidence of a reduced mortality risk. Extension trials were included in this comparison. Patients randomized to receive placebo or anti-osteoporotic drugs after 5 years were re-randomized to respectively receive anti-osteoporotic drugs or placebo. A reduced risk of non-vertebral fracture was associated with the continuous treatment (0.37; 0.26 to 0.54), whereas there was no evidence of decreased risk of vertebral or any fracture. There was no reduced risk of adverse events associated with the continuous anti-osteoporotic therapy. Finally, studies that randomly assigned patients to daily anti-osteoporotic therapy or cyclical treatment found no statistical evidence of difference in fracture risk and adverse events (upper gastrointestinal or oesophageal disorders). Quality of evidence was low for studies investigating MPR and persistence, and moderate for studies balancing fracture risk and adverse events. Values and preferences. Only one RCT compared patients who had long-term adherence to oral bisphosphonate with those who did not adhere to therapy, without finding any significant difference in health-related quality of life at baseline and 12–24 months afterward (270). Postmenopausal women discontinued anti-osteoporotic treatment due to drug-related/fear of side effects or insufficient motivation (273). Poor compliance was related to benzodiazepine and gastroprotective use, whereas persistence to treatment was higher in patients with previous vertebral fractures, early menopause, or low bone mass values or treated with corticosteroid or anti-inflammatory medications. Higher education level and disease awareness were associated with better adherence to long-term treatment with alendronate, whereas onset of new diseases had induced treatment interruption (274). Medication routes of administration may directly influence adherence. Specifically, self-administered teriparatide injection was well tolerated (275). Subcutaneous injection of parathyroid hormone was shown to be correctly administered to elderly patients with trochanteric hip fracture (276). Treatment adherence to denosumab administered subcutaneously every 6 months was greater than adherence to oral alendronate taken once a week in postmenopausal women (277). Conversely, a Chinese study showed that patients with a history of fracture had a stronger preference for weekly oral tablets compared to other modes of administration such as annual intravenous infusion or 6-month subcutaneous injection (278). Finally, there was no statistical evidence of differences of administration route preference in patients undergoing a standardized educational session regarding the pathophysiology of osteoporosis and its complications (279). Although poor compliance might be associated with reduced clinical benefits and increased mortality (280), it would not necessarily affect the actual cost per fracture avoided (281). Based on model estimates, more fractures were avoided with monthly bisphosphonate (58.1 per 1,000 treated women) than with weekly bisphosphonates (33.8 per 1,000 treated women), resulting in lower incremental cost per QALY gained (282). Costs per QALY gained were estimated to increase with higher adherence to oral bisphosphonates whereas poor compliance would result in a decreased cost-effectiveness of drug therapy (283). Included studies might have limited generalizability across different countries because of distinctive resources and prioritized treatment options available in various healthcare settings. Understanding the recommendation. Notwithstanding questionable quality of available evidence, clinician opinion suggests that measures aimed at improving compliance with drugs effective in secondary prevention of fragility fracture are advised.

**Figure 6:**
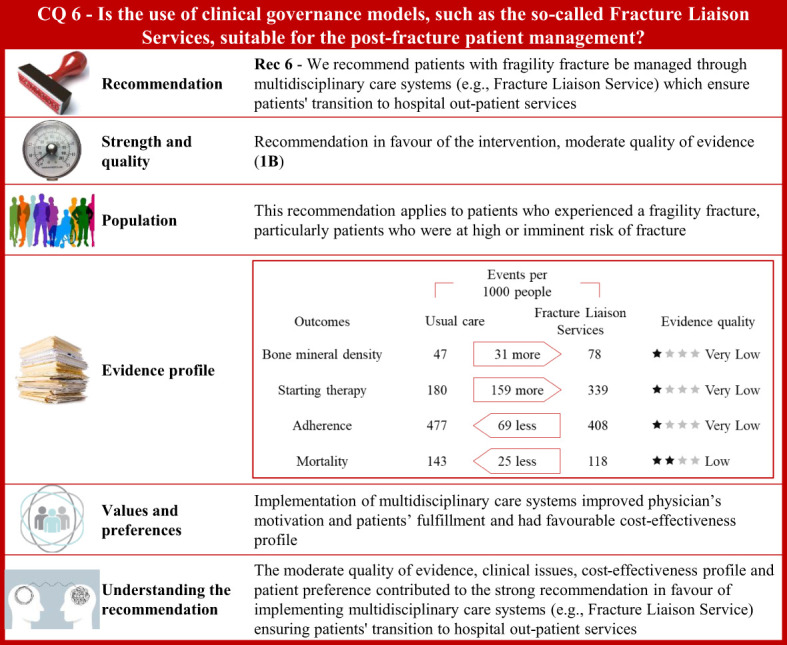
Visual summary for CQ6 (Is the use of clinical governance models, such as the so-called Fracture Liaison Services, suitable for patients’ post-fracture management)?. Rationale. Patients who experienced a fragility fracture should receive correct care planning after hospital discharge to ensure continuity of care through shared diagnostic-therapeutic pathways (284). According to the Fragility Fracture Network (285), global multidisciplinary collaborations should be carried out to improve the care of patients with fragility fractures (286). The Fracture Liaison Service (FLS) is a model of care designed to prevent recurrent fractures (44, 287–289) with coordinated strategies (290) and achieve optimal adherence to anti-osteoporotic medications (291, 292). The multidisciplinary team should be formed by the bone specialist (FLS coordinator), the orthopaedic surgeon, and the specialized bone nurse (43, 293, 294). Through updates of the most recently published systematic reviews on this issue (290, 295–298), and specific manual research, our systematic review included 77 publications on multidisciplinary care systems, such as nurse-led clinics, structured service delivery models, and FLS (299–375). Bone mineral density values, anti-osteoporotic therapy initiation, adherence to anti-osteoporotic therapy, and (re)fracture and mortality risk were the outcomes of interest. Clinical benefits. Compared with usual care, the multidisciplinary programs significantly increased body mineral density values, initiation to anti-osteoporotic treatment, and adherence to anti-osteoporotic therapy. Moreover, these coordinated models showed a reduction of fractures and a significant decrease in mortality risk. Quality of evidence was very low for body mineral density values, initiation to anti-osteoporotic treatment, and adherence to anti-osteoporotic therapy, and low for mortality rate. Values and preferences. Physicians’ motivation in implementing FLS is justified given barriers in treating fractures, gaps in osteoporosis knowledge, and difficulty in managing patients presenting with a fragility fracture observed by healthcare professionals (376). Chinese orthopaedic surgeons reported low sensitivity to the concept of fracture prevention as well as in the effectiveness of preventive measures for fragility fractures (377). Only 25% of patients who contacted their physician received anti-osteoporotic treatment, according to an RCT (378). Conversely, 61% of subjects with a low-trauma fracture were treated with an anti-osteoporotic medication according to a study investigating performance of FLS (Yates 2015) (379). However, the included studies considered various healthcare settings; thus, resource requirements might have limited generalizability across different countries. A decreased refracture risk and favourable cost-effectiveness profile of FLS models compared to usual care was reported from a systematic review evaluating FLS programs in the Asia-Pacific region (298). Compared to usual care or no treatment, FLS resulted in a favourable cost-effectiveness profile from another systematic review (Wu 2018) including osteoporotic patients aged 50 and above from Canada, Australia, the US, UK, Japan, Taiwan, and Sweden (380). FLS-based management of fragility fractures had been reported to be cost-effective in Canada with the reduction of subsequent hip fractures and a net hospital cost savings (381). The implementation of hip FLS co-managed by a nurse and physician showed a $54 incremental cost/patient with a modest gain of eight QALYs/1,000 patients (382). For every 10,000 patients that participated in FLS, an additional 400 patients would be treated with bisphosphonates, resulting in the avoidance of around four hip fractures. Furthermore, the proportion of patients who appropriately received bisphosphonate treatment increased in the year following fracture, from 4.3% to 17.5% (383). The FLS implementation in the USA resulted in 153 fewer fractures, 37.4 QALY gained, and $66,879 in cost savings for every 10,000 patients (384). An FLS implemented in UK was estimated to prevent at least 18 fractures and save £21,000 for every 1,000 patients (385). FLS organizations ensure that patients, affected by osteoporosis or fractures, receive appropriate evaluation and treatment (386, 387) although failures in entry registration, male gender, frailty, education level, living alone, or lack of motivation could be independent factors for FLS non-attendance (388). Additional potential barriers include lack of communication between patients and physicians or the need for patient education intervention (389). Some patients might refuse treatment because of concerns with costs or side effects. Thus, person-centred care should support the interaction between patients and healthcare professionals (389). Understanding the recommendation. The moderate quality of evidence, clinical issues, cost-effectiveness profile, and patient’s preference contributed to the strong recommendation in favour of implementing multidisciplinary care systems (e.g., Fracture Liaison Service) ensuring patients’ transition to hospital outpatient services.

Overall, 351 original papers were included in our systematic review (10, 11, 13, 16, 18, 42–44, 59–74–389), selected to answer six clinical questions. One of the six (CQ1) refers to the issue concerning frailty recognition as the cause of bone fracture (Might the recognition of frailty as the cause or contributing cause of fracture improve the patient’s prognosis)?. Two of the six questions (CQ2 and CQ3) refer to the issue concerning (re)fracture risk assessment for prioritizing interventions (What operational characteristics and applicability do the available risk assessment tools and algorithms show? and How can we identify patients at imminent risk of (re)fracture? Three of the six questions (CQ4, CQ5, and CQ6) refer to the issue concerning the treatment and management of patients experiencing fragility fracture (Which therapeutic strategy should be recommended in the short- and long-term treatment of patients at high or imminent risk of (re)fracture? Might it be advisable to discontinue a drug aimed at reducing the risk of adverse events in a patient at high risk of (re)fracture? Is the use of clinical governance models, such as the so-called Fracture Liaison Services, suitable for the post-fracture patient’s management)?. For each CQ, we have formulated one to three recommendations, which are synthesized in the corresponding visual summaries (**Figures 1**–**6**) whose footnotes report a broad and detailed description of rationale, clinical benefits, values and preferences, and understanding recommendations. Moreover, specific sections related to (i) search strategies, (ii) study selection flowchart, (iii) complete meta-analytic results, (iv) quality of evidence, and (v) SoF are reported for each CQ in the **Supplemental Material**.

Briefly, we recommend (i) recognizing bone fragility as the cause or contributing cause of the current fracture, (ii) measuring the individual (re)fracture risk using a validated tool, (iii) assessing the patient’s exposure to several factors associated with imminent (re)fracture risk, (iv) using a sequential pharmacologic scheme from anabolic to antiresorptive drugs, mainly in patients at higher/imminent risk of fracture, (v) avoiding treatment interruption, except for serious adverse events that occur, and (vi) implementing multidisciplinary care systems (e.g., Fracture Liaison Service), for ensuring patients’ transition to hospital outpatient services. Of these six recommendations, one was of high quality, another one of low quality, the remaining four being of moderate quality.

## Perspectives

From now on, as current delivery of secondary fracture prevention globally is lamentably suboptimal and taking into account the availability of guideline-based recommendations, the key challenge facing us all is how to ensure that guidelines-based care becomes usual care. The promotion of widespread awareness of the new guidelines, must necessarily be accompanied by a robust evaluation plan aimed of (i) monitoring the quality of services for secondary fracture prevention (are we providing healthcare according to recognized quality standards? what critical issues arise)? and (ii) assessing their impact (how and how much the guidelines adoption prevents the occurrence of secondary fractures and improve quality of life of patients? at what cost)?. In this regard, the combination of national clinical care standards and registries to enable benchmarking against such standards provides an opportunity to undertake so-called “real-world data” analyses for monitoring the changes and assessing the impact of usual care. There are currently 20 national hip fracture registries established worldwide, and the China National Hip Fracture Registry is at an advanced stage of development (390). Furthermore, there are currently national FLS registries at various stages of development in Australia and New Zealand (391, 392), Ireland (393), the UK (394), and USA (395). Several “real-world” evidence from the UK National Hip Fracture Database and Best Practice Tariff for hip fracture care have provided valuable insights (396–398). In Italy, the “real-world” monitoring changes and assessing impact of the new guidelines will be made possible by the “Italian Fragility Fracture Observatory,” a structure recently founded for bridging the gap between health institution and academy in generating knowledge (399) in the field of fragility fractures.

## Conclusion

The current guidelines provide guidance to support individualized management of patients experiencing non-traumatic bone fracture aimed of secondary prevention of (re)fracture. Although our recommendations are based on the best available evidence, questionable quality evidence is still available for some relevant clinical questions, so future research has the potential to reduce uncertainty about the effects of intervention and the reasons for doing so at a reasonable cost.

## Author contributions

All authors contributed to the preparation of the guidelines, all participated in the data collection, drafting, writing and editing the manuscript. Concept and design: MLB, GC, SL, MR, UT. Acquisition, analysis, or interpretation of data: AB, LC, DG, SM, EP, GP, MP, RR. Statistical analysis: AB, GP, RR. MLB, GC, SL, MR, UT take responsibility for the integrity of the data and the data analysis. The external experts APC, BF peer reviewed the guidelines. All authors contributed to the article and approved the submitted version.
