# Supplementary material for: Executive summary: Italian guidelines for diagnosis, risk stratification, and care continuity of fragility fractures 2021
Source: Front Endocrinol (Lausanne). 2023 Apr 18;14:1137671. doi: 10.3389/fendo.2023.1137671 (PMC10151776; doi:10.3389/fendo.2023.1137671)
Supplement: Supplementary file 1 [file DataSheet_1.pdf]

Supplemental Material

Table S1. Complete list of experts involved ... 1
Figure S1. Guideline / Systematic review selection process ... 3
CQ 1 ... 4
Search strategy ... 4
Flow chart ... 10
Results ... 11
Quality evaluation ... 14
Summary of Findings ... 16
CQ 2 ... 17
Search strategy ... 17
Flow chart ... 21
Results ... 22
Quality evaluation ... 35
Summary of Findings ... 38
CQ 3 ... 50
Search strategy ... 50
Flow chart ... 74
Results ... 75
Quality evaluation ... 82
Summary of Findings ... 86
CQ 4 ... 94
Search strategy ... 94
Flow chart ... 98
Results ... 99
Quality evaluation ... 102
Summary of Findings ... 103
CQ 5 ... 108
Search strategy ... 108
Flow chart ... 115
Results ... 116
Quality evaluation ... 120
Summary of Findings ... 122
CQ 6 ... 127
Search strategy ... 127

Flow chart 133
Results 134
Quality evaluation 138
Summary of Findings 140

Table S1. Complete list of experts involved

Fragility Fracture Team

Giovanni Adami	*Member of SIOMMMS - Società Italiana di Osteoporosi del metabolismo minerale e delle malattie dello scheletro*
Rosaria Alvaro	*Associate Professor in Nursing Sciences – University of Rome Tor Vergata*
Annalisa Biffi	*Department of Statistics and Quantitative Methods; Healthcare Research & Pharmacoepidemiology Interuniversity Center, University of Milan-Bicocca*
Riccardo Bogini	*General Pratictioner at USL Umbria*
Maria Luisa Brandi	*Full Professor of Endocrinology and Metabolic Bone Diseases - University of Florence; Director of the Regional Referral Center for Hereditary Endocrine Tumors; Director Clinical Unit on Metabolic Bone Disorders - University Hospital of Florence; President of FIRMO - Fondazione Italiana Ricerca sulle Malattie dell'Osso*
Achille Patrizio Caputi	*Emeritus Professor of Pharmacology; University of Messina*
Luisella Cianferotti	*Associate Professor of Endocrinology, University of Florence; member of FIRMO - Fondazione Italiana Ricerca sulle Malattie dell'Osso*
Giovanni Corrao	*Full Professor of Medical Statistics – University of Milan-Bicocca; Director of Healthcare Research & Pharmacoepidemiology Interuniversity Center*
Bruno Frediani	*Full Professor of Rheumatology; Director of the School of Specialization in Rheumatology - University of Siena; Director of the Complex Operational Unit in Rheumatology; Director of the Department of Medical Sciences*
Davide Gatti	*Associate Professor of Rheumatology – University of Verona; President of the Scientific Committee of the ASITOI - Associazione Italiana Osteogenesi Imperfetta; Coordinator of the Guideline Commission SIOMMMS - Società Italiana di Osteoporosi del metabolismo minerale e delle malattie dello scheletro*
Stefano Gonnelli	*Full Professor of Internal Medicine and Director of the School of Specialization in Iternal Medicine - University of Siena*
Giovanni Iolascon	*Full Professor of Physical and Rehabilitation Medicine – University of Campania "Luigi Vanvitelli"*
Andrea Lenzi	*Full Professor of Endocrinology - University of Rome La Sapienza; member of SIE – Società italiana di Endocrinologia*
Salvatore Leone	*Member of AMICI Onlus - Associazione nazionale per le Malattie Infiammatorie Croniche dell'Intestino*
Raffaella Michieli	*National Secretary SIMG – Società italiana di medicina generale e delle cure primarie*
Silvia Migliaccio	*Member of SIE – Società italiana di Endocrinologia; Associate Professor - University of Rome Foro Italico*
Tiziana Nicoletti	*Manager of CnAMC - Coordinamento nazionale delle Associazioni dei Malati Cronici e rari di Cittadinanzattiva*
Marco Paoletta	*Member of SIMFER - Società Italiana di Medicina Fisica e Riabilitativa*
Annalisa Pennini	*Member of FNOPI - Federazione Nazionale degli Ordini delle Professioni Infermieristiche per il progetto Fratture da Fragilità*
Eleonora Piccirilli	*Department of Orthopedics and Traumatology, University of Rome Tor Vergata*
Gloria Porcu	*Department of Statistics and Quantitative Methods; Healthcare Research & Pharmacoepidemiology Interuniversity Center, University of Milan-Bicocca*

Raffaella Ronco	*Department of Statistics and Quantitative Methods; Healthcare Research & Pharmacoepidemiology Interuniversity Center, University of Milan-Bicocca*
Maurizio Rossini	*Full Professor of Rheumatology; President of SIOMMMS - Società Italiana dell'Osteoporosi, del Metabolismo Minerale e delle Malattie dello Scheletro; Member of SIR - Società Italiana di Reumatologia*
Umberto Tarantino	*Full Professor of Diseases of the Locomotor System – University of Rome; Member of SIOT – Società italiana di ortopedia e traumatologia*

Figure S1. Guideline / Systematic review selection process

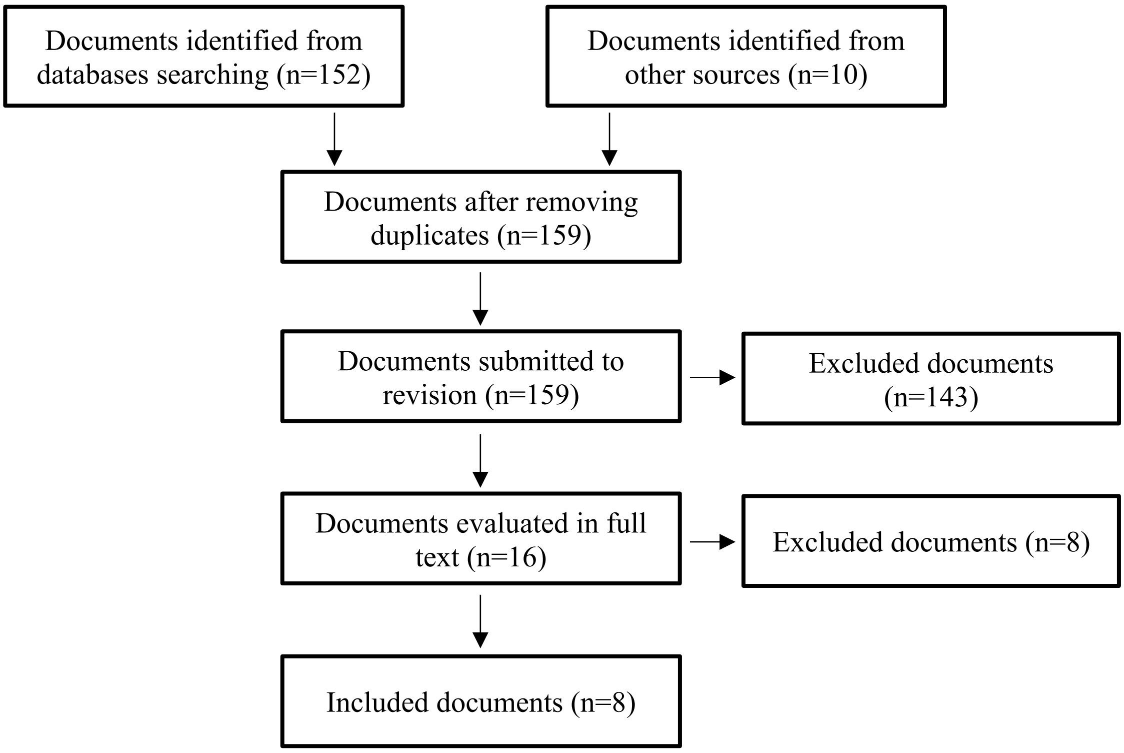

CQ 1

Might the recognition of frailty as the cause or contributing cause of the fracture improve the patient's prognosis?

Search strategy

MEDLINE

Up to 20 October 2020

#1:

(((wrist* or colles or radius or articulatio radiocarpea or carpus or carpal or radiocarp* or radial or forearm* or humerus or metacarp* or barton or monteggi* or ulna or ulnar or upper limb* or hip or hips or trochanteric or intertrochanteric or subtrochanteric or femoral neck or femur neck or spine or spinal or vertebra or vertebral or vertebrae or lumbar or shoulder* or glenohumeral or humeroscapular or scapulo humeral or proximal humeral) adj3 fractur*) or (exp hip fractures/ or spinal fractures/ or shoulder fractures/ or osteoporotic fractures/ or exp radius fractures/) or (fractures, bone/ and (exp wrist joint/ or exp spine/ or shoulder/ or shoulder joint/ or hip/))) and (exp osteoporosis/ or (osteoporo* or bone loss*))

#2:

“fragility fracture”[ti] OR “fragility fractures”[ti] OR “low energy fracture”[ti] OR “low energy fractures”[ti] OR “low-energy fracture”[ti] OR “low-energy fractures”[ti] OR “low trauma fracture”[ti] OR “low trauma fractures”[ti] OR “low-trauma fracture”[ti] OR “low-trauma fractures”[ti] OR “low energy trauma”[ti] OR “low-energy trauma”[ti] OR “low level trauma”[ti] OR “low-level trauma”[ti] OR “minor trauma fracture”[ti] OR “minor trauma fractures”[ti] OR “minor-trauma fracture”[ti] OR “minor-trauma fractures”[ti] OR “minor fracture”[ti] OR “minor fractures”[ti] OR “minor-fracture”[ti] OR “minor-fractures”[ti] OR “osteoporotic fracture”[ti] OR “osteoporotic fractures”[ti]

#3: #1 OR #2

#4:

exp bone density conservation agents/ or exp diphosphonates/ or exp calcitonin/ or exp selective estrogen receptor modulators/ or exp raloxifene hydrochloride/ or exp teriparatide/ or (exp antibodies, monoclonal/ and exp rank ligand/) or (aclasta or actonel or alend or alendro* or alovell or amgiva or aminodron* or aminobutane* or aminohexane* or aminohydroxy* or aminomux or aminopropane* or aminopropylidene* or aredia or aredronet or arendal or atelvia or belfosdil or benet or bifemelan or bifosa or binosto or bisphonal or bisphosphon* or bonapex or bondenza or bondronat* or bonefos

or boniva or bonmax or bonviva or butedron* or calcitar or calciton* or calcitrin or cangrelor or celvista or cibalcin or cimadron* or clodron* or coldron* or cycloheptylaminomethylenebis or defixal or denosumab or dequest or destara or diadronel or dichlorometh* or didronal or didronat* or didronel or difosfonal or difosfen or dinol or diphos or diphosphon* or dronal or dronate or editron* or ehdp or endronax or ethane* or ethylenehydroxy* or ethylidenebisphosphon* or etibon or etidron* or eucalen or evista or fixopan or forsteo or forteo or fosalan or fosamax or fosmin or fosval or hedp or hexane* or hydroxyeth* or hydroxyhex* or hydroxyl* or iasibon or ibandron* or incadron* or kengreal or kengrexal or keoxifene or lidadronate or lodronat* or loxar or loxifen or marvil or maxibone or mebonat or medron* or medrotec or methane* or methanon* or methylene* or minodron* or neobon or neridron* or nerixia or olpadron* or oncalst or onclast or optinate or optruma or orazol or osdron or osdronat or oseotenk or osficar or oslene or ossiten or ostac or osteof* or osteopam or osteopor or osteosan or osteotop or osteovan or osticalcin or pamidronate or pamisol or panolin or parathar or parathormone* or parathyroid hormone* or porosal or prolia or propane* or propylidenediphosphon* or raloxifene or raxeto or reclast or ribastamin or risedron* or serm or serms or skelid or staporos or teiroc or teriparatide or thyreocalciton* or thyrocalciton* or tiludron* or tibolene or turpinal or voroste or xgeva or xidifon or xidiphone or xydiphon* or zoledron* or zomera or zometa or abaloparatide or “strontium ranelate” or bazedoxifene) or (bone resorpti* adj3 inhibitor*) or ((estrogen or oestrogen) adj3 receptor modulator*) or ((anti-resorpti* or anti-osteopor* or bone density) adj3 (drug* or agent* or medicin* or medication* or therap* or treatment*))

#5:

bisphosphonates[tiab] OR “etidronic acid”[tiab] OR “clodronic acid”[tiab] OR “pamidronic acid”[tiab] OR “alendronic acid”[tiab] OR “tiludronic acid”[tiab] OR “ibandronic acid”[tiab] OR “risedronic acid”[tiab] OR “zoledronic acid”[tiab] OR alendronate[tiab] OR risedronate[tiab] OR zoledronate[tiab] OR ibandronate[tiab] OR abaloparatide[tiab] OR teriparatide[tiab] OR denosumab[tiab] OR pamidronate[tiab] OR “strontium ranelate”[tiab] OR “selective estrogen receptor modulators”[tiab] OR SERM[tiab] OR bazedoxifene[tiab] OR raloxifene[tiab] OR ((treatment[ti] OR treated[ti] OR treat*[ti] OR untreated[ti] OR medication[ti] OR medications[ti] OR drug[ti] OR drugs[ti] OR therapy[ti] OR therapeutic[ti] OR "Therapeutics"[Mesh] OR antifracturative[ti]) AND (osteoporosis[ti] OR osteoporotic[ti] OR osteop*[ti]))

#6: #4 OR #5

#7: #3 AND #6

#8: #7 AND Filters: Humans, from 2015/1/1 - 2020/10/20

EMBASE

Up to 20 October 2020

#1:

'wrist fracture'/exp OR 'hip fracture'/exp OR 'spine fracture'/exp OR 'shoulder fracture'/exp OR 'fragility fracture'/exp OR 'radius fracture'/exp OR ((wrist* OR colle* OR radius OR 'articulatio radiocarpea' OR carpus OR carpal OR radiocarp* OR radial OR forearm* OR humerus OR metacarp* OR barton OR monteggi* OR ulna OR ulnar OR 'upper limb' OR 'upper limbs' OR hip OR hips OR trochanteric OR intertrochanteric OR subtrochanteric OR 'femoral neck' OR 'femur neck' OR spine OR spinal OR vertebra* OR lumbar OR shoulder* OR glenohumeral OR humeroscapular OR 'scapulo humeral' OR 'proximal humeral') NEAR/3 fractur*):ab,ti OR ('fracture'/exp AND ('wrist'/exp OR 'hip'/exp OR 'spine'/exp OR 'shoulder'/exp OR 'wrist injury'/de OR 'shoulder injury'/exp OR 'hip injury'/exp OR 'spine injury'/exp)) AND ('osteoporosis'/exp OR osteoporo*:ab,ti OR 'bone loss':ab,ti) OR

#2: 'fragility fracture'/exp

#3: 'low energy fracture'/exp

#4: 'low trauma fracture'/exp

#5: 'low energy trauma'/exp

#6:

“fragility fracture”:ti OR “fragility fractures”:ti OR “low energy fracture”:ti OR “low energy fractures”:ti OR “low-energy fracture”:ti OR “low-energy fractures”:ti OR “low trauma fracture”:ti OR “low trauma fractures”:ti OR “low-trauma fracture”:ti OR “low-trauma fractures”:ti OR “low energy trauma”:ti OR “low-energy trauma”:ti OR “low level trauma”:ti OR “low-level trauma”:ti OR “minor trauma fracture”:ti OR “minor trauma fractures”:ti OR “minor-trauma fracture”:ti OR “minor-trauma fractures”:ti OR “minor fracture”:ti OR “minor fractures”:ti OR “minor-fracture”:ti OR “minor-fractures”:ti OR “osteoporotic fracture”:ti OR “osteoporotic fractures”:ti

#7: #1 OR #2 OR #3 OR #4 OR #5 OR #6

#8:

'bone density conservation agent'/exp OR 'osteoporosis'/exp/dm_dt OR 'bisphosphonic acid derivative'/exp OR 'calcitonin'/exp OR 'selective estrogen receptor modulator'/exp OR 'raloxifene'/exp OR 'denosumab'/exp OR 'parathyroid hormone[1-34]'/exp OR ('osteoclast differentiation factor'/exp AND 'monoclonal antibody'/exp) OR abaloparatide:ab,ti OR (strontium ranelate):ab,ti OR bazedoxifene:ab,ti OR aclasta:ab,ti OR actonel:ab,ti OR alend:ab,ti OR alendro*:ab,ti OR alovell:ab,ti OR amgiva:ab,ti OR aminodron*:ab,ti OR aminobutane*:ab,ti OR aminohexane*:ab,ti OR aminohydroxy*:ab,ti OR aminomux:ab,ti OR aminopropane*:ab,ti OR

aminopropylidene*:ab,ti OR aredia:ab,ti OR aredronet:ab,ti OR arendal:ab,ti OR atelvia:ab,ti OR belfosdil:ab,ti OR benet:ab,ti OR bifemelan:ab,ti OR bifosa:ab,ti OR binosto:ab,ti OR bisphonal:ab,ti OR bisphosphon*:ab,ti OR bonapex:ab,ti OR bondenza:ab,ti OR bondronat*:ab,ti OR bonefos:ab,ti OR boniva:ab,ti OR bonmax:ab,ti OR bonviva:ab,ti OR butedron*:ab,ti OR calcitar:ab,ti OR calciton*:ab,ti OR calcitrin:ab,ti OR cangrelor:ab,ti OR celvista:ab,ti OR cibalcin:ab,ti OR cimadron*:ab,ti OR clodron*:ab,ti OR coldron*:ab,ti OR cycloheptylaminomethylenebis:ab,ti OR defixal:ab,ti OR denosumab:ab,ti OR dequest:ab,ti OR destara:ab,ti OR diadronel:ab,ti OR dichlorometh*:ab,ti OR didronal:ab,ti OR didronat*:ab,ti OR didronel:ab,ti OR difosfonal:ab,ti OR difosfen:ab,ti OR dinol:ab,ti OR diphos:ab,ti OR diphosphon*:ab,ti OR dronal:ab,ti OR dronate:ab,ti OR editron*:ab,ti OR ehdp:ab,ti OR endronax:ab,ti OR ethane*:ab,ti OR ethylenehydroxy*:ab,ti OR ethylidenebisphosphon*:ab,ti OR etibon:ab,ti OR etidron*:ab,ti OR eucalen:ab,ti OR evista:ab,ti OR fixopan:ab,ti OR forsteo:ab,ti OR forteo:ab,ti OR fosalan:ab,ti OR fosamax:ab,ti OR fosmin:ab,ti OR fosval:ab,ti OR hedp:ab,ti OR hexane*:ab,ti OR hydroxyeth*:ab,ti OR hydroxyhex*:ab,ti OR hydroxyl*:ab,ti OR iasibon:ab,ti OR ibandron*:ab,ti OR incadron*:ab,ti OR kengreal:ab,ti OR kengrexal:ab,ti OR keoxifene:ab,ti OR lidadronate:ab,ti OR lodronat*:ab,ti OR loxar:ab,ti OR loxifen:ab,ti OR marvil:ab,ti OR maxibone:ab,ti OR mebonat:ab,ti OR medron*:ab,ti OR medrotec:ab,ti OR methane*:ab,ti OR methanon*:ab,ti OR methylene*:ab,ti OR minodron*:ab,ti OR neobon:ab,ti OR neridron*:ab,ti OR nerixia:ab,ti OR olpadron*:ab,ti OR oncalst:ab,ti OR onclast:ab,ti OR optinate:ab,ti OR optruma:ab,ti OR orazol:ab,ti OR osdron:ab,ti OR osdronat:ab,ti OR oseotenk:ab,ti OR osficar:ab,ti OR oslene:ab,ti OR ossiten:ab,ti OR ostac:ab,ti OR osteof*:ab,ti OR osteopam:ab,ti OR osteopor:ab,ti OR osteosan:ab,ti OR osteotop:ab,ti OR osteovan:ab,ti OR osticalcin:ab,ti OR pamidronate:ab,ti OR pamisol:ab,ti OR panolin:ab,ti OR parathar:ab,ti OR parathormone*:ab,ti OR 'parathyroid hormone':ab,ti OR 'parathyroid hormones':ab,ti OR porosal:ab,ti OR prolia:ab,ti OR propane*:ab,ti OR propylidenediphosphon*:ab,ti OR raloxifene:ab,ti OR raxeto:ab,ti OR reclast:ab,ti OR ribastamin:ab,ti OR risedron*:ab,ti OR serm:ab,ti OR serms:ab,ti OR skelid:ab,ti OR staporos:ab,ti OR teiroc:ab,ti OR teriparatide:ab,ti OR thyreocalciton*:ab,ti OR thyrocalciton* OR tiludron*:ab,ti OR tibolene:ab,ti OR turpinal:ab,ti OR voroste:ab,ti OR xgeva:ab,ti OR xidifon:ab,ti OR xidiphone:ab,ti OR xydiphon*:ab,ti OR zoledron*:ab,ti OR zomera:ab,ti OR zometa:ab,ti OR (bone NEAR/3 resorpti* NEAR/3 inhibitor*):ab,ti OR ((estrogen OR oestrogen) NEAR/3 receptor* NEAR/3 modulator*):ab,ti OR (('anti-resorption' OR 'anti-osteoporosis' OR 'anti-osteoporotic' OR 'bone density' OR osteopor* OR decalcificat*) NEAR/3 (drug* OR agent* OR medicin* OR medication* OR therap* OR treatment*)):ab,ti

#9: #7 AND #8

#10:
#9 NOT (cancer*:ti OR tumor*:ti OR tumour*:ti OR malignan*:ti OR neoplas*:ti OR carcinoma*:ti) NOT [medline]/lim NOT ([animals]/lim NOT [humans]/lim)
#11: #10 AND (2015:py OR 2016:py OR 2017:py OR 2018:py OR 2019:py OR 2020:py OR 2021:py)

COCHRANE
Up to 20 October 2020
#1:
((wrist* or colle* or radius or "articulatio radiocarpea" or carpus or carpal or radiocarp* or radial or forearm* or humerus or metacarp* or barton or monteggi* or ulna or ulnar or "upper limb" or "upper limbs" or hip or hips or trochanteric or intertrochanteric or subtrochanteric or "femoral neck" or "femur neck" or spine or spinal or vertebra* or lumbar or shoulder* or glenohumeral or humeroscapular or "scapulo humeral" or "proximal humeral") near/3 fractur*):ti,ab or [mh "hip fractures"] or [mh "spinal fractures"] or [mh "shoulder fractures"] or [mh "osteoporotic fractures"] or [mh "radius fractures"] or ([mh "bone fractures"] and ([mh "wrist joint"] or [mh spine] or [mh shoulder] or [mh "shoulder joint"] or [mh hip])) and ([mh osteoporosis] or (osteoporo* or "bone loss"):ti,ab)
#2: MeSH descriptor: [Osteoporotic Fractures] explode all trees
#3: MeSH descriptor: [Fractures, Spontaneous] explode all trees
#4:
(fragility fracture):ti OR (fragility fractures):ti OR (low energy fracture):ti OR (low energy fractures):ti OR (low-energy fracture):ti OR (low-energy fractures):ti OR (low trauma fracture):ti OR (low trauma fractures):ti OR (low-trauma fracture):ti OR (low-trauma fractures):ti OR (low energy trauma):ti OR (low-energy trauma):ti OR (low level trauma):ti OR (low-level trauma):ti OR (minor trauma fracture):ti OR (minor trauma fractures):ti OR (minor-trauma fracture):ti OR (minor-trauma fractures):ti OR (minor fracture):ti OR (minor fractures):ti OR (minor-fracture):ti OR (minor-fractures):ti OR (osteoporotic fracture):ti OR (osteoporotic fractures):ti OR (pathologic fracture):ti OR (pathological fractures):ti
#5: #1 OR #2 OR #3 OR #4
#6:
[mh "bone density conservation agents"] or [mh osteoporosis/DT] or [mh diphosphonates] or [mh calcitonin] or [mh "selective estrogen receptor modulators"] or [mh "raloxifene hydrochloride"] or

[mh teriparatide] or ([mh "antibodies, monoclonal"] and [mh "rank ligand"]) or (abaloparatide OR “strontium ranelate” OR bazedoxifene OR aclasta or actonel or alend or alendro* or alovell or amgiva or aminodron* or aminobutane* or aminohexane* or aminohydroxy* or aminomux or aminopropane* or aminopropylidene* or aredia or aredronet or arendal or atelvia or belfosdil or benet or bifemelan or bifosa or binosto or bisphonal or bisphosphon* or bonapex or bondenza or bondronat* or bonefos or boniva or bonmax or bonviva or butedron* or calcitar or calciton* or calcitrin or cangrelor or celvista or cibalcin or cimadron* or clodron* or coldron* or cycloheptylaminomethylenebis or defixal or denosumab or dequest or destara or diadronel or dichlorometh* or didronal or didronat* or didronel or difosfonal or difosfen or dinol or diphos or diphosphon* or dronal or dronate or editron* or ehdp or endronax or ethane* or ethylenehydroxy* or ethylidenebisphosphon* or etibon or etidron* or eucalen or evista or fixopan or forsteo or forteo or fosalan or fosamax or fosmin or fosval or hedp or hexane* or hydroxyeth* or hydroxyhex* or hydroxyl* or iasibon or ibandron* or incadron* or kengreal or kengrexal or keoxifene or lidadronate or lodronat* or loxar or loxifen or marvil or maxibone or mebonat or medron* or medrotec or methane* or methanon* or methylene* or minodron* or neobon or neridron* or nerixia or olpadron* or oncalst or onclast or optinate or optruma or orazol or osdron or osdronat or oseotenk or osficar or oslene or ossiten or ostac or osteof* or osteopam or osteopor or osteosan or osteotop or osteovan or osticalcin or pamidronate or pamisol or panolin or parathar or parathormone* or "parathyroid hormone*" or porosal or prolia or propane* or propylidenediphosphon* or raloxifene or raxeto or reclast or ribastamin or risedron* or serm or serms or skelid or staporos or teiroc or teriparetide or thyreocalictron* or thyrocalciton* or tiludron* or tibolene or turpinal or voroste or xgeva or xidifon or xidiphone or xydiphon* or zoledron* or zomera or zometa):ti,ab or (bone resorpti* near/3 inhibitor*):ti,ab or ((estrogen or oestrogen) near/3 "receptor modulator*"):ab,ti or ((anti-resorpti* or anti-osteopor* or bone density or osteoporosis) near/3 (drug* or agent* or medicin* or medication* or therap* or treatment*)):ti,ab

#7: #5 and #6

#8: #7 with Cochrane Library publication date from Jan 2015 to Oct 2020

Flow chart

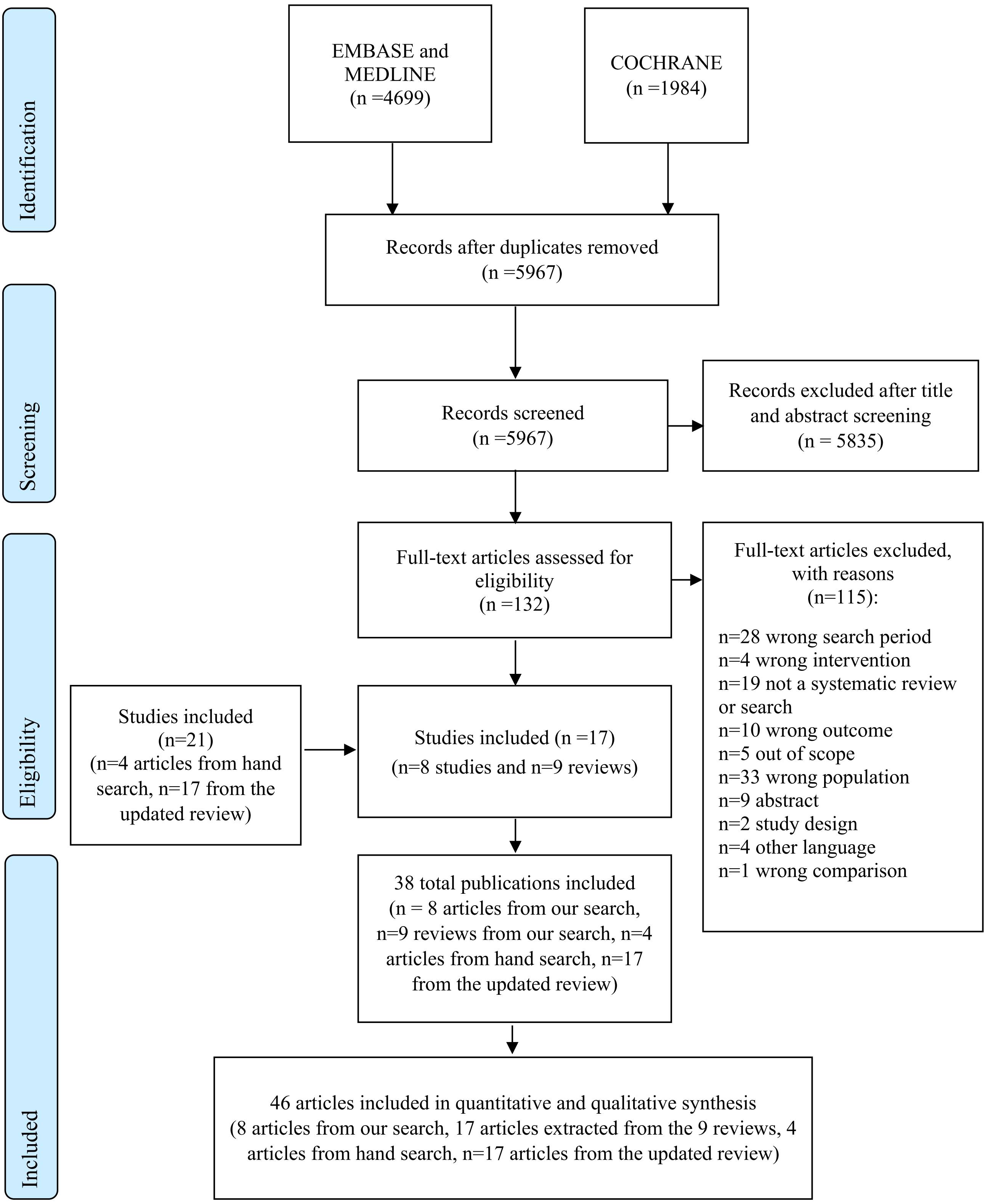
Identification
Screening
Eligibility
Included
EMBASE and MEDLINE (n =4699)
COCHRANE (n =1984)
Records after duplicates removed (n =5967)
Records screened (n =5967)
Records excluded after title and abstract screening (n = 5835)
Full-text articles assessed for eligibility (n =132)
Full-text articles excluded, with reasons (n=115):
n=28 wrong search period
n=4 wrong intervention
n=19 not a systematic review or search
n=10 wrong outcome
n=5 out of scope
n=33 wrong population
n=9 abstract
n=2 study design
n=4 other language
n=1 wrong comparison
Studies included (n=21) (n=4 articles from hand search, n=17 from the updated review)
Studies included (n =17) (n=8 studies and n=9 reviews)
38 total publications included (n = 8 articles from our search, n=9 reviews from our search, n=4 articles from hand search, n=17 from the updated review)
46 articles included in quantitative and qualitative synthesis (8 articles from our search, 17 articles extracted from the 9 reviews, 4 articles from hand search, n=17 articles from the updated review)

Results

Refracture at 12-18 months among fractured, treated or not, patients

Study or Subgroup	Experimental Events	Experimental Total	Control Events	Control Total	Weight	Risk Ratio M-H, Random, 95% CI
**27.1.1 ≥ 1 vertebral fracture**						
Boonen 2011_Vertebral_Denosumab	11	359	19	343	20.3%	0.55 [0.27, 1.15]
Maricic 2002_Vertebral_Raloxifene	3	974	26	2576	9.4%	0.31 [0.09, 1.01]
Meunier 2004_Vertebral_Strontium ranelate	44	686	87	699	43.3%	0.52 [0.36, 0.73]
Nakamura 2006_Vertebral_Raloxifene	0	66	1	37	1.5%	0.19 [0.01, 4.53]
Nakano 2014_Vertebral_Teriparatide	7	261	37	281	18.0%	0.20 [0.09, 0.45]
**Subtotal (95% CI)**		**2346**		**3936**	**92.5%**	**0.41 [0.27, 0.61]**
Total events	65		170			
Heterogeneity: Tau² = 0.06; Chi² = 5.55, df = 4 (P = 0.24); I² = 28%						
Test for overall effect: Z = 4.30 (P < 0.0001)						
**27.1.2 ≥1 non vertebral fracture**						
Cecilia 2008_Hip_Alendronate	2	68	2	79	3.9%	1.16 [0.17, 8.03]
Li 2016_Vertebral_Zoledronate	0	30	4	30	1.8%	0.11 [0.01, 1.98]
Nakamura 2006_Vertebral_Raloxifene	0	66	3	37	1.8%	0.08 [0.00, 1.53]
**Subtotal (95% CI)**		**164**		**146**	**7.5%**	**0.29 [0.05, 1.84]**
Total events	2		9			
Heterogeneity: Tau² = 0.99; Chi² = 3.17, df = 2 (P = 0.21); I² = 37%						
Test for overall effect: Z = 1.31 (P = 0.19)						
**Total (95% CI)**		**2510**		**4082**	**100.0%**	**0.40 [0.27, 0.60]**
Total events	67		179			
Heterogeneity: Tau² = 0.06; Chi² = 8.76, df = 7 (P = 0.27); I² = 20%						
Test for overall effect: Z = 4.51 (P < 0.00001)						
Test for subgroup differences: Chi² = 0.12, df = 1 (P = 0.73), I² = 0%						

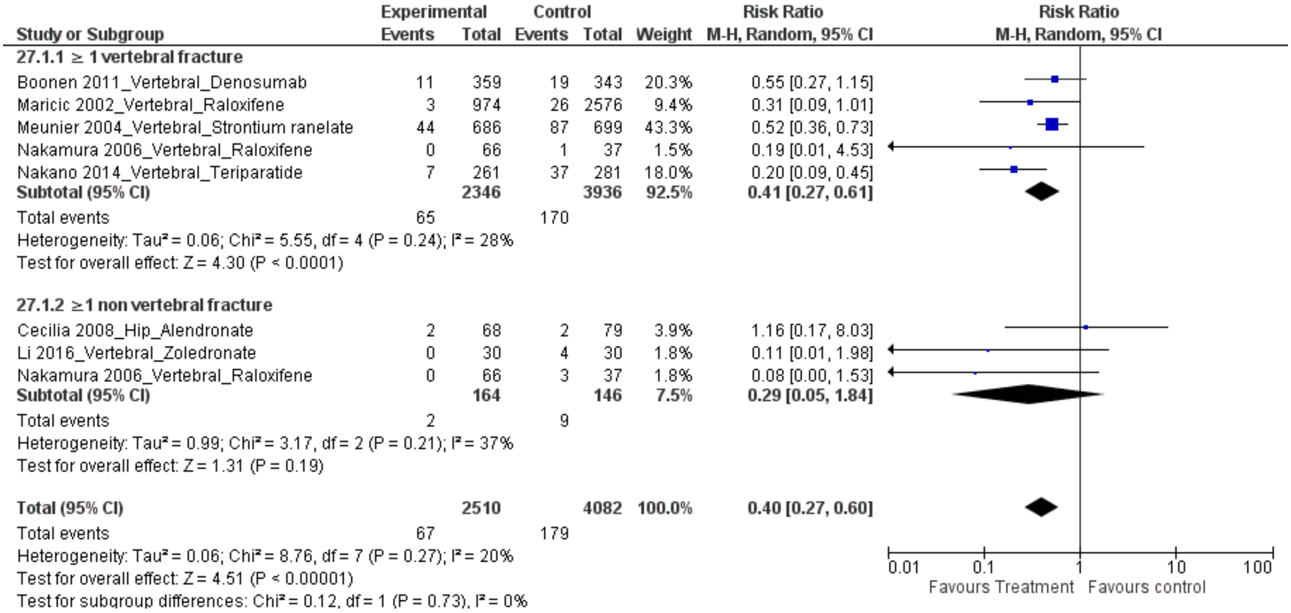

Refracture at 18-24 months among fractured, treated or not, patients

Study or Subgroup	Experimental Events	Experimental Total	Control Events	Control Total	Weight	Risk Ratio M-H, Random, 95% CI
**27.2.1 ≥ 1 vertebral fracture**						
Boonen 2011_Vertebral_Denosumab	16	359	43	343	7.5%	0.36 [0.20, 0.62]
Chesnut 2005_Vertebral_Calcitonin	2	46	2	45	1.6%	0.98 [0.14, 6.65]
Cosman 2016_Non vertebral_Abaloparatide	3	343	22	349	3.3%	0.14 [0.04, 0.46]
Cosman 2016_Vertebral_Abaloparatide	1	147	15	165	1.5%	0.07 [0.01, 0.56]
Fogelman 2000_Any fracture_Risedronate	16	172	17	125	6.7%	0.68 [0.36, 1.30]
Greenspan 2007_Vertebral_PTH	10	236	21	235	6.0%	0.47 [0.23, 0.98]
Kaufman 2005_Vertebral_Teriparatide	6	72	9	42	4.5%	0.39 [0.15, 1.02]
Lyles 2007_Vertebral_Zoledronate	21	1065	39	1062	7.8%	0.54 [0.32, 0.91]
Matsumoto 2009_Vertebral_Minodronate	27	253	62	239	8.8%	0.41 [0.27, 0.62]
Neer 2001_Vertebral_PTH	41	878	64	448	9.2%	0.33 [0.22, 0.48]
Reid 1994_Vertebral_Pamidronate	7	26	10	22	5.6%	0.59 [0.27, 1.29]
**Subtotal (95% CI)**		**3597**		**3075**	**62.5%**	**0.42 [0.33, 0.52]**
Total events	150		304			
Heterogeneity: Tau² = 0.03; Chi² = 13.00, df = 10 (P = 0.22); I² = 23%						
Test for overall effect: Z = 7.44 (P < 0.00001)						
**27.2.3 ≥1 non vertebral fracture**						
Cosman 2016_Non vertebral_Abaloparatide	19	416	11	405	6.0%	1.68 [0.81, 3.49]
Cosman 2016_Vertebral_Abaloparatide	10	188	4	177	3.6%	2.35 [0.75, 7.37]
Fogelman 2000_Any fracture_Risedronate	11	172	13	125	5.7%	0.61 [0.28, 1.33]
Lyles 2007_Vertebral_Zoledronate	79	1065	107	1062	10.1%	0.74 [0.56, 0.97]
Neer 2001_Vertebral_PTH	45	1093	39	544	8.8%	0.57 [0.38, 0.87]
Watts 2019_Wrist(Wrist)_Teriparatide	5	158	5	173	3.2%	1.09 [0.32, 3.71]
**Subtotal (95% CI)**		**3092**		**2486**	**37.5%**	**0.87 [0.60, 1.26]**
Total events	169		179			
Heterogeneity: Tau² = 0.10; Chi² = 10.76, df = 5 (P = 0.06); I² = 54%						
Test for overall effect: Z = 0.75 (P = 0.45)						
**Total (95% CI)**		**6689**		**5561**	**100.0%**	**0.55 [0.42, 0.71]**
Total events	319		483			
Heterogeneity: Tau² = 0.15; Chi² = 42.87, df = 16 (P = 0.0003); I² = 63%						
Test for overall effect: Z = 4.50 (P < 0.00001)						
Test for subgroup differences: Chi² = 10.77, df = 1 (P = 0.001), I² = 90.7%						

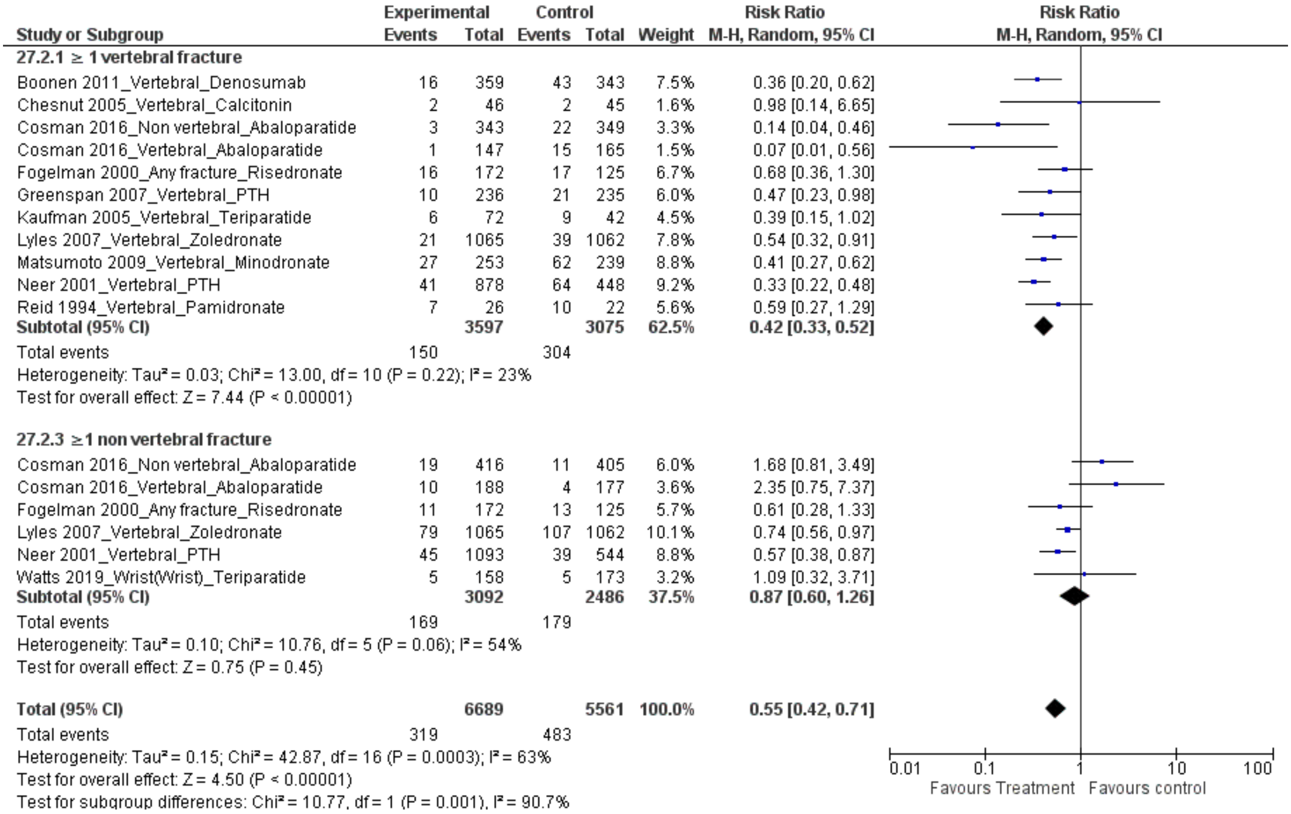

Among treated vs no treated, here the number of subjects affected by non vertebral refracture: Neer 2001 (foot 5 vs 4; hip 5 vs 4; humerus 7 vs 5; pelvic 1 vs 3; rib 10 vs 10; wrist 17 vs 13).

Refracture at 3 years among fractured, treated or not, patients

Study or Subgroup	Experimental Events	Experimental Total	Control Events	Control Total	Weight	Risk Ratio M-H, Random, 95% CI
**27.5.1 ≥1 vertebral fracture**						
Boonen 2011_Vertebral_Denosumab	27	359	57	343	3.2%	0.45 [0.29, 0.70]
Brumsen 2002_Vertebral_Pamidronate	5	46	15	45	1.0%	0.33 [0.13, 0.82]
Chesnut 2000_Vertebral_Calcitonin	140	614	60	203	5.1%	0.77 [0.60, 1.00]
Chesnut 2004_Vertebral_Ibandronate	37	977	73	975	3.6%	0.51 [0.34, 0.74]
Clemmesen 1997_Vertebral_Risedronate	13	29	30	31	3.4%	0.46 [0.31, 0.70]
Ettinger 1999_Vertebral_Raloxifene	195	1534	163	770	6.0%	0.60 [0.50, 0.73]
Frankel 2013_Vertebral_Alendronate	78	984	145	966	5.1%	0.53 [0.41, 0.69]
Harris 1999_Vertebral_Risedronate	61	696	93	678	4.5%	0.64 [0.47, 0.87]
Kendler 2019_Osteoporotic_Denosumab	7	272	39	438	1.4%	0.29 [0.13, 0.64]
Kendler 2019_Vertebral_Denosumab	3	103	17	145	0.7%	0.25 [0.07, 0.83]
Kushida 2004_Vertebral_Alendronate	1	90	6	80	0.2%	0.15 [0.02, 1.20]
Liberman 1995_Vertebral_Alendronate	13	97	13	68	1.6%	0.70 [0.35, 1.42]
McCloskey 2004_Vertebral (≥2 vertebral)_Clodronate	29	129	48	125	3.6%	0.59 [0.40, 0.86]
Meunier 2004_Vertebral_Strontium ranelate	115	719	195	723	5.8%	0.59 [0.48, 0.73]
Reginster 2000_Vertebral_Risedronate	53	344	89	346	4.5%	0.60 [0.44, 0.81]
Reginster 2005_Vertebral_Strontium Ranelate	133	587	201	637	6.1%	0.72 [0.59, 0.87]
Silverman 2008_Vertebral_Bazedoxifene	51	1909	47	981	3.6%	0.56 [0.38, 0.82]
Silverman 2008_Vertebral_Raloxifene	26	954	47	981	2.9%	0.57 [0.36, 0.91]
Sugimoto 2018_Vertebral_Elcatonin	33	166	35	194	3.3%	1.10 [0.72, 1.69]
**Subtotal (95% CI)**		**10609**		**8729**	**65.8%**	**0.60 [0.54, 0.66]**
Total events	1020		1373			
Heterogeneity: Tau² = 0.02; Chi² = 28.99, df = 18 (P = 0.05); I² = 38%						
Test for overall effect: Z = 9.61 (P < 0.00001)						
**27.5.3 ≥1 non vertebral fracture**						
Black 1996_Vertebral_Alendronate	122	981	148	965	5.6%	0.81 [0.65, 1.01]
Brumsen 2002_Vertebral_Pamidronate	1	46	3	45	0.2%	0.33 [0.04, 3.02]
Chesnut 2004_Vertebral_Ibandronate	89	977	80	975	4.7%	1.11 [0.83, 1.48]
Clemmesen 1997_Vertebral_Risedronate	4	29	4	31	0.6%	1.07 [0.29, 3.88]
Delmas 2003_Vertebral_Raloxifene	47	418	31	196	3.3%	0.71 [0.47, 1.08]
Harris 1999_Vertebral_Risedronate	33	812	52	815	3.3%	0.64 [0.42, 0.97]
Kendler 2019_Osteoporotic_Denosumab	14	272	17	438	1.7%	1.33 [0.66, 2.65]
Kendler 2019_Vertebral_Denosumab	4	103	4	145	0.5%	1.41 [0.36, 5.50]
McClung 2001_Vertebral_Risedronate	116	1128	93	575	5.2%	0.64 [0.49, 0.82]
Meunier 2004_Vertebral_Strontium ranelate	112	719	122	723	5.4%	0.92 [0.73, 1.17]
Reginster 2000_Vertebral_Risedronate	36	406	51	406	3.5%	0.71 [0.47, 1.06]
Schemitsch 2020_Vertebral_Romosozumab	6	238	1	87	0.2%	2.19 [0.27, 17.96]
**Subtotal (95% CI)**		**6129**		**5401**	**34.2%**	**0.82 [0.71, 0.95]**
Total events	584		606			
Heterogeneity: Tau² = 0.02; Chi² = 15.54, df = 11 (P = 0.16); I² = 29%						
Test for overall effect: Z = 2.69 (P = 0.007)						
**Total (95% CI)**		**16738**		**14130**	**100.0%**	**0.66 [0.60, 0.73]**
Total events	1604		1979			
Heterogeneity: Tau² = 0.04; Chi² = 63.85, df = 30 (P = 0.0003); I² = 53%						
Test for overall effect: Z = 7.98 (P < 0.00001)						
Test for subgroup differences: Chi² = 12.34, df = 1 (P = 0.0004), I² = 91.9%						

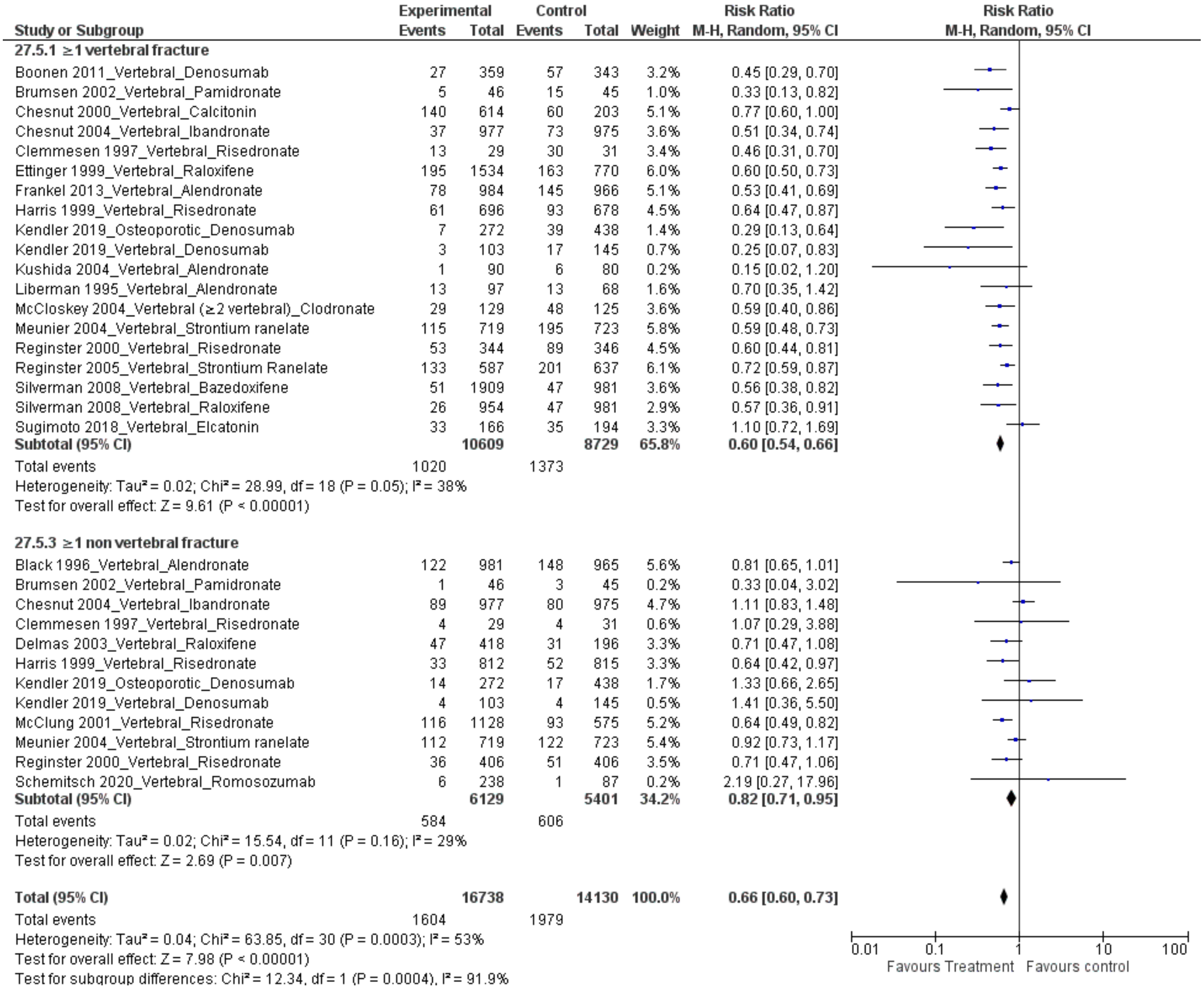

Among treated vs no treated, here the number of subjects affected by non vertebral refracture: with a baseline osteoporotic fracture in Kendler 2019 (foot 0 vs 1; forearm 5 vs 4; hand 0 vs 1; hip 3 vs 1; lower leg 1 vs 2; pelvic 1 vs 1; shoulder 3 vs 4; thorax 1 vs 3);with a baseline vertebral fracture in Schemitsch 2020 (femur 1 vs 0; hip 3 vs 0; pelvic 1 vs 0; tibia 0 vs 1; femoral neck 1 vs 0).

Refracture after 3 years among fractured, treated or not, patients

Study or Subgroup	Experimental Events	Experimental Total	Control Events	Control Total	Weight	Risk Ratio M-H, Random, 95% CI
**27.8.1 ≥1 vertebral fracture**						
Ensrud 2008_Vertebral_Raloxifene	4	5044	4	5057	1.0%	1.00 [0.25, 4.01]
Kanis 2005_Vertebral_Risedronate	106	910	171	892	23.1%	0.61 [0.49, 0.76]
Meunier 2009_Vertebral_Strontium ranelate	129	719	194	726	26.6%	0.67 [0.55, 0.82]
Palacios 2015_Vertebral_Bazedoxifene	63	1011	53	519	12.6%	0.61 [0.43, 0.87]
Sontag 2010_Vertebral_Raloxifene	140	975	204	936	26.8%	0.66 [0.54, 0.80]
**Subtotal (95% CI)**		**8659**		**8130**	**90.2%**	**0.65 [0.58, 0.72]**
Total events	442		626			
Heterogeneity: Tau² = 0.00; Chi² = 0.96, df = 4 (P = 0.92); I² = 0%						
Test for overall effect: Z = 7.67 (P < 0.00001)						
**27.8.2 ≥ non vertebral fracture**						
Ensrud 2008_Vertebral_Raloxifene	48	5044	43	5057	9.8%	1.12 [0.74, 1.69]
**Subtotal (95% CI)**		**5044**		**5057**	**9.8%**	**1.12 [0.74, 1.69]**
Total events	48		43			
Heterogeneity: Not applicable						
Test for overall effect: Z = 0.54 (P = 0.59)						
**Total (95% CI)**		**13703**		**13187**	**100.0%**	**0.68 [0.59, 0.78]**
Total events	490		669			
Heterogeneity: Tau² = 0.01; Chi² = 7.39, df = 5 (P = 0.19); I² = 32%						
Test for overall effect: Z = 5.31 (P < 0.00001)						
Test for subgroup differences: Chi² = 6.42, df = 1 (P = 0.01), I² = 84.4%						

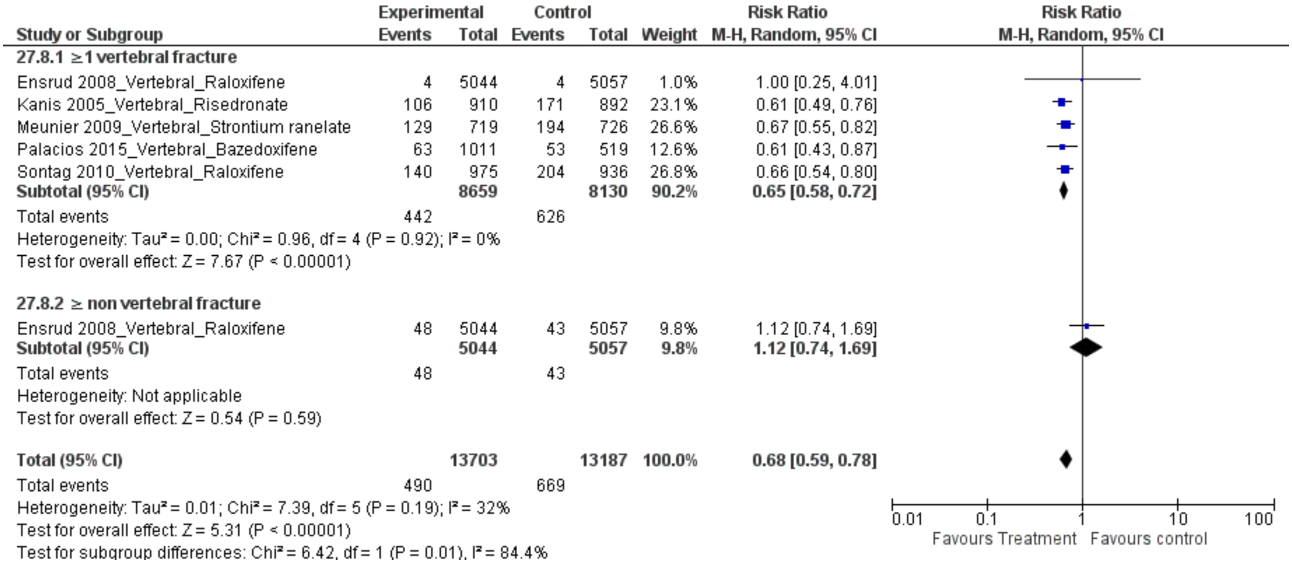

Quality evaluation

Randomized Controlled Trials evaluated with the Risk of Bias tool

	Random sequence generation (selection bias)	Allocation concealment (selection bias)	Blinding of participants and personnel (performance bias)	Blinding of outcome assessment (detection bias)	Incomplete outcome data (attrition bias)	Selective reporting (reporting bias)	Other bias
Active Trial	+	+	+	+	+	?	+
Beaupre 2011	?	?	?	+	?	+	?
Brumsen 2002	?	?	+	+	+	+	+
Cecilia 2008	?	?	−	+	+	+	+
Chesnut 2000	+	+	+	+	+	+	+
Chesnut 2004	?	?	+	+	+	+	−
Clemmesen 1997	?	?	+	+	+	+	?
Fogelman 2000	?	?	+	+	+	+	+
Fracture Intervention Trial	?	+	+	+	+	+	+
Fracture Prevention Trial	?	?	+	+	−	?	−
Freedom Trial	?	?	+	+	+	+	−
Greenspan 2007	+	+	+	+	+	+	+
Horizon Trial	+	+	+	+	+	+	?
Kaufman 2005	+	+	+	+	−	+	+

Kushida 2004	?	?	+	+	?	+	?
Li 2016	?	?	?	+	?	+	?
Liberman 1995	?	?	+	+	?	+	+
Matsumoto 2009	+	+	+	+	+	+	+
McCloskey 2004	?	?	+	+	+	+	+
McClung 2001	?	?	?	+	+	+	+
Meunier 2004	?	?	+	+	+	+	−
Meunier 2009	+	+	+	+	+	+	−
More Trial	?	?	+	+	+	+	−
Nakamura 2006	+	+	+	+	?	+	?
Palacios 2015	?	?	+	+	+	+	?
Quest Trial	?	?	+	+	+	+	+
Reid 1994	?	?	+	+	+	+	+
Ruth Trial	+	+	+	+	+	+	−
Schemitsch 2020	+	+	+	+	+	+	+
Silverman 2008	+	+	+	+	+	+	+
Sugimoto 2018	?	?	+	+	+	+	+
Tower Trial	?	?	+	+	?	+	+
Tropos Trial	?	?	+	+	?	+	+
Vert Trial	+	+	+	+	+	+	+

Summary of Findings

Certainty assessment							№ of patients		Effect		Certainty	Importance
**№ of studies**	**Study design**	**Risk of bias**	**Inconsistency**	**Indirectness**	**Imprecision**	**Other considerations**	**Treated subjects**	**placebo**	**Relative (95% CI)**	**Absolute (95% CI)**		
**Refracture at 1-1.5 years**												
7	randomised trials	serious [a]	not serious	not serious	not serious	none	67/2510 (2.7%)	179/4082 (4.4%)	**RR 0.40** (0.27 to 0.60)	**26 fewer per 1.000** (from 32 fewer to 18 fewer)	⊕⊕⊕◯ MODERATE	CRITICAL
**Refracture at 1.5-2 years**												
12	randomised trials	serious [b]	not serious	not serious	not serious	none	319/6689 (4.8%)	483/5561 (8.7%)	**RR 0.55** (0.42 to 0.71)	**39 fewer per 1.000** (from 50 fewer to 25 fewer)	⊕⊕⊕◯ MODERATE	CRITICAL
**Refracture at 3 years**												
23	randomised trials	serious [c]	not serious	not serious	not serious	none	1604/16738 (9.6%)	1979/14130 (14.0%)	**RR 0.66** (0.60 to 0.73)	**48 fewer per 1.000** (from 56 fewer to 38 fewer)	⊕⊕⊕◯ MODERATE	CRITICAL
**Refracture more than 3 years**												
5	randomised trials	serious [d]	not serious	not serious	not serious	none	490/13703 (3.6%)	669/13187 (5.1%)	**RR 0.68** (0.59 to 0.78)	**16 fewer per 1.000** (from 21 fewer to 11 fewer)	⊕⊕⊕◯ MODERATE	CRITICAL

CI: Confidence interval; **RR:** Risk ratio

Explanations

a. UNCLEAR Risk of bias for the random sequence generation and the allocation concealment: Boonen 2011, MORE Trial, Meunier 2004, Tower Trial, Cecilia 2008, Li 2016. UNCLEAR (Li 2016) and HIGH (Cecilia 2008) Risk of bias for Blinding of participants and personnel. UNCLEAR Risk of bias for incomplete outcome data: Nakamura 2006, Tower Trial, Li 2016. HIGH (Boonen 2011, MORE Trial, Meunier 2004) and UNCLEAR (Nakamura 2006, Li 2016) Risk of bias for other bias.

b. UNCLEAR Risk of bias for the random sequence generation and the allocation concealment: Boonen 2011, Chesnut 2005, Fogelman 2000, Fracture Prevention Trial, Reid 1994. UNCLEAR Risk of bias for selective reporting outcome: Active Trial, Fracture Prevention Trial. HIGH Risk of bias for incomplete outcome data: Kaufman 2005, Fracture Prevention Trial. HIGH (Boonen 2011, Fracture Prevention Trial) and UNCLEAR (Horizon Trial) for other bias

c. UNCLEAR Risk of bias for the random sequence generation: Fracture Intervention Trial. UNCLEAR Risk of bias for the random sequence generation and the allocation concealment: Boonen 2011, Brumsen 2002, Chesnut 2004, Clemmesen 1997, MORE Trial, Freedom Trial, Liberman 1995, McCloskey 2004, Kushida 2004, Meunier 2004, Tropos Trial, Sugimoto 2018, McClung 2001. UNCLEAR Risk of bias for blinding of participants and personnel: McClung 2001. UNCLEAR Risk of bias for incomplete outcome data: Liberman 1995, Kushida 2004, Tropos Trial. HIGH (Boonen 2011, Chesnut 2004, More Trial, Freedom Trial, Meunier 2004) and UNCLEAR (Clemmesen 1997, Kushida 2004) Risk of bias for other bias.

d. UNCLEAR Risk of bias for the random sequence generation and the allocation concealment: Palacios 2015, More Trial. HIGH (Ruth Trial, Meunier 2009, More Trial) and UNCLEAR (Palacios 2015) Risk of bias for other bias.

CQ 2

What operational characteristics and applicability do the available risk assessment tools and algorithms show?

Search strategy

From the NICE Guideline:

FRAX and QFracture

Search constructed by combining the columns in the following table using the AND Boolean operator

Population	Exposure/Intervention	Study filter used	Date parameters
FRAX or QFracture*	None	None	All years -21/7/11 and a top up on 14/9/11

**Non-standard population used.*

MEDLINE

Up to 7 December 2020

FRAX TOOL

#1: FRAX[tiab] OR FRAXTM[tiab]

#2: risk*[tiab] AND assess*[tiab] AND tool*[tiab]

#3: fracture*[tiab]

#4: #2 AND #3

#5: “fracture risk assessment tool”[tiab]

#6: #1 OR #4 OR #5

#7: Letter/

#8: Editorial/

#9: News/

#10: exp Historical article/

#11: Anecdotes as topic/

#12: Comment/

#13: Case report/

#14: Letter[ti] OR comment*[ti] OR abstracts[ti]

#15: OR/7-14

#16: #6 NOT #15 Filters: Humans, from 2011/9/14

Up to 8 December 2020

DEFRA, FRA-HS TOOL

#1: DEFRA[tiab] OR FRA-HS[tiab] OR FRAHS[tiab]

#2: Letter/

#3: Editorial/

#4: News/

#5: exp Historical article/

#6: Anecdotes as topic/

#7: Comment/

#8: Case report/

#9: Letter[ti] OR comment*[ti] OR abstracts[ti]

#10: or/2-9

EMBASE

Up to 7 December 2020

FRAX TOOL

#1: frax:ti,ab OR fraxtm:ti,ab

#2: risk*:ti,ab AND assess*:ti,ab AND tool*:ti,ab

#3: fracture*:ti,ab

#4: #2 and #3

#5: "fracture risk assessment tool":ti,ab

#6: #1 OR #4 OR #5

#7: letter.pt. OR Letter

#8: note.pt

#9: editorial.pt

#10: (Case report) OR (Case study)

#11: letter:ti OR comment*:ti

#12: #7 OR #8 OR #9 OR #10 OR #11

#13: "Randomized controlled trial":ti,ab OR random*:ti,ab

#14: #12 NOT #13

#15: Animal NOT Human

#16: Nonhuman

#17: exp Animal experiment

#18: exp Experimental animal

#19: Animal model

#20: exp Rodent

#21: Rat:ti OR rats:ti OR mouse:ti OR mice:ti

#22 #14 OR #15 OR#16 OR #17 OR #18 OR #19 OR #20 OR #21

#23: #6 NOT #22

#24:

#23 AND (2011:py OR 2012:py OR 2013:py OR 2014:py OR 2015:py OR 2016:py OR 2017:py OR 2018:py OR 2019:py OR 2020:py OR 2021:py) AND [embase]/lim NOT ([embase]/lim AND [medline]/lim)

Up to 8 December 2020

DEFRA, FRA-HS TOOL

#1: defra:ti,ab OR frahs:ti,ab OR fra-hs:ti,ab

#2: letter.pt. OR Letter

#3: note.pt

#4: editorial.pt

#5: (Case report) OR (Case study)

#6: letter:ti OR comment*:ti

#7: #2 OR #3 OR #4 OR #5 OR #6

#8: "Randomized controlled trial":ti,ab OR random*:ti,ab

#9: #7 NOT #8

#10: Animal NOT Human

#11: Nonhuman

#12: exp Animal experiment

#13: exp Experimental animal

#14: Animal model

#15: exp Rodent

#16: Rat:ti OR rats:ti OR mouse:ti OR mice:ti

#17 #9 OR#10 OR #11 OR#12 OR #13 OR #14 OR #15 OR #16

#18: #1 NOT #17

#19: #18 AND [embase]/lim NOT ([embase]/lim AND [medline]/lim)

COCHRANE

Up to 7 December 2020

FRAX TOOL

#1: (FRAX or FRAXTM):ti,ab,kw

#2: (fracture* risk assess* tool*):ti,ab,kw

#3: (risk* AND assess* AND tool*):ti,ab

#4: fracture*:ti,ab

#5: (#3 AND #4)

#6. (#1 OR #2 OR #5) with Cochrane Library publication date from Sep 2011 to present

Up to 8 December 2020

DEFRA, FRA-HS TOOL

#1: (defra OR frahs OR fra-hs):ti,ab,kw

Flow chart

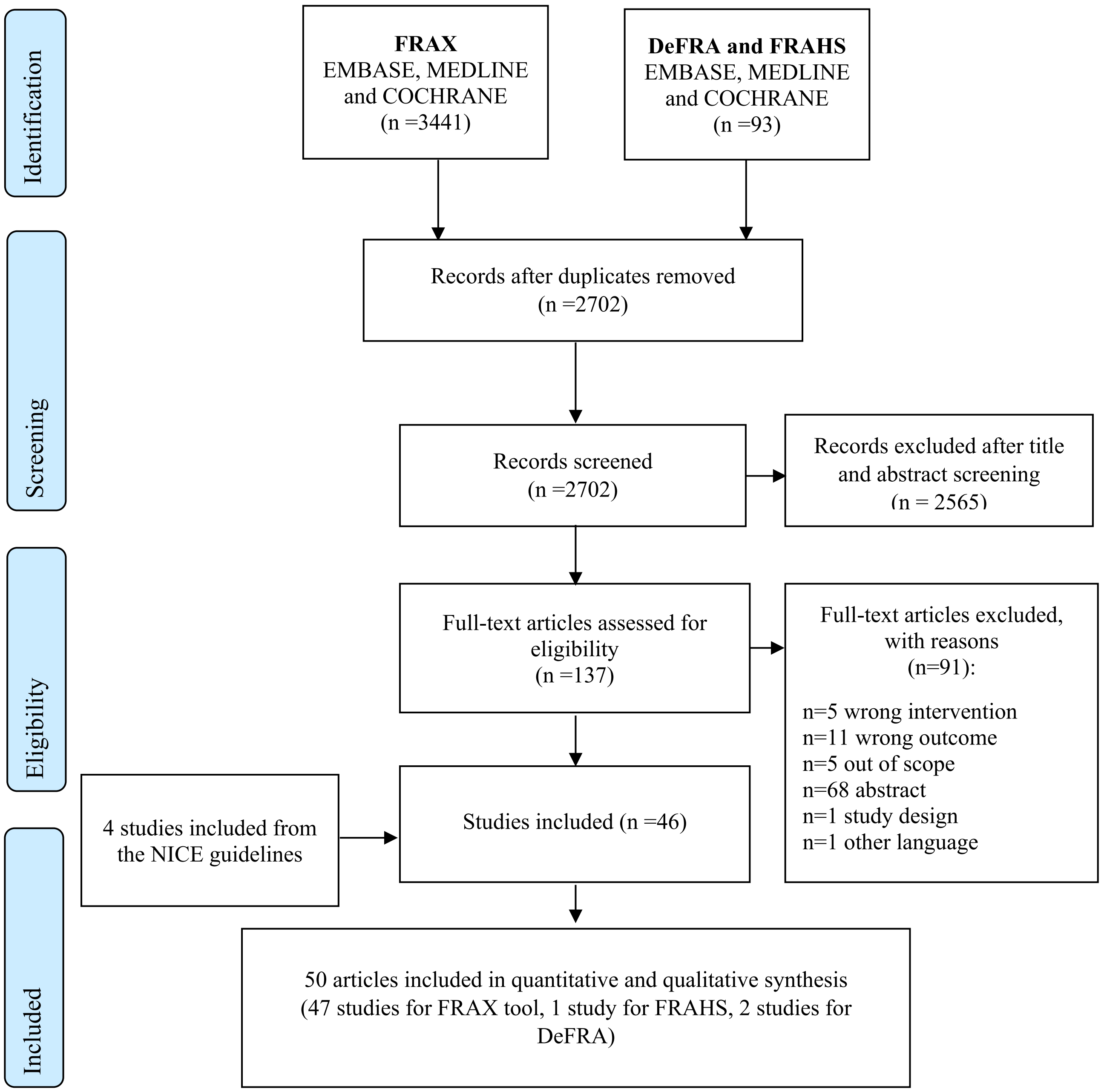
Identification
Screening
Eligibility
Included
FRAX
EMBASE, MEDLINE
and COCHRANE
(n =3441)
DeFRA and FRAHS
EMBASE, MEDLINE
and COCHRANE
(n =93)
Records after duplicates removed
(n =2702)
Records screened
(n =2702)
Records excluded after title
and abstract screening
(n = 2565)
Full-text articles assessed for
eligibility
(n =137)
Full-text articles excluded,
with reasons
(n=91):
n=5 wrong intervention
n=11 wrong outcome
n=5 out of scope
n=68 abstract
n=1 study design
n=1 other language
4 studies included from
the NICE guidelines
Studies included (n =46)
50 articles included in quantitative and qualitative synthesis
(47 studies for FRAX tool, 1 study for FRAHS, 2 studies for
DeFRA)

Results

Sensitivity and Specificity, FRAX

WOMEN

MOF (major osteoporotic fracture) without BMD (bone mineral density) at 3%

Study	TP	FP	FN	TN	Sensitivity (95% CI)	Specificity (95% CI)
El Maghraoui 2014	101	156	76	576	0.57 [0.49, 0.64]	0.79 [0.76, 0.82]
Villa 2016	180	47	31	24	0.85 [0.80, 0.90]	0.34 [0.23, 0.46]

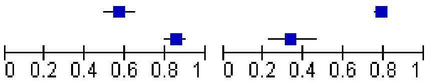

MOF without BMD at 5%

Study	TP	FP	FN	TN	Sensitivity (95% CI)	Specificity (95% CI)
El Maghraoui 2014	61	82	116	649	0.34 [0.27, 0.42]	0.89 [0.86, 0.91]

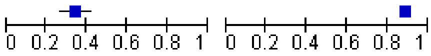

MOF without BMD at 10%

Study	TP	FP	FN	TN	Sensitivity (95% CI)	Specificity (95% CI)
Bolland 2011	147	561	82	632	0.64 [0.58, 0.70]	0.53 [0.50, 0.56]
Ensrud 2009	1037	4998	0	0	1.00 [1.00, 1.00]	0.00 [0.00, 0.00]
van Geel 2014	22	106	26	352	0.46 [0.31, 0.61]	0.77 [0.73, 0.81]

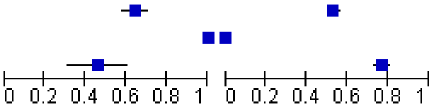

MOF without BMD at 20%

Study	TP	FP	FN	TN	Sensitivity (95% CI)	Specificity (95% CI)
van Geel 2014	4	22	44	436	0.08 [0.02, 0.20]	0.95 [0.93, 0.97]

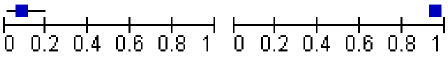

MOF without BMD at 30%

Study	TP	FP	FN	TN	Sensitivity (95% CI)	Specificity (95% CI)
van Geel 2014	2	4	47	453	0.04 [0.00, 0.14]	0.99 [0.98, 1.00]

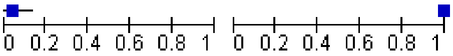

MOF with BMD at 3%

Study	TP	FP	FN	TN	Sensitivity (95% CI)	Specificity (95% CI)
Chandran 2018	6	17	3	50	0.67 [0.30, 0.93]	0.75 [0.63, 0.84]

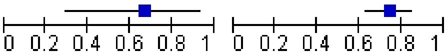

MOF with BMD at 5%

Study	TP	FP	FN	TN	Sensitivity (95% CI)	Specificity (95% CI)
Chandran 2018	96	187	50	457	0.66 [0.57, 0.73]	0.71 [0.67, 0.74]

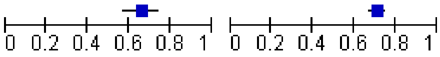

MOF with BMD at 10%

Study	TP	FP	FN	TN	Sensitivity (95% CI)	Specificity (95% CI)
Bolland 2011	96	286	133	907	0.42 [0.35, 0.49]	0.76 [0.73, 0.78]
Cheung 2014	66	573	40	1588	0.62 [0.52, 0.71]	0.73 [0.72, 0.75]
Ensrud 2009	1005	4264	32	734	0.97 [0.96, 0.98]	0.15 [0.14, 0.16]
Kharroubi 2017	77	43	32	229	0.71 [0.61, 0.79]	0.84 [0.79, 0.88]
van Geel 2014	20	72	27	386	0.43 [0.28, 0.58]	0.84 [0.81, 0.87]

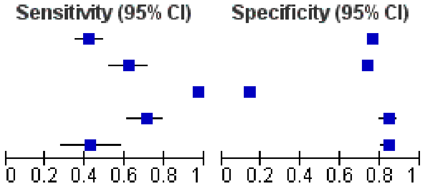

MOF with BMD at 20%

Study	TP	FP	FN	TN	Sensitivity (95% CI)	Specificity (95% CI)
Bolland 2011	21	48	208	1145	0.09 [0.06, 0.14]	0.96 [0.95, 0.97]
Crandall 2019	1257	3523	4936	44742	0.20 [0.19, 0.21]	0.93 [0.92, 0.93]
Ensrud 2009	422	946	615	4052	0.41 [0.38, 0.44]	0.81 [0.80, 0.82]
van Geel 2014	4	13	44	445	0.08 [0.02, 0.20]	0.97 [0.95, 0.98]

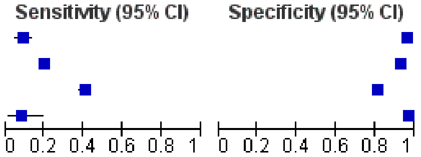

MOF with BMD at 30%

Study	TP	FP	FN	TN	Sensitivity (95% CI)	Specificity (95% CI)
van Geel 2014	0	6	0	500	Not estimable	0.99 [0.97, 1.00]

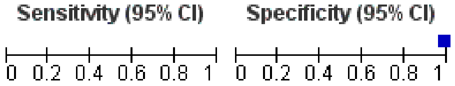

HIP fracture without BMD at 3%

Study	TP	FP	FN	TN	Sensitivity (95% CI)	Specificity (95% CI)
Bolland 2011	172	731	51	468	0.77 [0.71, 0.82]	0.39 [0.36, 0.42]
Hippisley-Cox 2009	4249	123396	2818	748372	0.60 [0.59, 0.61]	0.86 [0.86, 0.86]
Indhavivadhana 2016	1	1	11	1025	0.08 [0.00, 0.38]	1.00 [0.99, 1.00]

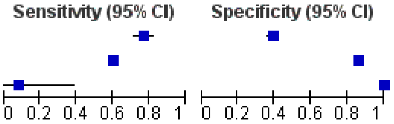

HIP fracture without BMD at 5%

Study	TP	FP	FN	TN	Sensitivity (95% CI)	Specificity (95% CI)
Bolland 2011	117	453	112	740	0.51 [0.44, 0.58]	0.62 [0.59, 0.65]
Ensrud 2009	304	2924	85	2920	0.78 [0.74, 0.82]	0.50 [0.49, 0.51]
Hippisley-Cox 2009	2957	66739	4110	805029	0.42 [0.41, 0.43]	0.92 [0.92, 0.92]

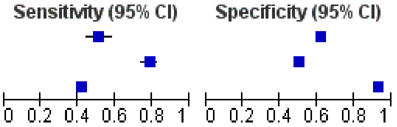

HIP fracture with BMD at 3%

Study	TP	FP	FN	TN	Sensitivity (95% CI)	Specificity (95% CI)
Bolland 2011	105	334	124	859	0.46 [0.39, 0.53]	0.72 [0.69, 0.75]
Chandran 2018	77	84	68	560	0.53 [0.45, 0.61]	0.87 [0.84, 0.89]
Crandall 2019	1193	10981	725	41560	0.62 [0.60, 0.64]	0.79 [0.79, 0.79]
van Geel 2014	20	79	27	380	0.43 [0.28, 0.58]	0.83 [0.79, 0.86]

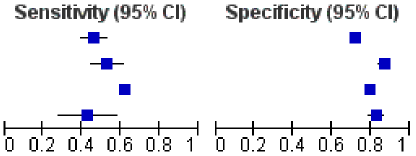

HIP fracture with BMD at 5%

Study	TP	FP	FN	TN	Sensitivity (95% CI)	Specificity (95% CI)
Bolland 2011	66	167	163	1026	0.29 [0.23, 0.35]	0.86 [0.84, 0.88]
Ensrud 2009	297	2184	92	3660	0.76 [0.72, 0.80]	0.63 [0.61, 0.64]
van Geel 2014	15	40	33	418	0.31 [0.19, 0.46]	0.91 [0.88, 0.94]

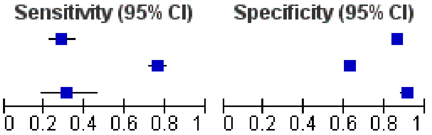

HIP fracture with BMD at 10%

Study	TP	FP	FN	TN	Sensitivity (95% CI)	Specificity (95% CI)
Crandall 2014	105	660	210	4191	0.33 [0.28, 0.39]	0.86 [0.85, 0.87]

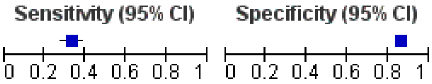

TOTAL POPULATION

MOF without BMD at 3%

Study	TP	FP	FN	TN	Sensitivity (95% CI)	Specificity (95% CI)
El Maghraoui 2014	101	156	76	576	0.57 [0.49, 0.64]	0.79 [0.76, 0.82]
Singh 2020	57	26	53	58	0.52 [0.42, 0.61]	0.69 [0.58, 0.79]
Villa 2016	180	47	31	24	0.85 [0.80, 0.90]	0.34 [0.23, 0.46]

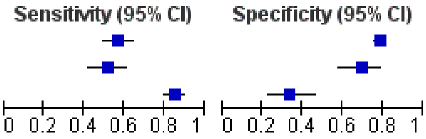

MOF without BMD at 5%

Study	TP	FP	FN	TN	Sensitivity (95% CI)	Specificity (95% CI)
El Maghraoui 2014	61	82	116	649	0.34 [0.27, 0.42]	0.89 [0.86, 0.91]
Singh 2020	38	16	72	68	0.35 [0.26, 0.44]	0.81 [0.71, 0.89]

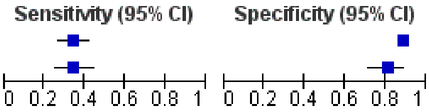

MOF without BMD at 10%

Study	TP	FP	FN	TN	Sensitivity (95% CI)	Specificity (95% CI)
Bolland 2011	147	561	82	632	0.64 [0.58, 0.70]	0.53 [0.50, 0.56]
Ensrud 2009	1037	4998	0	0	1.00 [1.00, 1.00]	0.00 [0.00, 0.00]
Fraser 2011	316	1724	319	4338	0.50 [0.46, 0.54]	0.72 [0.70, 0.73]
Leslie 2010	1653	15348	890	21712	0.65 [0.63, 0.67]	0.59 [0.58, 0.59]
Singh 2020	26	6	84	78	0.24 [0.16, 0.33]	0.93 [0.85, 0.97]
van Geel 2014	22	106	26	352	0.46 [0.31, 0.61]	0.77 [0.73, 0.81]

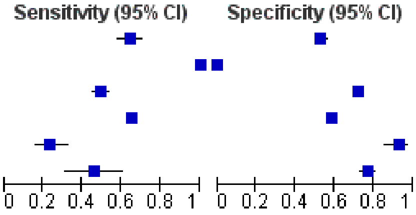

MOF without BMD at 20%

Study	TP	FP	FN	TN	Sensitivity (95% CI)	Specificity (95% CI)
Fraser 2011	102	398	533	5664	0.16 [0.13, 0.19]	0.93 [0.93, 0.94]
Leslie 2010	744	4628	1799	32432	0.29 [0.27, 0.31]	0.88 [0.87, 0.88]
van Geel 2014	4	22	44	436	0.08 [0.02, 0.20]	0.95 [0.93, 0.97]

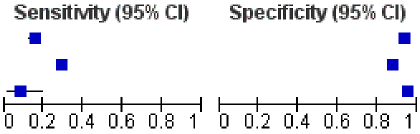

MOF without BMD at 30%

Study	TP	FP	FN	TN	Sensitivity (95% CI)	Specificity (95% CI)
Fraser 2011	28	76	607	5986	0.04 [0.03, 0.06]	0.99 [0.98, 0.99]
Leslie 2010	255	1080	2288	35980	0.10 [0.09, 0.11]	0.97 [0.97, 0.97]
van Geel 2014	2	4	47	453	0.04 [0.00, 0.14]	0.99 [0.98, 1.00]

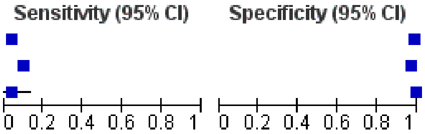

MOF with BMD at 10%

Study	TP	FP	FN	TN	Sensitivity (95% CI)	Specificity (95% CI)
Bolland 2011	96	286	133	907	0.42 [0.35, 0.49]	0.76 [0.73, 0.78]
Cheung 2014	66	573	40	1588	0.62 [0.52, 0.71]	0.73 [0.72, 0.75]
Ensrud 2009	1005	4264	32	734	0.97 [0.96, 0.98]	0.15 [0.14, 0.16]
Fraser 2011	338	1705	297	4357	0.53 [0.49, 0.57]	0.72 [0.71, 0.73]
Kharroubi 2017	77	43	32	229	0.71 [0.61, 0.79]	0.84 [0.79, 0.88]
Leslie 2010	1728	14733	815	22327	0.68 [0.66, 0.70]	0.60 [0.60, 0.61]
van Geel 2014	20	72	27	386	0.43 [0.28, 0.58]	0.84 [0.81, 0.87]

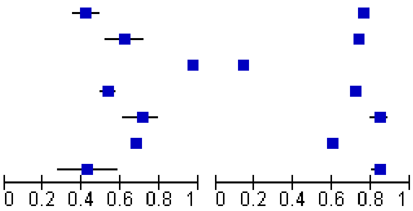

MOF with BMD at 20%

Study	TP	FP	FN	TN	Sensitivity (95% CI)	Specificity (95% CI)
Bolland 2011	21	48	208	1145	0.09 [0.06, 0.14]	0.96 [0.95, 0.97]
Crandall 2019	1257	3523	4936	44742	0.20 [0.19, 0.21]	0.93 [0.92, 0.93]
Ensrud 2009	422	946	615	4052	0.41 [0.38, 0.44]	0.81 [0.80, 0.82]
Fraser 2011	113	382	522	5680	0.18 [0.15, 0.21]	0.94 [0.93, 0.94]
Leslie 2010	707	3512	1836	33548	0.28 [0.26, 0.30]	0.91 [0.90, 0.91]
van Geel 2014	4	13	44	445	0.08 [0.02, 0.20]	0.97 [0.95, 0.98]

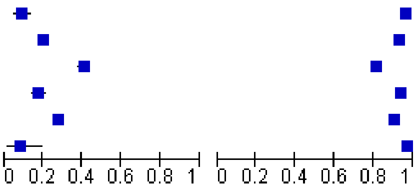

MOF with BMD at 30%

Study	TP	FP	FN	TN	Sensitivity (95% CI)	Specificity (95% CI)
Fraser 2011	37	102	598	5960	0.06 [0.04, 0.08]	0.98 [0.98, 0.99]
Leslie 2010	240	806	2303	36254	0.09 [0.08, 0.11]	0.98 [0.98, 0.98]
van Geel 2014	0	6	0	500	Not estimable	0.99 [0.97, 1.00]

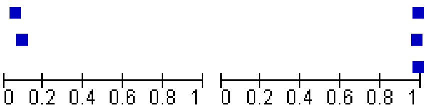

HIP fracture without BMD at 3%

Study	TP	FP	FN	TN	Sensitivity (95% CI)	Specificity (95% CI)
Bolland 2011	172	731	51	468	0.77 [0.71, 0.82]	0.39 [0.36, 0.42]
Fraser 2011	93	1533	64	5007	0.59 [0.51, 0.67]	0.77 [0.76, 0.78]
Hippisley-Cox 2009	4249	123396	2818	748372	0.60 [0.59, 0.61]	0.86 [0.86, 0.86]
Indhavivadhana 2016	1	1	11	1025	0.08 [0.00, 0.38]	1.00 [0.99, 1.00]
Leslie 2010	426	12940	123	23114	0.78 [0.74, 0.81]	0.64 [0.64, 0.65]
Singh 2020	29	8	81	76	0.26 [0.18, 0.36]	0.90 [0.82, 0.96]

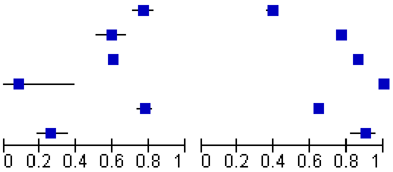

HIP fracture without BMD at 5%

Study	TP	FP	FN	TN	Sensitivity (95% CI)	Specificity (95% CI)
Bolland 2011	117	453	112	740	0.51 [0.44, 0.58]	0.62 [0.59, 0.65]
Ensrud 2009	304	2924	85	2920	0.78 [0.74, 0.82]	0.50 [0.49, 0.51]
Fraser 2011	61	828	96	5712	0.39 [0.31, 0.47]	0.87 [0.87, 0.88]
Hippisley-Cox 2009	2957	66739	4110	805029	0.42 [0.41, 0.43]	0.92 [0.92, 0.92]
Leslie 2010	359	8940	190	30114	0.65 [0.61, 0.69]	0.77 [0.77, 0.78]
Singh 2020	23	3	83	85	0.22 [0.14, 0.31]	0.97 [0.90, 0.99]

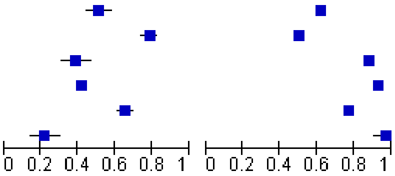

HIP fracture with BMD at 3%

Study	TP	FP	FN	TN	Sensitivity (95% CI)	Specificity (95% CI)
Bolland 2011	105	334	124	859	0.46 [0.39, 0.53]	0.72 [0.69, 0.75]
Chandran 2018	77	84	68	560	0.53 [0.45, 0.61]	0.87 [0.84, 0.89]
Crandall 2019	1193	10981	725	41560	0.62 [0.60, 0.64]	0.79 [0.79, 0.79]
Fraser 2011	99	1316	58	5224	0.63 [0.55, 0.71]	0.80 [0.79, 0.81]
Leslie 2010	425	10818	124	28236	0.77 [0.74, 0.81]	0.72 [0.72, 0.73]
van Geel 2014	20	79	27	380	0.43 [0.28, 0.58]	0.83 [0.79, 0.86]

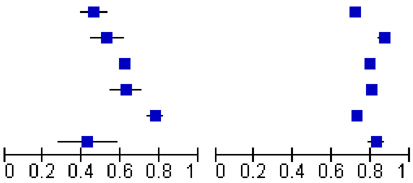

HIP fracture with BMD at 5%

Study	TP	FP	FN	TN	Sensitivity (95% CI)	Specificity (95% CI)
Bolland 2011	66	167	163	1026	0.29 [0.23, 0.35]	0.86 [0.84, 0.88]
Ensrud 2009	297	2184	92	3660	0.76 [0.72, 0.80]	0.63 [0.61, 0.64]
Fraser 2011	68	732	89	5808	0.43 [0.35, 0.51]	0.89 [0.88, 0.90]
Leslie 2010	362	6654	187	32400	0.66 [0.62, 0.70]	0.83 [0.83, 0.83]
van Geel 2014	15	40	33	418	0.31 [0.19, 0.46]	0.91 [0.88, 0.94]

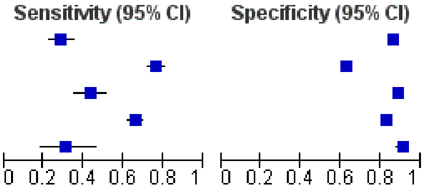

Area under the curve
FRAX Tool

WOMEN

MOF with BMD

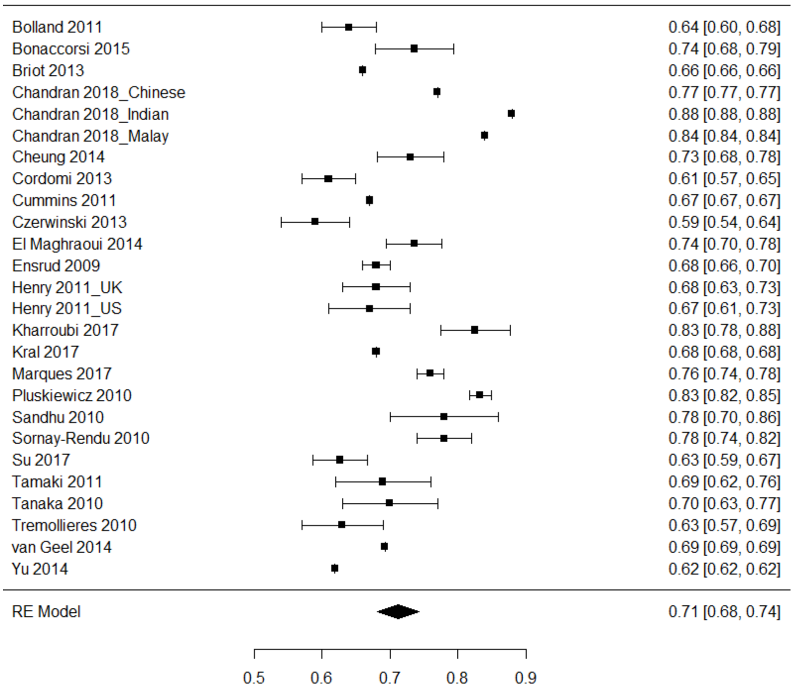

MOF without BMD

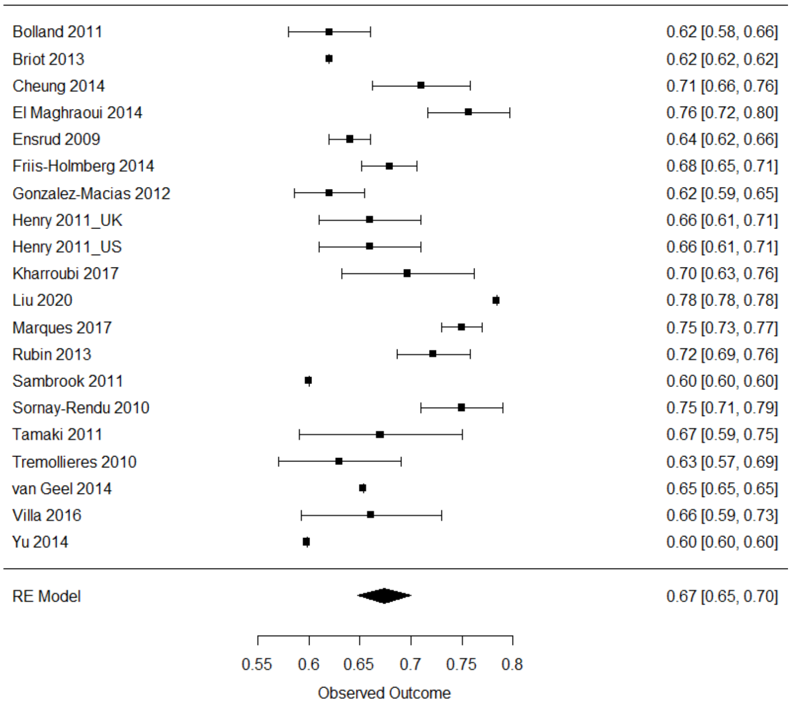

HIP fracture with BMD

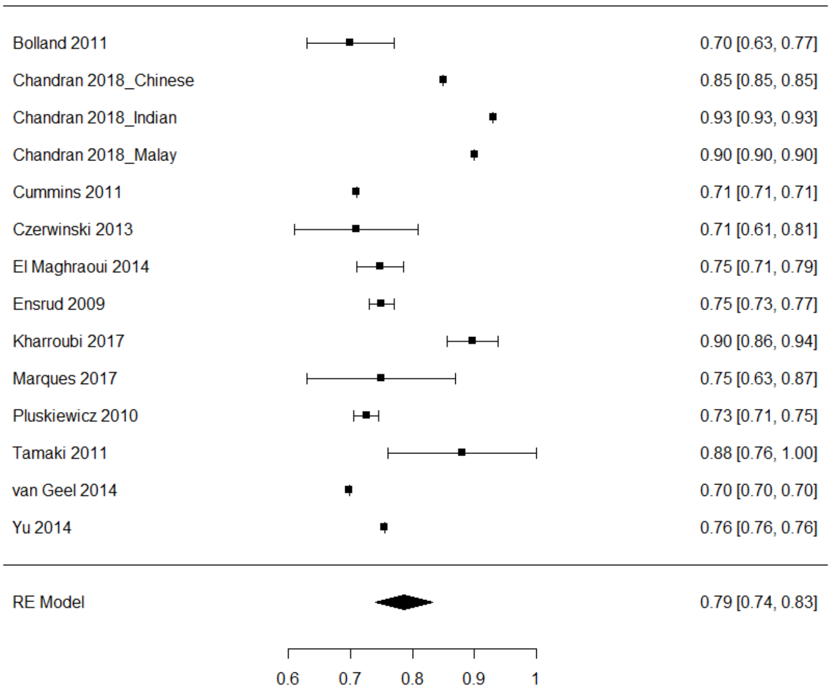

HIP fracture without BMD

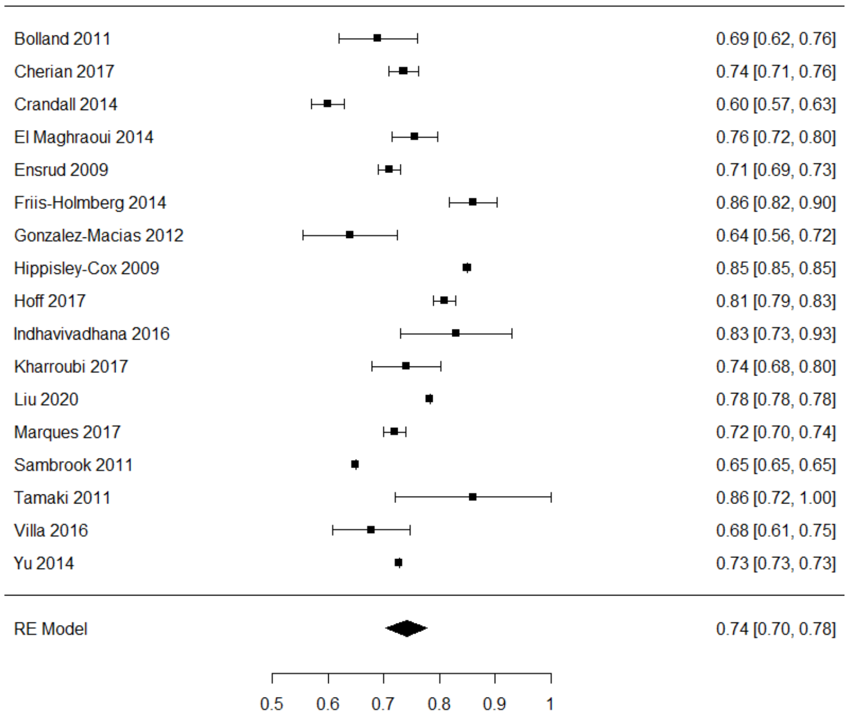

MEN

MOF with BMD

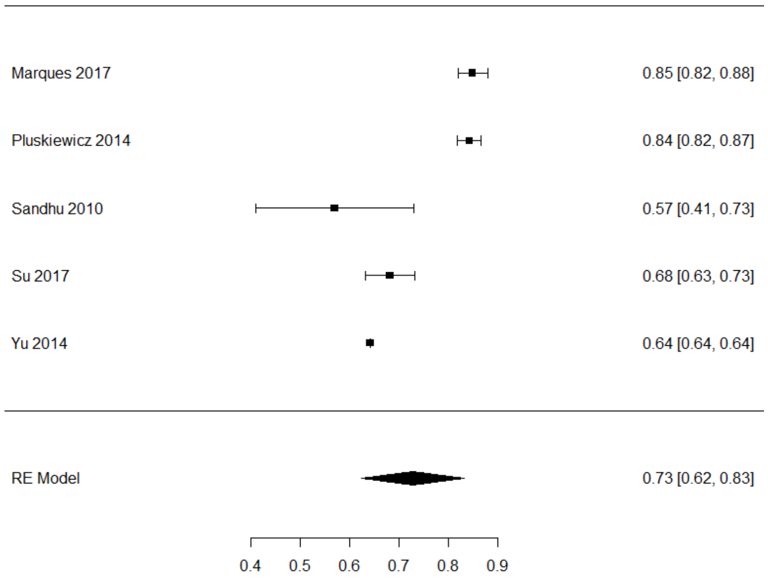

MOF without BMD

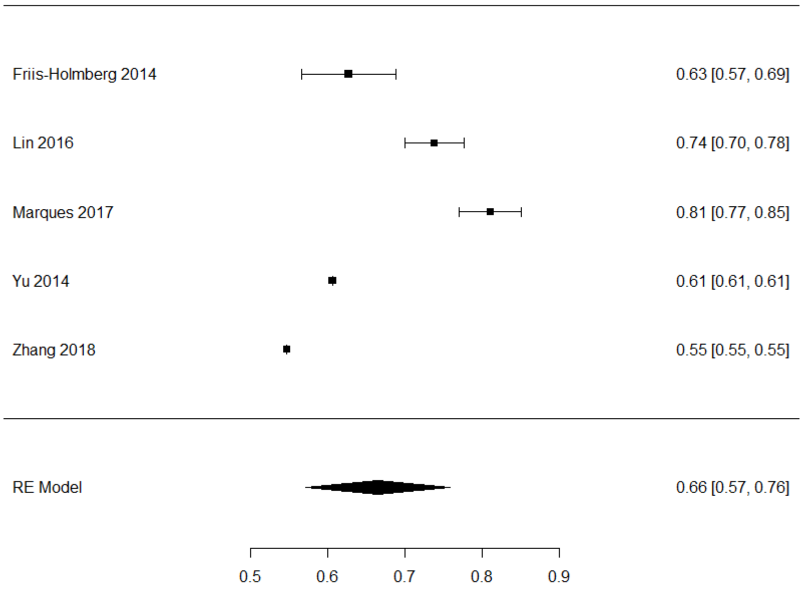

HIP fracture with BMD

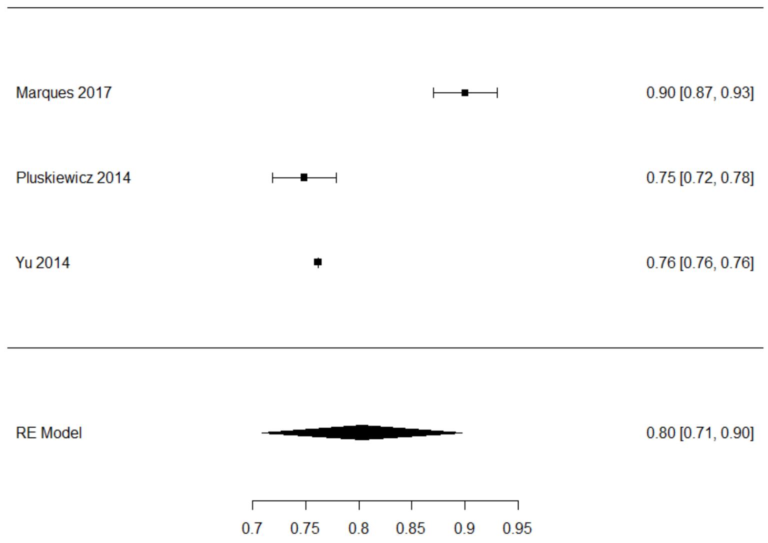

HIP fracture without BMD

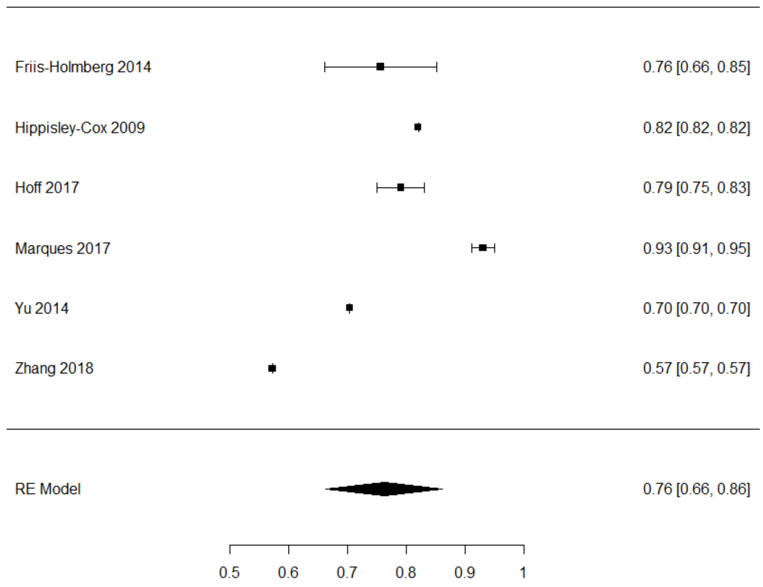

TOTAL POPULATION

MOF with BMD

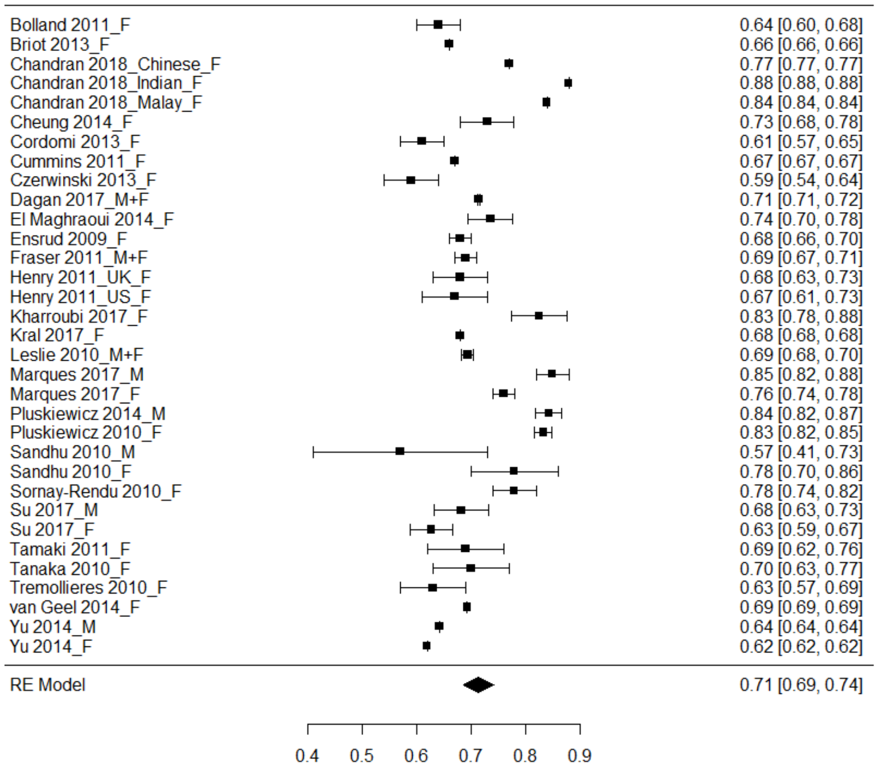

MOF without BMD

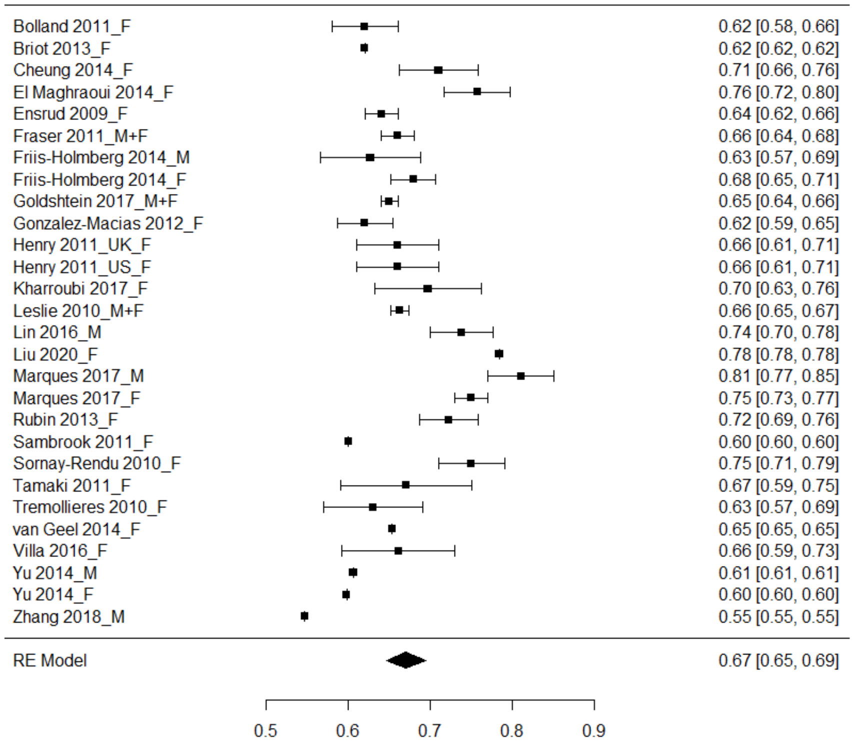

HIP fracture with BMD

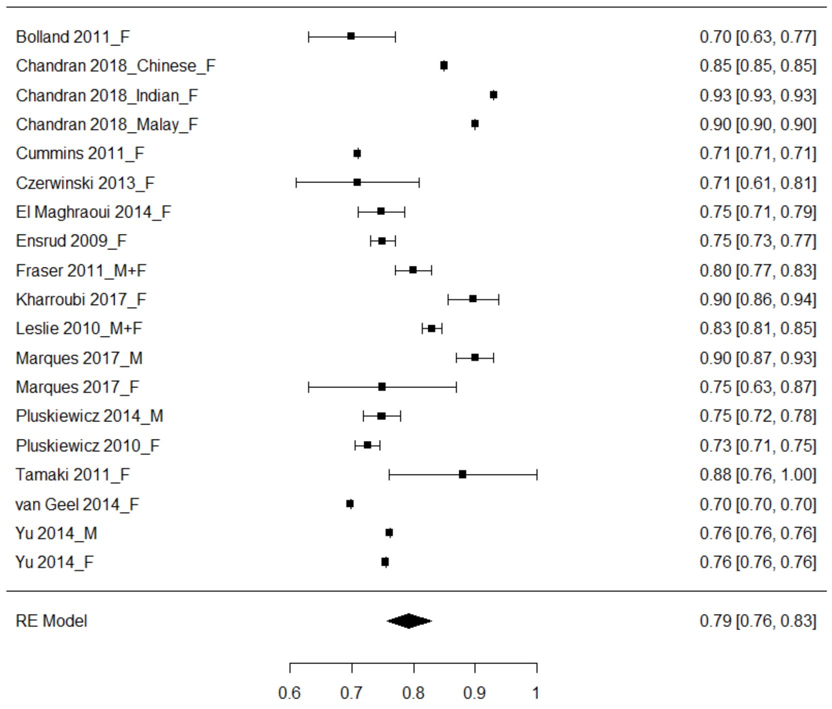

HIP fracture without BMD

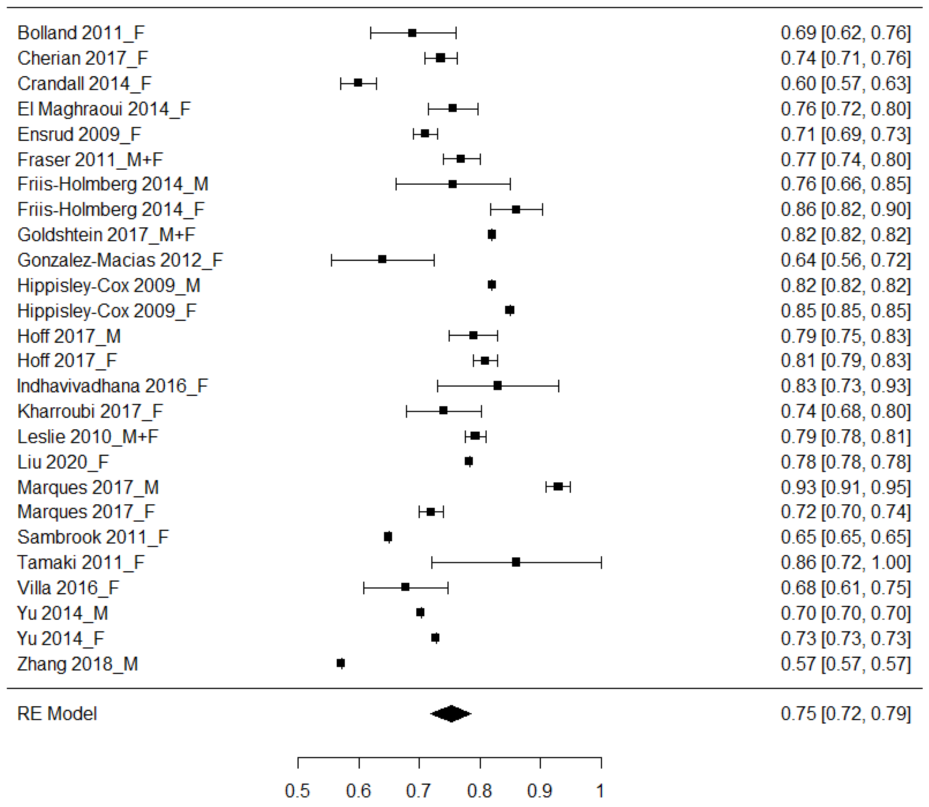

FRA-HS Tool

MEN, WOMEN, TOTAL POPULATION

MOF and HIP fracture without BMD

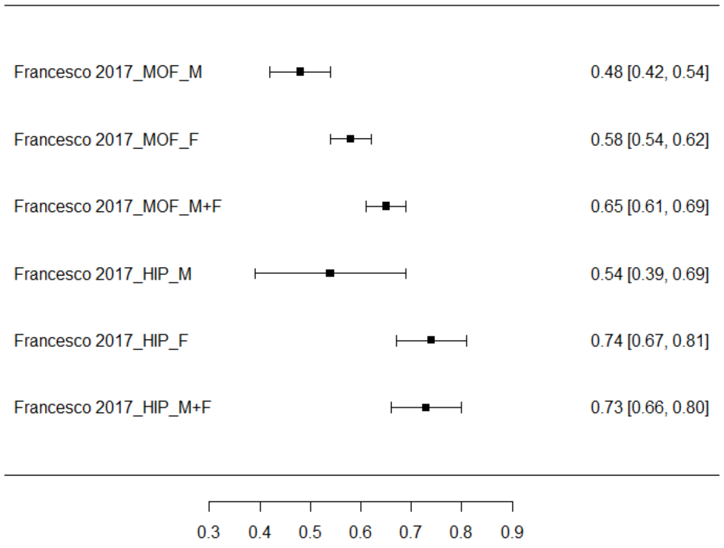

DeFRA and FRAX Tools

WOMEN

MOF with BMD

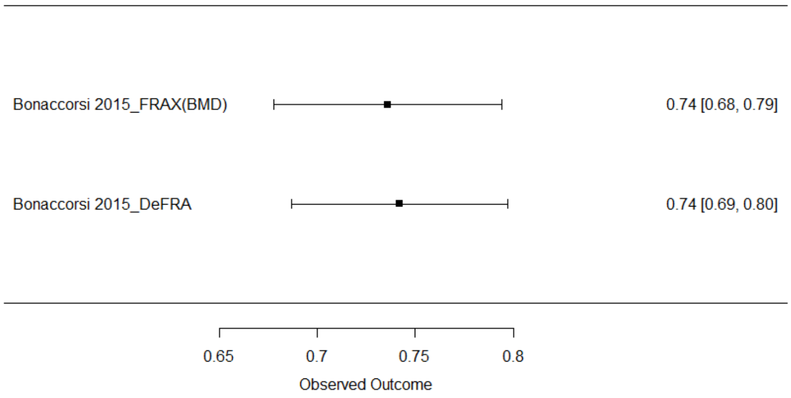

DIABETIC POPULATION

MOF with BMD

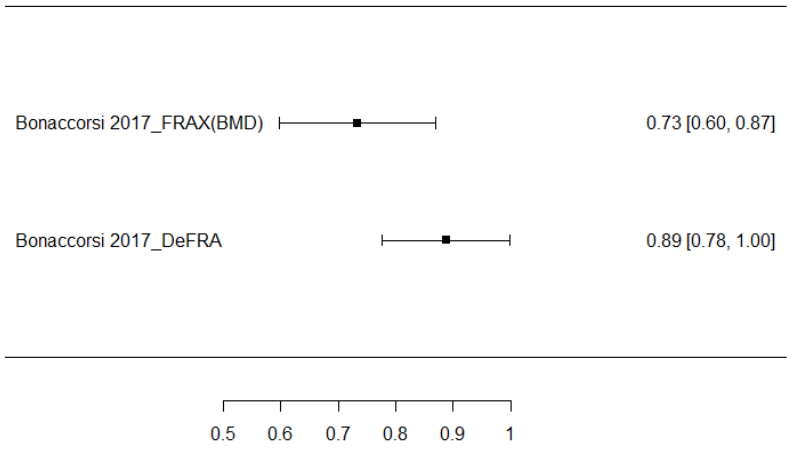

Quality evaluation

Diagnostic accuracy studies evaluated with the QUADAS-2

Study	***Risk of Bias***				***Applicability concerns***		
	Patient selection	***Index test***	***Reference standard***	***Flow and timing***	***Patient selection***	***Index test***	***Reference standard***
Bonaccorsi 2015	Low	Low	Low	Low	Yes	Yes	Yes
Dagan 2017	Low	Low	Low	Low	Yes	Yes	Yes
Fraser 2011	Low	Low	Low	Low	Yes	Yes	Yes
Friis - Holmberg 2014	Low	Low	Low	Low	Yes	Yes	Yes
Goldshtein 2018	Low	Low	Low	Low	Yes	Yes	Yes
González-Macías 2012	Low	Low	Low	Low	Yes	Yes	Yes
Hoff 2017	Low	Low	Low	Low	Yes	Yes	Yes
Lapi 2017	Low	Low	Low	Low	Yes	Yes	Yes
Leslie 2010	Low	Low	Low	Low	Yes	Yes	Yes
Marques 2017	Low	Low	Low	Low	Yes	Yes	Yes
Sandhu 2010	High	Low	Low	Low	Unclear	Yes	Yes
Su 2017	Unclear	Low	Low	Low	Yes	Yes	Yes
Tamaki 2011	Low	Low	Low	Low	Yes	Yes	Yes
van Geel 2014	Low	Low	Low	Low	Yes	Yes	Yes

Yu 2014	Low	Low	Low	Low	Yes	Yes	Yes
Zhang 2018	Low	Low	Low	Low	Yes	Yes	Yes
Pluskewicz 2014	Unclear	Low	Low	Low	Unclear	Yes	Yes
Lin 2016	High	Low	Low	Low	Unclear	Yes	Yes
Bonaccorsi 2017	High	Low	Low	Low	Unclear	Yes	Yes
Czerwiński 2013	Low	Low	Low	Low	Yes	Yes	Yes
Villa 2016	Low	Low	Low	Low	Yes	Yes	Yes
Rubin 2013	Unclear	Low	Low	Low	Yes	Yes	Yes
Bolland 2011	Low	Low	Low	Low	Yes	Yes	Yes
Briot 2013	Low	Low	Low	Low	Yes	Yes	Yes
Ensrud 2009	Low	Low	Low	Low	Yes	Yes	Yes
Henry 2011	Low	Low	Low	Low	Yes	Yes	Yes
Pluskiewicz 2010	Low	Low	Low	Low	Yes	Yes	Yes
Sornay-Rendu 2010	Low	Low	Low	Low	Yes	Yes	Yes
Trémollierres 2010	Low	Low	Low	Low	Yes	Yes	Yes
Cherian 2018	Unclear	Low	Low	Low	Yes	Yes	Yes
Kharroubi 2017	Unclear	Low	Low	Low	Yes	Yes	Yes
Chandran 2018	Low	Low	Low	Low	Yes	Yes	Yes
Kral 2017	High	Low	Low	Low	Yes	Yes	Yes
Crandall 2014	Unclear	Low	Low	Low	Yes	Yes	Yes
Cheung 2014	Low	Low	Low	Low	Yes	Yes	Yes

Liu 2020	Low	Low	Low	Low	Yes	Yes	Yes
El Maghraoui 2014	Low	Low	Low	Low	Yes	Yes	Yes
Indhavivaghana 2016	Unclear	Low	Low	Low	Yes	Yes	Yes
Tebé Cordomí 2013	Low	Low	Low	Low	Yes	Yes	Yes
Hippisley – Cox 2009	Low	Low	Low	Low	Yes	Yes	Yes
Sambrook 2011	Low	Low	Low	Low	Yes	Yes	Yes
Cummins 2011	High	Low	Low	Low	Unclear	Yes	Yes
Tanaka 2010	Low	Low	Low	Low	Unclear	Yes	Yes

Summary of Findings

MOF: FRAX without BMD at 3% used to diagnose fracture in fracture-free or fractured patients

Sensitivity (median)	0.57 (95% CI: 0.49 to 0.64)
Specificity (median)	0.69 (95% CI: 0.58 to 0.79)

Prevalences (median)	57%

Outcome	№ of studies (№ of patients)	Study design	Factors that may decrease certainty of evidence					Effect per 1.000 patients tested	Test accuracy CoE
			Risk of bias	Indirectness	Inconsistency	Imprecision	Publication bias	pre-test probability of 57%	
**True positives** (patients with fracture)	3 studies 498 patients	cross-sectional (cohort type accuracy study)	not serious	not serious	serious [a]	serious [b]	none	325 (279 to 365)	⊕⊕◯◯ LOW
**False negatives** (patients incorrectly classified as not having fracture)								245 (205 to 291)	
**True negatives** (patients without fracture)	3 studies 887 patients	cross-sectional (cohort type accuracy study)	not serious	not serious	serious [a]	very serious [b]	none	297 (249 to 340)	⊕◯◯◯ VERY LOW
**False positives** (patients incorrectly classified as having fracture)								133 (90 to 181)	

Explanations

a. Studies were downgraded by one increment for inconsistency (was assessed by inspection of the sensitivity/specificity RevMan 5.4 plots).

b. Downgrading by one increment was applied for confidence intervals 10-20% or by two increments for confidence intervals more than 20%.

MOF: FRAX without BMD at 5% used to diagnose fracture in fracture-free or fractured patients

Sensitivity (median)	0.34 (95% CI: 0.27 to 0.43)
Specificity (median)	0.85 (95% CI: 0.79 to 0.90)

Prevalences (median)	38%

Outcome	№ of studies (№ of patients)	Study design	Factors that may decrease certainty of evidence					Effect per 1.000 patients tested	Test accuracy CoE
			Risk of bias	Indirectness	Inconsistency	Imprecision	Publication bias	pre-test probability of 38%	
**True positives** (patients with fracture)	2 studies 287 patients	cross-sectional (cohort type accuracy study)	not serious	not serious	not serious	serious [a]	none	131 (101 to 163)	⨁⨁⨁◯ MODERATE
**False negatives** (patients incorrectly classified as not having fracture)								249 (217 to 279)	
**True negatives** (patients without fracture)	2 studies 815 patients	cross-sectional (cohort type accuracy study)	not serious	not serious	not serious	serious [a]	none	527 (487 to 558)	⨁⨁⨁◯ MODERATE
**False positives** (patients incorrectly classified as having fracture)								93 (62 to 133)	

Explanations

a. Downgrading by one increment was applied for confidence intervals 10-20% or by two increments for confidence intervals more than 20%.

MOF: FRAX without BMD at 10% used to diagnose fracture in fracture-free or fractured patients

Sensitivity (median)	0.57 (95% CI: 0.52 to 0.64)
Specificity (median)	0.66 (95% CI: 0.64 to 0.66)

Prevalences (median)	9%

Outcome	№ of studies (№ of patients)	Study design	Factors that may decrease certainty of evidence					Effect per 1.000 patients tested	Test accuracy CoE
			Risk of bias	Indirectness	Inconsistency	Imprecision	Publication bias	pre-test probability of 9%	
**True positives** (patients with fracture)	6 studies 4602 patients	cross-sectional (cohort type accuracy study)	not serious	not serious	very serious [a]	serious [b]	none	51 (47 to 58)	⨁◯◯◯ VERY LOW
**False negatives** (patients incorrectly classified as not having fracture)								39 (32 to 43)	
**True negatives** (patients without fracture)	6 studies 49855 patients	cross-sectional (cohort type accuracy study)	not serious	not serious	very serious [a]	not serious	none	596 (582 to 601)	⨁⨁◯◯ LOW
**False positives** (patients incorrectly classified as having fracture)								314 (309 to 328)	

Explanations

a. Studies were downgraded by one increment for inconsistency (was assessed by inspection of the sensitivity/specificity RevMan 5.4 plots).

b. Downgrading by one increment was applied for confidence intervals 10-20% or by two increments for confidence intervals more than 20%.

MOF: FRAX without BMD at 20% used to diagnose fracture in fracture-free or fractured patients

Sensitivity (median)	0.16 (95% CI: 0.13 to 0.20)
Specificity (median)	0.93 (95% CI: 0.93 to 0.94)

Prevalences (median)	9%

Outcome	№ of studies (№ of patients)	Study design	Factors that may decrease certainty of evidence					Effect per 1.000 patients tested	Test accuracy CoE
			Risk of bias	Indirectness	Inconsistency	Imprecision	Publication bias	pre-test probability of 9%	
**True positives** (patients with fracture)	3 studies 3226 patients	cross-sectional (cohort type accuracy study)	not serious	not serious	serious [a]	not serious	none	14 (12 to 18)	⨁⨁⨁◯ MODERATE
**False negatives** (patients incorrectly classified as not having fracture)								76 (72 to 78)	
**True negatives** (patients without fracture)	3 studies 43580 patients	cross-sectional (cohort type accuracy study)	not serious	not serious	serious [a]	not serious	none	846 (846 to 855)	⨁⨁⨁◯ MODERATE
**False positives** (patients incorrectly classified as having fracture)								64 (55 to 64)	

Explanations

a. Studies were downgraded by one increment for inconsistency (was assessed by inspection of the sensitivity/specificity RevMan 5.4 plots).

MOF: FRAX without BMD at 30% used to diagnose fracture in fracture-free or fractured patients

Sensitivity (median)	0.04 (95% CI: 0.03 to 0.11)
Specificity (median)	0.99 (95% CI: 0.98 to 0.99)

Prevalences (median)	9%

Outcome	№ of studies (№ of patients)	Study design	Factors that may decrease certainty of evidence					Effect per 1.000 patients tested	Test accuracy CoE
			Risk of bias	Indirectness	Inconsistency	Imprecision	Publication bias	pre-test probability of 9%	
**True positives** (patients with fracture)	3 studies 3227 patients	cross-sectional (cohort type accuracy study)	not serious	not serious	not serious	not serious	none	4 (3 to 10)	⨁⨁⨁⨁ HIGH
**False negatives** (patients incorrectly classified as not having fracture)								86 (80 to 87)	
**True negatives** (patients without fracture)	3 studies 43579 patients	cross-sectional (cohort type accuracy study)	not serious	not serious	not serious	not serious	none	901 (892 to 901)	⨁⨁⨁⨁ HIGH
**False positives** (patients incorrectly classified as having fracture)								9 (9 to 18)	

MOF: FRAX with BMD at 10% used to diagnose fracture in fracture-free or fractured patients

Sensitivity (median)	0.62 (95% CI: 0.52 to 0.70)
Specificity (median)	0.73 (95% CI: 0.72 to 0.75)

Prevalences (median)	9%

Outcome	№ of studies (№ of patients)	Study design	Factors that may decrease certainty of evidence					Effect per 1.000 patients tested	Test accuracy CoE
			Risk of bias	Indirectness	Inconsistency	Imprecision	Publication bias	pre-test probability of 9%	
**True positives** (patients with fracture)	7 studies 4706 patients	cross-sectional (cohort type accuracy study)	not serious	not serious	serious [a]	serious [b]	none	56 (47 to 63)	⨁⨁◯◯ LOW
**False negatives** (patients incorrectly classified as not having fracture)								34 (27 to 43)	
**True negatives** (patients without fracture)	7 studies 52204 patients	cross-sectional (cohort type accuracy study)	not serious	not serious	very serious [a]	not serious	none	664 (655 to 683)	⨁⨁◯◯ LOW
**False positives** (patients incorrectly classified as having fracture)								246 (227 to 255)	

Explanations

a. Studies were downgraded by one increment for inconsistency (was assessed by inspection of the sensitivity/specificity RevMan 5.4 plots).

b. Downgrading by one increment was applied for confidence intervals 10-20% or by two increments for confidence intervals more than 20%.

MOF: FRAX with BMD at 20% used to diagnose fracture in fracture-free or fractured patients

Sensitivity (median)	0.19 (95% CI: 0.17 to 0.21)
Specificity (median)	0.94 (95% CI: 0.93 to 0.94)

Prevalences (median)	10%

Outcome	№ of studies (№ of patients)	Study design	Factors that may decrease certainty of evidence					Effect per 1.000 patients tested	Test accuracy CoE
			Risk of bias	Indirectness	Inconsistency	Imprecision	Publication bias	pre-test probability of 10%	
**True positives** (patients with fracture)	6 studies 10685 patients	cross-sectional (cohort type accuracy study)	not serious	not serious	not serious	not serious	none	19 (17 to 21)	⊕⊕⊕⊕ HIGH
**False negatives** (patients incorrectly classified as not having fracture)								81 (79 to 83)	
**True negatives** (patients without fracture)	6 studies 98036 patients	cross-sectional (cohort type accuracy study)	not serious	not serious	not serious	not serious	none	842 (833 to 842)	⊕⊕⊕⊕ HIGH
**False positives** (patients incorrectly classified as having fracture)								58 (58 to 67)	

MOF: FRAX with BMD at 30% used to diagnose fracture in fracture-free or fractured patients

Sensitivity (median)	0.06 (95% CI: 0.04 to 0.08)
Specificity (median)	0.98 (95% CI: 0.98 to 0.99)

Prevalences (median)	7.5%

Outcome	№ of studies (№ of patients)	Study design	Factors that may decrease certainty of evidence					Effect per 1.000 patients tested	Test accuracy CoE
			Risk of bias	Indirectness	Inconsistency	Imprecision	Publication bias	pre-test probability of 7.5%	
**True positives** (patients with fracture)	3 studies 3178 patients	cross-sectional (cohort type accuracy study)	not serious	not serious	not serious	not serious	none	5 (3 to 6)	⨁⨁⨁⨁ HIGH
**False negatives** (patients incorrectly classified as not having fracture)								70 (69 to 72)	
**True negatives** (patients without fracture)	3 studies 43628 patients	cross-sectional (cohort type accuracy study)	not serious	not serious	not serious	not serious	none	907 (907 to 916)	⨁⨁⨁⨁ HIGH
**False positives** (patients incorrectly classified as having fracture)								18 (9 to 18)	

HIP fracture: FRAX without BMD at 3% used to diagnose fracture in fracture-free or fractured patients

Sensitivity (median)	0.59 (95% CI: 0.55 to 0.64)	Prevalences (median)	1.5%
Specificity (median)	0.81 (95% CI: 0.79 to 0.82)		

Outcome	№ of studies (№ of patients)	Study design	Factors that may decrease certainty of evidence					Effect per 1.000 patients tested	Test accuracy CoE
			Risk of bias	Indirectness	Inconsistency	Imprecision	Publication bias	pre-test probability of 1.5%	
**True positives** (patients with fracture)	6 studies 8118 patients	cross-sectional (cohort type accuracy study)	not serious	not serious	very serious [a]	serious [b]	none	9 (8 to 10)	⨁◯◯◯ VERY LOW
**False negatives** (patients incorrectly classified as not having fracture)								6 (5 to 7)	
**True negatives** (patients without fracture)	6 studies 916671 patients	cross-sectional (cohort type accuracy study)	not serious	not serious	very serious [a]	not serious	none	803 (778 to 808)	⨁⨁◯◯ LOW
**False positives** (patients incorrectly classified as having fracture)								182 (177 to 207)	

Explanations

a. Studies were downgraded by one increment for inconsistency (was assessed by inspection of the sensitivity/specificity RevMan 5.4 plots).

b. Downgrading by one increment was applied for confidence intervals 10-20% or by two increments for confidence intervals more than 20%.

HIP fracture: FRAX without BMD at 5% used to diagnose fracture in fracture-free or fractured patients

Sensitivity (median)	0.47 (95% CI: 0.42 to 0.53)
Specificity (median)	0.82 (95% CI: 0.82 to 0.83)

Prevalences (median)	4%

Outcome	№ of studies (№ of patients)	Study design	Factors that may decrease certainty of evidence					Effect per 1.000 patients tested	Test accuracy CoE
			Risk of bias	Indirectness	Inconsistency	Imprecision	Publication bias	pre-test probability of 4%	
**True positives** (patients with fracture)	6 studies 8497 patients	cross-sectional (cohort type accuracy study)	not serious	not serious	very serious [a]	serious [b]	none	19 (17 to 21)	⨁◯◯◯ VERY LOW
**False negatives** (patients incorrectly classified as not having fracture)								21 (19 to 23)	
**True negatives** (patients without fracture)	6 studies 924487 patients	cross-sectional (cohort type accuracy study)	not serious	not serious	very serious [a]	not serious	none	787 (787 to 797)	⨁⨁◯◯ LOW
**False positives** (patients incorrectly classified as having fracture)								173 (163 to 173)	

Explanations

a. Studies were downgraded by one increment for inconsistency (was assessed by inspection of the sensitivity/specificity RevMan 5.4 plots).

b. Downgrading by one increment was applied for confidence intervals 10-20% or by two increments for confidence intervals more than 20%.

HIP fracture: FRAX with BMD at 3% used to diagnose fracture in fracture-free or fractured patients

Sensitivity (median)	0.57 (95% CI: 0.50 to 0.63)
Specificity (median)	0.80 (95% CI: 0.79 to 0.80)

Prevalences (median)	6%

Outcome	№ of studies (№ of patients)	Study design	Factors that may decrease certainty of evidence					Effect per 1.000 patients tested	Test accuracy CoE
			Risk of bias	Indirectness	Inconsistency	Imprecision	Publication bias	pre-test probability of 6%	
**True positives** (patients with fracture)	6 studies 3045 patients	cross-sectional (cohort type accuracy study)	not serious	not serious	not serious	serious [a]	none	34 (30 to 38)	⨁⨁⨁◯ MODERATE
**False negatives** (patients incorrectly classified as not having fracture)								26 (22 to 30)	
**True negatives** (patients without fracture)	6 studies 100431 patients	cross-sectional (cohort type accuracy study)	not serious	not serious	not serious	not serious	none	747 (743 to 752)	⨁⨁⨁⨁ HIGH
**False positives** (patients incorrectly classified as having fracture)								193 (188 to 197)	

Explanations

a. Downgrading by one increment was applied for confidence intervals 10-20% or by two increments for confidence intervals more than 20%.

HIP fracture: FRAX with BMD at 5% used to diagnose fracture in fracture-free or fractured patients

Sensitivity (median)	0.43 (95% CI: 0.35 to 0.51)
Specificity (median)	0.86 (95% CI: 0.84 to 0.88)

Prevalences (median)	6%

Outcome	№ of studies (№ of patients)	Study design	Factors that may decrease certainty of evidence					Effect per 1.000 patients tested	Test accuracy CoE
			Risk of bias	Indirectness	Inconsistency	Imprecision	Publication bias	pre-test probability of 6%	
**True positives** (patients with fracture)	5 studies 1372 patients	cross-sectional (cohort type accuracy study)	not serious	not serious	serious [a]	serious [b]	none	26 (21 to 31)	⨁⨁◯◯ LOW
**False negatives** (patients incorrectly classified as not having fracture)								34 (29 to 39)	
**True negatives** (patients without fracture)	5 studies 53089 patients	cross-sectional (cohort type accuracy study)	not serious	not serious	serious [a]	not serious	none	808 (790 to 827)	⨁⨁⨁◯ MODERATE
**False positives** (patients incorrectly classified as having fracture)								132 (113 to 150)	

Explanations

a. Studies were downgraded by one increment for inconsistency (was assessed by inspection of the sensitivity/specificity RevMan 5.4 plots).

b. Downgrading by one increment was applied for confidence intervals 10-20% or by two increments for confidence intervals more than 20%.

CQ 3

How can we identify patients at imminent risk of (re)fracture?

Search strategy

From the NICE guideline:

Review question 1

How useful are simple clinical measures for targeting people for risk assessment of fragility fracture?

**Population**	Adult men or women (over 18 years), including those without known osteoporosis or previous fragility fracture.
**Prognostic factor**	BMI, oral glucocorticoid use, family history of fracture, previous fracture, smoking, alcohol, history of falls.
**Outcomes**	Risk of fractures including:<br>• vertebral<br>• hip<br>• forearm<br>• any fragility fracture.

MEDLINE

Up to 9 February 2021

BODY MASS INDEX

#1:

((wrist*[ti] or colles[ti] or radius[ti] or “articulatio radiocarpea” [ti] or carpus[ti] or carpal[ti] or radiocarp*[ti] or radial[ti] or forearm*[ti] or humerus[ti] or metacarp*[ti] or barton[ti] or monteggi*[ti] or ulna[ti] or ulnar[ti] or limb*[ti] or hip[ti] or hips[ti] or trochanteric[ti] or intertrochanteric[ti] or subtrochanteric[ti] or femoral[ti] or femur[ti] or spine[ti] or spinal[ti] or vertebra[ti] or vertebral[ti] or vertebrae[ti] or lumbar[ti] or shoulder*[ti] or glenohumeral[ti] or humeroscapular[ti] or humeral[ti] or radius[ti] or wrist[ti] or fragil*[ti] osteoporosis[ti] or osteoporo*[ti]) AND fractur*[ti])

#2:

“fragility fracture”[ti] OR “fragility fractures”[ti] OR “low energy fracture”[ti] OR “low energy fractures”[ti] OR “low-energy fracture”[ti] OR “low-energy fractures”[ti] OR “low trauma fracture”[ti] OR “low trauma fractures”[ti] OR “low-trauma fracture”[ti] OR “low-trauma fractures”[ti] OR “low energy trauma”[ti] OR “low-energy trauma”[ti] OR “low level trauma”[ti] OR “low-level trauma”[ti] OR “minor trauma fracture”[ti] OR “minor trauma fractures”[ti] OR “minor-trauma fracture”[ti] OR “minor-trauma fractures”[ti] OR “minor fracture”[ti] OR “minor

fractures”[ti] OR “minor-fracture”[ti] OR “minor-fractures”[ti] OR “osteoporotic fracture”[ti] OR “osteoporotic fractures”[ti]

#3: #1 OR #2

#4:

BMI[tiab] OR “body mass index”[tiab] OR adipos*[tiab] OR obes*[tiab] OR thinness[tiab] OR anorex*[tiab] OR bodymass[tiab] OR bodyweight[tiab] OR “body mass”[tiab] OR weight*[tiab] OR overweight[tiab] OR underweight[tiab] OR "Body Mass Index"[Mesh] OR "Body Weight"[Mesh] OR "Weight Loss"[Mesh] OR "Weight Gain"[Mesh] OR "Body Weight Changes"[Mesh]

#5 (#3 AND #4) AND limit: Humans

EMBASE

Up to 9 February 2021

#1:

((wrist*:ti or colles:ti or radius:ti or “articulatio radiocarpea”:ti or carpus:ti or carpal:ti or radiocarp*:ti or radial:ti or forearm*:ti or humerus:ti or metacarp*:ti or barton:ti or monteggi*:ti or ulna:ti or ulnar:ti or limb*:ti or hip:ti or hips:ti or trochanteric:ti or intertrochanteric:ti or subtrochanteric:ti or femoral:ti or femur:ti or spine:ti or spinal:ti or vertebra:ti or vertebral:ti or vertebrae:ti or lumbar:ti or shoulder*:ti or glenohumeral:ti or humeroscapular:ti or humeral:ti or radius:ti or wrist:ti or fragil*:ti osteoporosis:ti or osteoporo*:ti) AND fractur*:ti)

#2: 'fragility fracture'/exp

#3: 'low energy fracture'/exp

#4: 'low trauma fracture'/exp

#5: 'low energy trauma'/exp

#6:

“fragility fracture”:ti OR “fragility fractures”:ti OR “low energy fracture”:ti OR “low energy fractures”:ti OR “low-energy fracture”:ti OR “low-energy fractures”:ti OR “low trauma fracture”:ti OR “low trauma fractures”:ti OR “low-trauma fracture”:ti OR “low-trauma fractures”:ti OR “low energy trauma”:ti OR “low-energy trauma”:ti OR “low level trauma”:ti OR “low-level trauma”:ti OR “minor trauma fracture”:ti OR “minor trauma fractures”:ti OR “minor-trauma fracture”:ti OR “minor-trauma fractures”:ti OR “minor fracture”:ti OR “minor fractures”:ti OR “minor-fracture”:ti OR “minor-fractures”:ti OR “osteoporotic fracture”:ti OR “osteoporotic fractures”:ti

#7: #1 OR #2 OR #3 OR #4 OR #5 OR #6

#8:

BMI:ti,ab OR “body mass index”:ti,ab OR adipos*:ti,ab OR obes*:ti,ab OR thinness:ti,ab OR anorex*:ti,ab OR bodymass:ti,ab OR bodyweight:ti,ab OR “body mass”:ti,ab OR weight*:ti,ab OR overweight:ti,ab OR underweight:ti,ab

#9: 'body weight'

#10: 'obesity'

#11: 'body weight change'

#12: 'body weight fluctuation'

#13: 'body weight gain'

#14: 'body weight loss'

#15: 'body weight disorder'

#16: 'underweight'

#17: #8 OR #9 OR #10 OR #11 OR #12 OR #13 OR #14 OR #15 OR #16

#18: #7 AND #17

#19: #18 NOT ((MH "Animals+") OR (MH "Animal Studies") OR (TI "animal model*"))

COCHRANE SEARCH

Up to 9 February 2021

#1:

((wrist*:ti or colles:ti or radius:ti or “articulatio radiocarpea”:ti or carpus:ti or carpal:ti or radiocarp*:ti or radial:ti or forearm*:ti or humerus:ti or metacarp*:ti or barton:ti or monteggi*:ti or ulna:ti or ulnar:ti or limb*:ti or hip:ti or hips:ti or trochanteric:ti or intertrochanteric:ti or subtrochanteric:ti or femoral:ti or femur:ti or spine:ti or spinal:ti or vertebra:ti or vertebral:ti or vertebrae:ti or lumbar:ti or shoulder*:ti or glenohumeral:ti or humeroscapular:ti or humeral:ti or radius:ti or wrist:ti or fragil*:ti osteoporosis:ti or osteoporo*:ti) AND fractur*:ti)

#2: MeSH descriptor: [Osteoporotic Fractures] explode all trees

#3: MeSH descriptor: [Fractures, Spontaneous] explode all trees

#4:

(fragility fracture):ti OR (fragility fractures):ti OR (low energy fracture):ti OR (low energy fractures):ti OR (low-energy fracture):ti OR (low-energy fractures):ti OR (low trauma fracture):ti OR (low trauma fractures):ti OR (low-trauma fracture):ti OR (low-trauma fractures):ti OR (low energy trauma):ti OR (low-energy trauma):ti OR (low level trauma):ti OR (low-level trauma):ti OR (minor trauma fracture):ti OR (minor trauma fractures):ti OR (minor-trauma fracture):ti OR (minor-trauma

fractures):ti OR (minor fracture):ti OR (minor fractures):ti OR (minor-fracture):ti OR (minor-fractures):ti OR (osteoporotic fracture):ti OR (osteoporotic fractures):ti OR (pathologic fracture):ti OR (pathological fractures):ti

#5: #1 OR #2 OR #3 OR #4

#6:

BMI:ti,ab OR "body mass index":ti,ab OR adipos*:ti,ab OR obes*:ti,ab OR thinness:ti,ab OR anorex*:ti,ab OR bodymass:ti,ab OR bodyweight:ti,ab OR "body mass":ti,ab OR weight*:ti,ab OR overweight:ti,ab OR underweight:ti,ab

#7: MeSH descriptor: [Body Weight] explode all trees

#8: MeSH descriptor: [Obesity] explode all trees

#9: MeSH descriptor: [Body Weight Changes] explode all trees

#10: MeSH descriptor: [Thinness] explode all trees

#11: #6 OR #7 OR #8 OR #9 OR #10

#12: #5 AND #11

GLUCOCORTICOIDS

MEDLINE

Up to 9 February 2021

#1:

"fragility fracture"[ti] OR "fragility fractures"[ti] OR "low energy fracture"[ti] OR "low energy fractures"[ti] OR "low-energy fracture"[ti] OR "low-energy fractures"[ti] OR "low trauma fracture"[ti] OR "low trauma fractures"[ti] OR "low-trauma fracture"[ti] OR "low-trauma fractures"[ti] OR "low energy trauma"[ti] OR "low-energy trauma"[ti] OR "low level trauma"[ti] OR "low-level trauma"[ti] OR "minor trauma fracture"[ti] OR "minor trauma fractures"[ti] OR "minor-trauma fracture"[ti] OR "minor-trauma fractures"[ti] OR "minor fracture"[ti] OR "minor fractures"[ti] OR "minor-fracture"[ti] OR "minor-fractures"[ti] OR "osteoporotic fracture"[ti] OR "osteoporotic fractures"[ti] or fracture[ti]

#2:

glucocorticoid*[tiab] or steroid*[tiab] or corticosteroid*[tiab] or budesonide[tiab] or entocort[tiab] or budenofalk[tiab] or "mometasone furoate"[tiab] or asmanex[tiab] or betamethasone[tiab] or betametasone[tiab] or betnelan[tiab] or betnesol[tiab] or cortisone[tiab] or deflazacort[tiab] or calcot[tiab] or dexamethasone[tiab] or hydrocortisone[tiab] or efcortesol[tiab] or solu-cortef[tiab] or methylprednisolone[tiab] or medrone[tiab] or solu-medrone[tiab] or prednisolone[tiab] or

prednisone[tiab] or lodotra[tiab] or "Adrenal Cortex Hormones"[Mesh] OR "Glucocorticoids"[Mesh] OR "Budesonide"[Mesh] OR "Betamethasone"[Mesh] OR "Dexamethasone"[Mesh] OR "Hydrocortisone"[Mesh] OR "Methylprednisolone"[Mesh] OR "Prednisolone"[Mesh]

#5: (#3 AND #4) AND limit: Humans

EMBASE

Up to 9 February 2021

#1:

“fragility fracture”:ti OR “fragility fractures”:ti OR “low energy fracture”:ti OR “low energy fractures”:ti OR “low-energy fracture”:ti OR “low-energy fractures”:ti OR “low trauma fracture”:ti OR “low trauma fractures”:ti OR “low-trauma fracture”:ti OR “low-trauma fractures”:ti OR “low energy trauma”:ti OR “low-energy trauma”:ti OR “low level trauma”:ti OR “low-level trauma”:ti OR “minor trauma fracture”:ti OR “minor trauma fractures”:ti OR “minor-trauma fracture”:ti OR “minor-trauma fractures”:ti OR “minor fracture”:ti OR “minor fractures”:ti OR “minor-fracture”:ti OR “minor-fractures”:ti OR “osteoporotic fracture”:ti OR “osteoporotic fractures”:ti OR fracture:ti

#2:

glucocorticoid*:ti,ab or steroid*:ti,ab or corticosteroid*:ti,ab or budesonide:ti,ab or entocort:ti,ab or budenofalk:ti,ab or “mometasone furoate”:ti,ab or asmanex:ti,ab or betamethasone:ti,ab or betametasone:ti,ab or betnelan:ti,ab or betnesol:ti,ab or cortisone:ti,ab or deflazacort:ti,ab or calcot:ti,ab or dexamethasone:ti,ab or hydrocortisone:ti,ab or efcortesol:ti,ab or solu-cortef:ti,ab or methylprednisolone:ti,ab or medrone:ti,ab or solu-medrone:ti,ab or prednisolone:ti,ab or prednisone:ti,ab or lodotra:ti,ab

#3: 'corticosteroid'

#4: 'glucocorticoid'

#5: 'budesonide'

#6: 'betamethasone'

#7: 'dexamethasone'

#8: 'hydrocortisone'

#9: 'methylprednisolone'

#10: 'prednisolone'

#11: #2 OR #3 OR #4 OR #5 OR #6 OR #7 OR #8 OR #9 OR #10

#12: #1 AND #11

COCHRANE

Up to 9 February 2021

#1:

(fragility fracture):ti OR (fragility fractures):ti OR (low energy fracture):ti OR (low energy fractures):ti OR (low-energy fracture):ti OR (low-energy fractures):ti OR (low trauma fracture):ti OR (low trauma fractures):ti OR (low-trauma fracture):ti OR (low-trauma fractures):ti OR (low energy trauma):ti OR (low-energy trauma):ti OR (low level trauma):ti OR (low-level trauma):ti OR (minor trauma fracture):ti OR (minor trauma fractures):ti OR (minor-trauma fracture):ti OR (minor-trauma fractures):ti OR (minor fracture):ti OR (minor fractures):ti OR (minor-fracture):ti OR (minor-fractures):ti OR (osteoporotic fracture):ti OR (osteoporotic fractures):ti OR (pathologic fracture):ti OR (pathological fractures):ti

#2:

glucocorticoid*:ti,ab or steroid*:ti,ab or corticosteroid*:ti,ab or budesonide:ti,ab or entocort:ti,ab or budenofalk:ti,ab or "mometasone furoate":ti,ab or asmanex:ti,ab or betamethasone:ti,ab or betametasone:ti,ab or betnelan:ti,ab or betnesol:ti,ab or cortisone:ti,ab or deflazacort:ti,ab or calcot:ti,ab or dexamethasone:ti,ab or hydrocortisone:ti,ab or efcortesol:ti,ab or solu-cortef:ti,ab or methylprednisolone:ti,ab or medrone:ti,ab or solu-medrone:ti,ab or prednisolone:ti,ab or prednisone:ti,ab or lodotra:ti,ab

#3: MeSH descriptor: [Adrenal Cortex Hormones] explode all trees

#4: MeSH descriptor: [Glucocorticoids] explode all trees

#5: MeSH descriptor: [Budesonide] explode all trees

#6: MeSH descriptor: [Betamethasone] explode all trees

#7: MeSH descriptor: [Dexamethasone] explode all trees

#8: MeSH descriptor: [Hydrocortisone] explode all trees

#9: MeSH descriptor: [Methylprednisolone] explode all trees

#10: MeSH descriptor: [Prednisolone] explode all trees

#11: #2 OR #3 OR #4 OR #5 OR #6 OR #7 OR #8 OR #9 OR #10

#12: #1 AND #11

PREVIOUS FALL

MEDLINE

Up to 9 February 2021

#1:

((wrist*[ti] or colles[ti] or radius[ti] or "articulatio radiocarpea" [ti] or carpus[ti] or carpal[ti] or radiocarp*[ti] or radial[ti] or forearm*[ti] or humerus[ti] or metacarp*[ti] or barton[ti] or monteggi*[ti] or ulna[ti] or ulnar[ti] or limb*[ti] or hip[ti] or hips[ti] or trochanteric[ti] or intertrochanteric[ti] or subtrochanteric[ti] or femoral[ti] or femur[ti] or spine[ti] or spinal[ti] or vertebra[ti] or vertebral[ti] or vertebrae[ti] or lumbar[ti] or shoulder*[ti] or glenohumeral[ti] or humeroscapular[ti] or humeral[ti] or radius[ti] or wrist[ti] or fragil*[ti] osteoporosis[ti] or osteoporo*[ti]) AND fractur*[ti])

#2:

"fragility fracture"[ti] OR "fragility fractures"[ti] OR "low energy fracture"[ti] OR "low energy fractures"[ti] OR "low-energy fracture"[ti] OR "low-energy fractures"[ti] OR "low trauma fracture"[ti] OR "low trauma fractures"[ti] OR "low-trauma fracture"[ti] OR "low-trauma fractures"[ti] OR "low energy trauma"[ti] OR "low-energy trauma"[ti] OR "low level trauma"[ti] OR "low-level trauma"[ti] OR "minor trauma fracture"[ti] OR "minor trauma fractures"[ti] OR "minor-trauma fracture"[ti] OR "minor-trauma fractures"[ti] OR "minor fracture"[ti] OR "minor fractures"[ti] OR "minor-fracture"[ti] OR "minor-fractures"[ti] OR "osteoporotic fracture"[ti] OR "osteoporotic fractures"[ti]

#3: #1 OR #2

#4: fall[tiab] OR "Accidental Falls"[Mesh]

#5: (#3 AND #4) AND limit: Humans

EMBASE

Up to 8 February 2021

#1:

((wrist*:ti or colles:ti or radius:ti or "articulatio radiocarpea":ti or carpus:ti or carpal:ti or radiocarp*:ti or radial:ti or forearm*:ti or humerus:ti or metacarp*:ti or barton:ti or monteggi*:ti or ulna:ti or ulnar:ti or limb*:ti or hip:ti or hips:ti or trochanteric:ti or intertrochanteric:ti or subtrochanteric:ti or femoral:ti or femur:ti or spine:ti or spinal:ti or vertebra:ti or vertebral:ti or vertebrae:ti or lumbar:ti or shoulder*:ti or glenohumeral:ti or humeroscapular:ti or humeral:ti or radius:ti or wrist:ti or fragil*:ti osteoporosis:ti or osteoporo*:ti) AND fractur*:ti)

#2: 'fragility fracture'/exp

#3: 'low energy fracture'/exp

#4: 'low trauma fracture'/exp

#5: 'low energy trauma'/exp

#6:

“fragility fracture”:ti OR “fragility fractures”:ti OR “low energy fracture”:ti OR “low energy fractures”:ti OR “low-energy fracture”:ti OR “low-energy fractures”:ti OR “low trauma fracture”:ti OR “low trauma fractures”:ti OR “low-trauma fracture”:ti OR “low-trauma fractures”:ti OR “low energy trauma”:ti OR “low-energy trauma”:ti OR “low level trauma”:ti OR “low-level trauma”:ti OR “minor trauma fracture”:ti OR “minor trauma fractures”:ti OR “minor-trauma fracture”:ti OR “minor-trauma fractures”:ti OR “minor fracture”:ti OR “minor fractures”:ti OR “minor-fracture”:ti OR “minor-fractures”:ti OR “osteoporotic fracture”:ti OR “osteoporotic fractures”:ti

#7: #1 OR #2 OR #3 OR #4 OR #5 OR #6

#8: fall:ti,ab

#9: 'falling'

#10: #8 OR #9

#11: #7 AND #10

#12: #11 AND [embase]/lim NOT ([embase]/lim AND [medline]/lim)

COCHRANE

Up to 8 February 2021

#1:

((wrist*:ti or colles:ti or radius:ti or “articulatio radiocarpea”:ti or carpus:ti or carpal:ti or radiocarp*:ti or radial:ti or forearm*:ti or humerus:ti or metacarp*:ti or barton:ti or monteggi*:ti or ulna:ti or ulnar:ti or limb*:ti or hip:ti or hips:ti or trochanteric:ti or intertrochanteric:ti or subtrochanteric:ti or femoral:ti or femur:ti or spine:ti or spinal:ti or vertebra:ti or vertebral:ti or vertebrae:ti or lumbar:ti or shoulder*:ti or glenohumeral:ti or humeroscapular:ti or humeral:ti or radius:ti or wrist:ti or fragil*:ti osteoporosis:ti or osteoporo*:ti) AND fractur*:ti)

#2: MeSH descriptor: [Osteoporotic Fractures] explode all trees

#3: MeSH descriptor: [Fractures, Spontaneous] explode all trees

#4:

(fragility fracture):ti OR (fragility fractures):ti OR (low energy fracture):ti OR (low energy fractures):ti OR (low-energy fracture):ti OR (low-energy fractures):ti OR (low trauma fracture):ti OR

(low trauma fractures):ti OR (low-trauma fracture):ti OR (low-trauma fractures):ti OR (low energy trauma):ti OR (low-energy trauma):ti OR (low level trauma):ti OR (low-level trauma):ti OR (minor trauma fracture):ti OR (minor trauma fractures):ti OR (minor-trauma fracture):ti OR (minor-trauma fractures):ti OR (minor fracture):ti OR (minor fractures):ti OR (minor-fracture):ti OR (minor-fractures):ti OR (osteoporotic fracture):ti OR (osteoporotic fractures):ti OR (pathologic fracture):ti OR (pathological fractures):ti

#5: #1 OR #2 OR #3 OR #4

#6: fall:ti,ab

#7: MeSH descriptor: [Accidental Falls] explode all trees

#8: #6 OR #7

#9: #5 AND #8

PREVIOUS FRACTURE

MEDLINE

Up to 10 February 2021

#1:

((wrist*[ti] or colles[ti] or radius[ti] or “articulatio radiocarpea” [ti] or carpus[ti] or carpal[ti] or radiocarp*[ti] or radial[ti] or forearm*[ti] or humerus[ti] or metacarp*[ti] or barton[ti] or monteggi*[ti] or ulna[ti] or ulnar[ti] or limb*[ti] or hip[ti] or hips[ti] or trochanteric[ti] or intertrochanteric[ti] or subtrochanteric[ti] or femoral[ti] or femur[ti] or spine[ti] or spinal[ti] or vertebra[ti] or vertebral[ti] or vertebrae[ti] or lumbar[ti] or shoulder*[ti] or glenohumeral[ti] or humeroscapular[ti] or humeral[ti] or radius[ti] or wrist[ti] or fragil*[ti] osteoporosis[ti] or osteoporo*[ti] OR torax[ti] OR chest[ti] OR clavicle[ti] OR rib[ti] OR pelvis[ti] OR tibia[ti] OR fragil*[ti] OR any[ti] OR ankle[ti]) AND fractur*[ti])

#2:

“fragility fracture”[tiab] OR “fragility fractures”[tiab] OR “low energy fracture”[tiab] OR “low energy fractures”[tiab] OR “low-energy fracture”[tiab] OR “low-energy fractures”[tiab] OR “low trauma fracture”[tiab] OR “low trauma fractures”[tiab] OR “low-trauma fracture”[tiab] OR “low-trauma fractures”[tiab] OR “low energy trauma”[tiab] OR “low-energy trauma”[tiab] OR “low level trauma”[tiab] OR “low-level trauma”[tiab] OR “minor trauma fracture”[tiab] OR “minor trauma fractures”[tiab] OR “minor-trauma fracture”[tiab] OR “minor-trauma fractures”[tiab] OR “minor fracture”[tiab] OR “minor fractures”[tiab] OR “minor-fracture”[tiab] OR “minor-fractures”[tiab] OR “osteoporotic fracture”[tiab] OR “osteoporotic fractures”[tiab] OR osteopor*[tiab]

#3: #1 AND #2

#4:

((recurrent[tiab] or recurring[tiab] or repeated[tiab] or history[tiab] or chronic[tiab] or previous[tiab] or prior[tiab] or habitual[tiab]) AND fracture*[tiab]) OR "Medical History Taking"[Mesh]

#5: (#3 AND #4) AND limit: Humans

EMBASE

Up to 10 February 2021

#1:

((wrist*:ti or colles:ti or radius:ti or “articulatio radiocarpea”:ti or carpus:ti or carpal:ti or radiocarp*:ti or radial:ti or forearm*:ti or humerus:ti or metacarp*:ti or barton:ti or monteggi*:ti or ulna:ti or ulnar:ti or limb*:ti or hip:ti or hips:ti or trochanteric:ti or intertrochanteric:ti or subtrochanteric:ti or femoral:ti or femur:ti or spine:ti or spinal:ti or vertebra:ti or vertebral:ti or vertebrae:ti or lumbar:ti or shoulder*:ti or glenohumeral:ti or humeroscapular:ti or humeral:ti or radius:ti or wrist:ti or fragil*:ti osteoporosis:ti or osteoporo*:ti OR torax:ti OR chest:ti OR clavicle:ti OR rib:ti OR pelvis:ti OR tibia:ti OR fragil*:ti OR any:ti OR ankle:ti) AND fractur*:ti)

#2:

“fragility fracture”:ti,ab OR “fragility fractures”:ti,ab OR “low energy fracture”:ti,ab OR “low energy fractures”:ti,ab OR “low-energy fracture”:ti,ab OR “low-energy fractures”:ti,ab OR “low trauma fracture”:ti,ab OR “low trauma fractures”:ti,ab OR “low-trauma fracture”:ti,ab OR “low-trauma fractures”:ti,ab OR “low energy trauma”:ti,ab OR “low-energy trauma”:ti,ab OR “low level trauma”:ti,ab OR “low-level trauma”:ti,ab OR “minor trauma fracture”:ti,ab OR “minor trauma fractures”:ti,ab OR “minor-trauma fracture”:ti,ab OR “minor-trauma fractures”:ti,ab OR “minor fracture”:ti,ab OR “minor fractures”:ti,ab OR “minor-fracture”:ti,ab OR “minor-fractures”:ti,ab OR “osteoporotic fracture”:ti,ab OR “osteoporotic fractures”:ti,ab OR osteopor*:ti,ab

#3: #1 AND #2

#4: ((recurrent:ti,ab or recurring:ti,ab or repeated:ti,ab or history:ti,ab or chronic:ti,ab or previous:ti,ab or prior:ti,ab or habitual:ti,ab) AND fracture*:ti,ab)

#5: 'medical history'

#6: #4 OR #5

#7: #3 AND #6

#8: #7 AND [embase]/lim NOT ([embase]/lim AND [medline]/lim)

COCHRANE

Up to 10 February 2021

#1:

((wrist*:ti or colles:ti or radius:ti or “articulatio radiocarpea”:ti or carpus:ti or carpal:ti or radiocarp*:ti or radial:ti or forearm*:ti or humerus:ti or metacarp*:ti or barton:ti or monteggi*:ti or ulna:ti or ulnar:ti or limb*:ti or hip:ti or hips:ti or trochanteric:ti or intertrochanteric:ti or subtrochanteric:ti or femoral:ti or femur:ti or spine:ti or spinal:ti or vertebra:ti or vertebral:ti or vertebrae:ti or lumbar:ti or shoulder*:ti or glenohumeral:ti or humeroscapular:ti or humeral:ti or radius:ti or wrist:ti or fragil*:ti osteoporosis:ti or osteoporo*:ti OR torax:ti OR chest:ti OR clavicle:ti OR rib:ti OR pelvis:ti OR tibia:ti OR fragil*:ti OR any:ti OR ankle:ti) AND fractur*:ti)

#2:

(“fragility fracture”:ti,ab OR “fragility fractures”:ti,ab OR “low energy fracture”:ti,ab OR “low energy fractures”:ti,ab OR “low-energy fracture”:ti,ab OR “low-energy fractures”:ti,ab OR “low trauma fracture”:ti,ab OR “low trauma fractures”:ti,ab OR “low-trauma fracture”:ti,ab OR “low-trauma fractures”:ti,ab OR “low energy trauma”:ti,ab OR “low-energy trauma”:ti,ab OR “low level trauma”:ti,ab OR “low-level trauma”:ti,ab OR “minor trauma fracture”:ti,ab OR “minor trauma fractures”:ti,ab OR “minor-trauma fracture”:ti,ab OR “minor-trauma fractures”:ti,ab OR “minor fracture”:ti,ab OR “minor fractures”:ti,ab OR “minor-fracture”:ti,ab OR “minor-fractures”:ti,ab OR “osteoporotic fracture”:ti,ab OR “osteoporotic fractures”:ti,ab OR osteopor*:ti,ab)

#3: #1 AND #2

#4:

((recurrent:ti,ab or recurring:ti,ab or repeated:ti,ab or history:ti,ab or chronic:ti,ab or previous:ti,ab or prior:ti,ab or habitual:ti,ab) AND fracture*:ti,ab)

#5: MeSH descriptor: [Medical History Taking] explode all trees

#6: #4 OR #5

#7: #3 AND #6

FAMILY HISTORY OF FRACTURE

MEDLINE

Up to 8 February 2021

#1:

((wrist*[ti] or colles[ti] or radius[ti] or “articulatio radiocarpea” [ti] or carpus[ti] or carpal[ti] or radiocarp*[ti] or radial[ti] or forearm*[ti] or humerus[ti] or metacarp*[ti] or barton[ti] or

monteggi*[ti] or ulna[ti] or ulnar[ti] or limb*[ti] or hip[ti] or hips[ti] or trochanteric[ti] or intertrochanteric[ti] or subtrochanteric[ti] or femoral[ti] or femur[ti] or spine[ti] or spinal[ti] or vertebra[ti] or vertebral[ti] or vertebrae[ti] or lumbar[ti] or shoulder*[ti] or glenohumeral[ti] or humeroscapular[ti] or humeral[ti] or radius[ti] or wrist[ti] or fragil*[ti] osteoporosis[ti] or osteoporo*[ti]) AND fractur*[ti])

#2:

"fragility fracture"[ti] OR "fragility fractures"[ti] OR "low energy fracture"[ti] OR "low energy fractures"[ti] OR "low-energy fracture"[ti] OR "low-energy fractures"[ti] OR "low trauma fracture"[ti] OR "low trauma fractures"[ti] OR "low-trauma fracture"[ti] OR "low-trauma fractures"[ti] OR "low energy trauma"[ti] OR "low-energy trauma"[ti] OR "low level trauma"[ti] OR "low-level trauma"[ti] OR "minor trauma fracture"[ti] OR "minor trauma fractures"[ti] OR "minor-trauma fracture"[ti] OR "minor-trauma fractures"[ti] OR "minor fracture"[ti] OR "minor fractures"[ti] OR "minor-fracture"[ti] OR "minor-fractures"[ti] OR "osteoporotic fracture"[ti] OR "osteoporotic fractures"[ti]

#3: #1 OR #2

#4:

((familial[tiab] or inherit*[tiab] or heredit*[tiab] or predispos*[tiab] or susceptib*[tiab] OR family[tiab] or maternal[tiab] or parental[tiab]) AND histor*[tiab]) OR "Genetic Predisposition to Disease"[Mesh]

#5: (#3 AND #4) AND limit: Humans

EMBASE

Up to 8 February 2021

#1:

((wrist*:ti or colles:ti or radius:ti or "articulatio radiocarpea":ti or carpus:ti or carpal:ti or radiocarp*:ti or radial:ti or forearm*:ti or humerus:ti or metacarp*:ti or barton:ti or monteggi*:ti or ulna:ti or ulnar:ti or limb*:ti or hip:ti or hips:ti or trochanteric:ti or intertrochanteric:ti or subtrochanteric:ti or femoral:ti or femur:ti or spine:ti or spinal:ti or vertebra:ti or vertebral:ti or vertebrae:ti or lumbar:ti or shoulder*:ti or glenohumeral:ti or humeroscapular:ti or humeral:ti or radius:ti or wrist:ti or fragil*:ti osteoporosis:ti or osteoporo*:ti) AND fractur*:ti)

#2: 'fragility fracture'/exp

#3: 'low energy fracture'/exp

#4: 'low trauma fracture'/exp

#5: 'low energy trauma'/exp

#6:

“fragility fracture”:ti OR “fragility fractures”:ti OR “low energy fracture”:ti OR “low energy fractures”:ti OR “low-energy fracture”:ti OR “low-energy fractures”:ti OR “low trauma fracture”:ti OR “low trauma fractures”:ti OR “low-trauma fracture”:ti OR “low-trauma fractures”:ti OR “low energy trauma”:ti OR “low-energy trauma”:ti OR “low level trauma”:ti OR “low-level trauma”:ti OR “minor trauma fracture”:ti OR “minor trauma fractures”:ti OR “minor-trauma fracture”:ti OR “minor-trauma fractures”:ti OR “minor fracture”:ti OR “minor fractures”:ti OR “minor-fracture”:ti OR “minor-fractures”:ti OR “osteoporotic fracture”:ti OR “osteoporotic fractures”:ti

#7: #1 OR #2 OR #3 OR #4 OR #5 OR #6

#8

((familial:ti,ab or inherit*:ti,ab or heredit*:ti,ab or predispos*:ti,ab or susceptib*:ti,ab OR family:ti,ab or maternal:ti,ab or parental:ti,ab) AND histor*:ti,ab)

#9: 'genetic predisposition'

#10: #8 OR #9

#11: #7 AND #10

#12: #11 AND [embase]/lim NOT ([embase]/lim AND [medline]/lim)

COCHRANE

Up to 8 February 2021

#1:

((wrist*:ti or colles:ti or radius:ti or “articulatio radiocarpea”:ti or carpus:ti or carpal:ti or radiocarp*:ti or radial:ti or forearm*:ti or humerus:ti or metacarp*:ti or barton:ti or monteggi*:ti or ulna:ti or ulnar:ti or limb*:ti or hip:ti or hips:ti or trochanteric:ti or intertrochanteric:ti or subtrochanteric:ti or femoral:ti or femur:ti or spine:ti or spinal:ti or vertebra:ti or vertebral:ti or vertebrae:ti or lumbar:ti or shoulder*:ti or glenohumeral:ti or humeroscapular:ti or humeral:ti or radius:ti or wrist:ti or fragil*:ti osteoporosis:ti or osteoporo*:ti) AND fractur*:ti)

#2: MeSH descriptor: [Osteoporotic Fractures] explode all trees

#3: MeSH descriptor: [Fractures, Spontaneous] explode all trees

#4:

(fragility fracture):ti OR (fragility fractures):ti OR (low energy fracture):ti OR (low energy fractures):ti OR (low-energy fracture):ti OR (low-energy fractures):ti OR (low trauma fracture):ti OR (low trauma fractures):ti OR (low-trauma fracture):ti OR (low-trauma fractures):ti OR (low energy

trauma):ti OR (low-energy trauma):ti OR (low level trauma):ti OR (low-level trauma):ti OR (minor trauma fracture):ti OR (minor trauma fractures):ti OR (minor-trauma fracture):ti OR (minor-trauma fractures):ti OR (minor fracture):ti OR (minor fractures):ti OR (minor-fracture):ti OR (minor-fractures):ti OR (osteoporotic fracture):ti OR (osteoporotic fractures):ti OR (pathologic fracture):ti OR (pathological fractures):ti

#5: #1 OR #2 OR #3 OR #4

#6:

((familial:ti,ab or inherit*:ti,ab or heredit*:ti,ab or predispos*:ti,ab or susceptib*:ti,ab OR family:ti,ab or maternal:ti,ab or parental:ti,ab) AND histor*:ti,ab)

#7: MeSH descriptor: [Genetic Predisposition to Disease] explode all trees

#8: #6 OR #7

#9: #5 AND #8

AGE

MEDLINE

Up to 10 February 2021

#1:

((wrist*[ti] or colles[ti] or radius[ti] or "articulatio radiocarpea" [ti] or carpus[ti] or carpal[ti] or radiocarp*[ti] or radial[ti] or forearm*[ti] or humerus[ti] or metacarp*[ti] or barton[ti] or monteggi*[ti] or ulna[ti] or ulnar[ti] or limb*[ti] or hip[ti] or hips[ti] or trochanteric[ti] or intertrochanteric[ti] or subtrochanteric[ti] or femoral[ti] or femur[ti] or spine[ti] or spinal[ti] or vertebra[ti] or vertebral[ti] or vertebrae[ti] or lumbar[ti] or shoulder*[ti] or glenohumeral[ti] or humeroscapular[ti] or humeral[ti] or radius[ti] or wrist[ti] or fragil*[ti] osteoporosis[ti] or osteoporo*[ti] OR torax[ti] OR chest[ti] OR clavicle[ti] OR rib[ti] OR pelvis[ti] OR tibia[ti] OR fragil*[ti] OR any[ti] OR ankle[ti]) AND fractur*[ti])

#2:

"fragility fracture"[tiab] OR "fragility fractures"[tiab] OR "low energy fracture"[tiab] OR "low energy fractures"[tiab] OR "low-energy fracture"[tiab] OR "low-energy fractures"[tiab] OR "low trauma fracture"[tiab] OR "low trauma fractures"[tiab] OR "low-trauma fracture"[tiab] OR "low-trauma fractures"[tiab] OR "low energy trauma"[tiab] OR "low-energy trauma"[tiab] OR "low level trauma"[tiab] OR "low-level trauma"[tiab] OR "minor trauma fracture"[tiab] OR "minor trauma fractures"[tiab] OR "minor-trauma fracture"[tiab] OR "minor-trauma fractures"[tiab] OR "minor

fracture”[tiab] OR “minor fractures”[tiab] OR “minor-fracture”[tiab] OR “minor-fractures”[tiab] OR “osteoporotic fracture”[tiab] OR “osteoporotic fractures”[tiab] OR osteopor*[tiab]

#3: #1 AND #2

#4: age[tiab] OR year*[tiab]

#5: (#3 AND #4) AND limit: Humans

EMBASE

Up to 10 February 2021

#1:

((wrist*:ti or colles:ti or radius:ti or “articulatio radiocarpea”:ti or carpus:ti or carpal:ti or radiocarp*:ti or radial:ti or forearm*:ti or humerus:ti or metacarp*:ti or barton:ti or monteggi*:ti or ulna:ti or ulnar:ti or limb*:ti or hip:ti or hips:ti or trochanteric:ti or intertrochanteric:ti or subtrochanteric:ti or femoral:ti or femur:ti or spine:ti or spinal:ti or vertebra:ti or vertebral:ti or vertebrae:ti or lumbar:ti or shoulder*:ti or glenohumeral:ti or humeroscapular:ti or humeral:ti or radius:ti or wrist:ti or fragil*:ti osteoporosis:ti or osteoporo*:ti OR torax:ti OR chest:ti OR clavicle:ti OR rib:ti OR pelvis:ti OR tibia:ti OR fragil*:ti OR any:ti OR ankle:ti) AND fractur*:ti)

#2:

“fragility fracture”:ti,ab OR “fragility fractures”:ti,ab OR “low energy fracture”:ti,ab OR “low energy fractures”:ti,ab OR “low-energy fracture”:ti,ab OR “low-energy fractures”:ti,ab OR “low trauma fracture”:ti,ab OR “low trauma fractures”:ti,ab OR “low-trauma fracture”:ti,ab OR “low-trauma fractures”:ti,ab OR “low energy trauma”:ti,ab OR “low-energy trauma”:ti,ab OR “low level trauma”:ti,ab OR “low-level trauma”:ti,ab OR “minor trauma fracture”:ti,ab OR “minor trauma fractures”:ti,ab OR “minor-trauma fracture”:ti,ab OR “minor-trauma fractures”:ti,ab OR “minor fracture”:ti,ab OR “minor fractures”:ti,ab OR “minor-fracture”:ti,ab OR “minor-fractures”:ti,ab OR “osteoporotic fracture”:ti,ab OR “osteoporotic fractures”:ti,ab OR osteopor*:ti,ab

#3: #1 AND #2

#4: age:ti,ab OR year*:ti,ab

#5: #3 AND #4

#6: #5 AND [embase]/lim NOT ([embase]/lim AND [medline]/lim)

COCHRANE

Up to 10 February 2021

#1:

((wrist*:ti or colles:ti or radius:ti or “articulatio radiocarpea”:ti or carpus:ti or carpal:ti or radiocarp*:ti or radial:ti or forearm*:ti or humerus:ti or metacarp*:ti or barton:ti or monteggi*:ti or ulna:ti or ulnar:ti or limb*:ti or hip:ti or hips:ti or trochanteric:ti or intertrochanteric:ti or subtrochanteric:ti or femoral:ti or femur:ti or spine:ti or spinal:ti or vertebra:ti or vertebral:ti or vertebrae:ti or lumbar:ti or shoulder*:ti or glenohumeral:ti or humeroscapular:ti or humeral:ti or radius:ti or wrist:ti or fragil*:ti osteoporosis:ti or osteoporo*:ti OR torax:ti OR chest:ti OR clavicle:ti OR rib:ti OR pelvis:ti OR tibia:ti OR fragil*:ti OR any:ti OR ankle:ti) AND fractur*:ti)

#2:

(“fragility fracture”:ti,ab OR “fragility fractures”:ti,ab OR “low energy fracture”:ti,ab OR “low energy fractures”:ti,ab OR “low-energy fracture”:ti,ab OR “low-energy fractures”:ti,ab OR “low trauma fracture”:ti,ab OR “low trauma fractures”:ti,ab OR “low-trauma fracture”:ti,ab OR “low-trauma fractures”:ti,ab OR “low energy trauma”:ti,ab OR “low-energy trauma”:ti,ab OR “low level trauma”:ti,ab OR “low-level trauma”:ti,ab OR “minor trauma fracture”:ti,ab OR “minor trauma fractures”:ti,ab OR “minor-trauma fracture”:ti,ab OR “minor-trauma fractures”:ti,ab OR “minor fracture”:ti,ab OR “minor fractures”:ti,ab OR “minor-fracture”:ti,ab OR “minor-fractures”:ti,ab OR “osteoporotic fracture”:ti,ab OR “osteoporotic fractures”:ti,ab OR osteopor*:ti,ab)

#3: #1 AND #2

#4: age:ti,ab OR year*:ti,ab

#5: #3 AND #4

REMAINING FACTORS NOT CONSIDERED BY LG NICE

MEDLINE

Up to 25 February 2021

#1:

“fragility fracture”[tiab] OR “fragility fractures”[tiab] OR “low energy fracture”[tiab] OR “low energy fractures”[tiab] OR “low-energy fracture”[tiab] OR “low-energy fractures”[tiab] OR “low trauma fracture”[tiab] OR “low trauma fractures”[tiab] OR “low-trauma fracture”[tiab] OR “low-trauma fractures”[tiab] OR “low energy trauma”[tiab] OR “low-energy trauma”[tiab] OR “low level trauma”[tiab] OR “low-level trauma”[tiab] OR “minor trauma fracture”[tiab] OR “minor trauma

fractures”[tiab] OR “minor-trauma fracture”[tiab] OR “minor-trauma fractures”[tiab] OR “minor fracture”[tiab] OR “minor fractures”[tiab] OR “minor-fracture”[tiab] OR “minor-fractures”[tiab] OR “osteoporotic fracture”[tiab] OR “osteoporotic fractures”[tiab] OR (osteoporo*[tiab] AND fractur*[tiab])

#2:

menopaus*[tiab] OR antiandrogen*[tiab] OR “Gonadotropin-Releasing Hormone”[tiab] OR “Gonadotropin Releasing Hormone”[tiab] OR GnRH[tiab] OR Buserelin[tiab] OR Goserelin[tiab] OR Leuprolide[tiab] OR Nafarelin[tiab] OR “Triptorelin Pamoate”[tiab] OR “Triptorelin-Pamoate”[tiab] OR antiandrogen*[tiab] OR anti-androgen*[tiab] OR “androgen antagonist”[tiab] OR “androgen antagonists”[tiab] OR “androgen receptor antagonist”[tiab] OR “androgen receptor antagonists”[tiab] OR “androgen receptor blocker”[tiab] OR “androgen receptor blockers”[tiab] OR “nonsteroidal anti-androgens”[tiab] OR “nonsteroidal antiandrogens”[tiab] OR “nonsteroidal anti-androgen”[tiab] OR “nonsteroidal antiandrogen”[tiab] OR “non-steroidal anti-androgens”[tiab] OR “non-steroidal antiandrogens”[tiab] OR “non-steroidal anti-androgen”[tiab] OR “non-steroidal antiandrogen”[tiab] OR “lupus erythematosus”[tiab] OR lupus[tiab] OR “lupus nephritis”[tiab] OR “lupus vasculitis”[tiab] OR “physical disability”[tiab] OR “physical disabilities”[tiab] OR “physical impairment”[tiab] OR “physical impairments”[tiab] OR “cerebral palsy”[tiab] OR “cerebral-palsy”[tiab] OR spastic*[tiab] OR “spinal cord injury”[tiab] OR “spinal cord injuries”[tiab] OR parapleg*[tiab] OR (parkinson*[tiab] AND (“vertebral fracture”[tiab] OR “vertebral fractures”[tiab])) OR ("Dementia"[Mesh] OR "Alzheimer Disease"[Mesh] OR dement*[tiab] OR Alzheimer*[tiab] OR AD*[tiab] OR “mild cognitive impairment”[tiab] OR "mild cognitive impairments" [tiab] OR MCI*[tiab] OR "mild neurocognitive disorder"[tiab] OR "mild neurocognitive disorders"[tiab] OR "Cognition Disorders"[Mesh] OR "Cognitive Dysfunction"[Mesh] OR “peripheral vascular disease”[tiab] OR “peripheral vascular diseases”[tiab] OR "Peripheral Vascular Diseases"[Mesh] OR “peripheral vascular disorder”[tiab] OR “peripheral vascular disorders”[tiab] OR “peripheral vascular malformation”[tiab] OR “peripheral vascular malformations”[tiab] OR “peripheral vascular anomaly”[tiab] OR “peripheral vascular anomalies”[tiab] OR “peripheral vascular abnormality”[tiab] OR “peripheral vascular abnormalities”[tiab] OR “peripheral vascular dysfunction”[tiab] OR “peripheral vascular dysfunctions”[tiab] OR PVD*[tiab] OR “peripheric vascular disease”[tiab] OR “peripheric vascular diseases”[tiab] OR "Peripheric Vascular Diseases"[Mesh] OR “peripheric vascular disorder”[tiab] OR “peripheric vascular disorders”[tiab] OR “peripheric vascular malformation”[tiab] OR “peripheric vascular malformations”[tiab] OR “peripheric vascular anomaly”[tiab] OR “peripheric vascular anomalies”[tiab] OR “peripheric vascular abnormality”[tiab] OR “peripheric vascular

abnormalities”[tiab] OR “peripheric vascular dysfunction”[tiab] OR “peripheric vascular dysfunctions”[tiab] OR “chronic renal failure”[tiab] OR “chronic kidney disease”[tiab] OR “chronic renal failures”[tiab] OR “chronic kidney diseases”[tiab] OR "Kidney Failure, Chronic"[Mesh] OR “chronic renal insufficiency”[tiab] OR “chronic renal insufficiencies”[tiab] OR ESRD*[tiab] “end stage renal”[tiab] OR “end-stage renal”[tiab] OR CRF*[tiab] OR CRD*[tiab] OR CRI*[tiab] OR CKF*[tiab] OR CKD*[tiab] OR CKI*[tiab])

#3: diabet*[tiab] OR “diabetes mellitus”[tiab] OR “diabetes”[tiab]

#4: #3 Filters: from 2019/1/1 - 3000/12/12

#5:

HIV[tiab] OR AIDS[tiab] OR “Acquired Immunodeficiency Syndrome”[tiab] OR “Human immunodeficiency virus”[tiab]

#6: #5 Filters: from 2019/8/1 - 3000/12/12

#7:

parkinson*[tiab] AND (“hip fracture”[tiab] OR “hip fractures”[tiab] OR “non vertebral fracture”[tiab] OR “non vertebral fractures”[tiab] OR “non-vertebral fracture”[tiab] OR “non-vertebral fractures”[tiab])

#8: #7 Filters: from 2019/3/1 - 3000/12/12

#9: “systemic sclerosis”[tiab] OR scleroderma[tiab]

#10: #9 Filters: from 2018/1/1 - 3000/12/12

#11:

COPD[tiab] OR “chronic obstructive pulmonary disease”[tiab] OR “chronic obstructive pulmonary diseases”[tiab]

#12: #11 Filters: from 2017/10/1 - 3000/12/12

#13: “Rheumatoid arthritis”[tiab]

#14: #13 Filters: from 2017/9/1 - 3000/12/12

#15:

“Inflammatory Bowel Diseases”[tiab] OR “Inflammatory Bowel Disease”[tiab] OR “Crohn's Disease"[tiab] OR “Crohn Disease"[tiab] OR “Crohn's Diseases"[tiab] OR “Crohn Diseases"[tiab]

#16: #15 Filters: from 2017/2/1 - 3000/12/12

#17:

“Psoriatic arthritis”[tiab] OR “Psoriatic-arthritis”[tiab] OR Spondylarthropath*[tiab] OR “connective tissue disease”[tiab] OR “connective tissue diseases”[tiab] OR “skin disease”[tiab] OR “skin diseases”[tiab] OR "Connective Tissue Diseases"[Mesh]

#18: #17 Filters: from 2016/1/1 - 3000/12/12

#19:

LnRH[tiab] OR “luteinizing hormone-releasing hormone”[tiab] OR “luteinizing hormone releasing hormone”[tiab] OR “aromatase inhibitors”[tiab] OR “aromatase inhibitor”[tiab] OR tamoxifen[tiab] OR “Adjuvant hormonal therapy” ((adjuvant[tiab] OR hormonal[tiab]) AND (cancer[tiab] OR tumor[tiab] OR malignan*[tiab] OR neoplasm*[tiab]))

#20: #19 Filters: from 2015/5/1 - 3000/12/12

#21: “multiple sclerosis”[tiab]

#22: #21 Filters: from 2012/11/1 - 3000/12/12

#23:

gender[tiab] OR sex[tiab] OR (men[tiab] AND women[tiab]) OR (male[tiab] AND female[tiab])

#24: #23 Filters: from 2002/9/1 - 3000/12/12

#25:

#2 OR #4 OR #6 OR #8 OR #10 OR #12 OR #14 OR #16 OR #18 OR #20 OR #22 OR #24

#26: #1 AND #25

EMBASE

Up to 25 February 2021

#1:

“fragility fracture”:ti,ab OR “fragility fractures”:ti,ab OR “low energy fracture”:ti,ab OR “low energy fractures”:ti,ab OR “low-energy fracture”:ti,ab OR “low-energy fractures”:ti,ab OR “low trauma fracture”:ti,ab OR “low trauma fractures”:ti,ab OR “low-trauma fracture”:ti,ab OR “low-trauma fractures”:ti,ab OR “low energy trauma”:ti,ab OR “low-energy trauma”:ti,ab OR “low level trauma”:ti,ab OR “low-level trauma”:ti,ab OR “minor trauma fracture”:ti,ab OR “minor trauma fractures”:ti,ab OR “minor-trauma fracture”:ti,ab OR “minor-trauma fractures”:ti,ab OR “minor fracture”:ti,ab OR “minor fractures”:ti,ab OR “minor-fracture”:ti,ab OR “minor-fractures”:ti,ab OR “osteoporotic fracture”:ti,ab OR “osteoporotic fractures”:ti,ab OR (osteoporo*:ti,ab AND fractur*:ti,ab)

#2:

menopaus*:ti,ab OR antiandrogen*:ti,ab OR “Gonadotropin-Releasing Hormone”:ti,ab OR “Gonadotropin Releasing Hormone”:ti,ab OR GnRH:ti,ab OR Buserelin:ti,ab OR Goserelin:ti,ab OR Leuprolide:ti,ab OR Nafarelin:ti,ab OR “Triptorelin Pamoate”:ti,ab OR “Triptorelin-Pamoate”:ti,ab OR antiandrogen*:ti,ab OR anti-androgen*:ti,ab OR “androgen antagonist”:ti,ab OR “androgen antagonists”:ti,ab OR “androgen receptor antagonist”:ti,ab OR “androgen receptor antagonists”:ti,ab

OR “androgen receptor blocker”:ti,ab OR “androgen receptor blockers”:ti,ab OR “nonsteroidal anti-androgens”:ti,ab OR “nonsteroidal antiandrogens”:ti,ab OR “nonsteroidal anti-androgen”:ti,ab OR “nonsteroidal antiandrogen”:ti,ab OR “non-steroidal anti-androgens”:ti,ab OR “non-steroidal antiandrogens”:ti,ab OR “non-steroidal anti-androgen”:ti,ab OR “non-steroidal antiandrogen”:ti,ab OR “lupus erythematosus”:ti,ab OR lupus:ti,ab OR “lupus nephritis”:ti,ab OR “lupus vasculitis”:ti,ab OR “physical disability”:ti,ab OR “physical disabilities”:ti,ab OR “physical impairment”:ti,ab OR “physical impairments”:ti,ab OR “cerebral palsy”:ti,ab OR “cerebral-palsy”:ti,ab OR spastic*:ti,ab OR “spinal cord injury”:ti,ab OR “spinal cord injuries”:ti,ab OR parapleg*:ti,ab OR (parkinson*:ti,ab AND (“vertebral fracture”:ti,ab OR “vertebral fractures”:ti,ab)) OR (“dementia” OR "Alzheimer Disease" OR "Cognitive defect" OR "Peripheral Vascular Diseases" OR "chronic kidney failure" OR dement*:ti,ab OR Alzheimer*:ti,ab OR AD*:ti,ab OR “mild cognitive impairment”:ti,ab OR "mild cognitive impairments":ti,ab OR MCI*:ti,ab OR "mild neurocognitive disorder":ti,ab OR "mild neurocognitive disorders":ti,ab OR “peripheral vascular disease”:ti,ab OR “peripheral vascular diseases”:ti,ab OR “peripheral vascular disorder”:ti,ab OR “peripheral vascular disorders”:ti,ab OR “peripheral vascular malformation”:ti,ab OR “peripheral vascular malformations”:ti,ab OR “peripheral vascular anomaly”:ti,ab OR “peripheral vascular anomalies”:ti,ab OR “peripheral vascular abnormality”:ti,ab OR “peripheral vascular abnormalities”:ti,ab OR “peripheral vascular dysfunction”:ti,ab OR “peripheral vascular dysfunctions”:ti,ab OR PVD*:ti,ab OR “peripheric vascular disease”:ti,ab OR “peripheric vascular diseases”:ti,ab OR “peripheric vascular disorder”:ti,ab OR “peripheric vascular disorders”:ti,ab OR “peripheric vascular malformation”:ti,ab OR “peripheric vascular malformations”:ti,ab OR “peripheric vascular anomaly”:ti,ab OR “peripheric vascular anomalies”:ti,ab OR “peripheric vascular abnormality”:ti,ab OR “peripheric vascular abnormalities”:ti,ab OR “peripheric vascular dysfunction”:ti,ab OR “peripheric vascular dysfunctions”:ti,ab OR “chronic renal failure”:ti,ab OR “chronic kidney disease”:ti,ab OR “chronic renal failures”:ti,ab OR “chronic kidney diseases”:ti,ab OR “chronic renal insufficiency”:ti,ab OR “chronic renal insufficiencies”:ti,ab OR ESRD*:ti,ab “end stage renal”:ti,ab OR “end-stage renal”:ti,ab OR CRF*:ti,ab OR CRD*:ti,ab OR CRI*:ti,ab OR CKF*:ti,ab OR CKD*:ti,ab OR CKI*:ti,ab)

#3: diabet*:ti,ab OR “diabetes mellitus”:ti,ab OR “diabetes”:ti,ab

#4: #3 Filters: from 2019/1/1 - 3000/12/12

#5:

HIV:ti,ab OR AIDS:ti,ab OR “Acquired Immunodeficiency Syndrome”:ti,ab OR “Human immunodeficiency virus”:ti,ab

#6: #5 Filters: from 2019/8/1 - 3000/12/12

#7:

parkinson*:ti,ab AND (“hip fracture”:ti,ab OR “hip fractures”:ti,ab OR “non vertebral fracture”:ti,ab OR “non vertebral fractures”:ti,ab OR “non-vertebral fracture”:ti,ab OR “non-vertebral fractures”:ti,ab)

#8: #7 Filters: from 2019/3/1 - 3000/12/12

#9: “systemic sclerosis”:ti,ab OR scleroderma:ti,ab

#10: #9 Filters: from 2018/1/1 - 3000/12/12

#11:

COPD:ti,ab OR “chronic obstructive pulmonary disease”:ti,ab OR “chronic obstructive pulmonary diseases”:ti,ab

#12: #11 Filters: from 2017/10/1 - 3000/12/12

#13: “Rheumatoid arthritis”:ti,ab

#14: #13 Filters: from 2017/9/1 - 3000/12/12

#15:

“Inflammatory Bowel Diseases”:ti,ab OR “Inflammatory Bowel Disease”:ti,ab OR “Crohn's Disease”:ti,ab OR “Crohn Disease”:ti,ab OR “Crohn's Diseases”:ti,ab OR “Crohn Diseases”:ti,ab

#16: #15 Filters: from 2017/2/1 - 3000/12/12

#17:

“Psoriatic arthritis”:ti,ab OR “Psoriatic-arthritis”:ti,ab OR Spondylarthropath*:ti,ab OR “connective tissue disease”:ti,ab OR “connective tissue diseases”:ti,ab OR “skin disease”:ti,ab OR “skin diseases”:ti,ab

#18: #17 Filters: from 2016/1/1 - 3000/12/12

#19:

LnRH:ti,ab OR “luteinizing hormone-releasing hormone”:ti,ab OR “luteinizing hormone releasing hormone”:ti,ab OR “aromatase inhibitors”:ti,ab OR “aromatase inhibitor”:ti,ab OR tamoxifen:ti,ab OR “Adjuvant hormonal therapy” ((adjuvant:ti,ab OR hormonal:ti,ab) AND (cancer:ti,ab OR tumor:ti,ab OR malignan*:ti,ab OR neoplasm*:ti,ab))

#20: #19 Filters: from 2015/5/1 - 3000/12/12

#21: “multiple sclerosis”:ti,ab

#22: #21 Filters: from 2012/11/1 - 3000/12/12

#23:

gender:ti,ab OR sex:ti,ab OR (men:ti,ab AND women:ti,ab) OR (male:ti,ab AND female:ti,ab)

#24: #23 Filters: from 2002/9/1 - 3000/12/12

#25:

#2 OR #4 OR #6 OR #8 OR #10 OR #12 OR #14 OR #16 OR #18 OR #20 OR #22 OR #24

#26: #1 AND #25

COCHRANE

Up to 25 February 2021

#1:

“fragility fracture”:ti,ab OR “fragility fractures”:ti,ab OR “low energy fracture”:ti,ab OR “low energy fractures”:ti,ab OR “low-energy fracture”:ti,ab OR “low-energy fractures”:ti,ab OR “low trauma fracture”:ti,ab OR “low trauma fractures”:ti,ab OR “low-trauma fracture”:ti,ab OR “low-trauma fractures”:ti,ab OR “low energy trauma”:ti,ab OR “low-energy trauma”:ti,ab OR “low level trauma”:ti,ab OR “low-level trauma”:ti,ab OR “minor trauma fracture”:ti,ab OR “minor trauma fractures”:ti,ab OR “minor-trauma fracture”:ti,ab OR “minor-trauma fractures”:ti,ab OR “minor fracture”:ti,ab OR “minor fractures”:ti,ab OR “minor-fracture”:ti,ab OR “minor-fractures”:ti,ab OR “osteoporotic fracture”:ti,ab OR “osteoporotic fractures”:ti,ab OR (osteoporo*:ti,ab AND fractur*:ti,ab)

#2:

menopaus*:ti,ab OR antiandrogen*:ti,ab OR “Gonadotropin-Releasing Hormone”:ti,ab OR “Gonadotropin Releasing Hormone”:ti,ab OR GnRH:ti,ab OR Buserelin:ti,ab OR Goserelin:ti,ab OR Leuprolide:ti,ab OR Nafarelin:ti,ab OR “Triptorelin Pamoate”:ti,ab OR “Triptorelin-Pamoate”:ti,ab OR antiandrogen*:ti,ab OR anti-androgen*:ti,ab OR “androgen antagonist”:ti,ab OR “androgen antagonists”:ti,ab OR “androgen receptor antagonist”:ti,ab OR “androgen receptor antagonists”:ti,ab OR “androgen receptor blocker”:ti,ab OR “androgen receptor blockers”:ti,ab OR “nonsteroidal anti-androgens”:ti,ab OR “nonsteroidal antiandrogens”:ti,ab OR “nonsteroidal anti-androgen”:ti,ab OR “nonsteroidal antiandrogen”:ti,ab OR “non-steroidal anti-androgens”:ti,ab OR “non-steroidal antiandrogens”:ti,ab OR “non-steroidal anti-androgen”:ti,ab OR “non-steroidal antiandrogen”:ti,ab OR “lupus erythematosus”:ti,ab OR lupus:ti,ab OR “lupus nephritis”:ti,ab OR “lupus vasculitis”:ti,ab OR “physical disability”:ti,ab OR “physical disabilities”:ti,ab OR “physical impairment”:ti,ab OR “physical impairments”:ti,ab OR “cerebral palsy”:ti,ab OR “cerebral-palsy”:ti,ab OR spastic*:ti,ab OR “spinal cord injury”:ti,ab OR “spinal cord injuries”:ti,ab OR parapleg*:ti,ab OR (parkinson*:ti,ab AND (“vertebral fracture”:ti,ab OR “vertebral fractures”:ti,ab)) OR (demented:ti,ab OR dementic:ti,ab OR dementia:ti,ab OR Alzheimer*:ti,ab OR AD*:ti,ab OR “mild cognitive impairment”:ti,ab OR "mild cognitive impairments":ti,ab OR MCI*:ti,ab OR "mild neurocognitive

disorder":ti,ab OR "mild neurocognitive disorders":ti,ab OR “peripheral vascular disease”:ti,ab OR “peripheral vascular diseases”:ti,ab OR “peripheral vascular disorder”:ti,ab OR “peripheral vascular disorders”:ti,ab OR “peripheral vascular malformation”:ti,ab OR “peripheral vascular malformations”:ti,ab OR “peripheral vascular anomaly”:ti,ab OR “peripheral vascular anomalies”:ti,ab OR “peripheral vascular abnormality”:ti,ab OR “peripheral vascular abnormalities”:ti,ab OR “peripheral vascular dysfunction”:ti,ab OR “peripheral vascular dysfunctions”:ti,ab OR PVD*:ti,ab OR “peripheric vascular disease”:ti,ab OR “peripheric vascular diseases”:ti,ab OR “peripheric vascular disorder”:ti,ab OR “peripheric vascular disorders”:ti,ab OR “peripheric vascular malformation”:ti,ab OR “peripheric vascular malformations”:ti,ab OR “peripheric vascular anomaly”:ti,ab OR “peripheric vascular anomalies”:ti,ab OR “peripheric vascular abnormality”:ti,ab OR “peripheric vascular abnormalities”:ti,ab OR “peripheric vascular dysfunction”:ti,ab OR “peripheric vascular dysfunctions”:ti,ab OR “chronic renal failure”:ti,ab OR “chronic kidney disease”:ti,ab OR “chronic renal failures”:ti,ab OR “chronic kidney diseases”:ti,ab OR “chronic renal insufficiency”:ti,ab OR “chronic renal insufficiencies”:ti,ab OR ESRD*:ti,ab “end stage renal”:ti,ab OR “end-stage renal”:ti,ab OR CRF*:ti,ab OR CRD*:ti,ab OR CRI*:ti,ab OR CKF*:ti,ab OR CKD*:ti,ab OR CKI*:ti,ab OR (MeSH descriptor: [Renal Insufficiency, Chronic] explode all trees) OR (MeSH descriptor: [Peripheral Vascular Diseases] explode all trees) OR (MeSH descriptor: [Dementia] explode all trees))

#3: diabet*:ti,ab OR “diabetes mellitus”:ti,ab OR “diabetes”:ti,ab

#4: #3 Filters: from 2019/1/1 - 3000/12/12

#5:

HIV:ti,ab OR AIDS:ti,ab OR “Acquired Immunodeficiency Syndrome”:ti,ab OR “Human immunodeficiency virus”:ti,ab

#6: #5 Filters: from 2019/8/1 - 3000/12/12

#7:

parkinson*:ti,ab AND (“hip fracture”:ti,ab OR “hip fractures”:ti,ab OR “non vertebral fracture”:ti,ab OR “non vertebral fractures”:ti,ab OR “non-vertebral fracture”:ti,ab OR “non-vertebral fractures”:ti,ab)

#8: #7 Filters: from 2019/3/1 - 3000/12/12

#9: “systemic sclerosis”:ti,ab OR scleroderma:ti,ab

#10: #9 Filters: from 2018/1/1 - 3000/12/12

#11:

COPD:ti,ab OR “chronic obstructive pulmonary disease”:ti,ab OR “chronic obstructive pulmonary diseases”:ti,ab

#12: #11 Filters: from 2017/10/1 - 3000/12/12

#13: “Rheumatoid arthritis”:ti,ab

#14: #13 Filters: from 2017/9/1 - 3000/12/12

#15:

“Inflammatory Bowel Diseases”:ti,ab OR “Inflammatory Bowel Disease”:ti,ab OR “Crohn's Disease":ti,ab OR “Crohn Disease":ti,ab OR “Crohn's Diseases":ti,ab OR “Crohn Diseases":ti,ab

#16: #15 Filters: from 2017/2/1 - 3000/12/12

#17:

“Psoriatic arthritis”:ti,ab OR “Psoriatic-arthritis”:ti,ab OR Spondylarthropath*:ti,ab OR “connective tissue disease”:ti,ab OR “connective tissue diseases”:ti,ab OR “skin disease”:ti,ab OR “skin diseases”:ti,ab OR "Connective Tissue Diseases"[Mesh]

#18: #17 Filters: from 2016/1/1 - 3000/12/12

#19:

LnRH:ti,ab OR “luteinizing hormone-releasing hormone”:ti,ab OR “luteinizing hormone releasing hormone”:ti,ab OR “aromatase inhibitors”:ti,ab OR “aromatase inhibitor”:ti,ab OR tamoxifen:ti,ab OR “Adjuvant hormonal therapy” ((adjuvant:ti,ab OR hormonal:ti,ab) AND (cancer:ti,ab OR tumor:ti,ab OR malignan*:ti,ab OR neoplasm*:ti,ab))

#20: #19 Filters: from 2015/5/1 - 3000/12/12

#21: “multiple sclerosis”:ti,ab

#22: #21 Filters: from 2012/11/1 - 3000/12/12

#23:

gender:ti,ab OR sex:ti,ab OR (men:ti,ab AND women:ti,ab) OR (male:ti,ab AND female:ti,ab)

#24: #23 Filters: from 2002/9/1 - 3000/12/12

#25: #2 OR #4 OR #6 OR #8 OR #10 OR #12 OR #14 OR #16 OR #18 OR #20 OR #22 OR #24

#26: #1 AND #25

Flow chart

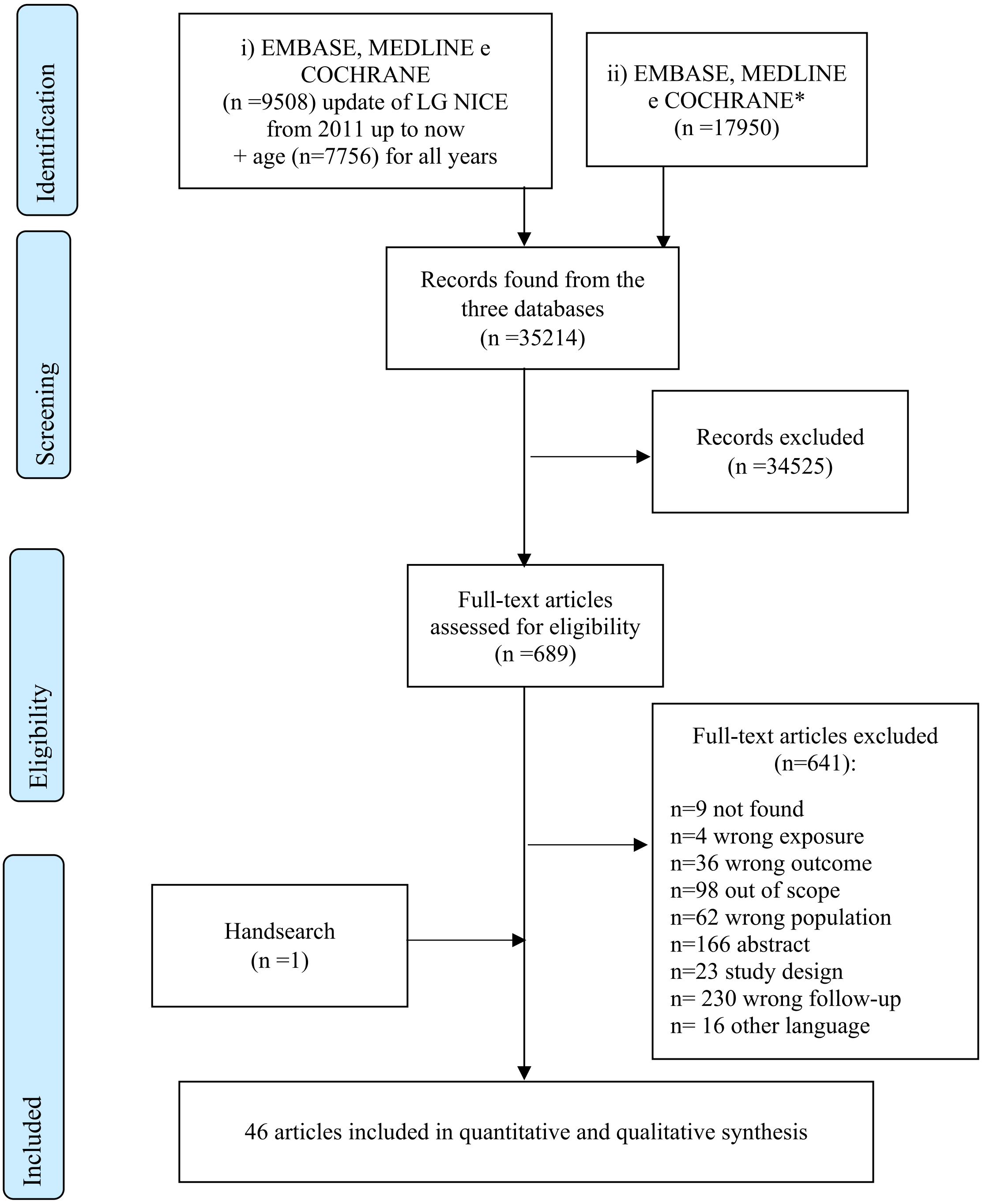
Identification
Screening
Eligibility
Included
i) EMBASE, MEDLINE e COCHRANE (n =9508) update of LG NICE from 2011 up to now + age (n=7756) for all years
ii) EMBASE, MEDLINE e COCHRANE* (n =17950)
Records found from the three databases (n =35214)
Records excluded (n =34525)
Full-text articles assessed for eligibility (n =689)
Full-text articles excluded (n=641):
n=9 not found
n=4 wrong exposure
n=36 wrong outcome
n=98 out of scope
n=62 wrong population
n=166 abstract
n=23 study design
n= 230 wrong follow-up
n= 16 other language
Handsearch (n =1)
46 articles included in quantitative and qualitative synthesis

Results

(Re)fracture among patients, fractured or not, exposed to adjuvant hormone therapy compared to unexposed

Study or Subgroup	log[Hazard Ratio]	SE	Weight	Hazard Ratio IV, Fixed, 95% CI
Reber 2018	0.3001	0.1634	100.0%	1.35 [0.98, 1.86]
**Total (95% CI)**			**100.0%**	**1.35 [0.98, 1.86]**

Heterogeneity: Not applicable
Test for overall effect: Z = 1.84 (P = 0.07)

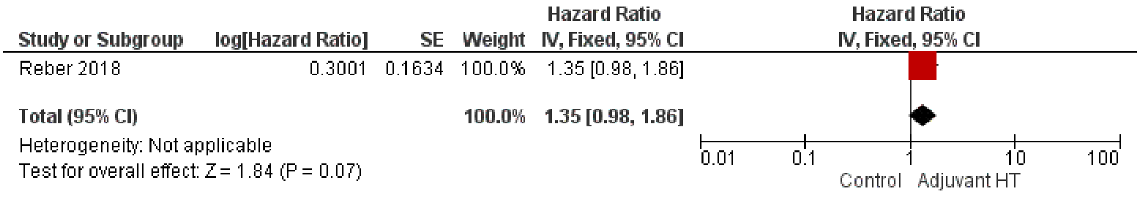

(Re)fracture among patients, fractured or not, exposed to acquired immunodeficiency syndrome compared to unexposed

Study or Subgroup	log[Odds Ratio]	SE	Weight	Odds Ratio IV, Fixed, 95% CI
Prieto-Alhambra 2014	0.1823	0.4169	100.0%	1.20 [0.53, 2.72]
**Total (95% CI)**			**100.0%**	**1.20 [0.53, 2.72]**

Heterogeneity: Not applicable
Test for overall effect: Z = 0.44 (P = 0.66)

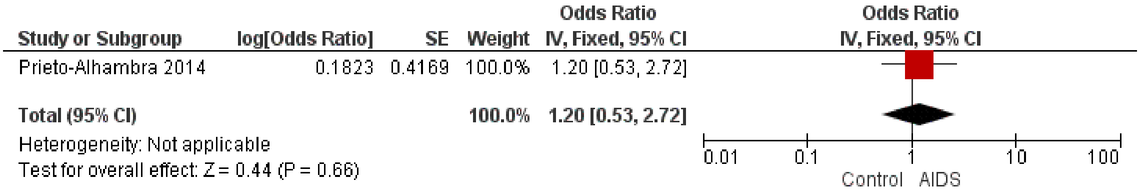

(Re)fracture among patients, fractured or not, exposed to chronic obstructive pulmonary disease compared to unexposed

Study or Subgroup	log[Risk Ratio]	SE	Weight	Risk Ratio IV, Random, 95% CI
Adas-Okuma 2020	1.8532	0.3633	2.5%	6.38 [3.13, 13.00]
Dennison 2012	0.4055	0.073	21.0%	1.50 [1.30, 1.73]
Morse 2009	0.47	0.5765	1.1%	1.60 [0.52, 4.95]
Soriano 2005	0.4574	0.0995	16.6%	1.58 [1.30, 1.92]
Vestergaard 2007	0.174	0.0264	28.7%	1.19 [1.13, 1.25]
Yusuf 2020	0.157	0.0088	30.2%	1.17 [1.15, 1.19]
**Total (95% CI)**			**100.0%**	**1.36 [1.21, 1.53]**

Heterogeneity: Tau² = 0.01; Chi² = 42.31, df = 5 (P < 0.00001); I² = 88%
Test for overall effect: Z = 5.15 (P < 0.00001)

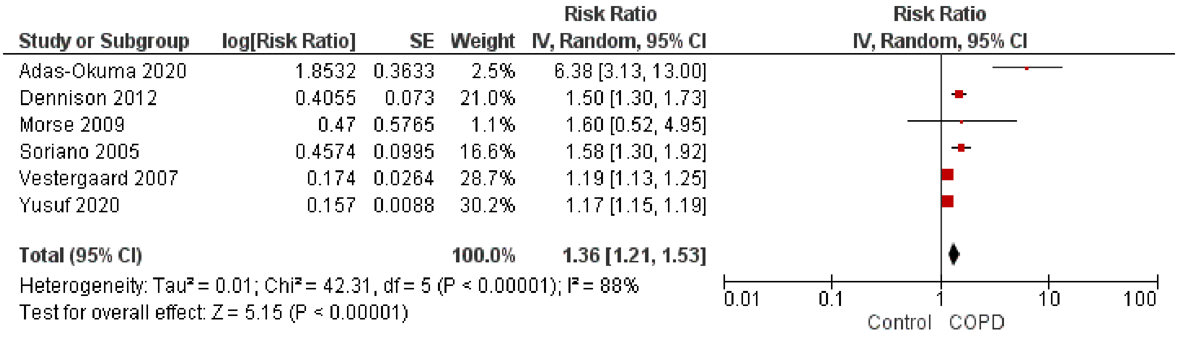

(Re)fracture among patients, fractured or not, exposed to corticosteroid compared to unexposed

Study or Subgroup	log[Risk Ratio]	SE	Weight	Risk Ratio IV, Random, 95% CI
Adas-Okuma 2020	1.6919	0.3416	1.2%	5.43 [2.78, 10.61]
Bonafede 2016	0.0488	0.0249	19.4%	1.05 [1.00, 1.10]
Cooper 1995	0.9163	0.4189	0.8%	2.50 [1.10, 5.68]
Hannan 2019	0.4547	0.4406	0.8%	1.58 [0.66, 3.74]
Prieto-Alhambra 2015	0.1083	0.0049	21.0%	1.11 [1.10, 1.13]
Reber 2018	0.2151	0.0519	15.4%	1.24 [1.12, 1.37]
Vestergaard 2007	0.392	0.014	20.5%	1.48 [1.44, 1.52]
Yusuf 2020	0.1222	0.0045	21.0%	1.13 [1.12, 1.14]
**Total (95% CI)**			**100.0%**	**1.23 [1.13, 1.32]**

Heterogeneity: Tau² = 0.01; Chi² = 411.13, df = 7 (P < 0.00001); I² = 98%
Test for overall effect: Z = 5.19 (P < 0.00001)

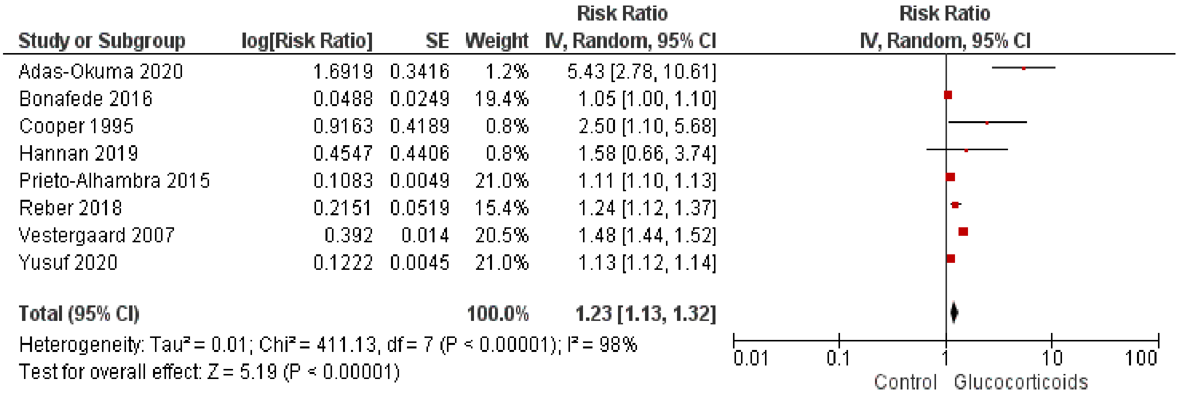

(Re)fracture among patients, fractured or not, exposed to diabetes compared to unexposed

Study or Subgroup	log[Risk Ratio]	SE	Weight	Risk Ratio IV, Random, 95% CI
Bonafede 2016	0.0953	0.0335	26.8%	1.10 [1.03, 1.17]
Hannan 2019	0.0372	0.2881	1.5%	1.04 [0.59, 1.83]
Ivers 2001	-0.3567	0.2855	1.5%	0.70 [0.40, 1.22]
Morse 2009	-0.7395	0.7125	0.3%	0.48 [0.12, 1.93]
Nickolas 2006	-0.3567	0.3837	0.9%	0.70 [0.33, 1.48]
Vestergaard 2005_type 1 diabetes	0.2624	0.0581	18.4%	1.30 [1.16, 1.46]
Vestergaard 2005_type 2 diabetes	0.174	0.0355	26.1%	1.19 [1.11, 1.28]
Vestergaard 2005_unspecified type of diabetes	0.1823	0.0585	18.2%	1.20 [1.07, 1.35]
Yu 2021	-0.0278	0.1413	5.5%	0.97 [0.74, 1.28]
Zhou 2021	0.1927	0.4133	0.8%	1.21 [0.54, 2.73]
**Total (95% CI)**			**100.0%**	**1.15 [1.07, 1.24]**

Heterogeneity: $Tau^2 = 0.00$; $Chi^2 = 15.34$, df = 9 (P = 0.08); $I^2 = 41\%$
Test for overall effect: Z = 3.93 (P < 0.0001)

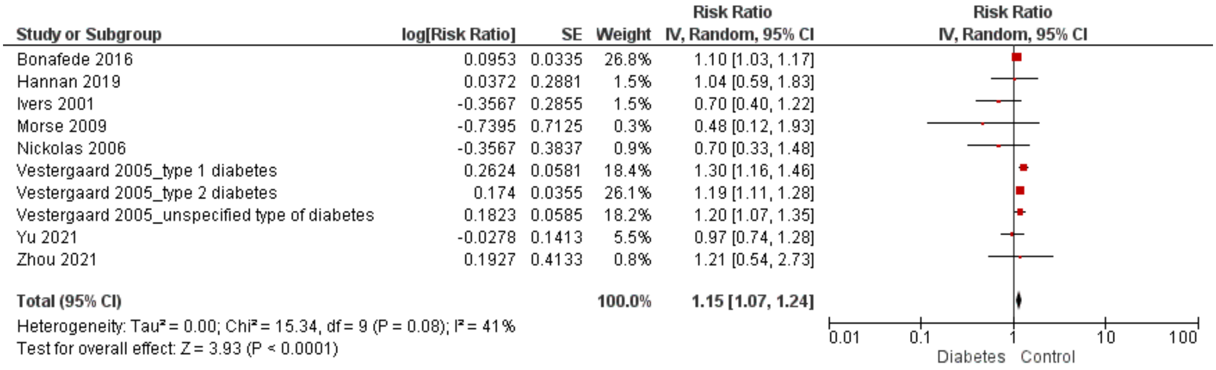

(Re)fracture among patients, fractured or not, aged more than 65 years compared to subjects aged 50-64 years

Study or Subgroup	log[Risk Ratio]	SE	Weight	Risk Ratio IV, Fixed, 95% CI
**13.3.1 65-74 years**				
Dennison 2012	0.1823	0.0444	100.0%	1.20 [1.10, 1.31]
**Subtotal (95% CI)**			**100.0%**	**1.20 [1.10, 1.31]**
Heterogeneity: Not applicable				
Test for overall effect: Z = 4.11 (P < 0.0001)				
**13.3.2 75-84 years**				
Dennison 2012	0.5878	0.0601	100.0%	1.80 [1.60, 2.03]
**Subtotal (95% CI)**			**100.0%**	**1.80 [1.60, 2.03]**
Heterogeneity: Not applicable				
Test for overall effect: Z = 9.78 (P < 0.00001)				
**13.3.3 85+ years**				
Dennison 2012	0.8329	0.0713	100.0%	2.30 [2.00, 2.64]
**Subtotal (95% CI)**			**100.0%**	**2.30 [2.00, 2.64]**
Heterogeneity: Not applicable				
Test for overall effect: Z = 11.68 (P < 0.00001)				

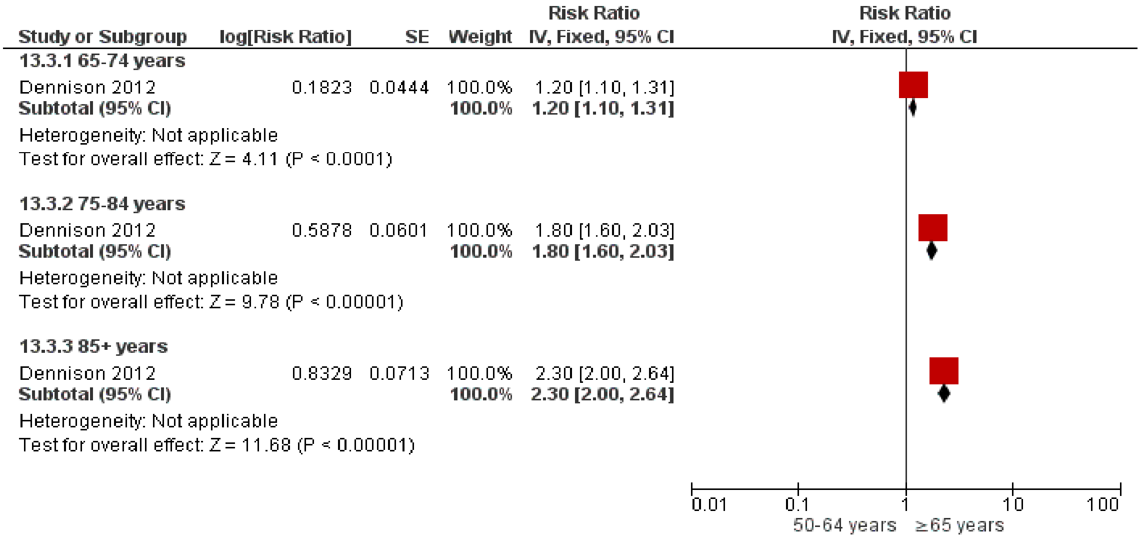

(Re)fracture among patients, fractured or not, exposed to family history of fractures compared to unexposed

Study or Subgroup	log[Risk Ratio]	SE	Weight	Risk Ratio IV, Random, 95% CI
Anpalahan 2014	1.6956	0.8119	9.7%	5.45 [1.11, 26.76]
Bonafede 2016	1.4884	0.0407	19.4%	4.43 [4.09, 4.80]
Nickolas 2006	0.6313	0.3021	17.0%	1.88 [1.04, 3.40]
Siris 2001	0.239	0.0462	19.4%	1.27 [1.16, 1.39]
Sosa 2012	0.7934	0.139	18.9%	2.21 [1.68, 2.90]
Torgerson 1996	1.4227	0.3962	15.7%	4.15 [1.91, 9.02]
**Total (95% CI)**			**100.0%**	**2.66 [1.32, 5.35]**

Heterogeneity: $Tau^2 = 0.65$; $Chi^2 = 416.27$, df = 5 (P < 0.00001); $I^2 = 99\%$
Test for overall effect: Z = 2.75 (P = 0.006)

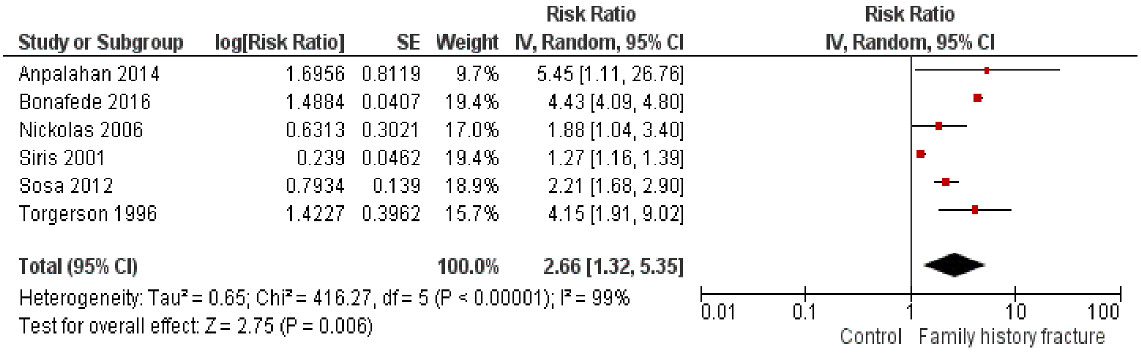

(Re)fracture among women, fractured or not, compared to men

Study or Subgroup	log[Risk Ratio]	SE	Weight	Risk Ratio IV, Random, 95% CI
Bonafede 2016	-0.5447	0.0271	9.1%	0.58 [0.55, 0.61]
Nickolas 2006	0.4637	0.2117	6.9%	1.59 [1.05, 2.41]
Reber 2018	0.7885	0.0311	9.0%	2.20 [2.07, 2.34]
Ruan 2011	0.3075	0.0152	9.1%	1.36 [1.32, 1.40]
Rudman 2019	-0.656	0.15	7.9%	0.52 [0.39, 0.70]
Sheer 2020	0.3784	0.0178	9.1%	1.46 [1.41, 1.51]
Shim 2020	0.2546	0.0285	9.0%	1.29 [1.22, 1.36]
Sosa 2012	0.0277	0.1065	8.4%	1.03 [0.83, 1.27]
Vestergaard 2007	0	0.0049	9.1%	1.00 [0.99, 1.01]
Waterloo 2012	-0.1508	0.0981	8.5%	0.86 [0.71, 1.04]
Yusuf 2020	0.6152	0.0083	9.1%	1.85 [1.82, 1.88]
Zhou 2021	0.8408	0.3571	4.8%	2.32 [1.15, 4.67]
**Total (95% CI)**			**100.0%**	**1.19 [0.95, 1.49]**

Heterogeneity: $Tau^2 = 0.14$; $Chi^2 = 5446.10$, df = 11 ($P < 0.00001$); $I^2 = 100\%$
Test for overall effect: Z = 1.53 (P = 0.13)

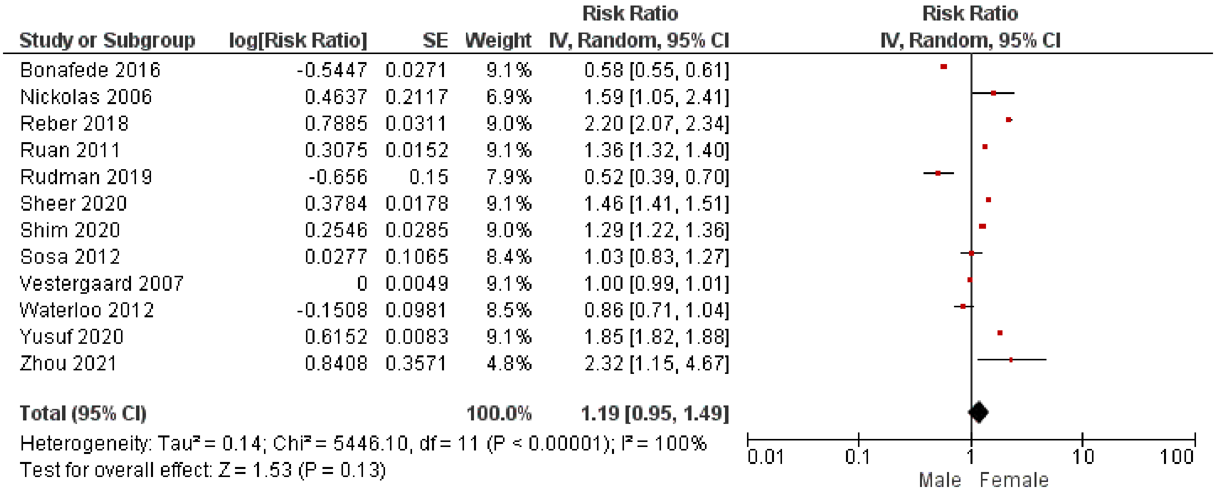

(Re)fracture among patients, fractured or not, exposed to severe physical disabilities compared to unexposed

Study or Subgroup	log[Risk Ratio]	SE	Weight	Risk Ratio IV, Random, 95% CI
Vestergaard 1998: feet/toe	0.8755	0.3128	17.6%	2.40 [1.30, 4.43]
Vestergaard 1998: finger/hand	-0.3567	0.2855	17.9%	0.70 [0.40, 1.22]
Vestergaard 1998: lower leg	1.6487	0.202	18.8%	5.20 [3.50, 7.73]
Vestergaard 1998: other	0.6931	0.6143	13.4%	2.00 [0.60, 6.67]
Vestergaard 1998: spine	1.1314	0.4056	16.4%	3.10 [1.40, 6.86]
Vestergaard 1998: upper arm	-0.3567	0.4323	16.0%	0.70 [0.30, 1.63]
**Total (95% CI)**			**100.0%**	**1.86 [0.85, 4.06]**

Heterogeneity: $Tau^2 = 0.80$; $Chi^2 = 41.84$, df = 5 ($P < 0.00001$); $I^2 = 88\%$
Test for overall effect: Z = 1.56 (P = 0.12)

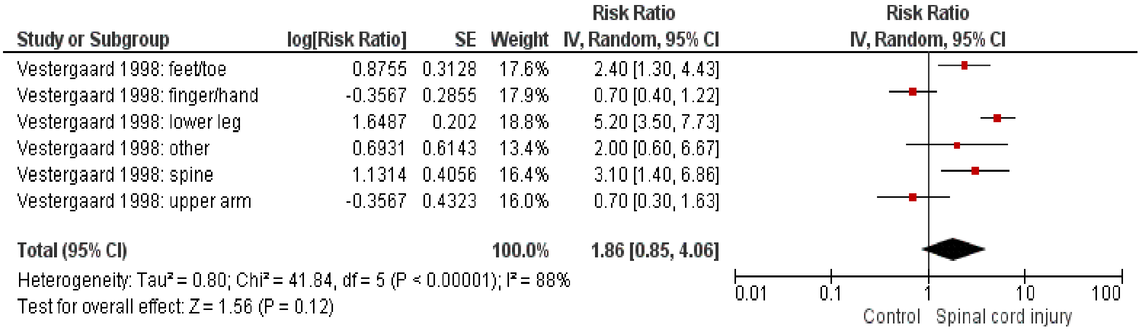

(Re)fracture among patients, fractured or not, low bone mass index to compared to normoweight subjects

Study or Subgroup	log[Odds Ratio]	SE	Weight	Odds Ratio IV, Random, 95% CI
Compston 2011	0.07257069	0.0465106	23.7%	1.08 [0.98, 1.18]
Cooper 1995	1.10866263	0.23616568	9.1%	3.03 [1.91, 4.81]
Gibson-Smith 2014	0.12783337	0.0474	23.6%	1.14 [1.04, 1.25]
Jadoul 2006	-0.03922071	0.1151	17.8%	0.96 [0.77, 1.20]
Meyer 1995	0.63487827	0.2568	8.2%	1.89 [1.14, 3.12]
Morse 2009	0.01005034	0.414	3.9%	1.01 [0.45, 2.27]
Yu 2021	0.37106368	0.1651	13.6%	1.45 [1.05, 2.00]
**Total (95% CI)**			**100.0%**	**1.28 [1.07, 1.52]**

Heterogeneity: $Tau^2 = 0.03$; $Chi^2 = 26.90$, df = 6 ($P = 0.0002$); $I^2 = 78\%$
Test for overall effect: Z = 2.74 (P = 0.006)

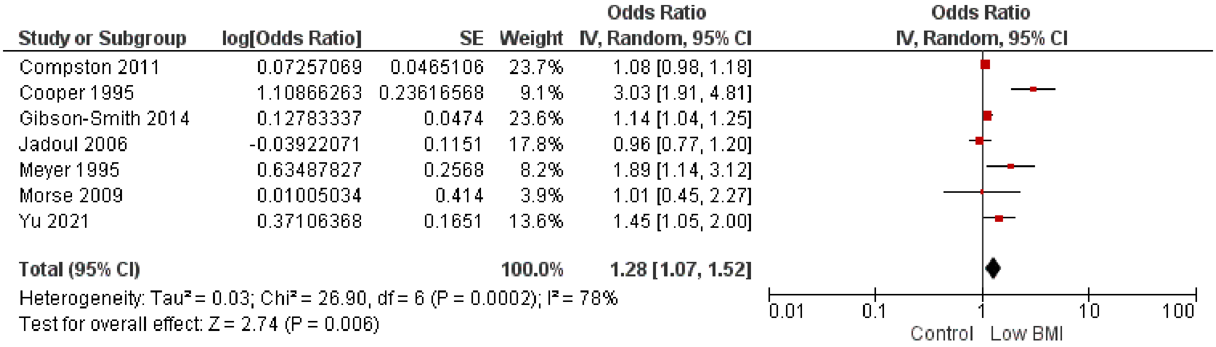

(Re)fracture among patients, fractured or not, exposed to inflammatory bowel diseases compared to unexposed

Study or Subgroup	log[Risk Ratio]	SE	Weight	Risk Ratio IV, Fixed, 95% CI
**17.1.1 Any fracture**				
Weiss 2010_ulcerative colitis	0.3365	0.123	100.0%	1.40 [1.10, 1.78]
**Subtotal (95% CI)**			**100.0%**	**1.40 [1.10, 1.78]**
Heterogeneity: Not applicable				
Test for overall effect: Z = 2.74 (P = 0.006)				
**17.1.2 Hip fracture**				
Weiss 2010_ulcerative colitis	0.2624	0.0852	100.0%	1.30 [1.10, 1.54]
**Subtotal (95% CI)**			**100.0%**	**1.30 [1.10, 1.54]**
Heterogeneity: Not applicable				
Test for overall effect: Z = 3.08 (P = 0.002)				
**17.1.3 Vertebral fracture**				
Weiss 2010_ulcerative colitis	0.5306	0.2221	100.0%	1.70 [1.10, 2.63]
**Subtotal (95% CI)**			**100.0%**	**1.70 [1.10, 2.63]**
Heterogeneity: Not applicable				
Test for overall effect: Z = 2.39 (P = 0.02)				

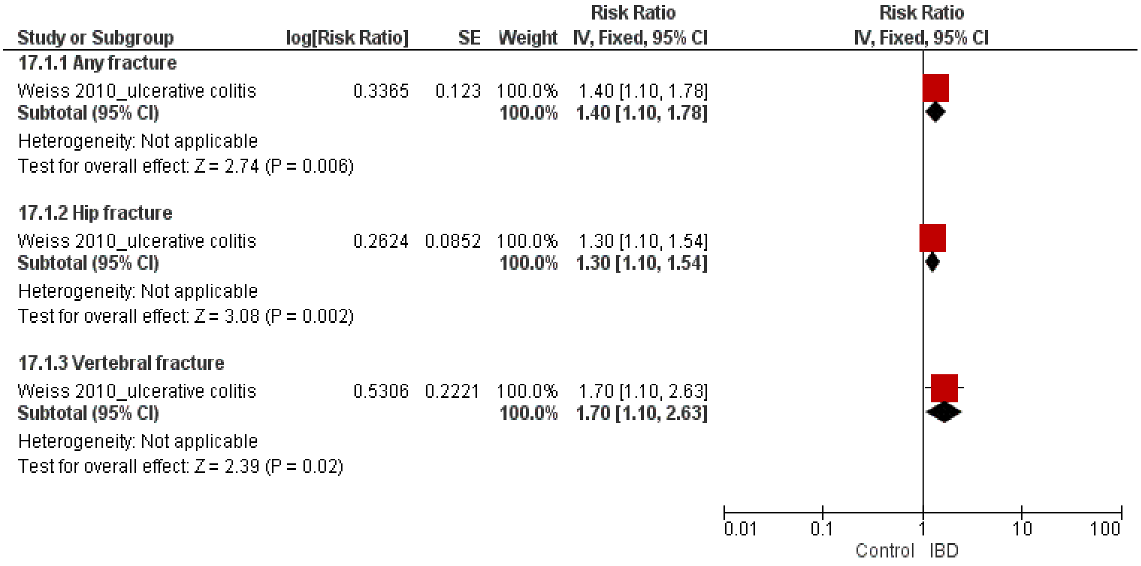

(Re)fracture among patients, fractured or not, exposed to autoimmune diseases compared to unexposed

Study or Subgroup	log[Risk Ratio]	SE	Weight	Risk Ratio IV, Random, 95% CI
**20.1.1 Any fracture**				
Atteritano 2013	2.5649	1.0202	3.8%	13.00 [1.76, 96.01]
Cooper 1995	0.6419	0.3812	16.3%	1.90 [0.90, 4.01]
Rhew 2008	1.1939	0.5605	10.0%	3.30 [1.10, 9.90]
Tedeschi 2019	0.5539	0.0656	34.4%	1.74 [1.53, 1.98]
Weiss 2010	1.0647	0.0179	35.5%	2.90 [2.80, 3.00]
**Subtotal (95% CI)**			**100.0%**	**2.43 [1.61, 3.67]**
Heterogeneity: Tau² = 0.12; Chi² = 59.81, df = 4 (P < 0.00001); I² = 93%				
Test for overall effect: Z = 4.25 (P < 0.0001)				
**20.1.2 Hip fracture**				
Rhew 2008_nonvertebral	1.0986	0.5605	6.9%	3.00 [1.00, 9.00]
Weiss 2010	1.1314	0.1523	93.1%	3.10 [2.30, 4.18]
**Subtotal (95% CI)**			**100.0%**	**3.09 [2.32, 4.13]**
Heterogeneity: Tau² = 0.00; Chi² = 0.00, df = 1 (P = 0.95); I² = 0%				
Test for overall effect: Z = 7.68 (P < 0.00001)				
**20.1.3 Vertebral fracture**				
Rhew 2008	1.0986	1.6269	5.8%	3.00 [0.12, 72.77]
Weiss 2010	0.7885	0.4023	94.2%	2.20 [1.00, 4.84]
**Subtotal (95% CI)**			**100.0%**	**2.24 [1.04, 4.82]**
Heterogeneity: Tau² = 0.00; Chi² = 0.03, df = 1 (P = 0.85); I² = 0%				
Test for overall effect: Z = 2.06 (P = 0.04)				

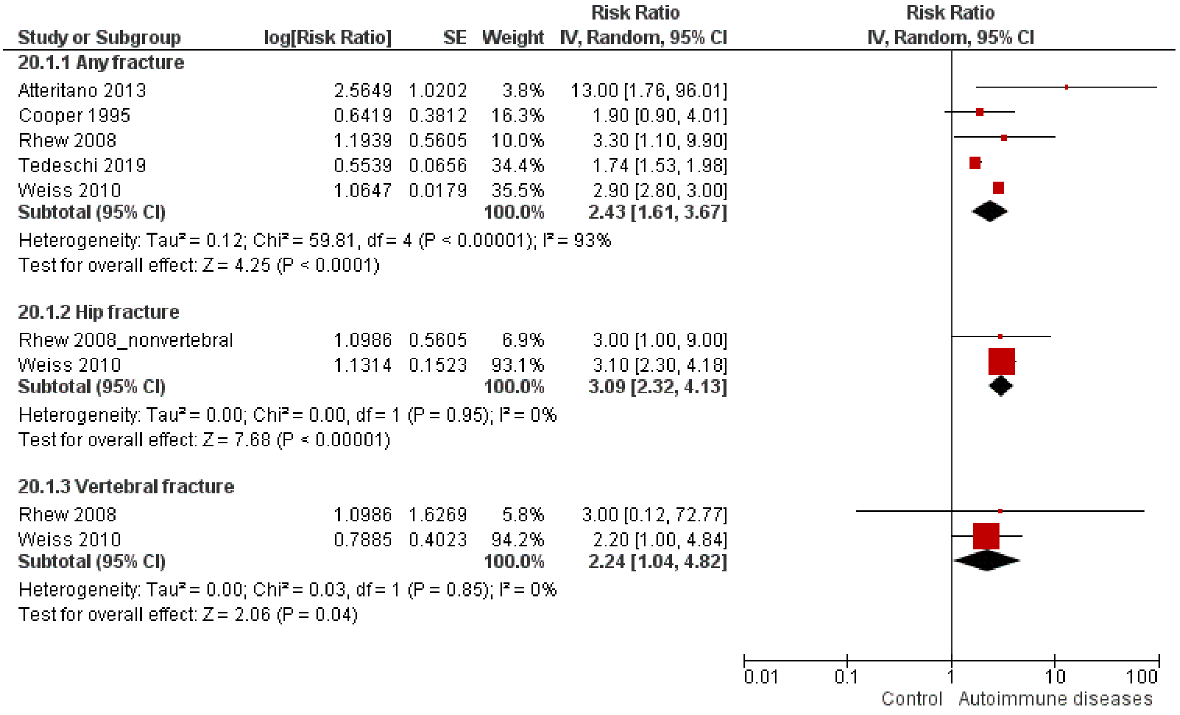

(Re)fracture among patients, fractured or not, exposed to menopausal state compared to unexposed

Study or Subgroup	log[Risk Ratio]	SE	Weight	Risk Ratio IV, Random, 95% CI
Nickolas 2006	0.678	0.7204	18.6%	1.97 [0.48, 8.08]
Sosa 2012	1.211	0.3446	81.4%	3.36 [1.71, 6.60]
**Total (95% CI)**			**100.0%**	**3.04 [1.65, 5.59]**

Heterogeneity: Tau² = 0.00; Chi² = 0.45, df = 1 (P = 0.50); I² = 0%
Test for overall effect: Z = 3.58 (P = 0.0003)

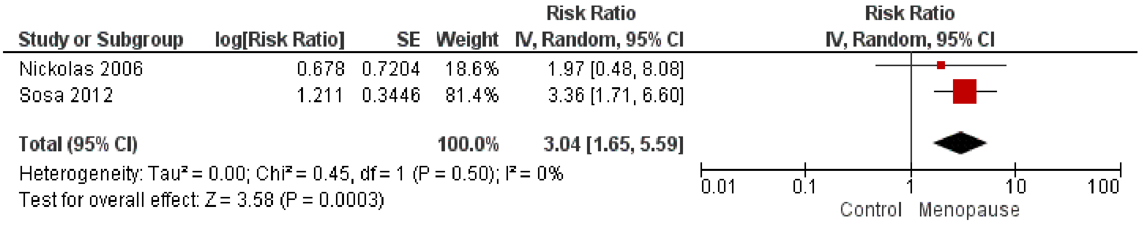

(Re)fracture among patients, fractured or not, exposed to Parkinson disease compared to unexposed

Study or Subgroup	log[Risk Ratio]	SE	Weight	Risk Ratio IV, Random, 95% CI
**18.1.1 Any fracture**				
Bonafede 2016	0.1823	0.0398	19.4%	1.20 [1.11, 1.30]
Hannan 2019	-0.341	1.3842	0.4%	0.71 [0.05, 10.72]
Lee 2018	0.522	0.0532	18.8%	1.69 [1.52, 1.87]
Pouwels 2013	0.6366	0.0631	18.3%	1.89 [1.67, 2.14]
Vestergaard 2007	0.5308	0.0311	19.8%	1.70 [1.60, 1.81]
Weycker 2017_nonvertebral	0.2624	0.1339	13.5%	1.30 [1.00, 1.69]
Yu 2021	0.3805	0.1954	9.8%	1.46 [1.00, 2.15]
**Subtotal (95% CI)**			**100.0%**	**1.53 [1.30, 1.81]**
Heterogeneity: Tau² = 0.04; Chi² = 64.70, df = 6 (P < 0.00001); I² = 91%				
Test for overall effect: Z = 5.01 (P < 0.00001)				
**18.1.2 Hip fracture**				
Bonafede 2016	0.2151	0.0565	34.6%	1.24 [1.11, 1.39]
Pouwels 2013	1.1249	0.1209	33.0%	3.08 [2.43, 3.90]
Vestergaard 2007	0.4824	0.1364	32.5%	1.62 [1.24, 2.12]
**Subtotal (95% CI)**			**100.0%**	**1.83 [1.04, 3.20]**
Heterogeneity: Tau² = 0.23; Chi² = 46.99, df = 2 (P < 0.00001); I² = 96%				
Test for overall effect: Z = 2.10 (P = 0.04)				
**18.1.3 Vertebral fracture**				
Pouwels 2013	0.4318	0.2358	74.4%	1.54 [0.97, 2.44]
Vestergaard 2007	-0.0101	0.4023	25.6%	0.99 [0.45, 2.18]
**Subtotal (95% CI)**			**100.0%**	**1.38 [0.92, 2.05]**
Heterogeneity: Tau² = 0.00; Chi² = 0.90, df = 1 (P = 0.34); I² = 0%				
Test for overall effect: Z = 1.57 (P = 0.12)				

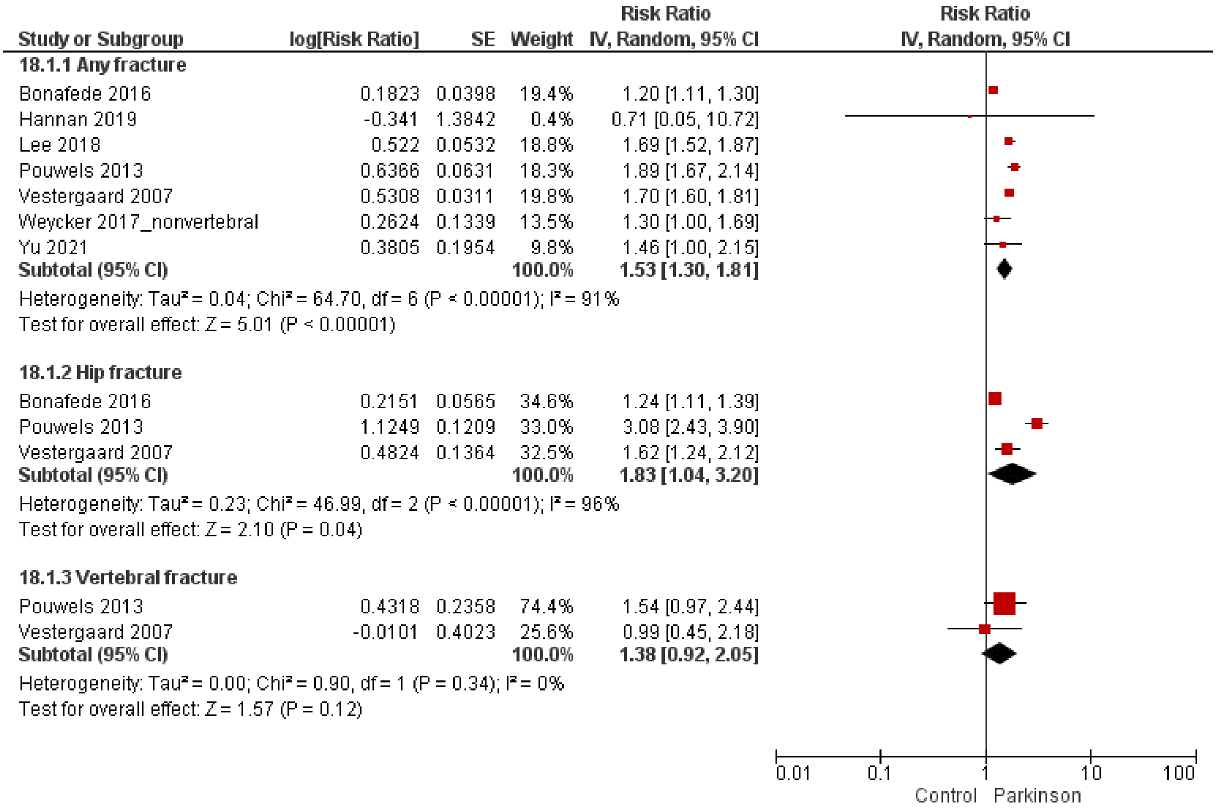

(Re)fracture among patients, fractured or not, exposed to previous fractures compared to unexposed

Study or Subgroup	log[Risk Ratio]	SE	Weight	Risk Ratio IV, Random, 95% CI
Adachi 2019	0.5306	0.2707	0.8%	1.70 [1.00, 2.89]
Anpalahan 2014	0.6931	0.3239	0.6%	2.00 [1.06, 3.77]
Dennison 2012	0.7885	0.0237	10.4%	2.20 [2.10, 2.30]
Gehlbach 2011	0.7839	0.0387	9.0%	2.19 [2.03, 2.36]
Gibson-Smith 2014	0.2624	0.0538	7.6%	1.30 [1.17, 1.44]
Hannan 2019	0.4055	0.1536	2.2%	1.50 [1.11, 2.03]
Inose 2021	0.3688	0.5071	0.2%	1.45 [0.54, 3.91]
Jadoul 2006	1.1537	0.2029	1.4%	3.17 [2.13, 4.72]
Krege 2006	1.5254	0.2757	0.8%	4.60 [2.68, 7.89]
Loh 2008	0.2469	0.4039	0.4%	1.28 [0.58, 2.83]
Prieto-Alhambra 2015	0.7125	0.0048	11.4%	2.04 [2.02, 2.06]
Reber 2018	0.9123	0.0609	6.9%	2.49 [2.21, 2.81]
Sheer 2020	0.2311	0.0378	9.1%	1.26 [1.17, 1.36]
Simonelli 2003	0.3389	0.4672	0.3%	1.40 [0.56, 3.51]
Siris 2001 (wrist)	0.5822	0.0702	6.1%	1.79 [1.56, 2.05]
Torgerson 1996	1.07	0.2989	0.7%	2.92 [1.62, 5.24]
Vestergaard 2007	0.7125	0.0048	11.4%	2.04 [2.02, 2.06]
Vochteloo 2012	0.3047	0.1019	4.0%	1.36 [1.11, 1.66]
Weycker 2017	0.3365	0.123	3.1%	1.40 [1.10, 1.78]
Yu 2021	0.227	0.1805	1.7%	1.25 [0.88, 1.79]
Yusuf 2020	0.793	0.0093	11.3%	2.21 [2.17, 2.25]
Zhou 2021	0.9002	0.3478	0.5%	2.46 [1.24, 4.86]
**Total (95% CI)**			**100.0%**	**1.88 [1.79, 1.98]**

Heterogeneity: Tau² = 0.01; Chi² = 386.40, df = 21 (P < 0.00001); I² = 95%
Test for overall effect: Z = 24.81 (P < 0.00001)

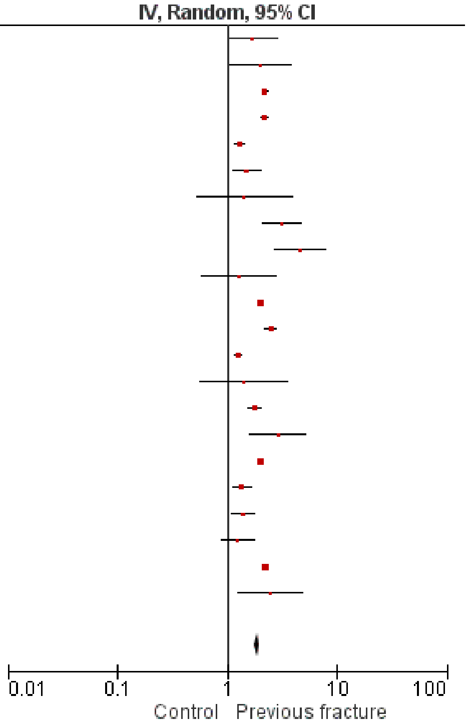

(Re)fracture among patients, fractured or not, exposed to dementia compared to unexposed

Study or Subgroup	log[Risk Ratio]	SE	Weight	Risk Ratio IV, Random, 95% CI
**4.1.1 Any fracture**				
Bonafede 2016	0.2231	0.047	17.0%	1.25 [1.14, 1.37]
Sheer 2020	0.1989	0.0214	82.2%	1.22 [1.17, 1.27]
Vestergaard 2007	0.9625	0.8645	0.1%	2.62 [0.48, 14.25]
**Subtotal (95% CI)**			**99.3%**	**1.23 [1.18, 1.27]**
Heterogeneity: Tau² = 0.00; Chi² = 0.99, df = 2 (P = 0.61); I² = 0%				
Test for overall effect: Z = 10.45 (P < 0.00001)				
**4.1.2 Vertebral fracture**				
Yu 2021	-0.1369	0.2685	0.5%	0.87 [0.52, 1.48]
**Subtotal (95% CI)**			**0.5%**	**0.87 [0.52, 1.48]**
Heterogeneity: Not applicable				
Test for overall effect: Z = 0.51 (P = 0.61)				
**4.1.3 Non vertebral fracture**				
Hannan 2019	0.6931	0.4189	0.2%	2.00 [0.88, 4.55]
**Subtotal (95% CI)**			**0.2%**	**2.00 [0.88, 4.55]**
Heterogeneity: Not applicable				
Test for overall effect: Z = 1.65 (P = 0.10)				
**Total (95% CI)**			**100.0%**	**1.22 [1.18, 1.27]**

Heterogeneity: Tau² = 0.00; Chi² = 3.96, df = 4 (P = 0.41); I² = 0%
Test for overall effect: Z = 10.45 (P < 0.00001)
Test for subgroup differences: Chi² = 2.97, df = 2 (P = 0.23), I² = 32.7%

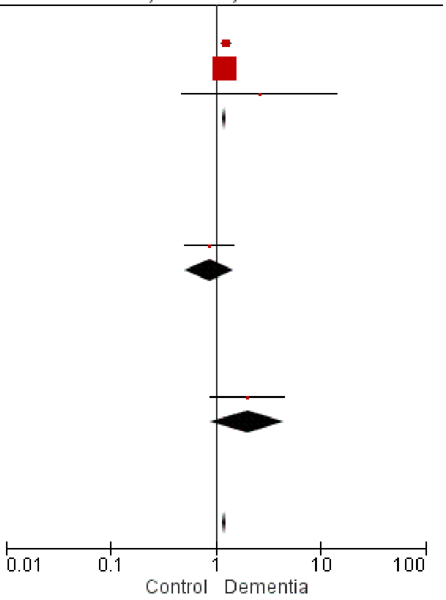

(Re)fracture among patients, fractured or not, exposed to chronic kidney disease compared to unexposed

Study or Subgroup	log[Risk Ratio]	SE	Weight	Risk Ratio IV, Random, 95% CI
**5.1.1 Any fracture**				
Malmgren 2019	0.793	0.2826	25.2%	2.21 [1.27, 3.85]
**Subtotal (95% CI)**			**25.2%**	**2.21 [1.27, 3.85]**
Heterogeneity: Not applicable				
Test for overall effect: Z = 2.81 (P = 0.005)				
**5.1.2 Hip fracture**				
Nickolas 2006	0.8416	0.367	19.2%	2.32 [1.13, 4.76]
**Subtotal (95% CI)**			**19.2%**	**2.32 [1.13, 4.76]**
Heterogeneity: Not applicable				
Test for overall effect: Z = 2.29 (P = 0.02)				
**5.1.3 Vertebral fracture**				
Yu 2021	-0.01	0.2318	29.6%	0.99 [0.63, 1.56]
**Subtotal (95% CI)**			**29.6%**	**0.99 [0.63, 1.56]**
Heterogeneity: Not applicable				
Test for overall effect: Z = 0.04 (P = 0.97)				
**5.1.4 Non vertebral fracture**				
Hannan 2019	0.3365	0.2729	26.0%	1.40 [0.82, 2.39]
**Subtotal (95% CI)**			**26.0%**	**1.40 [0.82, 2.39]**
Heterogeneity: Not applicable				
Test for overall effect: Z = 1.23 (P = 0.22)				
**Total (95% CI)**			**100.0%**	**1.56 [1.04, 2.35]**
Heterogeneity: Tau² = 0.09; Chi² = 6.56, df = 3 (P = 0.09); I² = 54%				
Test for overall effect: Z = 2.13 (P = 0.03)				
Test for subgroup differences: Chi² = 6.56, df = 3 (P = 0.09), I² = 54.3%				

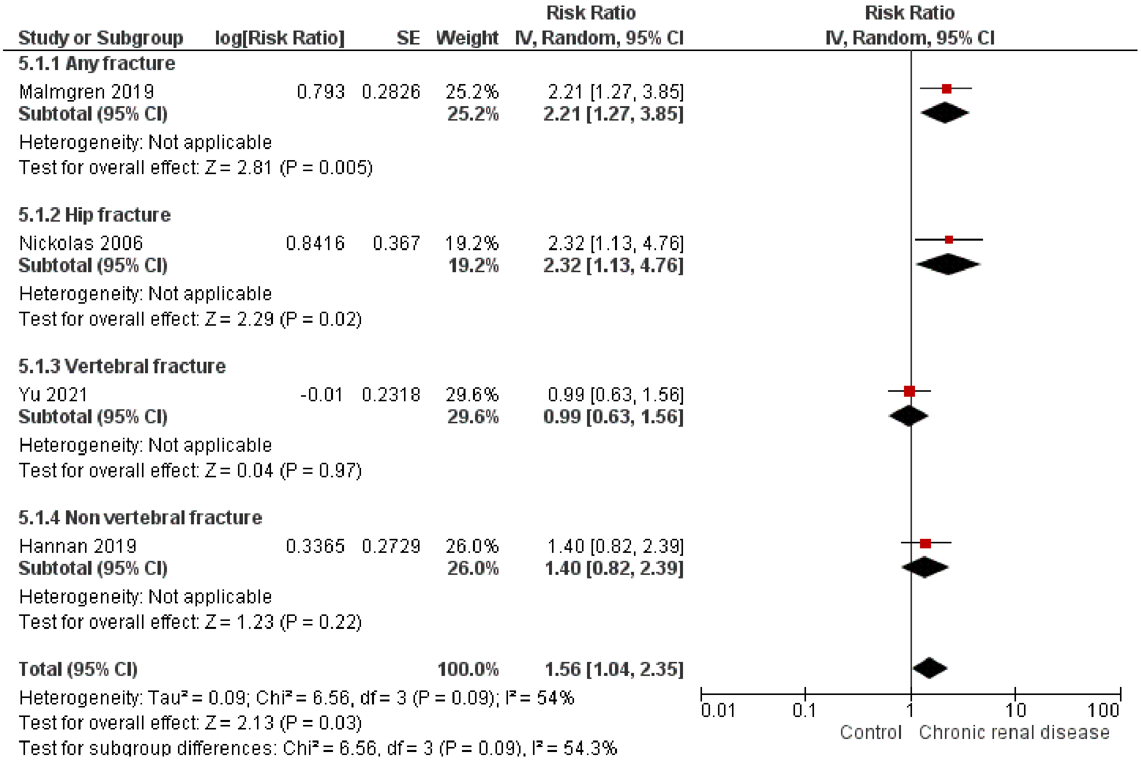

Quality evaluation

Observational studies evaluated with the Newcastle Ottawa Scale

	Selection				Comparability	Outcome			
Cohort study	Representativeness of the exposed cohort	Selection of the non exposed cohort	Ascertainment of exposure	Demonstration that outcome of interest was not present at start of study	Comparability of cohorts on the basis of the design or analysis	Assessment of outcome	Was follow-up long enough for outcomes to occur	Adequacy of follow-up of cohorts	tot
Adachi 2019	1		1	1		1	1		5
Berry 2017	1	1	1	1	1	1	1		7
Compston 2011	1	1		1	1	1	1		6
Dennison 2012	1	1		1	1	1	1		6
Dobnig 2006	1		1	1	1	1	1		6
Gehlbach 2011	1			1	1	1	1		5
Gibson-Smith 2014	1	1	1	1	1	1	1		7
Hannan 2019	1		1	1	1	1	1		6
Inose 2020	1	1	1	1	1	1	1		7
Ivers 2001	1	1	1	1	1	1	1		7
Krege 2006	1	1	1	1		1	1		6
Lindsay 2018	1		1	1	1	1	1		6

Loh 2008	1		1	1	1	1	1		6
Lyles 2008	1	1	1	1	1	1	1		7
Malmgren 2020	1	1	1	1	1	1	1		7
Morse 2009		1	1	1	1	1	1		6
Pouwels 2013	1	1	1	1	1	1	1		7
Reber 2018	1		1	1		1	1		5
Rhew 2007	1	1	1	1		1	1	1	7
Ruan 2011	1	1	1	1		1	1		6
Sheer 2020	1	1	1	1	1	1	1		7
Shim 2020	1	1	1	1	1	1	1		7
Simonelli 2003	1	1	1	1		1	1		6
Siris 2001	1	1		1	1	1	1		6
Soriano 2005	1	1	1	1		1	1		6
Tedeschi 2019		1	1	1	1	1	1		6
Torgerson 1996			1	1	1	1	1		5
Vestergaard 2005	1		1	1	1	1	1		6
Vochteloo 2012			1	1	1	1	1		5
Waterloo 2012	1	1	1	1	1				5
Weycker 2017			1	1	1	1	1		5

Yusuf 2020	1		1	1	1	1	1		6
Zhou 2021			1	1	1	1	1	1	6

	Selection				Comparability	Exposure			
Case-control	Is the case definition adequate	Representativeness of the cases	Selection of controls	Definition of controls	Comparability of cases and controls on the basis of the design or analysis	Ascertainment of exposure	Same method of ascertainment for cases and controls	Non-response rate	tot
Anpalahan 2014	1	1		1		1	1		5
Bonafede 2016	1	1	1	1		1	1		6
Cooper 1995	1	1	1	1	1	1	1		7
Lee 2018	1	1	1	1	1	1	1		7
Meyer 1995	1	1	1	1	1	1	1		7
Prieto-Alhambra 2014	1	1	1	1	1	1	1		7
Prieto-Alhambra 2015	1	1	1	1	1	1	1		7
Vestergaard 1998		1	1	1			1	1	5
Vestergaard 2007	1	1	1	1	1	1	1		7
Weiss 2010	1	1		1	1	1	1		6
Yu 2021	1	1		1	1	1	1		6

	Selection				Comparability	Outcome		
Cross-sectional*	Representativeness of the sample	Sample size	Non-respondents	Ascertainment of the exposure (risk factor)	The subjects in different outcome groups are comparable, based on the study design or analysis.	Assessment of the outcome	Statistical test	tot
Atteritano 2013	1			1	1	1	1	5
Adas-Okuma 2020	1			1	1	1	1	5
Jadoul 2005	1	1		1	1	1	1	6
Nickolas 2006	1			1	1	1	1	5
Sosa-Henrlquez 2012	1				1	1	1	4

*From: Herzog R, Álvarez-Pasquin MJ, Díaz C, Del Barrio JL, Estrada JM, Gil Á. Are healthcare workers' intentions to vaccinate related to their knowledge, beliefs and attitudes? A systematic review. BMC Public Health. 2013 Feb 19;13:154. doi: 10.1186/1471-2458-13-154. PMID: 23421987; PMCID: PMC3602084.

Summary of Findings

CI: Confidence interval; **HR:** Hazard Ratio; **OR**: Odds Ratio; **RR**: relative risk

Certainty assessment							Effect		Certainty	Importance
**№ of studies**	**Study design**	**Risk of bias**	**Inconsistency**	**Indirectness**	**Imprecision**	**Other considerations**	**Relative (95% CI)**	**Absolute (95% CI)**		
**Adjuvant hormone therapy**										
1	observational studies	serious [a]	not serious	not serious	serious [b]	none	**HR 1.35** (0.98 to 1.86)	**1 fewer per 1.000** (from 2 fewer to 1 fewer)	⊕◯◯◯ VERY LOW	CRITICAL

Explanations

a. Risk of Bias due to Selection of the non exposed cohort, Comparability of cohorts on the basis of the design or analysis, Adequacy of follow-up of cohorts
b. Confidence intervals crossed the line of no difference with plausible effects in favor to the experimental group.

Certainty assessment							Effect		Certainty	Importance
**№ of studies**	**Study design**	**Risk of bias**	**Inconsistency**	**Indirectness**	**Imprecision**	**Other considerations**	**Relative (95% CI)**	**Absolute (95% CI)**		
**AIDS**										
1	observational studies	not serious	not serious	not serious	serious [a]	none	**OR 1.20** (0.53 to 2.72)	**0 fewer per 1.000** (from 0 fewer to 0 fewer)	⊕◯◯◯ VERY LOW	CRITICAL

Explanations

a. Confidence intervals crossed the line of no difference with plausible effects in favor to the experimental group.

Certainty assessment							Effect		Certainty	Importance
№ of studies	Study design	Risk of bias	Inconsistency	Indirectness	Imprecision	Other considerations	Relative (95% CI)	Absolute (95% CI)		
**COPD**										
6	observational studies	not serious	serious [a]	not serious	not serious	none	**RR 1.36** (1.21 to 1.53)	**1 fewer per 1.000** (from 2 fewer to 1 fewer)	⊕◯◯◯ VERY LOW	CRITICAL

Explanations

a. I2 > 75%

Certainty assessment							Effect		Certainty	Importance
№ of studies	Study design	Risk of bias	Inconsistency	Indirectness	Imprecision	Other considerations	Relative (95% CI)	Absolute (95% CI)		
**Corticosteroid**										
8	observational studies	not serious	very serious [a]	not serious	not serious	none	**RR 1.23** (1.13 to 1.32)	**0 fewer per 1.000** (from 0 fewer to 0 fewer)	⊕◯◯◯ VERY LOW	CRITICAL

Explanations

a. I2 > 90%

Certainty assessment							Effect		Certainty	Importance
№ of studies	Study design	Risk of bias	Inconsistency	Indirectness	Imprecision	Other considerations	Relative (95% CI)	Absolute (95% CI)		
**Diabetes**										
10	observational studies	not serious	not serious	not serious	not serious	none	**RR 1.15** (1.07 to 1.24)	**1 fewer per 1.000** (from 1 fewer to 1 fewer)	⊕⊕◯◯ LOW	CRITICAL

Certainty assessment							Effect		Certainty	Importance
№ of studies	Study design	Risk of bias	Inconsistency	Indirectness	Imprecision	Other considerations	Relative (95% CI)	Absolute (95% CI)		
**65-74 years**										
1	observational study	not serious	not serious	not serious	not serious	none	**RR 1.20** (1.10 to 1.31)	**1 fewer per 1.000** (from 1 fewer to 1 fewer)	⨁⨁◯◯ LOW	CRITICAL
**75-84 years**										
1	observational study	not serious	not serious	not serious	not serious	none	**RR 1.80** (1.60 to 2.03)	**2 fewer per 1.000** (from 2 fewer to 2 fewer)	⨁⨁◯◯ LOW	CRITICAL
**85 + years**										
1	observational study	not serious	not serious	not serious	not serious	none	**RR 2.30** (2.00 to 2.64)	**2 fewer per 1.000** (from 3 fewer to 2 fewer)	⨁⨁◯◯ LOW	CRITICAL

Certainty assessment							Effect		Certainty	Importance
№ of studies	Study design	Risk of bias	Inconsistency	Indirectness	Imprecision	Other considerations	Relative (95% CI)	Absolute (95% CI)		
**Family history of fractures**										
6	observational studies	serious [a]	very serious [b]	not serious	not serious	none	**RR 2.66** (1.32 to 5.35)	**3 fewer per 1.000** (from 5 fewer to 1 fewer)	⨁◯◯◯ VERY LOW	CRITICAL

Explanations

a. Risk of Bias due to Representativeness of the exposed cohort (Torgerson 1996), Selection of the non exposed cohort (Anpalahan 2014, Sosa 2012, Torgerson 1996), Ascertainment of exposure (Sosa 2012), Comparability of cohorts on the basis of the design or analysis (Anpalahan 2014, Sosa 2012), Adequacy of follow-up of cohorts (Anpalahan 2014, Sosa 2012, Torgerson 1996)

b. I2 > 90%

Certainty assessment							Effect		Certainty	Importance
№ of studies	Study design	Risk of bias	Inconsistency	Indirectness	Imprecision	Other considerations	Relative (95% CI)	Absolute (95% CI)		
**Women**										
12	observational studies	not serious	very serious [a]	not serious	serious [b]	none	**RR 1.19** (0.95 to 1.49)	**1 fewer per 1.000** (from 1 fewer to 1 fewer)	⊕◯◯◯ VERY LOW	CRITICAL

Explanations

a. I2 > 90%

b. Confidence intervals crossed the line of no difference with plausible effects in favor to the experimental group.

Certainty assessment							Effect		Certainty	Importance
№ of studies	Study design	Risk of bias	Inconsistency	Indirectness	Imprecision	Other considerations	Relative (95% CI)	Absolute (95% CI)		
**Severe physical disabilities**										
6	observational studies	serious [a]	serious [b]	not serious	serious [c]	none	**RR 1.86** (0.85 to 4.06)	**0 fewer per 1.000** (from 0 fewer to 0 fewer)	⊕◯◯◯ VERY LOW	CRITICAL

Explanations

a. Risk of bias due to Selection of the non exposed cohort, Ascertainment of exposure, Comparability of cohorts on the basis of the design or analysis, Adequacy of follow-up of cohorts

b. I2 > 75%

c. Confidence intervals crossed the line of no difference with plausible effects in favor to the experimental group.

Certainty assessment							Effect		Certainty	Importance
**№ of studies**	**Study design**	**Risk of bias**	**Inconsistency**	**Indirectness**	**Imprecision**	**Other considerations**	**Relative (95% CI)**	**Absolute (95% CI)**		
**low BMI vs normal**										
7	observational studies	not serious	serious [a]	not serious	not serious	none	**OR 1.28** (1.07 to 1.52)	**1 fewer per 1.000** (from 2 fewer to 1 fewer)	⊕◯◯◯ VERY LOW	CRITICAL

Explanations

a. I2 > 75%

Certainty assessment							Effect		Certainty	Importance
**№ of studies**	**Study design**	**Risk of bias**	**Inconsistency**	**Indirectness**	**Imprecision**	**Other considerations**	**Relative (95% CI)**	**Absolute (95% CI)**		
**Inflammatory bowel disease**										
1	observational studies	not serious	not serious	not serious	not serious	none	**RR 1.40** (1.10 to 1.78)	**0 fewer per 1.000** (from 0 fewer to 0 fewer)	⊕⊕◯◯ LOW	CRITICAL

Certainty assessment							Effect		Certainty	Importance
№ of studies	Study design	Risk of bias	Inconsistency	Indirectness	Imprecision	Other considerations	Relative (95% CI)	Absolute (95% CI)		
**Autoimmune diseases**										
5	observational studies	not serious	very serious [a]	not serious	not serious	none	**RR 2.43** (1.61 to 3.67)	**2 fewer per 1.000** (from 4 fewer to 2 fewer)	⨁◯◯◯ VERY LOW	CRITICAL

Explanations

a. I2 > 90%

Certainty assessment							Effect		Certainty	Importance
№ of studies	Study design	Risk of bias	Inconsistency	Indirectness	Imprecision	Other considerations	Relative (95% CI)	Absolute (95% CI)		
**Parkinson disease**										
7	observational studies	not serious	very serious [a]	not serious	not serious	none	**RR 1.53** (1.30 to 1.81)	**0 fewer per 1.000** (from 0 fewer to 0 fewer)	⨁◯◯◯ VERY LOW	CRITICAL

Explanations

a. I2 > 90%

Certainty assessment							Effect		Certainty	Importance
№ of studies	Study design	Risk of bias	Inconsistency	Indirectness	Imprecision	Other considerations	Relative (95% CI)	Absolute (95% CI)		
**Previous fractures**										
22	observational studies	not serious	very serious [a]	not serious	not serious	none	**RR 1.88** (1.79 to 1.98)	**2 fewer per 1.000** (from 2 fewer to 2 fewer)	⊕◯◯◯ VERY LOW	CRITICAL

Explanations

a. I2>90%

b. Risk of bias due to Representativeness of the exposed cohort (Torgerson 1996), Selection of the non exposed cohort (Torgerson 1996, Gehlback 2011), Ascertainment of exposure (Gehlback 2011), Adequacy of follow-up of cohorts (Torgerson 1996, Gehlback 2011)

Certainty assessment							Effect		Certainty	Importance
№ of studies	Study design	Risk of bias	Inconsistency	Indirectness	Imprecision	Other considerations	Relative (95% CI)	Absolute (95% CI)		
**Menopausal state**										
2	observational studies	serious [a]	not serious	not serious	not serious	not	**RR 3.04** (1.65 to 5.59)	**3 fewer per 1.000** (from 6 fewer to 2 fewer)	⊕◯◯◯ VERY LOW	CRITICAL

CI: Confidence interval; **RR:** Risk ratio

Explanations

a. Risk of Bias (Sosa 2012) due to Selection of the non exposed cohort, Ascertainment of exposure, Comparability of cohorts on the basis of the design or analysis and Adequacy of follow-up of cohorts

Certainty assessment							Effect		Certainty	Importance
**№ of studies**	**Study design**	**Risk of bias**	**Inconsistency**	**Indirectness**	**Imprecision**	**Other considerations**	**Relative (95% CI)**	**Absolute (95% CI)**		
**Dementia**										
5	observational studies	not serious	not serious	not serious	not serious	none	**RR 1.22** (1.18 to 1.27)	**0 fewer per 1.000** (from 0 fewer to 0 fewer)	⨁⨁◯◯ LOW	CRITICAL

CI: Confidence interval; **RR:** Risk ratio

Certainty assessment							Effect		Certainty	Importance
**№ of studies**	**Study design**	**Risk of bias**	**Inconsistency**	**Indirectness**	**Imprecision**	**Other considerations**	**Relative (95% CI)**	**Absolute (95% CI)**		
**Chronic renal disease**										
4	observational studies	not serious	not serious	not serious	not serious	none	**RR 1.56** (1.04 to 2.35)	**2 fewer per 1.000** (from 2 fewer to 1 fewer)	⨁⨁◯◯ LOW	CRITICAL

CI: Confidence interval; **RR:** Risk ratio

CQ 4

Which therapeutic strategy should be recommended in the short- and long-term treatment of patients at high or imminent risk of (re)fracture?

Search strategy

MEDLINE

Up to 22 February 2021

#1:

(((wrist* or colles or radius or articulatio radiocarpea or carpus or carpal or radiocarp* or radial or forearm* or humerus or metacarp* or barton or monteggi* or ulna or ulnar or upper limb* or hip or hips or trochanteric or intertrochanteric or subtrochanteric or femoral neck or femur neck or spine or spinal or vertebra or vertebral or vertebrae or lumbar or shoulder* or glenohumeral or humeroscapular or scapulo humeral or proximal humeral) adj3 fractur*) or (exp hip fractures/ or spinal fractures/ or shoulder fractures/ or osteoporotic fractures/ or exp radius fractures/) or (fractures, bone/ and (exp wrist joint/ or exp spine/ or shoulder/ or shoulder joint/ or hip/))) and (exp osteoporosis/ or (osteoporo* or bone loss*))

#2:

“fragility fracture”[ti] OR “fragility fractures”[ti] OR “low energy fracture”[ti] OR “low energy fractures”[ti] OR “low-energy fracture”[ti] OR “low-energy fractures”[ti] OR “low trauma fracture”[ti] OR “low trauma fractures”[ti] OR “low-trauma fracture”[ti] OR “low-trauma fractures”[ti] OR “low energy trauma”[ti] OR “low-energy trauma”[ti] OR “low level trauma”[ti] OR “low-level trauma”[ti] OR “minor trauma fracture”[ti] OR “minor trauma fractures”[ti] OR “minor-trauma fracture”[ti] OR “minor-trauma fractures”[ti] OR “minor fracture”[ti] OR “minor fractures”[ti] OR “minor-fracture”[ti] OR “minor-fractures”[ti] OR “osteoporotic fracture”[ti] OR “osteoporotic fractures”[ti]

#3: #1 OR #2

#4:

switch*[tiab] OR sequen*[tiab] OR “after therapy”[tiab] OR pretreated[tiab] OR “pre-treated”[tiab] OR “previous treated”[tiab] or “previous treatment”[tiab] OR pretreatment[tiab] OR “pre-treatment”[tiab] OR “prior treated”[tiab] OR “prior treatment”[tiab] OR transition*[tiab] OR follow*[tiab] OR after[ti]

#5:

teriparatide[ti] OR romosozumab[ti] OR risedronate[ti] OR denosumab[ti] OR alendronate[ti] OR zoledronate[ti] OR bisphosphonate*[ti] OR anabolic[ti] OR antiresorptive[ti]

#6: #4 AND #5

#7: #3 AND #6

#8: #7 Filters: Humans, from 2019 - 2021

EMBASE

Up to 22 February 2021

#1:

'wrist fracture'/exp OR 'hip fracture'/exp OR 'spine fracture'/exp OR 'shoulder fracture'/exp OR 'fragility fracture'/exp OR 'radius fracture'/exp OR ((wrist* OR colle* OR radius OR 'articulatio radiocarpea' OR carpus OR carpal OR radiocarp* OR radial OR forearm* OR humerus OR metacarp* OR barton OR monteggi* OR ulna OR ulnar OR 'upper limb' OR 'upper limbs' OR hip OR hips OR trochanteric OR intertrochanteric OR subtrochanteric OR 'femoral neck' OR 'femur neck' OR spine OR spinal OR vertebra* OR lumbar OR shoulder* OR glenohumeral OR humeroscapular OR 'scapulo humeral' OR 'proximal humeral') NEAR/3 fractur*):ab,ti OR ('fracture'/exp AND ('wrist'/exp OR 'hip'/exp OR 'spine'/exp OR 'shoulder'/exp OR 'wrist injury'/de OR 'shoulder injury'/exp OR 'hip injury'/exp OR 'spine injury'/exp)) AND ('osteoporosis'/exp OR osteoporo*:ab,ti OR 'bone loss':ab,ti)

#2: 'fragility fracture'/exp

#3: 'low energy fracture'/exp

#4: 'low trauma fracture'/exp

#5: 'low energy trauma'/exp

#6:

“fragility fracture”:ti OR “fragility fractures”:ti OR “low energy fracture”:ti OR “low energy fractures”:ti OR “low-energy fracture”:ti OR “low-energy fractures”:ti OR “low trauma fracture”:ti OR “low trauma fractures”:ti OR “low-trauma fracture”:ti OR “low-trauma fractures”:ti OR “low energy trauma”:ti OR “low-energy trauma”:ti OR “low level trauma”:ti OR “low-level trauma”:ti OR “minor trauma fracture”:ti OR “minor trauma fractures”:ti OR “minor-trauma fracture”:ti OR “minor-trauma fractures”:ti OR “minor fracture”:ti OR “minor fractures”:ti OR “minor-fracture”:ti OR “minor-fractures”:ti OR “osteoporotic fracture”:ti OR “osteoporotic fractures”:ti

#7: #1 OR #2 OR #3 OR #4 OR #5 OR #6

#8:

switch*:ti,ab OR sequen*:ti,ab OR “after therapy”:ti,ab OR pretreated:ti,ab OR “pre-treated”:ti,ab OR “previous treated”:ti,ab or “previous treatment”:ti,ab OR pretreatment:ti,ab OR “pre-treatment”:ti,ab OR “prior treated”:ti,ab OR “prior treatment”:ti,ab OR transition*:ti,ab OR follow*:ti,ab OR after:ti

#9:

teriparatide:ti OR romosozumab:ti OR risedronate:ti OR denosumab:ti OR alendronate:ti OR zoledronate:ti OR bisphosphonate*:ti OR anabolic:ti OR antiresorptive:ti

#10: #8 AND #9

#11: #7 AND #10

#12:

#11 AND (2019:py OR 2020:py OR 2021:py) AND [embase]/lim NOT ([embase]/lim AND [medline]/lim)

COCHRANE

Up to 22 February 2021

#1:

((wrist* or colle* or radius or "articulatio radiocarpea" or carpus or carpal or radiocarp* or radial or forearm* or humerus or metacarp* or barton or monteggi* or ulna or ulnar or "upper limb" or "upper limbs" or hip or hips or trochanteric or intertrochanteric or subtrochanteric or "femoral neck" or "femur neck" or spine or spinal or vertebra* or lumbar or shoulder* or glenohumeral or humeroscapular or "scapulo humeral" or "proximal humeral") near/3 fractur*):ti,ab or [mh "hip fractures"] or [mh "spinal fractures"] or [mh "shoulder fractures"] or [mh "osteoporotic fractures"] or [mh "radius fractures"] or ([mh "bone fractures"] and ([mh "wrist joint"] or [mh spine] or [mh shoulder] or [mh "shoulder joint"] or [mh hip])) and ([mh osteoporosis] or (osteoporo* or "bone loss" OR fragility):ti,ab)

#2: MeSH descriptor: [Osteoporotic Fractures] explode all trees

#3: MeSH descriptor: [Fractures, Spontaneous] explode all trees

#4:

(fragility fracture):ti OR (fragility fractures):ti OR (low energy fracture):ti OR (low energy fractures):ti OR (low-energy fracture):ti OR (low-energy fractures):ti OR (low trauma fracture):ti OR (low trauma fractures):ti OR (low-trauma fracture):ti OR (low-trauma fractures):ti OR (low energy trauma):ti OR (low-energy trauma):ti OR (low level trauma):ti OR (low-level trauma):ti OR (minor

trauma fracture):ti OR (minor trauma fractures):ti OR (minor-trauma fracture):ti OR (minor-trauma fractures):ti OR (minor fracture):ti OR (minor fractures):ti OR (minor-fracture):ti OR (minor-fractures):ti OR (osteoporotic fracture):ti OR (osteoporotic fractures):ti OR (pathologic fracture):ti OR (pathological fractures):ti

#5: #1 OR #2 OR #3 OR #4

#6:

switch*:ti,ab OR sequen*:ti,ab OR “after therapy”:ti,ab OR pretreated:ti,ab OR “pre-treated”:ti,ab OR “previous treated”:ti,ab or “previous treatment”:ti,ab OR pretreatment:ti,ab OR “pre-treatment”:ti,ab OR “prior treated”:ti,ab OR “prior treatment”:ti,ab OR transition*:ti,ab OR follow*:ti,ab OR after:ti

#7:

teriparatide:ti OR romosozumab:ti OR risedronate:ti OR denosumab:ti OR alendronate:ti OR zoledronate:ti OR bisphosphonate*:ti OR anabolic:ti OR antiresorptive:ti

#8: #6 AND #7

#9: #5 AND #8

#10: #9 with Cochrane Library publication date from Jan 2019 to Dec 2021

Flow chart

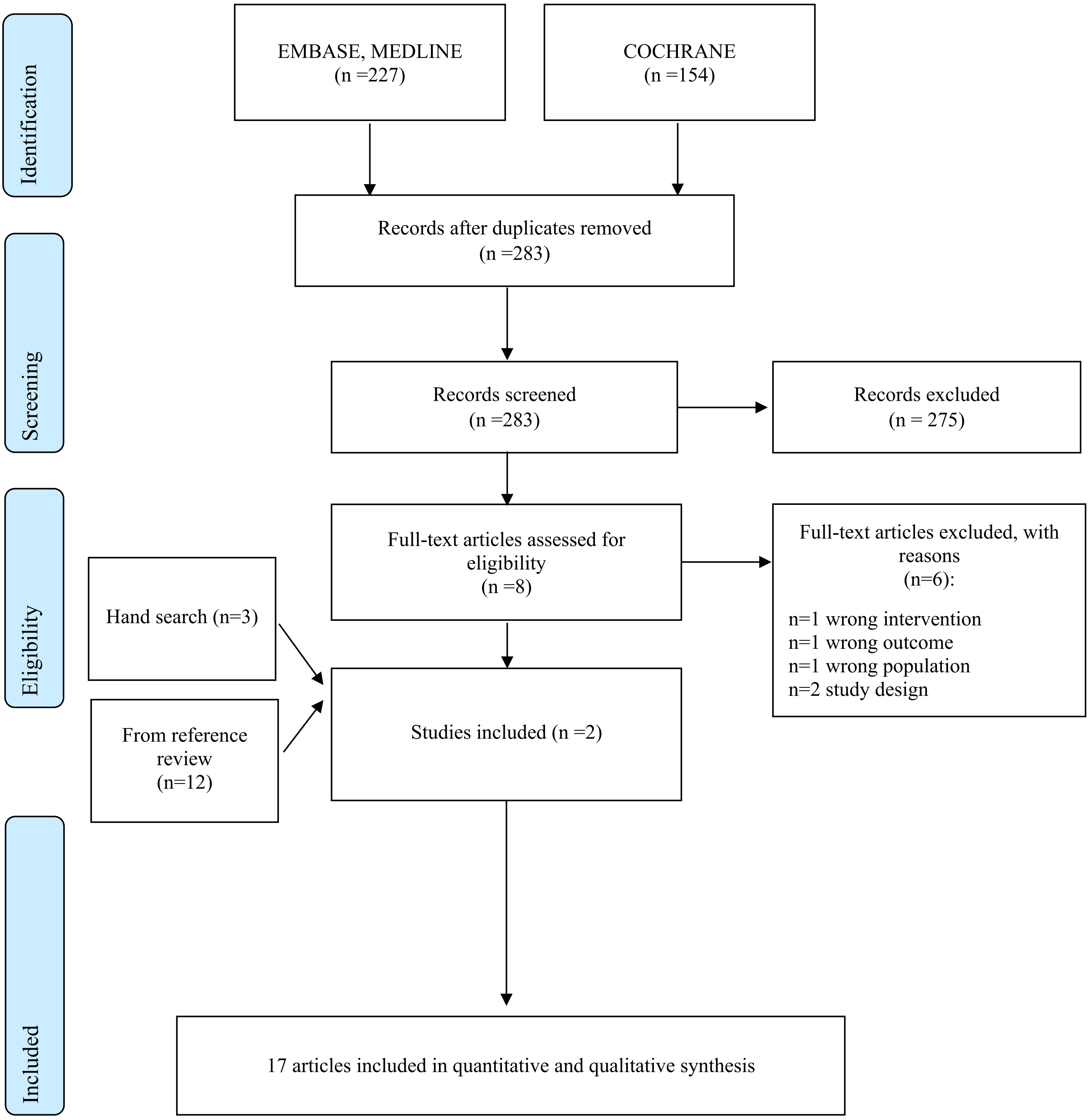
Identification
Screening
Eligibility
Included
EMBASE, MEDLINE (n =227)
COCHRANE (n =154)
Records after duplicates removed (n =283)
Records screened (n =283)
Records excluded (n = 275)
Full-text articles assessed for eligibility (n =8)
Full-text articles excluded, with reasons (n=6):
n=1 wrong intervention
n=1 wrong outcome
n=1 wrong population
n=2 study design
Hand search (n=3)
From reference review (n=12)
Studies included (n =2)
17 articles included in quantitative and qualitative synthesis

Results

DXA change from the switching: Anabolic to Antiresorptive, or viceversa

First author, Year	Months from baseline	Months from switch	Comparative LS BMD change from baseline (mean%)			Comparative TH BMD change from baseline (mean%)			Comparative FN BMD change from baseline (mean%)		
			Group 1	Group 2	p-value	Group 1	Group 2	p-value	Group 1	Group 2	p-value
**Group 1: Romo to Dmab; Group 2: placebo to Dmab**											
Cosman 2016			N=65	N=61		N=66	N=62		N=66	N=62	
FRAME Study	18	6	15.1	3.3	P < 0.001	8.4	1.6	P < 0.001	6.7	-0.2	P < 0.001
	24	12	17.6	5.0	P < 0.001	8.8	2.9	P < 0.001	6.6	0.6	P < 0.001
Lewiecki 2019			N=3169	N=3176		N=3237	N=3256		N=3237	N=3256	
Extension of	24	12	16.6	5.5	P < 0.001	8.5	3.2	P < 0.001	7.3	2.3	P < 0.001
FRAME study	36	24	18.1	7.5	P < 0.001	9.4	4.2	P < 0.001	8.2	3.4	P < 0.001
Miyauchi 2019			N=205	N=190		N=205	N=200		N=205	N=200	
Subgroup	24	12	20.2	7.3	P < 0.001	7.9	2.9	P < 0.001	7.8	3.6	P < 0.001
analysis of			N=207	N=195		N=218	N=210		N=218	N=210	
FRAME Study	36	24	22.1	9.5	P < 0.001	8.7	4.5	P < 0.001	8.6	4.4	P < 0.001
**Group 1: Teriparatide to Dmab; Group 2: Teriparatide to ALN; Group 3: Teriparatide to minodronate**											
Niimi 2018		12	Group 1 N=100 4.3 ± 3.5; Group 2 N=100 1.3 ± 5.1	Group 3 N=100 0.5 ± 4.6	P < 0.01 Dmab vs Mino or ALN				Group 1 N=100 1.4 ± 3.4; Group 2 N=100 0.7 ± 4.6	Group 3 N=100 0.2 ± 4.6	P=0.16
**Group 1: Romosozumab to ALN; Group 2: only ALN**											
Saag 2017 *			N=2046	N=2047		N=2046	N=2047		N=2046	N=2047	
Extension of ARCH study	36	24	14.9	8.5	P < 0.001	7.0	3.6	P < 0.001	5.9	2.7	P < 0.001
Cosman 2020			N=1739	N=1726		N=1739	N=1726		N=1739	N=1726	
ARCH Study	24	12	15.5 ± 0.4	7.3 ± 0.3	P < 0.001	7.3 ± 0.2	3.5 ± 0.2	P < 0.001	6.1 ± 0.4	2.3 ± 0.3	P < 0.001
**Group 1: PTH to BisP; Group 2: PTH to placebo**											
Black 2005			N=12	N=7		N=12	N=7		N=12	N=7	
PATH study	24	12	12.1	4.0	P <0.05	4.0	0.0	P <0.05	4.0	1.0	P <0.05
**Group 1: Romosozumab to Dmab; Group 2: Romosozumab to placebo**											
Kendler 2019			N=16	N=19		N=16	N=19		N=16	N=19	
	24-36	0-12	2.5 ± 1.5	-9.1 ± 1.6		2.0 ± 1.3	-5.3 ± 2.0		1.3 ± 1.3	-4.3 ± 2.3	
**Group 1: ALN to Teriparatide; Group 2: treatment naive to Teriparatide**											
Fahrleitner-Pammer 2016			N=29	N=16							
		12	1.1	6.2	P=0.004						
		24	5.3	10.2	P=0.077						

**Group 1: BisP or AR to TPTD; Group 2: no trt to TPTD**																	
Middleton 2007			N=38	N=14				N=38	N=14								
		12	9.0	7.8			P=0.54	1.0	-0.3			P=0.36					
		18	9.8	6.1			P=0.30	2.8	1.3			P=0.44					
Obermayer-			N=134	N=84				N=134	N=84				N=134	N=84			
Pietsch 2008		6	3.5	5.8			P < 0.001	-0.3	0.5				-0.3	1.2			P < 0.05
EUROFORS		12	6.6	9.3			P < 0.001	0.6	1.8			P < 0.05	1.1	2.2			
Study		18	8.6	11.1			P < 0.01	0.6	2.7			P < 0.05	2.1	3.1			
		24	10.2	13.1			P < 0.01	2.3	3.8			P < 0.01	3.4	4.8			
**Group 1: AR to Teriparatide; Group 2: no change AR**																	
Gonnelli 2006			N=27	N=28				N=27	N=28				N=27	N=28			
		6	5.6 ± 6.7	1.2 ± 3.4			P<0.05	-2.1 ± 3.5	0.20 ± 2.9			NS	-1.8 ± 8.7	1.6 ± 3.1			NS
		12	7.1 ± 5.9	1.5 ± 4.3			P<0.05	-0.8 ± 2.7	1.2 ± 4.2			NS	2.6 ± 5.1	1.1 ± 3.8			NS
**Group 1: ALN to Romosozumab; Group 2: ALN to Teriparatide**																	
Langdhal 2017			N=206	N=209				N=206	N=209				N=206	N=209			
STRUCTURE		6	7.2 (6.6-7.8)	3.5 (2.9-4.0)			P<0.0001	2.3 (1.9-2.7)	-0.8 (-1.2 to -0.4)			P=0.0001	2.1 (1.6-2.7)	-1.1 (-1.6 to -0.5)			P=0.0003
study		12	9.8 (9.0-10.5)	5.4 (4.7– 6.1)			P<0.0001	2.9 (2.5-3.4)	-0.5 (-0.9 to -0.0)			P=0.0357	3.2 (2.6-3.8)	-0.2 (-0.8 to 0.4)			P=0.4566
**Group 1: RIS to Teriparatide; Group 2: ALN to Teriparatide**																	
Miller 2008			N=158	N=166				N=158	N=166								
		6	3.0	2.0				-1.2	-1.9			P=0.07					
		12	5.1	3.6			P < 0.05	-0.3	-1.7			P=0.07					
**Group 1: RIS to Teriparatide; Group 2: ALN to Teriparatide; Group 3: ETN to Teriparatide; Group 4: Non-BisP to Teriparatide**																	
Boonen 2008			Group 1	Group 2	Group 3	Group 4		Group 1	Group 2	Group 3	Group 4		Group 1	Group 2	Group	Group 4	
EUROFORS		6	N=59	N=107	N=30	N=49	P < 0.05	N=59	N=107	N=30	N=49		N=59	N=107	3	N=49	
study			2.3	3.0	5.8	4.0	ETN vs RIS, ALN	-1.6	-1.2	-0.7	-0.3	NS	-1.1	-1.8	N=30	-1.4	NS
															-0.9		
		12	5.6	5.4	8.8	5.3	P < 0.05 ETN vs RIS, ALN	-0.4	-0.6	1.1	-0.4	NS	0.2	-0.5	1.5	-0.3	NS
		18	7.7	7.8	11.6	8.2	P < 0.05 ETN vs NON-BPs	0.9	0.6	2.4	1.4	NS	1.6	1.3	3.8	2.3	P < 0.005 ALN vs ETI
		24	9.4	9.2	13.5	9.3	P < 0.05 ETN vs NON-BPs	2.9	2.1	3.7	1.8	NS	4.1	3.4	3.7	2.7	NS

Risk of refracture: switching from Anabolic to Antiresorptive, or viceversa

**First author, Year**	**Months from baseline**	**Months from switch**	**Site of fracture**	**Incidence of fracture**		
				**Group 1**	**Group 2**	**RR (95% CI)**
**Group 1: Romo to Dmab; Group 2: placebo to Dmab**						
Cosman 2016<br>*FRAME Study*	24	12	Vertebral fracture	21/3325 (0.6%)	84/3327 (2.5%)	0.25 (0.16 – 0.40)
			Non vertebral fracture	96/3589 (2.7%)	129/3591 (3.6%)	0.75 (0.57 – 0.97)
			Major non vertebral fracture	67/3589 (1.9%)	101/3591 (2.8%)	0.67 (0.49 – 0.91)
			Hip fracture	11/3589 (0.3%)	22/3591 (0.6%)	0.50 (0.24 – 1.04)
			Major osteoporotic fracture	68/3589 (1.9%)	110/3591 (3.1%)	0.62 (0.46 – 0.84)
Lewiecki 2019<br>*Extension of FRAME study*	36	24	Vertebral fracture	32/3325 (1.0%)	94/3327 (2.8%)	0.34 (0.23 to 0.51)
			Non vertebral fracture	139/3589 (3.9%)	176/3591 (4.9%)	0.79 (0.64 to 0.98)
			Major non vertebral fracture	100/3589 (2.8%)	138/3591 (3.8%)	0.73 (0.56 to 0.93)
			Hip fracture	18/3589 (0.5%)	31/3591 (0.9%)	0.58 (0.33 to 1.04)
			Major osteoporotic fracture	103/3589 (2.9%)	147/3591 (4.1%)	0.70 (0.55 to 0.90)
Miyuachi 2019<br>*Subgroup analysis of FRAME Study*	36	24	Vertebral fracture	4/237 (1.7%)	11/243 (4.5%)	0.37 (0.12 to 1.15)
			Non vertebral fracture	7/247 (2.8%)	15/245 (6.1%)	0.46 (0.19 to 1.12)
			Major non vertebral fracture	4/247 (1.6%)	7/245 (2.9%)	0.57 (0.17 to 1.91)
			Hip fracture	0/247 (0.0%)	2/245 (0.8%)	0.20 (0.01 to 4.11)
			Major osteoporotic fracture	5/247 (2.0%)	8/245 (3.3%)	0.62 (0.21 to 1.87)
**Group 1: Romosozumab to ALN; Group 2: only ALN**						
Cosman 2020<br>*Post hoc analysis of ARCH Study*	24	12	Non vertebral fracture	105/1739 (6.0%)	127/1726 (7.4%)	0.81 (0.63-1.05)*
			Hip fracture	25/1739 (1.4%)	42/1726 (2.4%)	0.60 (0.37-0.99)*
Saag 2017<br>*ARCH Study*	24	12	Vertebral fracture	127/2046 (6.2%)	243/2047 (11.9%)	0.52 (0.40-0.66)**
**Group 1: Teriparatide 20 µg to BPs; Group 2: Teriparatide 40 µg to BPs; Group 3: Placebo to BPs**						
Prince 2005		30	Non vertebral fracture	Group 1<br>30/436 (6.9%)	Group 2<br>22/412 (5.3%)<br>Group 3<br>38/414 (9.2%)	20 µg: 0.70 (0.43-1.13)***<br>40 µg: 0.54 (0.32-0.91)***
**Group 1: BisP or AR to Teriparatide; Group 2: placebo to Teriparatide**						
Obermayer-Pietsch 2008<br>*EUROFORS Study*		24	Any fracture	3/134 (2.2%)	5/84 (5.9%)	0.38 (0.09 to 1.53)

** adjusted for baseline BMD, age strata, and presence of severe vertebral fracture at baseline*
*** adjusted for age (<75 vs. ≥75 years), the presence or absence of severe vertebral fracture at baseline, and baseline bone mineral density T score at the total hip*
**** adjusted for duration of osteoporosis drug treatment*

Quality evaluation

Randomized controlled trials evaluated with the Risk of Bias tool

	Random sequence generation (selection bias)	Allocation concealment (selection bias)	Blinding of participants and personnel (performance bias)	Blinding of outcome assessment (detection bias)	Incomplete outcome data (attrition bias)	Selective reporting (reporting bias)	Other bias
ARCH (Saag 2017, Cosman 2020)	+	+	+	+	+	+	−
Black 2005	?	?	+	+	?	+	+
EUROFORS (Boonen 2008, Obermayer-Pietsch 2008)	?	?	+	+	+	+	−
Fahrleitner-Pammer 2016	?	?	+	+	?	+	+
FRAME (Lewiecki 2018, Cosman 2016)	+	+	+	+	+	+	+
Gonnelli 2006	?	?	+	+	+	+	?
Kendler 2019	?	?	+	+	+	+	+
Langdhal 2017	+	+	+	+	+	+	−
Leder 2015	+	+	+	+	+	+	+
Middleton 2007	−	?	?	+	?	+	?
Miller 2008	+	+	+	+	+	+	−
Miyauchi 2019	?	?	+	+	+	+	?
Niimi 2018	+	+	+	+	+	+	?
Prince 2005	?	?	+	+	?	+	−

Summary of Findings

Anabolic – Anti-resorptive

Romosozumab - Denosumab vs Placebo - Denosumab

Certainty assessment							№ of patients		Effect		Certainty	Importance
**№ of studies**	**Study design**	**Risk of bias**	**Inconsistency**	**Indirectness**	**Imprecision**	**Other considerations**	**2(AN)->1(AR)**	**0->1(AR)**	**Relative (95% CI)**	**Absolute (95% CI)**		
**Fracture at 12 months from switch - Vertebral fracture**												
1	RCTs	not serious	not serious	not serious	not serious	none	21/3325 (0.6%)	84/3327 (2.5%)	**RR 0.25** (0.16 to 0.39)	**19 fewer per 1.000** (from 21 fewer to 15 fewer)	⊕⊕⊕⊕ HIGH	CRITICAL
**Fracture at 12 months from switch - Nonvertebral fracture**												
1	RCTs	not serious	not serious	not serious	not serious	none	96/3589 (2.7%)	129/3591 (3.6%)	**RR 0.75** (0.57 to 0.99)	**9 fewer per 1.000** (from 15 fewer to 0 fewer)	⊕⊕⊕⊕ HIGH	CRITICAL
**Fracture at 12 months from switch - Major nonvertebral fracture**												
1	RCTs	not serious	not serious	not serious	not serious	none	67/3589 (1.9%)	101/3591 (2.8%)	**RR 0.67** (0.49 to 0.92)	**9 fewer per 1.000** (from 14 fewer to 2 fewer)	⊕⊕⊕⊕ HIGH	CRITICAL
**Fracture at 12 months from switch - Hip fracture**												
1	RCTs	not serious	not serious	not serious	serious [a]	none	11/3589 (0.3%)	22/3591 (0.6%)	**RR 0.50** (0.24 to 1.04)	**3 fewer per 1.000** (from 5 fewer to 0 fewer)	⊕⊕⊕◯ MODERATE	CRITICAL
**Fracture at 12 months from switch - Major osteoporotic fracture**												

Certainty assessment							№ of patients		Effect		Certainty	Importance
№ of studies	Study design	Risk of bias	Inconsistency	Indirectness	Imprecision	Other considerations	2(AN)->1(AR)	0->1(AR)	Relative (95% CI)	Absolute (95% CI)		
1	RCTs	not serious	not serious	not serious	not serious	none	68/3589 (1.9%)	110/3591 (3.1%)	**RR 0.62** (0.46 to 0.84)	**12 fewer per 1.000** (from 17 fewer to 5 fewer)	⊕⊕⊕⊕ HIGH	CRITICAL
**Fracture at 24 months from switch - Vertebral fracture**												
2	RCTs	not serious	not serious	not serious	not serious	none	36/3562 (1.0%)	109/3570 (3.1%)	**RR 0.37** (0.12 to 1.15)<br><br>**RR 0.34** (0.23 to 0.51)	**29 fewer per 1.000** (from 40 fewer to 7 fewer)<br><br>**19 fewer per 1.000** (from 22 fewer to 14 fewer)	⊕⊕⊕⊕ HIGH	CRITICAL
**Fracture at 24 months from switch - Nonvertebral fracture**												
2	RCTs	not serious	not serious	not serious	serious [a]	none	146/3572 (4.1%)	191/3836 (5.0%)	**RR 0.46** (0.19 to 1.12)<br><br>**RR 0.79** (0.64 to 0.98)	**33 fewer per 1.000** (from 50 fewer to 7 more)<br><br>**10 fewer per 1.000** (from 18 fewer to 1 fewer)	⊕⊕⊕◯ MODERATE	CRITICAL
**Fracture at 24 months from switch - Major nonvertebral fracture**												
2	RCTs	not serious	not serious	not serious	not serious	none	104/3572 (2.9%)	145/3836 (3.8%)	**RR 0.57** (0.17 to 1.91)<br><br>**RR 0.73** (0.56 to 0.93)	**12 fewer per 1.000** (from 24 fewer to 26 more)<br><br>**10 fewer per 1.000** (from 17 fewer to 3 fewer)	⊕⊕⊕⊕ HIGH	CRITICAL

Fracture at 24 months from switch - Hip fracture

Certainty assessment							№ of patients		Effect		Certainty	Importance
№ of studies	Study design	Risk of bias	Inconsistency	Indirectness	Imprecision	Other considerations	2(AN)->1(AR)	0->1(AR)	Relative (95% CI)	Absolute (95% CI)		
2	RCTs	not serious	not serious	not serious	not serious	none	18/3572 (0.5%)	33/3836 (0.9%)	**RR 0.20** (0.01 to 4.11)<br><br>**RR 0.58** (0.33 to 1.04)	**7 fewer per 1.000** (from 8 fewer to 25 more)<br><br>**4 fewer per 1.000** (from 6 fewer to 0 fewer)	⊕⊕⊕⊕ HIGH	CRITICAL
**Fracture at 24 months from switch - Major osteoporotic fracture**												
2	RCTs	not serious	not serious	not serious	not serious	none	108/3572 (3.0%)	155/3836 (4.0%)	**RR 0.62** (0.21 to 1.87)<br><br>**RR 0.70** (0.55 to 0.90)	**12 fewer per 1.000** (from 26 fewer to 28 more)<br><br>**12 fewer per 1.000** (from 18 fewer to 4 fewer)	⊕⊕⊕⊕ HIGH	CRITICAL
**Fracture at 30 months from switch**												
1	RCTs	serious [b]	not serious	not serious	not serious	none	52/848 (6.1%)	76/828 (9.2%)	**RR 0.62** (0.43 to 0.89)	**35 fewer per 1.000** (from 52 fewer to 10 fewer)	⊕⊕⊕◯ MODERATE	CRITICAL

Romosozumab - Alendronate vs Only alendronate

Certainty assessment							№ of patients		Effect		Certainty	Importance
**№ of studies**	**Study design**	**Risk of bias**	**Inconsistency**	**Indirectness**	**Imprecision**	**Other considerations**	**2(AN)->1(AR)**	**0->1(AR)**	**Relative (95% CI)**	**Absolute (95% CI)**		
**Fracture at 12 months from switch - Nonvertebral fracture**												
1	RCTs	not serious	not serious	not serious	serious [a]	none	105/1739 (6.0%)	127/1726 (7.4%)	**RR 0.81** (0.63 to 1.04)	**14 fewer per 1.000** (from 27 fewer to 3 more)	⊕⊕⊕◯ MODERATE	CRITICAL
**Fracture at 12 months from switch - Hip fracture**												
1	RCTs	not serious	not serious	not serious	not serious	none	25/1739 (1.4%)	42/1726 (2.4%)	**RR 0.60** (0.37 to 0.97)	**10 fewer per 1.000** (from 15 fewer to 1 fewer)	⊕⊕⊕⊕ HIGH	CRITICAL
**Fracture at 12 months from switch - Vertebral fracture**												
1	RCTs	not serious	not serious	not serious	not serious	none	127/2046 (6.2%)	243/2047 (11.9%)	**RR 0.52** (0.40 to 0.68)	**57 fewer per 1.000** (from 71 fewer to 38 fewer)	⊕⊕⊕⊕ HIGH	CRITICAL

CI: Confidence interval; **RR:** Risk ratio

Explanations

a. Confidence intervals crossed the line of no difference with plausible effects in favor to the experimental group.

b. UNCLEAR Risk of Bias for the Random sequence generation, the allocation concealment and the incomplete outcome; HIGH Risk of Bias for Other Bias

Anti-resorptive – Anabolic

Anti-resorptive - Teriparatide vs Placebo – Teriparatide

Certainty assessment							№ of patients		Effect		Certainty	Importance
№ of studies	Study design	Risk of bias	Inconsistency	Indirectness	Imprecision	Other considerations	1(AR)->2(AB)	1(AR)->2(AB)	Relative (95% CI)	Absolute (95% CI)		
**Fracture 24 months from switch – Any**												
1	RCTs	serious [a]	not serious	not serious	serious [b]	none	3/134 (2.2%)	5/84 (6.0%)	**RR 0.38** (0.09 to 1.53)	**37 fewer per 1.000** (from 54 fewer to 32 more)	⨁⨁◯◯ LOW	CRITICAL

CI: Confidence interval; **RR:** Risk ratio

Explanations

a. UNCLEAR Risk of Bias for the Random sequence generation and the allocation concealment; HIGH Risk of Bias for Other Bias

b. Confidence intervals crossed the line of no difference with plausible effects in favor to the experimental group.

CQ 5

Might it be advisable to discontinue drugs aimed at reducing the risk of adverse events in patients at high risk of (re)fracture?

Search strategy

MEDLINE

Up to 25 November 2020

#1:

(((wrist* or colles or radius or articulatio radiocarpea or carpus or carpal or radiocarp* or radial or forearm* or humerus or metacarp* or barton or monteggi* or ulna or ulnar or upper limb* or hip or hips or trochanteric or intertrochanteric or subtrochanteric or femoral neck or femur neck or spine or spinal or vertebra or vertebral or vertebrae or lumbar or shoulder* or glenohumeral or humeroscapular or scapulo humeral or proximal humeral) adj3 fractur*) or (exp hip fractures/ or spinal fractures/ or shoulder fractures/ or osteoporotic fractures/ or exp radius fractures/) or (fractures, bone/ and (exp wrist joint/ or exp spine/ or shoulder/ or shoulder joint/ or hip/))) and (exp osteoporosis/ or (osteoporo* or bone loss*))

#2:

“fragility fracture”[ti] OR “fragility fractures”[ti] OR “low energy fracture”[ti] OR “low energy fractures”[ti] OR “low-energy fracture”[ti] OR “low-energy fractures”[ti] OR “low trauma fracture”[ti] OR “low trauma fractures”[ti] OR “low-trauma fracture”[ti] OR “low-trauma fractures”[ti] OR “low energy trauma”[ti] OR “low-energy trauma”[ti] OR “low level trauma”[ti] OR “low-level trauma”[ti] OR “minor trauma fracture”[ti] OR “minor trauma fractures”[ti] OR “minor-trauma fracture”[ti] OR “minor-trauma fractures”[ti] OR “minor fracture”[ti] OR “minor fractures”[ti] OR “minor-fracture”[ti] OR “minor-fractures”[ti] OR “osteoporotic fracture”[ti] OR “osteoporotic fractures”[ti]

#3: #1 OR #2

#4:

exp bone density conservation agents/ or exp diphosphonates/ or exp calcitonin/ or exp selective estrogen receptor modulators/ or exp raloxifene hydrochloride/ or exp teriparatide/ or (exp antibodies, monoclonal/ and exp rank ligand/) or (aclasta or actonel or alend or alendro* or alovell or amgiva or aminodron* or aminobutane* or aminohexane* or aminohydroxy* or aminomux or aminopropane* or aminopropylidene* or aredia or aredronet or arendal or atelvia or belfosdil or benet or bifemelan or bifosa or binosto or bisphonal or bisphosphon* or bonapex or bondenza or bondronat* or bonefos

or boniva or bonmax or bonviva or butedron* or calcitar or calciton* or calcitrin or cangrelor or celvista or cibalcin or cimadron* or clodron* or coldron* or cycloheptylaminomethylenebis or defixal or denosumab or dequest or destara or diadronel or dichlorometh* or didronal or didronat* or didronel or difosfonal or difosfen or dinol or diphos or diphosphon* or dronal or dronate or editron* or ehdp or endronax or ethane* or ethylenehydroxy* or ethylidenebisphosphon* or etibon or etidron* or eucalen or evista or fixopan or forsteo or forteo or fosalan or fosamax or fosmin or fosval or hedp or hexane* or hydroxyeth* or hydroxyhex* or hydroxyl* or iasibon or ibandron* or incadron* or kengreal or kengrexal or keoxifene or lidadronate or lodronat* or loxar or loxifen or marvil or maxibone or mebonat or medron* or medrotec or methane* or methanon* or methylene* or minodron* or neobon or neridron* or nerixia or olpadron* or oncalst or onclast or optinate or optruma or orazol or osdron or osdronat or oseotenk or osficar or oslene or ossiten or ostac or osteof* or osteopam or osteopor or osteosan or osteotop or osteovan or osticalcin or pamidronate or pamisol or panolin or parathar or parathormone* or parathyroid hormone* or porosal or prolia or propane* or propylidenediphosphon* or raloxifene or raxeto or reclast or ribastamin or risedron* or serm or serms or skelid or staporos or teiroc or teriparatide or thyreocalciton* or thyrocalciton* or tiludron* or tibolene or turpinal or voroste or xgeva or xidifon or xidiphone or xydiphon* or zoledron* or zomera or zometa or abaloparatide or “strontium ranelate” or bazedoxifene) or (bone resorpti* adj3 inhibitor*) or ((estrogen or oestrogen) adj3 receptor modulator*) or ((anti-resorpti* or anti-osteopor* or bone density) adj3 (drug* or agent* or medicin* or medication* or therap* or treatment*))

#5:

bisphosphonates[tiab] OR “etidronic acid”[tiab] OR “clodronic acid”[tiab] OR “pamidronic acid”[tiab] OR “alendronic acid”[tiab] OR “tiludronic acid”[tiab] OR “ibandronic acid”[tiab] OR “risedronic acid”[tiab] OR “zoledronic acid”[tiab] OR alendronate[tiab] OR risedronate[tiab] OR zoledronate[tiab] OR ibandronate[tiab] OR abaloparatide[tiab] OR teriparatide[tiab] OR denosumab[tiab] OR pamidronate[tiab] OR “strontium ranelate”[tiab] OR “selective estrogen receptor modulators”[tiab] OR SERM[tiab] OR bazedoxifene[tiab] OR raloxifene[tiab] OR ((treatment[ti] OR treated[ti] OR treat*[ti] OR untreated[ti] OR medication[ti] OR medications[ti] OR drug[ti] OR drugs[ti] OR therapy[ti] OR therapeutic[ti] OR "Therapeutics"[Mesh] OR antifracturative[ti]) AND (osteoporosis[ti] OR osteoporotic[ti] OR osteop*[ti] OR fragility[ti]))

#6: #4 OR #5

#7:

adherence[ti] OR adherent[ti] OR discontinuation[ti] OR compliance[ti] OR persistence[ti] OR cyclic[ti] OR intermittent[ti] OR continuous[ti] OR continuity[ti] OR adheren*[ti] OR discontin*[ti] discontinuous [ti] OR complian*[ti] OR persisten*[ti] OR intermitten*[ti] OR continu*[ti] OR

"Medication Adherence"[Mesh] OR "Treatment Adherence and Compliance"[Mesh] OR “non-compliance”[ti] OR “non compliance”[ti] OR noncompliance[ti] OR nonadherence[ti] OR “non-adherence”[ti] OR “non adherence”[ti] OR nonadherent[ti] OR dropout[ti] OR dropouts[ti] OR interruption[ti] OR deprescribing[ti] OR deprescription[ti] OR deprescriptions[ti] OR cyclical[ti] OR “treatment cessation”[ti] OR intermit[ti] OR suspension[ti] OR “drug holiday”[ti]

#8: #6 AND #7

#9: #3 AND #8; Filters: Humans

EMBASE

Up to 25 November 2020

#1:

(wrist*:ti OR colles:ti OR radius:ti OR “articulatio radiocarpea”:ti OR carpus:ti OR carpal:ti OR radiocarp*:ti OR radial:ti OR forearm*:ti OR humerus:ti OR metacarp*:ti OR barton:ti OR monteggi*:ti OR ulna:ti OR ulnar:ti OR limb*:ti OR hip:ti OR hips:ti OR trochanteric:ti OR intertrochanteric:ti OR subtrochanteric:ti OR “femoral neck”:ti OR “femur neck”:ti OR spine:ti OR spinal:ti OR vertebra:ti OR vertebral:ti OR vertebrae:ti OR lumbar:ti OR shoulder*:ti OR glenohumeral:ti OR humeroscapular:ti OR “scapulo humeral”:ti OR “proximal humeral”:ti or “hip fracture”:ti OR “spinal fracture”:ti OR “shoulder fracture”:ti OR “osteoporotic fracture”:ti OR “radius fracture”:ti OR “wrist joint”:ti OR spine:ti OR shoulder:ti OR “shoulder joint”:ti OR hip:ti) AND (fragility:ti OR osteoporosis:ti OR osteoporotic:ti)

#2: 'fragility fracture'/exp

#3: 'low energy fracture'/exp

#4: 'low trauma fracture'/exp

#5: 'low energy trauma'/exp

#6:

“fragility fracture”:ti OR “fragility fractures”:ti OR “low energy fracture”:ti OR “low energy fractures”:ti OR “low-energy fracture”:ti OR “low-energy fractures”:ti OR “low trauma fracture”:ti OR “low trauma fractures”:ti OR “low-trauma fracture”:ti OR “low-trauma fractures”:ti OR “low energy trauma”:ti OR “low-energy trauma”:ti OR “low level trauma”:ti OR “low-level trauma”:ti OR “minor trauma fracture”:ti OR “minor trauma fractures”:ti OR “minor-trauma fracture”:ti OR “minor-trauma fractures”:ti OR “minor fracture”:ti OR “minor fractures”:ti OR “minor-fracture”:ti OR “minor-fractures”:ti OR “osteoporotic fracture”:ti OR “osteoporotic fractures”:ti

#7: #1 OR #2 OR #3 OR #4 OR #5 OR #6

#8:

'bone density conservation agent'/exp OR 'osteoporosis'/exp/dm_dt OR 'bisphosphonic acid derivative'/exp OR 'calcitonin'/exp OR 'selective estrogen receptor modulator'/exp OR 'raloxifene'/exp OR 'denosumab'/exp OR 'parathyroid hormone[1-34]'/exp OR ('osteoclast differentiation factor'/exp AND 'monoclonal antibody'/exp) OR abaloparatide:ab,ti OR (strontium ranelate):ab,ti OR bazedoxifene:ab,ti OR aclasta:ab,ti OR actonel:ab,ti OR alend:ab,ti OR alendro*:ab,ti OR alovell:ab,ti OR amgiva:ab,ti OR aminodron*:ab,ti OR aminobutane*:ab,ti OR aminohexane*:ab,ti OR aminohydroxy*:ab,ti OR aminomux:ab,ti OR aminopropane*:ab,ti OR aminopropylidene*:ab,ti OR aredia:ab,ti OR aredronet:ab,ti OR arendal:ab,ti OR atelvia:ab,ti OR belfosdil:ab,ti OR benet:ab,ti OR bifemelan:ab,ti OR bifosa:ab,ti OR binosto:ab,ti OR bisphonal:ab,ti OR bisphosphon*:ab,ti OR bonapex:ab,ti OR bondenza:ab,ti OR bondronat*:ab,ti OR bonefos:ab,ti OR boniva:ab,ti OR bonmax:ab,ti OR bonviva:ab,ti OR butedron*:ab,ti OR calcitar:ab,ti OR calciton*:ab,ti OR calcitrin:ab,ti OR cangrelor:ab,ti OR celvista:ab,ti OR cibalcin:ab,ti OR cimadron*:ab,ti OR clodron*:ab,ti OR coldron*:ab,ti OR cycloheptylaminomethylenebis:ab,ti OR defixal:ab,ti OR denosumab:ab,ti OR dequest:ab,ti OR destara:ab,ti OR diadronel:ab,ti OR dichlorometh*:ab,ti OR didronal:ab,ti OR didronat*:ab,ti OR didronel:ab,ti OR difosfonal:ab,ti OR difosfen:ab,ti OR dinol:ab,ti OR diphos:ab,ti OR diphosphon*:ab,ti OR dronal:ab,ti OR dronate:ab,ti OR editron*:ab,ti OR ehdp:ab,ti OR endronax:ab,ti OR ethane*:ab,ti OR ethylenehydroxy*:ab,ti OR ethylidenebisphosphon*:ab,ti OR etibon:ab,ti OR etidron*:ab,ti OR eucalen:ab,ti OR evista:ab,ti OR fixopan:ab,ti OR forsteo:ab,ti OR forteo:ab,ti OR fosalan:ab,ti OR fosamax:ab,ti OR fosmin:ab,ti OR fosval:ab,ti OR hedp:ab,ti OR hexane*:ab,ti OR hydroxyeth*:ab,ti OR hydroxyhex*:ab,ti OR hydroxyl*:ab,ti OR iasibon:ab,ti OR ibandron*:ab,ti OR incadron*:ab,ti OR kengreal:ab,ti OR kengrexal:ab,ti OR keoxifene:ab,ti OR lidadronate:ab,ti OR lodronat*:ab,ti OR loxar:ab,ti OR loxifen:ab,ti OR marvil:ab,ti OR maxibone:ab,ti OR mebonat:ab,ti OR medron*:ab,ti OR medrotec:ab,ti OR methane*:ab,ti OR methanon*:ab,ti OR methylene*:ab,ti OR minodron*:ab,ti OR neobon:ab,ti OR neridron*:ab,ti OR nerixia:ab,ti OR olpadron*:ab,ti OR oncalst:ab,ti OR onclast:ab,ti OR optinate:ab,ti OR optruma:ab,ti OR orazol:ab,ti OR osdron:ab,ti OR osdronat:ab,ti OR oseotenk:ab,ti OR osficar:ab,ti OR oslene:ab,ti OR ossiten:ab,ti OR ostac:ab,ti OR osteof*:ab,ti OR osteopam:ab,ti OR osteopor:ab,ti OR osteosan:ab,ti OR osteotop:ab,ti OR osteovan:ab,ti OR osticalcin:ab,ti OR pamidronate:ab,ti OR pamisol:ab,ti OR panolin:ab,ti OR parathar:ab,ti OR parathormone*:ab,ti OR 'parathyroid hormone':ab,ti OR 'parathyroid hormones':ab,ti OR porosal:ab,ti OR prolia:ab,ti OR propane*:ab,ti OR propylidenediphosphon*:ab,ti OR raloxifene:ab,ti OR raxeto:ab,ti OR reclast:ab,ti OR ribastamin:ab,ti OR risedron*:ab,ti OR serm:ab,ti OR serms:ab,ti OR skelid:ab,ti OR staporos:ab,ti OR teiroc:ab,ti OR teriparatide:ab,ti OR

thyreocalciton*:ab,ti OR thyrocalciton* OR tiludron*:ab,ti OR tibolene:ab,ti OR turpinal:ab,ti OR voroste:ab,ti OR xgeva:ab,ti OR xidifon:ab,ti OR xidiphone:ab,ti OR xydiphon*:ab,ti OR zoledron*:ab,ti OR zomera:ab,ti OR zometa:ab,ti OR (bone NEAR/3 resorpti* NEAR/3 inhibitor*):ab,ti OR ((estrogen OR oestrogen) NEAR/3 receptor* NEAR/3 modulator*):ab,ti OR (('anti-resorption' OR 'anti-osteoporosis' OR 'anti-osteoporotic' OR 'bone density' OR osteopor* OR decalcificat* OR fragility) NEAR/3 (drug* OR agent* OR medicin* OR medication* OR therap* OR treatment*)):ab,ti

#9: ‘patient compliance’

#10: ‘adherence’

#11: ‘patient care’

#12: ‘persistence’

#13:

adherence:ti OR adherent:ti OR discontinuation:ti OR compliance:ti OR persistence:ti OR cyclic:ti OR intermittent:ti OR continuous:ti OR continuity:ti OR adheren*:ti OR discontin*:ti discontinuous:ti OR complian*:ti OR persisten*:ti OR intermitten*:ti OR continu*:ti OR “non-compliance”:ti OR “non compliance”:ti OR noncompliance:ti OR nonadherence:ti OR “non-adherence”:ti OR “non adherence”:ti OR nonadherent:ti OR dropout:ti OR dropouts:ti OR interruption:ti OR deprescribing:ti OR deprescription:ti OR deprescriptions:ti OR cyclical:ti OR “treatment cessation”:ti OR intermit:ti OR suspension:ti OR ‘drug holiday’:ti

#14: #9 OR #10 OR #11 OR #12 OR #13

#15: #8 AND #14

#16:

#15 NOT (cancer*:ti OR tumor*:ti OR tumour*:ti OR malignan*:ti OR neoplas*:ti OR carcinoma*:ti) NOT [medline]/lim NOT ([animals]/lim)

COCHRANE

Up to 25 November 2020

#1:

((wrist* or colle* or radius or "articulatio radiocarpea" or carpus or carpal or radiocarp* or radial or forearm* or humerus or metacarp* or barton or monteggi* or ulna or ulnar or "upper limb" or "upper limbs" or hip or hips or trochanteric or intertrochanteric or subtrochanteric or "femoral neck" or "femur neck" or spine or spinal or vertebra* or lumbar or shoulder* or glenohumeral or humeroscapular or "scapulo humeral" or "proximal humeral") near/3 fractur*):ti,ab or [mh "hip

fractures"] or [mh "spinal fractures"] or [mh "shoulder fractures"] or [mh "osteoporotic fractures"] or [mh "radius fractures"] or ([mh "bone fractures"] and ([mh "wrist joint"] or [mh spine] or [mh shoulder] or [mh "shoulder joint"] or [mh hip])) and ([mh osteoporosis] or (osteoporo* or "bone loss" OR fragility):ti,ab)

#2: MeSH descriptor: [Osteoporotic Fractures] explode all trees

#3: MeSH descriptor: [Fractures, Spontaneous] explode all trees

#4:

(fragility fracture):ti OR (fragility fractures):ti OR (low energy fracture):ti OR (low energy fractures):ti OR (low-energy fracture):ti OR (low-energy fractures):ti OR (low trauma fracture):ti OR (low trauma fractures):ti OR (low-trauma fracture):ti OR (low-trauma fractures):ti OR (low energy trauma):ti OR (low-energy trauma):ti OR (low level trauma):ti OR (low-level trauma):ti OR (minor trauma fracture):ti OR (minor trauma fractures):ti OR (minor-trauma fracture):ti OR (minor-trauma fractures):ti OR (minor fracture):ti OR (minor fractures):ti OR (minor-fracture):ti OR (minor-fractures):ti OR (osteoporotic fracture):ti OR (osteoporotic fractures):ti OR (pathologic fracture):ti OR (pathological fractures):ti

#5: #1 OR #2 OR #3 OR #4

#6:

[mh "bone density conservation agents"] or [mh osteoporosis/DT] or [mh diphosphonates] or [mh calcitonin] or [mh "selective estrogen receptor modulators"] or [mh "raloxifene hydrochloride"] or [mh teriparatide] or ([mh "antibodies, monoclonal"] and [mh "rank ligand"]) or (abaloparatide OR “strontium ranelate” OR bazedoxifene OR aclasta or actonel or alend or alendro* or alovell or amgiva or aminodron* or aminobutane* or aminohexane* or aminohydroxy* or aminomux or aminopropane* or aminopropylidene* or aredia or aredronet or arendal or atelvia or belfosdil or benet or bifemelan or bifosa or binosto or bisphonal or bisphosphon* or bonapex or bondenza or bondronat* or bonefos or boniva or bonmax or bonviva or butedron* or calcitar or calciton* or calcitrin or cangrelor or celvista or cibalcin or cimadron* or clodron* or coldron* or cycloheptylaminomethylenebis or defixal or denosumab or dequest or destara or diadronel or dichlorometh* or didronal or didronat* or didronel or difosfonal or difosfen or dinol or diphos or diphosphon* or dronal or dronate or editron* or ehdp or endronax or ethane* or ethylenehydroxy* or ethylidenebisphosphon* or etibon or etidron* or eucalen or evista or fixopan or forsteo or forteo or fosalan or fosamax or fosmin or fosval or hedp or hexane* or hydroxyeth* or hydroxyhex* or hydroxyl* or iasibon or ibandron* or incadron* or kengreal or kengrexal or keoxifene or lidadronate or lodronat* or loxar or loxifen or marvil or maxibone or mebonat or medron* or medrotec or methane* or methanon* or methylene* or minodron* or neobon or neridron* or nerixia or olpadron*

or oncalst or onclast or optinate or optruma or orazol or osdron or osdronat or oseotenk or osficar or oslene or ossiten or ostac or osteof* or osteopam or osteopor or osteosan or osteotop or osteovan or osticalcin or pamidronate or pamisol or panolin or parathar or parathormone* or "parathyroid hormone*" or porosal or prolia or propane* or propylidenediphosphon* or raloxifene or raxeto or reclast or ribastamin or risedron* or serm or serms or skelid or staporos or teiroc or teriparetide or thyreocalictron* or thyrocalciton* or tiludron* or tibolene or turpinal or voroste or xgeva or xidifon or xidiphone or xydiphon* or zoledron* or zomera or zometa):ti,ab or (bone resorpti* near/3 inhibitor*):ti,ab or ((estrogen or oestrogen) near/3 "receptor modulator*"):ab,ti or ((anti-resorpti* or anti-osteopor* or bone density or osteoporosis) near/3 (drug* or agent* or medicin* or medication* or therap* or treatment*)):ti,ab

#7:

(adherence):ti OR (adherent):ti OR (discontinuation):ti OR (compliance):ti OR (persistence):ti OR (cyclic):ti OR (intermittent):ti OR (continuous):ti OR (continuity):ti OR (adheren*):ti OR (discontin*):ti (discontinuous):ti OR (complian*):ti OR (persisten*):ti OR (intermitten*):ti OR (continu*):ti OR “non-compliance”:ti OR “non compliance”:ti OR noncompliance:ti OR nonadherence:ti OR “non-adherence”:ti OR “non adherence”:ti OR nonadherent:ti OR dropout:ti OR dropouts:ti OR interruption:ti OR deprescribing:ti OR deprescription:ti OR deprescriptions:ti OR cyclical:ti OR “treatment cessation”:ti OR intermit:ti OR suspension:ti OR (“non-compliance”):ti OR (“non compliance”):ti OR (noncompliance):ti OR (nonadherence):ti OR (“non-adherence”):ti OR (“non adherence”):ti OR (nonadherent):ti OR (dropout):ti OR (dropouts):ti OR (interruption):ti OR (deprescribing):ti OR (deprescription):ti OR (deprescriptions):ti OR (cyclical):ti OR (“treatment cessation”):ti OR (intermit):ti OR (suspension):ti

#8: MeSH descriptor: [Treatment Adherence and Compliance] explode all trees

#9: #7 OR #8

#10: #6 AND #9

#11: #5 AND #10

Flow chart

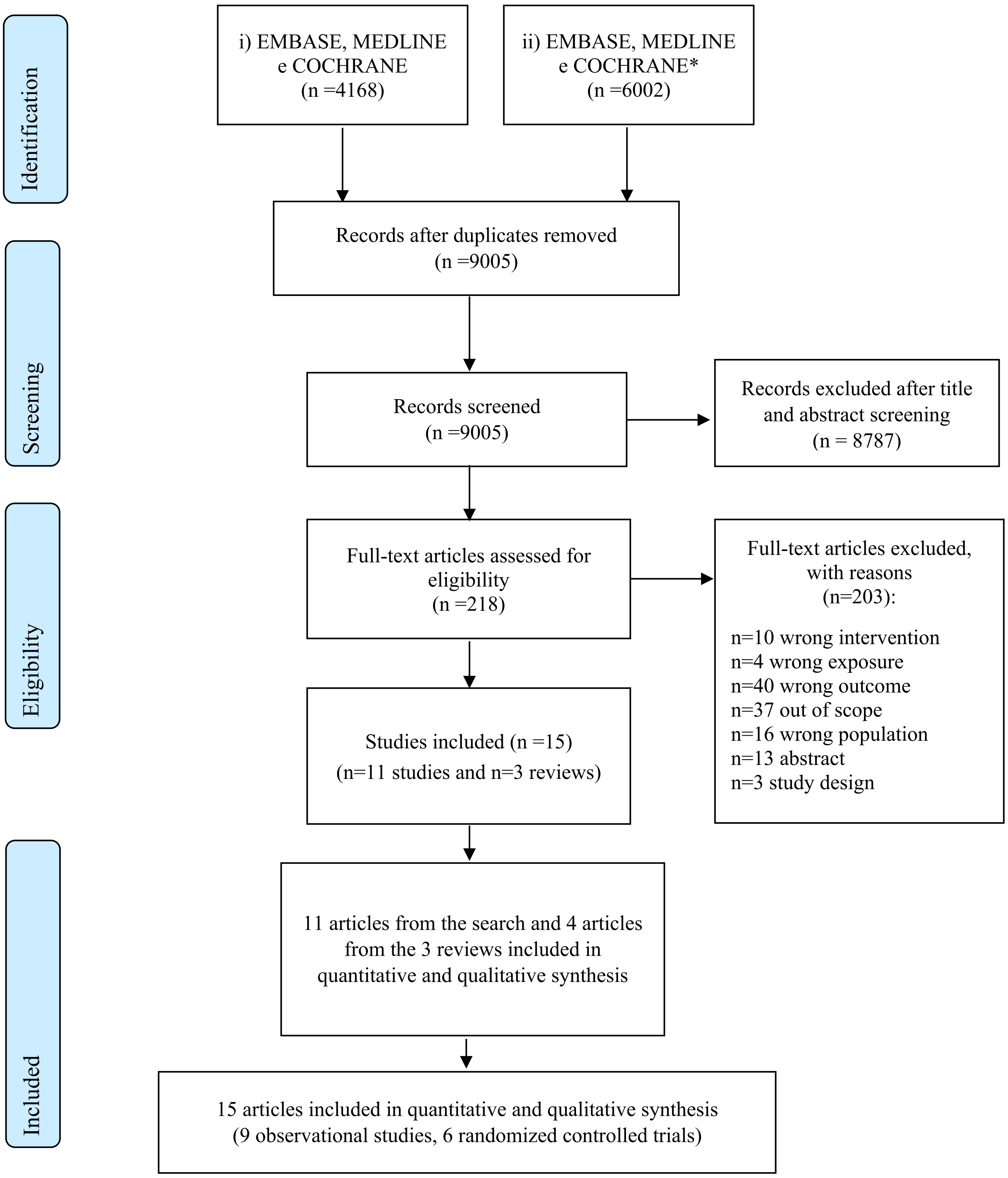
Identification
Screening
Eligibility
Included
i) EMBASE, MEDLINE e COCHRANE (n =4168)
ii) EMBASE, MEDLINE e COCHRANE* (n =6002)
Records after duplicates removed (n =9005)
Records screened (n =9005)
Records excluded after title and abstract screening (n = 8787)
Full-text articles assessed for eligibility (n =218)
Full-text articles excluded, with reasons (n=203):
n=10 wrong intervention
n=4 wrong exposure
n=40 wrong outcome
n=37 out of scope
n=16 wrong population
n=13 abstract
n=3 study design
Studies included (n =15) (n=11 studies and n=3 reviews)
11 articles from the search and 4 articles from the 3 reviews included in quantitative and qualitative synthesis
15 articles included in quantitative and qualitative synthesis (9 observational studies, 6 randomized controlled trials)

Results

Refracture among patients with MPR ≥ 80% compared to non-adherent subjects (MPR <80%)

Study or Subgroup	Experimental Events	Experimental Total	Control Events	Control Total	Weight	Risk Ratio M-H, Random, 95% CI
**1.1.1 Vertebral fracture**						
1) Keshishian 2017_Pelvis/Femur/Hip_Various	192	14112	164	9022	98.9%	0.75 [0.61, 0.92]
4) Sheehy 2009_Vertebral_Bisphosphonates	2	975	2	362	1.1%	0.37 [0.05, 2.63]
**Subtotal (95% CI)**		**15087**		**9384**	**100.0%**	**0.74 [0.60, 0.91]**
Total events	194		166			
Heterogeneity: Tau² = 0.00; Chi² = 0.49, df = 1 (P = 0.48); I² = 0%						
Test for overall effect: Z = 2.84 (P = 0.005)						
**1.1.2 Nonvertebral fracture**						
1) Keshishian 2017_Pelvis/Femur/Hip_Various	101	14112	85	9022	36.4%	0.76 [0.57, 1.01]
4) Lin 2011_Vertebral/Hip_Alendronate	46	3412	251	5524	36.1%	0.30 [0.22, 0.41]
4) Sheehy 2009_Vertebral_Bisphosphonates	12	975	15	362	27.5%	0.30 [0.14, 0.63]
**Subtotal (95% CI)**		**18499**		**14908**	**100.0%**	**0.42 [0.20, 0.87]**
Total events	159		351			
Heterogeneity: Tau² = 0.36; Chi² = 20.86, df = 2 (P < 0.0001); I² = 90%						
Test for overall effect: Z = 2.34 (P = 0.02)						
**1.1.3 Refracture**						
3) Soong 2013_Hip/Spine_Alendronate	1731	9344	197	871	100.0%	0.82 [0.72, 0.93]
**Subtotal (95% CI)**		**9344**		**871**	**100.0%**	**0.82 [0.72, 0.93]**
Total events	1731		197			
Heterogeneity: Not applicable						
Test for overall effect: Z = 3.01 (P = 0.003)						

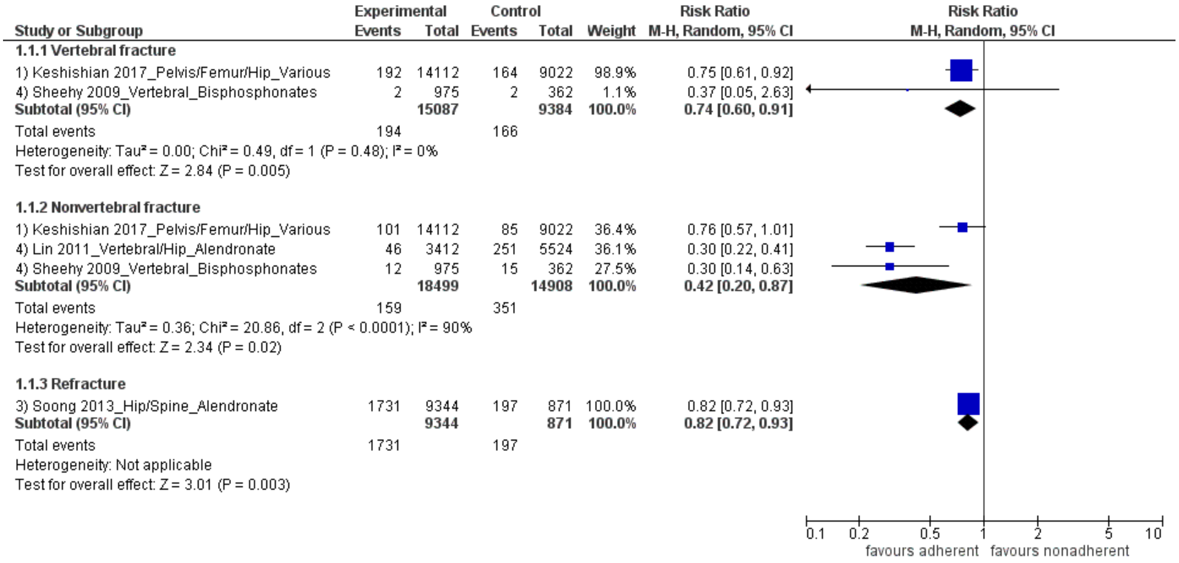

Refracture among adherent patients to anti-osteoporotic treatment for more than 12 months compared to non-adherent subjects

Study or Subgroup	Experimental Events	Experimental Total	Control Events	Control Total	Weight	Risk Ratio M-H, Fixed, 95% CI
**2.1.1 Nonvertebral fracture**						
2) Chan 2016_Fracture_Teriparatide	119	1569	286	3055	100.0%	0.81 [0.66, 0.99]
**Subtotal (95% CI)**		**1569**		**3055**	**100.0%**	**0.81 [0.66, 0.99]**
Total events	119		286			
Heterogeneity: Not applicable						
Test for overall effect: Z = 2.01 (P = 0.04)						

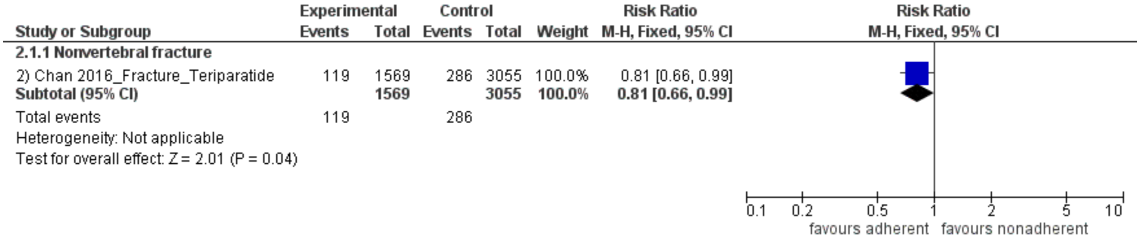

Mortality rate among patients with MPR ≥ 80% compared to non-adherent subjects (MPR <80%)

Study or Subgroup	Experimental Events	Experimental Total	Control Events	Control Total	Weight	Risk Ratio M-H, Random, 95% CI
**1.2.1 Mortality**						
3) Yu 2019_Hip_Various	51	446	444	1646	64.9%	0.42 [0.32, 0.56]
4) Chen 2017_Vertebral_Various	31	184	32	110	35.1%	0.58 [0.38, 0.89]
**Subtotal (95% CI)**		**630**		**1756**	**100.0%**	**0.47 [0.35, 0.64]**
Total events	82		476			
Heterogeneity: Tau² = 0.02; Chi² = 1.48, df = 1 (P = 0.22); I² = 32%						
Test for overall effect: Z = 4.95 (P < 0.00001)						

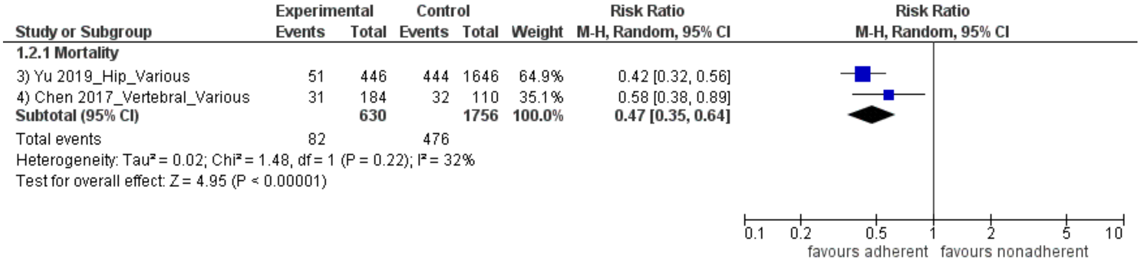

Heath related quality of life scores among adherent patients (adherence defined as taking more than 80% of pills consumed) compared to non-adherent subjects

Study or Subgroup	Experimental Mean	SD	Total	Control Mean	SD	Total	Weight	Mean Difference IV, Random, 95% CI
**1.5.1 1 year**								
McAlister 2019_Upper arm_Alendr/Risendr*_DASH	11.3	12.3	104	13.2	17.1	27	61.4%	-1.90 [-8.77, 4.97]
McAlister 2019_Upper arm_Alendr/Risendr*_OptQol	79.23	18.57	104	75.73	23.5	27	31.7%	3.50 [-6.06, 13.06]
McAlister 2019_Upper arm_Alendr/Risendr*_SF-12	51.6	8.25	104	51.65	54.1	27	6.9%	-0.05 [-20.52, 20.42]
**Subtotal (95% CI)**			**312**			**81**	**100.0%**	**-0.06 [-5.44, 5.32]**
Heterogeneity: Tau² = 0.00; Chi² = 0.81, df = 2 (P = 0.67); I² = 0%								
Test for overall effect: Z = 0.02 (P = 0.98)								
**1.5.2 2 years**								
McAlister 2019_Upper arm_Alendr/Risendr*_DASH	10.7	14.3	101	12.6	14.8	25	23.2%	-1.90 [-8.34, 4.54]
McAlister 2019_Upper arm_Alendr/Risendr*_OptQol	78.2	20.9	101	79.7	19.13	25	13.2%	-1.50 [-10.03, 7.03]
McAlister 2019_Upper arm_Alendr/Risendr*_SF-12	51.35	9.2	97	53.15	8.75	25	63.6%	-1.80 [-5.69, 2.09]
**Subtotal (95% CI)**			**299**			**75**	**100.0%**	**-1.78 [-4.88, 1.32]**
Heterogeneity: Tau² = 0.00; Chi² = 0.01, df = 2 (P = 1.00); I² = 0%								
Test for overall effect: Z = 1.13 (P = 0.26)								

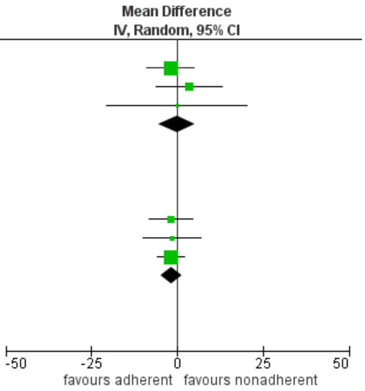

Refracture amont persistent patients (persistence defined by a gap of at least 30 days) compared to non-persistent subjects

Study or Subgroup	Experimental Events	Total	Control Events	Total	Weight	Risk Ratio M-H, Fixed, 95% CI
**4.1.1 Refracture**						
3) Soong 2013_Hip/Spine_Alendronate	298	7766	1121	24838	100.0%	0.85 [0.75, 0.96]
**Subtotal (95% CI)**		**7766**		**24838**	**100.0%**	**0.85 [0.75, 0.96]**
Total events	298		1121			
Heterogeneity: Not applicable						
Test for overall effect: Z = 2.54 (P = 0.01)						

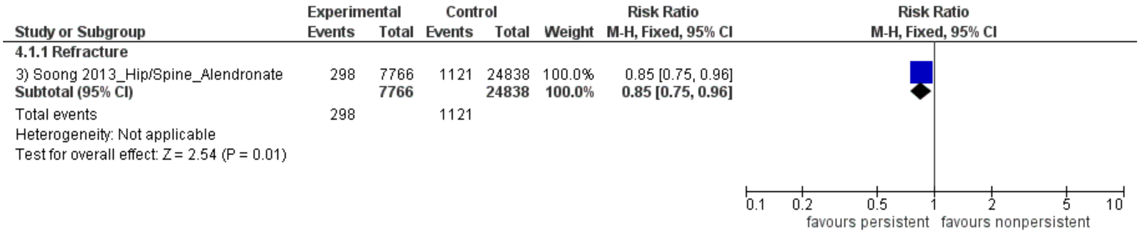

Refracture among persistent patients to anti-osteoporotic treatment for more than 12 months compared to non-persistent subjects

Study or Subgroup	Experimental Events	Total	Control Events	Total	Weight	Risk Ratio M-H, Random, 95% CI
**3.1.1 Vertebral fracture**						
4) Adams 2018_Hip/Femur/Vertebral_Bisphosphonates	54	1285	68	2202	100.0%	1.36 [0.96, 1.93]
**Subtotal (95% CI)**		**1285**		**2202**	**100.0%**	**1.36 [0.96, 1.93]**
Total events	54		68			
Heterogeneity: Not applicable						
Test for overall effect: Z = 1.72 (P = 0.08)						
**3.1.2 Nonvertebral fracture**						
2) Chan 2016_Fracture_Teriparatide	87	1153	332	3471	59.5%	0.79 [0.63, 0.99]
4) Adams 2018_Hip/Femur/Vertebral_Bisphosphonates	33	1285	46	2202	40.5%	1.23 [0.79, 1.91]
**Subtotal (95% CI)**		**2438**		**5673**	**100.0%**	**0.94 [0.62, 1.45]**
Total events	120		378			
Heterogeneity: Tau² = 0.07; Chi² = 3.07, df = 1 (P = 0.08); I² = 67%						
Test for overall effect: Z = 0.26 (P = 0.79)						
**3.1.3 Refracture**						
3) Hsu 2020_Hip_Various	20	210	112	736	45.5%	0.63 [0.40, 0.98]
4) Adams 2018_Hip/Femur/Vertebral_Bisphosphonates	196	1285	265	2202	54.5%	1.27 [1.07, 1.50]
**Subtotal (95% CI)**		**1495**		**2938**	**100.0%**	**0.92 [0.46, 1.83]**
Total events	216		377			
Heterogeneity: Tau² = 0.22; Chi² = 8.29, df = 1 (P = 0.004); I² = 88%						
Test for overall effect: Z = 0.24 (P = 0.81)						

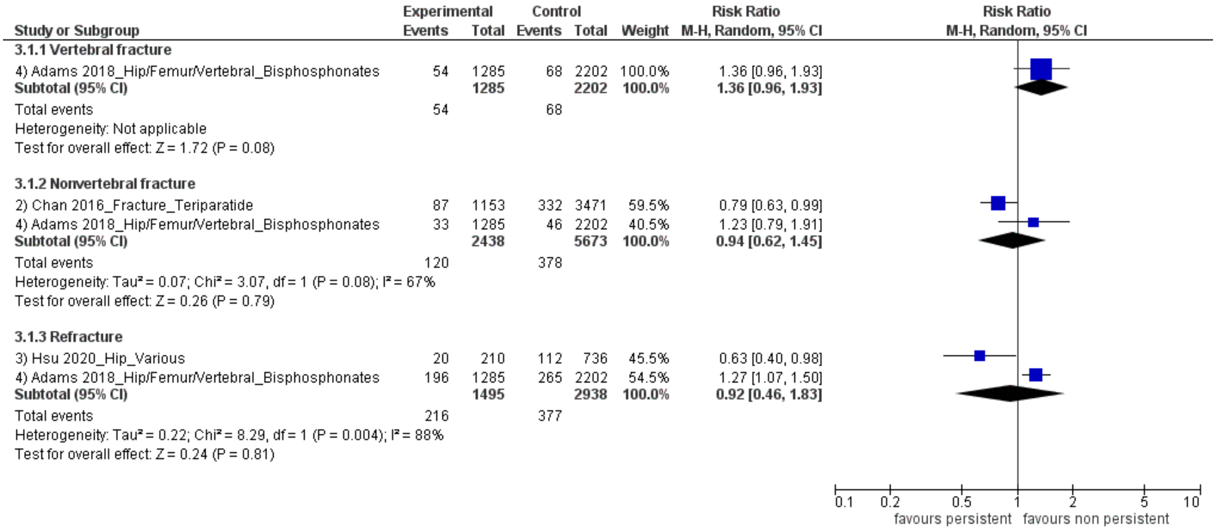

Refracture among patients with a continuous anti-osteoporotic treatment compared to subjects with a discontinued therapy

Study or Subgroup	Experimental Events	Experimental Total	Control Events	Control Total	Weight	Risk Ratio M-H, Random, 95% CI
**5.1.1 Vertebral fracture**						
2) Miller 1997_Vertebral_Etidronate*	4	93	15	100	45.6%	0.29 [0.10, 0.83]
4) Black 2006_Fracture_Alendronate*	12	226	9	150	54.4%	0.88 [0.38, 2.05]
**Subtotal (95% CI)**		**319**		**250**	**100.0%**	**0.53 [0.18, 1.60]**
Total events	16		24			
Heterogeneity: Tau² = 0.40; Chi² = 2.68, df = 1 (P = 0.10); I² = 63%						
Test for overall effect: Z = 1.13 (P = 0.26)						
**5.1.2 Nonvertebral fracture**						
4) Black 2006_Fracture_Alendronate*	35	226	62	150	100.0%	0.37 [0.26, 0.54]
**Subtotal (95% CI)**		**226**		**150**	**100.0%**	**0.37 [0.26, 0.54]**
Total events	35		62			
Heterogeneity: Not applicable						
Test for overall effect: Z = 5.35 (P < 0.00001)						
**5.1.3 Refracture**						
3) Cosman 2014_Vertebral_Zoledronate*	5	127	13	127	100.0%	0.38 [0.14, 1.05]
**Subtotal (95% CI)**		**127**		**127**	**100.0%**	**0.38 [0.14, 1.05]**
Total events	5		13			
Heterogeneity: Not applicable						
Test for overall effect: Z = 1.87 (P = 0.06)						

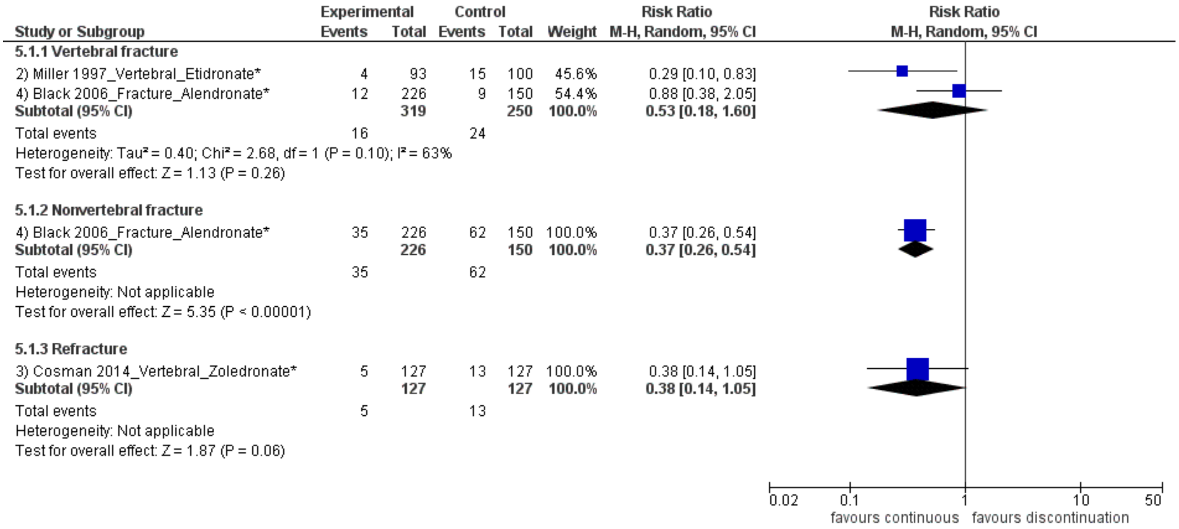

Mortality rate among persistent patients to anti-osteoporotic treatment for more than 12 months compared to non-persistent subjects

Study or Subgroup	Experimental Events	Experimental Total	Control Events	Control Total	Weight	Risk Ratio M-H, Fixed, 95% CI
**3.2.1 Mortality**						
3) Hsu 2020_Hip_Various	44	210	185	736	100.0%	0.83 [0.62, 1.11]
**Subtotal (95% CI)**		**210**		**736**	**100.0%**	**0.83 [0.62, 1.11]**
Total events	44		185			
Heterogeneity: Not applicable						
Test for overall effect: Z = 1.23 (P = 0.22)						

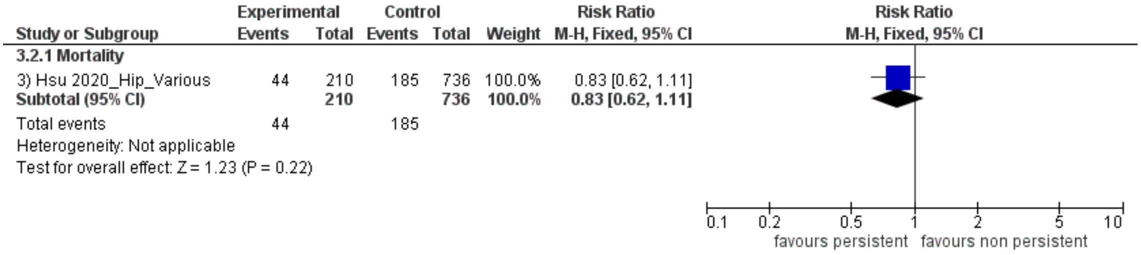

Adverse events among patients with a continuous anti-osteoporotic treatment compared to subjects with a discontinued therapy

Study or Subgroup	Experimental Events	Experimental Total	Control Events	Control Total	Weight	Risk Ratio M-H, Random, 95% CI
**5.2.1 Abdominal pain**						
2) Miller 1997_Vertebral_Etidronate*	14	93	9	100	100.0%	1.67 [0.76, 3.68]
**Subtotal (95% CI)**		**93**		**100**	**100.0%**	**1.67 [0.76, 3.68]**
Total events	14		9			
Heterogeneity: Not applicable						
Test for overall effect: Z = 1.28 (P = 0.20)						
**5.2.2 Diarrhea**						
2) Miller 1997_Vertebral_Etidronate*	12	93	16	100	100.0%	0.81 [0.40, 1.61]
**Subtotal (95% CI)**		**93**		**100**	**100.0%**	**0.81 [0.40, 1.61]**
Total events	12		16			
Heterogeneity: Not applicable						
Test for overall effect: Z = 0.61 (P = 0.54)						
**5.2.3 Nausea**						
2) Miller 1997_Vertebral_Etidronate*	12	93	13	100	100.0%	0.99 [0.48, 2.06]
**Subtotal (95% CI)**		**93**		**100**	**100.0%**	**0.99 [0.48, 2.06]**
Total events	12		13			
Heterogeneity: Not applicable						
Test for overall effect: Z = 0.02 (P = 0.98)						

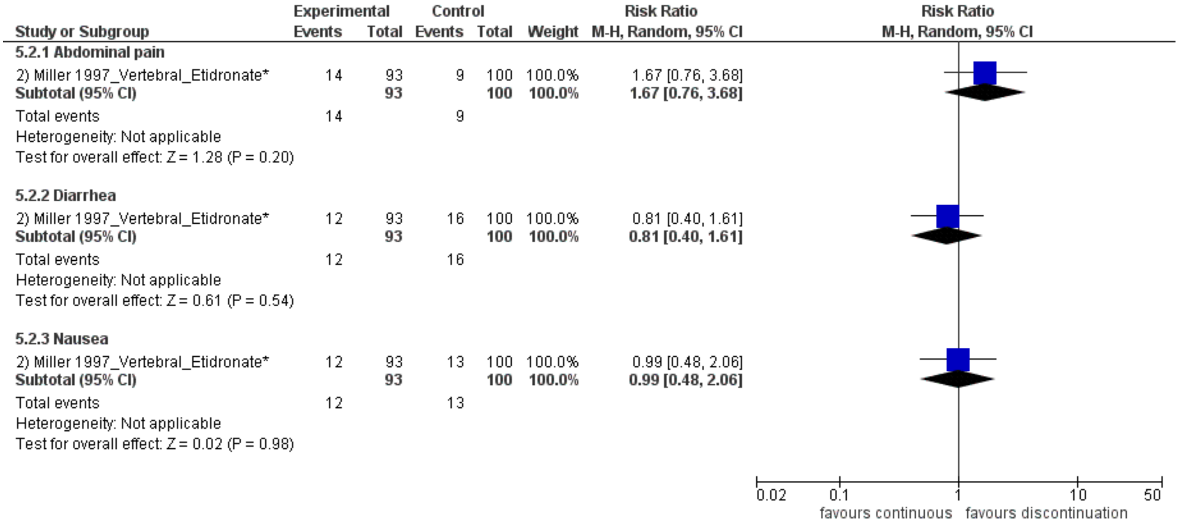

Refracture among patients with a continuous anti-osteoporotic treatment compared to subjects with a cyclic therapy

Study or Subgroup	Experimental Events	Experimental Total	Control Events	Control Total	Weight	Risk Ratio M-H, Random, 95% CI
**6.1.1 Vertebral fracture**						
3) Chesnut 2004_Vertebral_Ibandronate*	37	977	39	977	66.0%	0.95 [0.61, 1.47]
3) Clemmesen 1997_Fracture_Risedronate*	13	44	15	44	34.0%	0.87 [0.47, 1.60]
**Subtotal (95% CI)**		**1021**		**1021**	**100.0%**	**0.92 [0.64, 1.32]**
Total events	50		54			
Heterogeneity: Tau² = 0.00; Chi² = 0.06, df = 1 (P = 0.81); I² = 0%						
Test for overall effect: Z = 0.46 (P = 0.65)						
**6.1.2 Nonvertebral fracture**						
3) Clemmesen 1997_Fracture_Risedronate*	4	44	9	44	100.0%	0.44 [0.15, 1.34]
**Subtotal (95% CI)**		**44**		**44**	**100.0%**	**0.44 [0.15, 1.34]**
Total events	4		9			
Heterogeneity: Not applicable						
Test for overall effect: Z = 1.44 (P = 0.15)						
**6.1.3 Refracture**						
3) Chesnut 2004_Vertebral_Ibandronate*	22	977	22	977	100.0%	1.00 [0.56, 1.79]
**Subtotal (95% CI)**		**977**		**977**	**100.0%**	**1.00 [0.56, 1.79]**
Total events	22		22			
Heterogeneity: Not applicable						
Test for overall effect: Z = 0.00 (P = 1.00)						

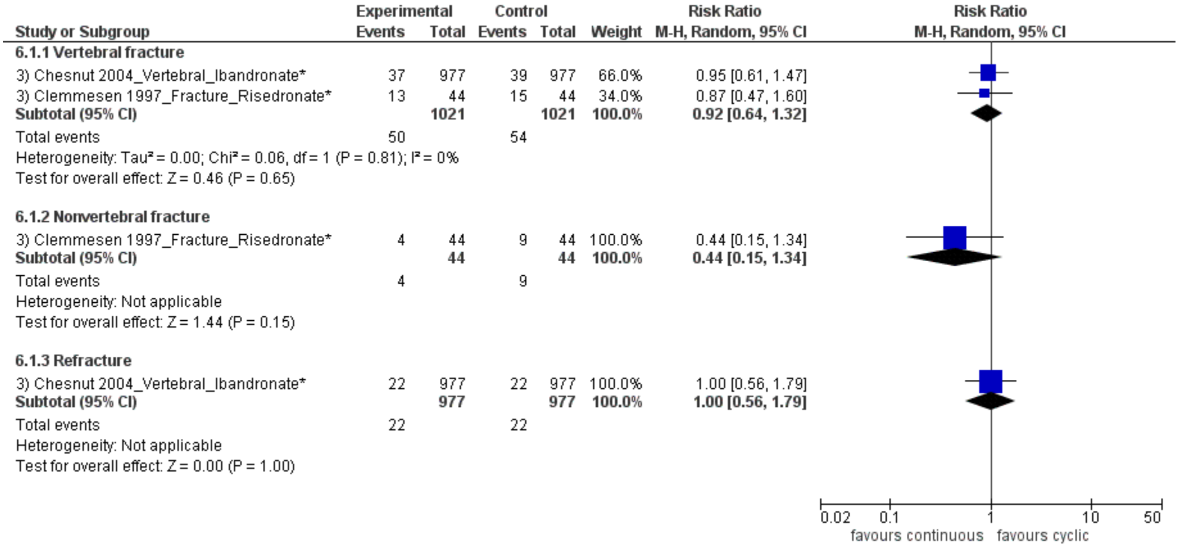

Adverse events among patients with a continuous anti-osteoporotic treatment compared to subjects with a cyclic therapy

Study or Subgroup	Experimental Events	Experimental Total	Control Events	Control Total	Weight	Risk Ratio M-H, Random, 95% CI
**6.2.1 Upper GI disorders**						
3) Chesnut 2004_Vertebral_Ibandronate*	303	977	297	977	99.3%	1.02 [0.89, 1.17]
3) Clemmesen 1997_Fracture_Risedronate*	3	44	3	44	0.7%	1.00 [0.21, 4.69]
**Subtotal (95% CI)**		**1021**		**1021**	**100.0%**	**1.02 [0.89, 1.16]**
Total events	306		300			
Heterogeneity: Tau² = 0.00; Chi² = 0.00, df = 1 (P = 0.98); I² = 0%						
Test for overall effect: Z = 0.29 (P = 0.77)						
**6.2.2 GI-esophagus**						
3) Chesnut 2004_Vertebral_Ibandronate*	19	977	11	977	71.1%	1.73 [0.83, 3.61]
3) Clemmesen 1997_Fracture_Risedronate*	1	44	3	44	28.9%	0.33 [0.04, 3.08]
**Subtotal (95% CI)**		**1021**		**1021**	**100.0%**	**1.07 [0.25, 4.64]**
Total events	20		14			
Heterogeneity: Tau² = 0.64; Chi² = 1.90, df = 1 (P = 0.17); I² = 47%						
Test for overall effect: Z = 0.10 (P = 0.92)						

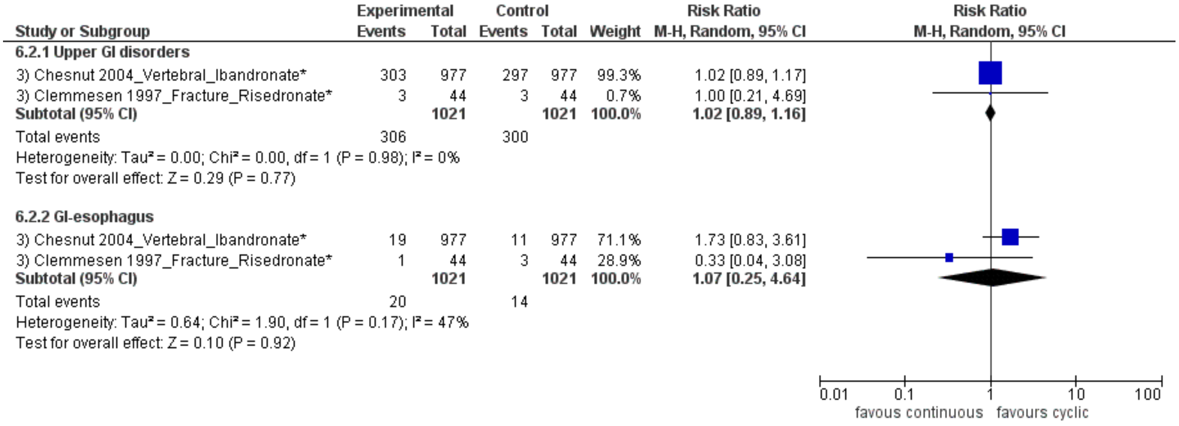

Quality evaluation

Observational studies evaluated with the Newcastle Ottawa Scale

	Selection				Comparability	Outcome			
Cohort study	Representativeness of the exposed cohort	Selection of the non exposed cohort	Ascertainment of exposure	Demonstration that outcome of interest was not present at start of study	Comparability of cohorts on the basis of the design or analysis	Assessment of outcome	Was follow-up long enough for outcomes to occur	Adequacy of follow-up of cohorts	tot
Lin 2011	*	*	*		*	*	*	*	7
Soong 2013	*	*	*		*	*	*	*	7
Keshishian 2017	*	*	*		*	*	*	*	7
Sheehy 2009	*	*	*			*	*	*	6
Hsu 2020	*	*	*			*	*	*	6
Chan 2016	*	*	*		*	*	*	*	7
Yu 2019	*	*	*			*	*	*	6
Chen 2017	*	*				*	*	*	5
Adams 2018	*	*	*		*	*	*	*	7

Randomized controlled trials evaluated with the Risk of Bias tool

	Random sequence generation (selection bias)	Allocation concealment (selection bias)	Blinding of participants and personnel (performance bias)	Blinding of outcome assessment (detection bias)	Incomplete outcome data (attrition bias)	Selective reporting (reporting bias)	Other bias
Black 2006	?	?	+	+	+	+	+
Chesnut 2004	?	?	+	+	+	+	+
Clemmesen 1997	?	?	+	+	?	+	?
Cosman 2014	?	?	+	+	?	+	+
McAlister 2019	?	?	+	+	+	+	+
Miller 1997	+	?	+	+	–	+	+

Summary of Findings

Adherence (MPR ≥80%) vs non-adherence

1. Certainty assessment							№ of patients		Effect		Certainty	Importance
**№ of studies**	**Study design**	**Risk of bias**	**Inconsistency**	**Indirectness**	**Imprecision**	**Other considerations**	**Adherent**	**Non adherent**	**Relative (95% CI)**	**Absolute (95% CI)**		
**Refracture**												
4	observational studies	not serious	serious [b]	not serious	not serious	none	714/25163 (2.8%)	2084/42930 (4.9%)	**RR 0.56** (0.39 to 0.80)	**12 fewer per 1.000** (from 17 fewer to 6 fewer)	⊕◯◯◯ VERY LOW	CRITICAL
**Mortality**												
2	observational studies	not serious	not serious	not serious	not serious	none	82/630 (13.0%)	476/1756 (27.1%)	**RR 0.47** (0.35 to 0.64)	**144 fewer per 1.000** (from 176 fewer to 98 fewer)	⊕⊕◯◯ LOW	CRITICAL
**Quality of life**												
1	randomised trial	not serious	not serious	not serious	serious [a]	none	1 year: 104<br>2 years: 101	1 year: 27<br>2 years: 25	-	1 year: MD 0.06 lower (5.44 lower to 5.32 higher)<br>2 years: MD 1.78 lower (4.88 lower to 1.32 higher)	⊕⊕⊕◯ MODERATE	IMPORTANT

CI: Confidence interval; **MD:** Mean difference; **RR:** Risk ratio

Explanations

a. Confidence intervals crossed the line of no difference with plausible effects in favour to the experimental group

b. I2>75%

Adherence (≥12 months) vs non-adherence

Certainty assessment							№ of patients		Effect		Certainty	Importance
№ of studies	Study design	Risk of bias	Inconsistency	Indirectness	Imprecision	Other considerations	Adherent >12 months	Adherent < 12 months	Relative (95% CI)	Absolute (95% CI)		
**Nonvertebral fracture**												
1	observational studies	not serious	not serious	not serious	not serious	none	119/1569 (7.6%)	286/3055 (9.4%)	**RR 0.81** (0.66 to 0.99)	**18 fewer per 1.000** (from 32 fewer to 1 fewer)	⨁⨁◯◯ LOW	CRITICAL

CI: Confidence interval; **RR:** Risk ratio

Explanations

a. Confidence intervals crossed the line of no difference with plausible effects in favour to the experimental group

Persistence (gap 30 days) vs non-persistence

Certainty assessment							№ of patients		Effect		Certainty	Importance
№ of studies	Study design	Risk of bias	Inconsistency	Indirectness	Imprecision	Other considerations	Persistent	Non persistent	Relative (95% CI)	Absolute (95% CI)		
**Refracture**												
1	observational studies	not serious	not serious	not serious	not serious	none	298/7766 (3.8%)	1121/24838 (4.5%)	**RR 0.85** (0.75 to 0.96)	**7 fewer per 1.000** (from 11 fewer to 2 fewer)	⨁⨁◯◯ LOW	CRITICAL

CI: Confidence interval; **MD:** Mean difference; **RR:** Risk ratio

Explanations

a. Confidence intervals crossed the line of no difference with plausible effects in favour to the experimental group

Persistence (≥12 months) vs non-persistence

Certainty assessment							№ of patients		Effect		Certainty	Importance
№ of studies	Study design	Risk of bias	Inconsistency	Indirectness	Imprecision	Other considerations	Persistent > 12 months	Persistent < 12 months	Relative (95% CI)	Absolute (95% CI)		
**Refracture**												
3	observational studies	not serious	serious [a]	not serious	serious [b]	none	390/5218 (7.5%)	823/10813 (7.6%)	**RR 1.02** (0.77 to 1.36)	**2 more per 1.000** (from 18 fewer to 27 more)	⨁◯◯◯ VERY LOW	CRITICAL
**Mortality**												
1	observational studies	not serious	not serious	not serious	serious [b]	none	44/210 (21.0%)	185/736 (25.1%)	**RR 0.83** (0.62 to 1.11)	**43 fewer per 1.000** (from 96 fewer to 28 more)	⨁◯◯◯ VERY LOW	CRITICAL

CI: Confidence interval; **RR:** Risk ratio

Explanations

a. I2>75%

b. Confidence intervals crossed the line of no difference with plausible effects in favour to the experimental group

Continuous vs discontinuous therapy

Certainty assessment							№ of patients		Effect		Certainty	Importance
**№ of studies**	**Study design**	**Risk of bias**	**Inconsistency**	**Indirectness**	**Imprecision**	**Other considerations**	**Continuous**	**Discontinuation**	**Relative (95% CI)**	**Absolute (95% CI)**		
**Refracture**												
3	randomised trials	serious [a]	not serious	not serious	not serious	none	56/672 (8.3%)	99/527 (18.8%)	**RR 0.43** (0.28 to 0.64)	**107 fewer per 1.000** (from 135 fewer to 68 fewer)	⨁⨁⨁◯ MODERATE	CRITICAL
**Adverse events**												
1	randomised trials	serious [a]	not serious	not serious	serious [b]	none	38/279 (13.6%)	38/300 (12.7%)	**RR 1.07** (0.70 to 1.63)	**9 more per 1.000** (from 38 fewer to 80 more)	⨁⨁◯◯ LOW	IMPORTANT

CI: Confidence interval; **MD:** Mean difference; **RR:** Risk ratio

Explanations

a. High risk of bias for incomplete outcome data (Miller 1997)

b. Confidence intervals crossed the line of no difference with plausible effects in favour to the experimental group

Continuous vs cyclic therapy

2. Certainty assessment							№ of patients		Effect		Certainty	Importance
**№ of studies**	**Study design**	**Risk of bias**	**Inconsistency**	**Indirectness**	**Imprecision**	**Other considerations**	**cyclic**	**continuous**	**Relative (95% CI)**	**Absolute (95% CI)**		
**Refracture**												
2	randomised trials	not serious	not serious	not serious	serious [a]	none	76/2042 (3.7%)	85/2042 (4.2%)	**RR 0.89** (0.66 to 1.20)	**5 fewer per 1.000** (from 14 fewer to 8 more)	⨁⨁⨁◯ MODERATE	CRITICAL
**GI disorders**												
2	randomised trials	not serious	not serious	not serious	serious [a]	none	326/2042 (16.0%)	314/2042 (15.4%)	**RR 1.03** (0.91 to 1.18)	**5 more per 1.000** (from 14 fewer to 28 more)	⨁⨁⨁◯ MODERATE	IMPORTANT

CI: Confidence interval; **RR:** Risk ratio

Explanations

a. Confidence intervals crossed the line of no difference with plausible effects in favour to the experimental group

CQ 6

Is the use of clinical governance models, such as the so-called Fracture Liaison Services, suitable for patients' post-fracture management?

Search strategy

From the SIGN Guideline

Review question 6:

8.1–8.3	**10. What is the clinical and cost effectiveness of integrated models of care (which include assessment, identification, treatment and follow up) compared with stand-alone elements for the primary and secondary prevention of fragility fracture?**
	**Population:**<br>individuals who have suffered a fragility fracture or identified as at increased risk of fracture<br>**Interventions:**<br>nurse-led clinics, structured service delivery models, fracture liaison service, educational materials (eg fracture/osteoporosis guidelines)<br>**Comparisons:**<br>individual osteoporosis services without integration (usual care)<br>**Outcomes:**<br>risk of vertebral/hip/other fracture at end of study/one year/ three years/five years/ 10 years, proportion of patients assessed and treated, adverse effects, incremental cost-effectiveness ratios

MEDLINE

Up to 17 December 2020

#1:

(((wrist* or colles or radius or articulatio radiocarpea or carpus or carpal or radiocarp* or radial or forearm* or humerus or metacarp* or barton or monteggi* or ulna or ulnar or upper limb* or hip or hips or trochanteric or intertrochanteric or subtrochanteric or femoral neck or femur neck or spine or spinal or vertebra or vertebral or vertebrae or lumbar or shoulder* or glenohumeral or humeroscapular or scapulo humeral or proximal humeral) adj3 fractur*) or (exp hip fractures/ or spinal fractures/ or shoulder fractures/ or osteoporotic fractures/ or exp radius fractures/) or (fractures, bone/ and (exp wrist joint/ or exp spine/ or shoulder/ or shoulder joint/ or hip/))) and (exp osteoporosis/ or (osteoporo* or bone loss*))

#2:

"fragility fracture"[ti] OR "fragility fractures"[ti] OR "low energy fracture"[ti] OR "low energy fractures"[ti] OR "low-energy fracture"[ti] OR "low-energy fractures"[ti] OR "low trauma fracture"[ti] OR "low trauma fractures"[ti] OR "low-trauma fracture"[ti] OR "low-trauma fractures"[ti] OR "low energy trauma"[ti] OR "low-energy trauma"[ti] OR "low level trauma"[ti] OR

“low-level trauma”[ti] OR “minor trauma fracture”[ti] OR “minor trauma fractures”[ti] OR “minor-trauma fracture”[ti] OR “minor-trauma fractures”[ti] OR “minor fracture”[ti] OR “minor fractures”[ti] OR “minor-fracture”[ti] OR “minor-fractures”[ti] OR “osteoporotic fracture”[ti] OR “osteoporotic fractures”[ti]

#3: #1 OR #2

#4: (exp Patient Care Team/) AND fracture*[tiab] AND (fragil*[tiab] OR osteopor*[tiab)

#5: exp Preventive Health Services/ AND fracture*[tiab] AND (fragil*[tiab] OR osteopor*[tiab)

#6:

((service*[Tiab] or program*[Tiab] or care[Tiab] or model*[Tiab] or intervention*[Tiab] or pathway*[Tiab]) AND (multifaceted[Tiab] or integrated[Tiab] or multimodal[Tiab] or multifaceted[Tiab] or coordinated[Tiab] or co-ordinated[Tiab])) AND (fracture*[tiab]) AND (fragil*[tiab] OR osteopor*[tiab)

#7:

(care pathway*[Tiab] or treatment pathway*[Tiab] or management pathway*[Tiab]) AND fracture*[tiab] AND (fragil*[tiab] OR osteopor*[tiab)

#8:

((service*[Tiab] or program*[Tiab] or care[Tiab]) AND delivery[Tiab]) AND (fracture*[tiab]) AND (fragil*[tiab] OR osteopor*[tiab)

#9:

(nurse*[Tiab] AND (clinic[Tiab] or clinics[Tiab])) AND fracture*[tiab] AND (fragil*[tiab] OR osteopor*[tiab)

#10:

(healthcare[Tiab] AND (delivery[Tiab] or model*[Tiab] or integrate*[Tiab])) AND (fracture*[tiab]) AND (fragil*[tiab] OR osteopor*[tiab)

#11:

(health care[Tiab] AND (delivery[Tiab] or model*[Tiab] or integrate*[Tiab])) AND (fracture*[tiab]) AND (fragil*[tiab] OR osteopor*[tiab)

#12:

(health service*[Tiab] AND (delivery[Tiab] or model*[Tiab] or integrate*[Tiab])) AND (fracture*[tiab]) AND (fragil*[tiab] OR osteopor*[tiab)

#13: (Recurrence/pc) AND (fragil*[tiab] OR osteopor*[tiab) AND fracture*[tiab]

#14:

((secondary fracture*[Tiab] or recurrent fracture*[Tiab] or subsequent fracture*[Tiab]) AND prevent*[Tiab]) AND (fragil*[tiab] OR osteopor*[tiab) AND fracture*[tiab]

#15: fracture*[Tiab] AND (clinic[Tiab] or clinics[Tiab]) AND (fragil*[tiab] OR osteopor*[tiab)

#16: fracture*[Tiab] AND (service*[Tiab] or team*[Tiab]) AND (fragil*[tiab] OR osteopor*[tiab)

#17: fracture liaison[Tiab] AND (fragil*[tiab] OR osteopor*[tiab) AND fracture*[tiab]

#18: after[Tiab] AND fracture*[Tiab] AND (fragil*[tiab] OR osteopor*[tiab])

#19: post[Tiab] AND fracture*[Tiab] AND (fragil*[tiab] OR osteopor*[tiab])

#20: postfracture[Tiab] AND (fragil*[tiab] OR osteopor*[tiab) AND fracture*[tiab]

#21:

(pathway*[Tiab] or service*[Tiab] or program*[Tiab] or model*[Tiab]) AND (fracture*[tiab]) AND (fragil*[tiab] OR osteopor*[tiab)

#22:

(discharge*[Tiab] AND (treat*[Tiab] or assess*[Tiab] or follow*[Tiab] or identif*[Tiab])) AND (fracture*[tiab]) AND (fragil*[tiab] OR osteopor*[tiab)

#23:

#4 OR #5 OR #6 OR #7 OR #8 OR #9 OR #10 OR #11 OR #12 OR #13 OR #14 OR #15 OR #16 OR #17 OR #18 OR #19 OR #20 OR #21 OR #22

#24: (#3 AND #23) AND limit: Humans

EMBASE

Up to 17 December 2020

#1:

'wrist fracture'/exp OR 'hip fracture'/exp OR 'spine fracture'/exp OR 'shoulder fracture'/exp OR 'fragility fracture'/exp OR 'radius fracture'/exp OR ((wrist* OR colle* OR radius OR 'articulatio radiocarpea' OR carpus OR carpal OR radiocarp* OR radial OR forearm* OR humerus OR metacarp* OR barton OR monteggi* OR ulna OR ulnar OR 'upper limb' OR 'upper limbs' OR hip OR hips OR trochanteric OR intertrochanteric OR subtrochanteric OR 'femoral neck' OR 'femur neck' OR spine OR spinal OR vertebra* OR lumbar OR shoulder* OR glenohumeral OR humeroscapular OR 'scapulo humeral' OR 'proximal humeral') NEAR/3 fractur*):ab,ti OR ('fracture'/exp AND ('wrist'/exp OR 'hip'/exp OR 'spine'/exp OR 'shoulder'/exp OR 'wrist injury'/de OR 'shoulder injury'/exp OR 'hip injury'/exp OR 'spine injury'/exp)) AND ('osteoporosis'/exp OR osteoporo*:ab,ti OR 'bone loss':ab,ti)

#2: 'fragility fracture'/exp

#3: 'low energy fracture'/exp

#4: 'low trauma fracture'/exp

#5: 'low energy trauma'/exp

#6:

“fragility fracture”:ti OR “fragility fractures”:ti OR “low energy fracture”:ti OR “low energy fractures”:ti OR “low-energy fracture”:ti OR “low-energy fractures”:ti OR “low trauma fracture”:ti OR “low trauma fractures”:ti OR “low-trauma fracture”:ti OR “low-trauma fractures”:ti OR “low energy trauma”:ti OR “low-energy trauma”:ti OR “low level trauma”:ti OR “low-level trauma”:ti OR “minor trauma fracture”:ti OR “minor trauma fractures”:ti OR “minor-trauma fracture”:ti OR “minor-trauma fractures”:ti OR “minor fracture”:ti OR “minor fractures”:ti OR “minor-fracture”:ti OR “minor-fractures”:ti OR “osteoporotic fracture”:ti OR “osteoporotic fractures”:ti

#7: #1 OR #2 OR #3 OR #4 OR #5 OR #6

#8: (‘patient Care’) AND fracture*:ti,ab AND (fragil*:ti,ab OR osteopor*:ti,ab)

#9: (‘preventive Health Service’) AND fracture*:ti,ab AND (fragil*:ti,ab OR osteopor*:ti,ab)

#10:

((service*:ti,ab or program*:ti,ab or care:ti,ab or model*:ti,ab or intervention*:ti,ab or pathway*:ti,ab) AND (multifaceted:ti,ab or integrated:ti,ab or multimodal:ti,ab or multifaceted:ti,ab or coordinated:ti,ab or co-ordinated:ti,ab)) AND (fracture*:ti,ab) AND (fragil*:ti,ab OR osteopor*:ti,ab)

#11:

(care pathway*:ti,ab or treatment pathway*:ti,ab or management pathway*:ti,ab) AND fracture*:ti,ab AND (fragil*:ti,ab OR osteopor*:ti,ab)

#12:

((service*:ti,ab or program*:ti,ab or care:ti,ab) AND delivery:ti,ab) AND (fracture*:ti,ab) AND (fragil*:ti,ab OR osteopor*:ti,ab)

#13:

(nurse*:ti,ab AND (clinic:ti,ab or clinics:ti,ab)) AND fracture*:ti,ab AND (fragil*:ti,ab OR osteopor*:ti,ab)

#14:

(Healthcare:ti,ab AND (delivery:ti,ab or model*:ti,ab or integrate*:ti,ab)) AND (fracture*:ti,ab) AND (fragil*:ti,ab OR osteopor*:ti,ab)

#15:

(health care:ti,ab AND (delivery:ti,ab or model*:ti,ab or integrate*:ti,ab)) AND (fracture*:ti,ab) AND (fragil*:ti,ab OR osteopor*:ti,ab)

#16:

(health service*:ti,ab AND (delivery:ti,ab or model*:ti,ab or integrate*:ti,ab)) AND (fracture*:ti,ab) AND (fragil*:ti,ab OR osteopor*:ti,ab)

#17: (‘recurrence risk’) AND (fracture*:ti,ab) AND (fragil*:ti,ab OR osteopor*:ti,ab)

#18:

((secondary fracture*:ti,ab or recurrent fracture*:ti,ab or subsequent fracture*:ti,ab) AND prevent*:ti,ab) AND fracture*:ti,ab AND (fragil*:ti,ab OR osteopor*:ti,ab)

#19: fracture*:ti,ab AND (clinic:ti,ab or clinics:ti,ab) AND (fragil*:ti,ab OR osteopor*:ti,ab)

#20: fracture*:ti,ab AND (service*:ti,ab or team*:ti,ab) AND (fragil*:ti,ab OR osteopor*:ti,ab)

#21: fracture liaison:ti,ab AND (fragil*:ti,ab OR osteopor*:ti,ab) AND fracture*:ti,ab

#22: after:ti,ab AND fracture*:ti,ab AND (fragil*:ti,ab OR osteopor*:ti,ab)

#23: post:ti,ab AND fracture*:ti,ab AND (fragil*:ti,ab OR osteopor*:ti,ab)

#24: postfracture:ti,ab AND (fragil*:ti,ab OR osteopor*:ti,ab) AND fracture*:ti,ab

#25:

(pathway*:ti,ab or service*:ti,ab or program*:ti,ab or model*:ti,ab) AND (fracture*:ti,ab) AND (fragil*:ti,ab OR osteopor*:ti,ab)

#26:

(discharge*:ti,ab AND (treat*:ti,ab or assess*:ti,ab or follow*:ti,ab or identif*:ti,ab)) AND (fracture*:ti,ab) AND (fragil*:ti,ab OR osteopor*:ti,ab)

#27:

#8 OR #9 OR #10 OR #11 OR #12 OR #13 OR #14 OR #15 OR #16 OR #17 OR #18 OR #19 OR #20 OR #21 OR #22 OR #23 OR #24 OR #25 OR #26

#28: (#7 AND #27) AND limit: Humans

COCHRANE SEARCH

Up to 13 January 2021

#1:

((wrist* or colle* or radius or "articulatio radiocarpea" or carpus or carpal or radiocarp* or radial or forearm* or humerus or metacarp* or barton or monteggi* or ulna or ulnar or "upper limb" or "upper limbs" or hip or hips or trochanteric or intertrochanteric or subtrochanteric or "femoral neck" or "femur neck" or spine or spinal or vertebra* or lumbar or shoulder* or glenohumeral or humeroscapular or "scapulo humeral" or "proximal humeral") near/3 fractur*):ti,ab or [mh "hip fractures"] or [mh "spinal fractures"] or [mh "shoulder fractures"] or [mh "osteoporotic fractures"] or

[mh "radius fractures"] or ([mh "bone fractures"] and ([mh "wrist joint"] or [mh spine] or [mh shoulder] or [mh "shoulder joint"] or [mh hip])) and ([mh osteoporosis] or (osteoporo* or "bone loss" OR fragility):ti,ab)

#2: MeSH descriptor: [Osteoporotic Fractures] explode all trees

#3: MeSH descriptor: [Fractures, Spontaneous] explode all trees

#4:

(fragility fracture):ti OR (fragility fractures):ti OR (low energy fracture):ti OR (low energy fractures):ti OR (low-energy fracture):ti OR (low-energy fractures):ti OR (low trauma fracture):ti OR (low trauma fractures):ti OR (low-trauma fracture):ti OR (low-trauma fractures):ti OR (low energy trauma):ti OR (low-energy trauma):ti OR (low level trauma):ti OR (low-level trauma):ti OR (minor trauma fracture):ti OR (minor trauma fractures):ti OR (minor-trauma fracture):ti OR (minor-trauma fractures):ti OR (minor fracture):ti OR (minor fractures):ti OR (minor-fracture):ti OR (minor-fractures):ti OR (osteoporotic fracture):ti OR (osteoporotic fractures):ti OR (pathologic fracture):ti OR (pathological fractures):ti

#5: #1 OR #2 OR #3 OR #4

#6:

“patient Care”:ti,ab OR “patient-care”:ti,ab OR fracture liaison:ti,ab OR clinic:ti,ab OR clinics:ti,ab OR service*:ti,ab OR team*:ti,ab OR care pathway*:ti,ab OR treatment pathway*:ti,ab OR management pathway*:ti,ab OR after:ti,ab OR post:ti,ab OR postfracture:ti,ab pathway*:ti,ab OR service*:ti,ab OR program*:ti,ab OR model*:ti,ab

#7: #6 AND (fracture*:ti,ab AND (fragil*:ti,ab OR osteopor*:ti,ab))

#8: #5 AND #7

#9: #8 with Cochrane Library publication date from Jan 2013 to present

#10: #9 NOT ((MH "Animals+") OR (MH "Animal Studies") OR (TI "animal model*"))

Flow chart

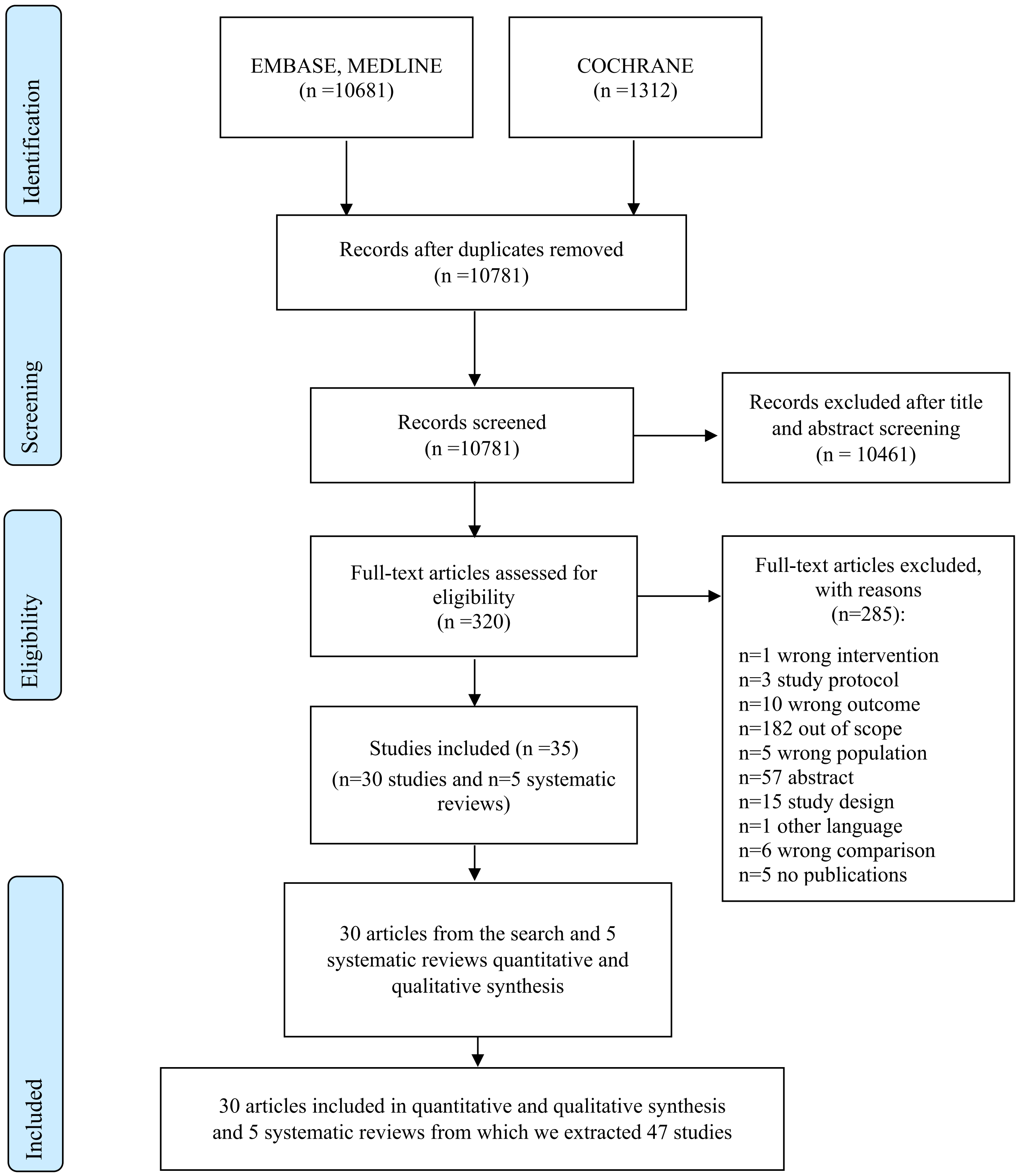
Identification
Screening
Eligibility
Included
EMBASE, MEDLINE
(n =10681)
COCHRANE
(n =1312)
Records after duplicates removed
(n =10781)
Records screened
(n =10781)
Records excluded after title and abstract screening
(n = 10461)
Full-text articles assessed for eligibility
(n =320)
Full-text articles excluded, with reasons
(n=285):
n=1 wrong intervention
n=3 study protocol
n=10 wrong outcome
n=182 out of scope
n=5 wrong population
n=57 abstract
n=15 study design
n=1 other language
n=6 wrong comparison
n=5 no publications
Studies included (n =35)
(n=30 studies and n=5 systematic reviews)
30 articles from the search and 5 systematic reviews quantitative and qualitative synthesis
30 articles included in quantitative and qualitative synthesis and 5 systematic reviews from which we extracted 47 studies

Results

DXA testing in the FLS group compared to usual care

Study or Subgroup	Experimental Events	Experimental Total	Control Events	Control Total	Weight	Risk Ratio M-H, Random, 95% CI
Amphansap 2016	36	75	34	120	2.7%	1.69 [1.17, 2.45]
Amphansap 2020	303	353	34	120	2.8%	3.03 [2.27, 4.04]
Aubry-Rozier 2018	239	332	57	214	2.9%	2.70 [2.14, 3.41]
Axelsson 2016	1036	2616	206	2713	3.0%	5.22 [4.54, 6.00]
Bachour 2017	64	98	28	100	2.7%	2.33 [1.65, 3.30]
Beaton 2017	216	225	129	199	3.1%	1.48 [1.33, 1.65]
Beaton 2017b	8180	39317	3677	24676	3.1%	1.40 [1.35, 1.45]
Brankin 2005	652	720	399	436	3.1%	0.99 [0.95, 1.03]
Chan 2015	405	123009	431	101649	3.0%	0.78 [0.68, 0.89]
Cosman 2017	39	60	26	75	2.7%	1.88 [1.31, 2.69]
Cranney 2008*	67	125	37	145	2.8%	2.10 [1.52, 2.90]
Davidson 2017	38	93	19	47	2.6%	1.01 [0.66, 1.55]
Davis 2007*	15	28	0	20	0.3%	22.45 [1.42, 354.54]
Greenspan 2018	98	148	273	344	3.0%	0.83 [0.73, 0.95]
Harrington 2005	27	37	0	55	0.3%	81.05 [5.10, 1288.83]
Hawker 2003	114	139	128	139	3.1%	0.89 [0.81, 0.98]
Heilmann 2012	289	827	94	302	3.0%	1.12 [0.93, 1.36]
Hofflich 2014	53	71	2	61	1.0%	22.77 [5.79, 89.57]
Jaglal 2009	23	93	33	103	2.5%	0.77 [0.49, 1.21]
Johnson 2005	85	136	16	126	2.5%	4.92 [3.06, 7.92]
Kamel 2000	8	157	0	12	0.3%	1.40 [0.09, 22.90]
Laslett 2007	20	68	7	28	2.0%	1.18 [0.56, 2.47]
Leslie 2012*	209	1421	58	1480	2.9%	3.75 [2.83, 4.97]
Majumdar 2004	34	55	8	47	2.1%	3.63 [1.87, 7.06]
Majumdar 2008*	71	137	24	135	2.7%	2.92 [1.96, 4.34]
Majumdar 2011*	17	21	3	25	1.4%	6.75 [2.29, 19.90]
Majumdar 2018*	128	180	108	181	3.0%	1.19 [1.02, 1.39]
Merle 2017*	90	220	54	216	2.9%	1.64 [1.24, 2.17]
Miki 2008*	7	31	26	31	2.1%	0.27 [0.14, 0.53]
Morrish 2009*	88	110	32	110	2.8%	2.75 [2.02, 3.74]
Murray 2005	11	144	137	335	2.3%	0.19 [0.10, 0.33]
Queally 2013*	21	31	11	30	2.4%	1.85 [1.09, 3.14]
Roux 2013*	192	311	72	200	3.0%	1.71 [1.40, 2.10]
Roy 2011	62	70	17	70	2.6%	3.65 [2.39, 5.56]
Ruggiero 2015	100	210	25	172	2.7%	3.28 [2.22, 4.83]
Sidwell 2004	179	193	20	178	2.6%	8.25 [5.45, 12.50]
Singh 2019	53	101	13	55	2.4%	2.22 [1.33, 3.70]
Sofie 2016	32	50	10	86	2.2%	5.50 [2.96, 10.22]
Tosi 2008	39	359	47	276	2.7%	0.64 [0.43, 0.95]
Vaculík 2017	6	47	3	49	1.1%	2.09 [0.55, 7.86]
Van Helden 2007	10	14	3	47	1.3%	11.19 [3.56, 35.13]
Yuksel 2010*	28	129	13	133	2.2%	2.22 [1.20, 4.09]
**Total (95% CI)**		**172561**		**135540**	**100.0%**	**1.91 [1.61, 2.27]**
Total events	13384		6314			

Heterogeneity: $Tau^2 = 0.25$; $Chi^2 = 1579.01$, df = 41 ($P < 0.00001$); $I^2 = 97\%$

Test for overall effect: Z = 7.37 ($P < 0.00001$)

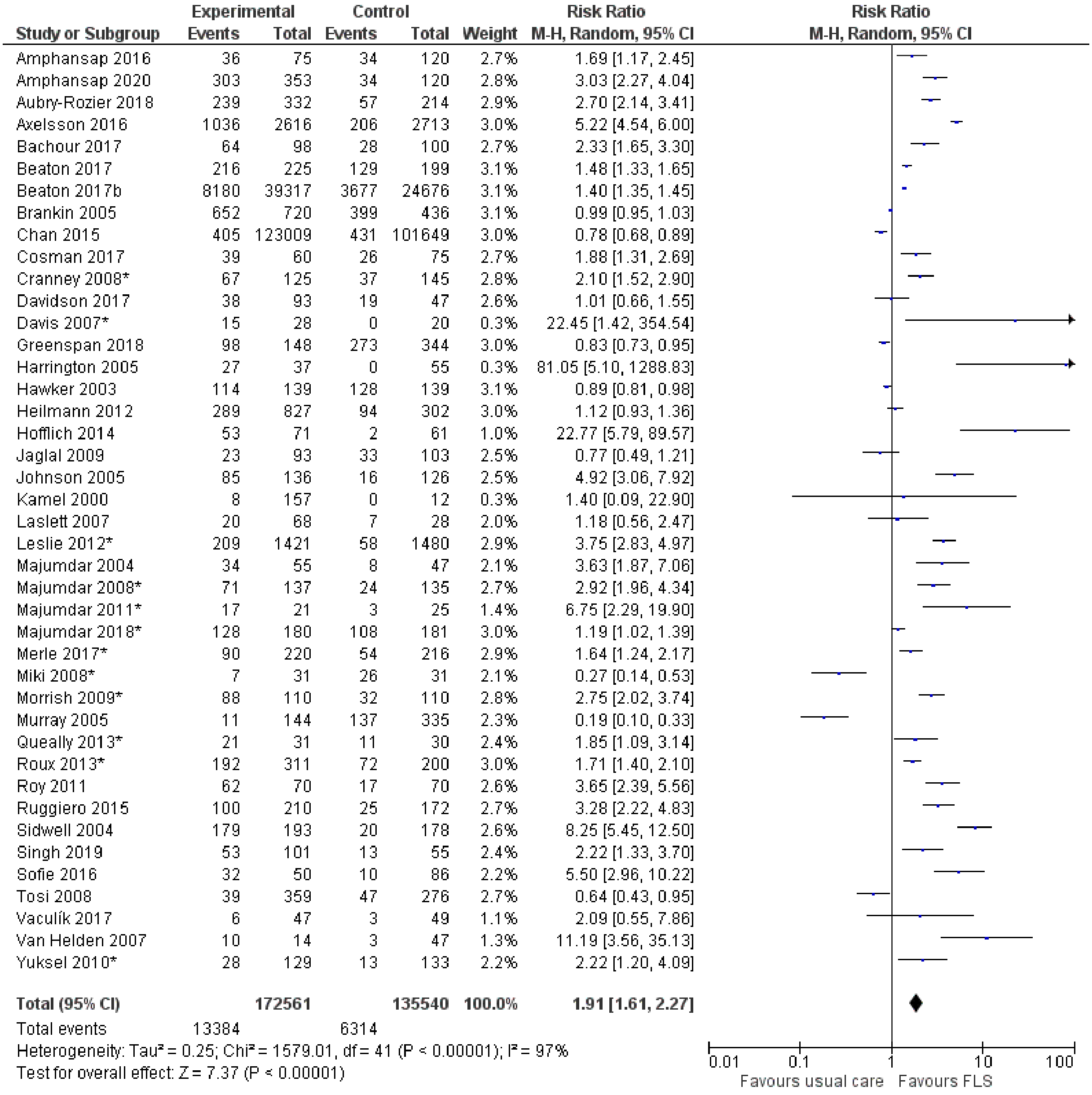

Antiosteoporotic initiation in the FLS group compared to usual care

	Experimental		Control			Risk Ratio
Study or Subgroup	Events	Total	Events	Total	Weight	M-H, Random, 95% CI
Amphansap 2016	17	75	18	120	2.2%	1.51 [0.83, 2.74]
Amphansap 2020	244	353	49	120	3.0%	1.69 [1.35, 2.12]
Astrand 2012	77	286	52	306	2.8%	1.58 [1.16, 2.17]
Axelsson 2016	832	2616	342	2713	3.1%	2.52 [2.25, 2.83]
Baroni 2019	60	112	63	210	2.9%	1.79 [1.36, 2.34]
Beaton 2017	72	225	32	199	2.7%	1.99 [1.37, 2.88]
Cosman 2017	11	60	25	75	2.1%	0.55 [0.30, 1.03]
Cranney 2008*	35	125	15	145	2.3%	2.71 [1.55, 4.72]
Davis 2007*	15	28	0	20	0.3%	22.45 [1.42, 354.54]
Fisher 2006	63	447	59	504	2.8%	1.20 [0.86, 1.68]
Greenspan 2018	40	74	44	226	2.8%	2.78 [1.98, 3.89]
Hawker 2003	24	139	22	139	2.3%	1.09 [0.64, 1.85]
Heilmann 2012	347	827	72	302	3.0%	1.76 [1.42, 2.19]
Hofflich 2014	43	71	5	61	1.6%	7.39 [3.12, 17.47]
Jachna 2003	19	29	46	53	2.9%	0.75 [0.57, 1.00]
Jaglal 2009	12	93	17	103	2.0%	0.78 [0.39, 1.55]
Johnson 2005	57	136	15	126	2.4%	3.52 [2.10, 5.89]
Jones 2005	54	93	60	161	2.9%	1.56 [1.20, 2.03]
Kamel 2000	2	157	1	12	0.4%	0.15 [0.01, 1.57]
Laslett 2007	56	68	7	28	2.1%	3.29 [1.72, 6.32]
Leslie 2012*	222	1421	157	1480	3.0%	1.47 [1.22, 1.78]
Lih 2011	198	246	71	157	3.0%	1.78 [1.48, 2.14]
Majumdar 2004	22	55	5	47	1.6%	3.76 [1.54, 9.15]
Majumdar 2008*	30	137	9	135	2.0%	3.28 [1.62, 6.65]
Majumdar 2011*	9	21	3	25	1.2%	3.57 [1.11, 11.52]
Majumdar 2018*	86	180	51	181	2.9%	1.70 [1.28, 2.24]
Merle 2017*	117	220	71	216	3.0%	1.62 [1.29, 2.03]
Miki 2008*	15	31	7	31	1.9%	2.14 [1.02, 4.52]
Morrish 2009*	56	110	24	110	2.6%	2.33 [1.57, 3.48]
Murray 2005	6	144	30	335	1.7%	0.47 [0.20, 1.09]
Naranjo 2017	14	66	50	93	2.4%	0.39 [0.24, 0.65]
Queally 2013*	28	31	30	30	3.1%	0.91 [0.80, 1.03]
Rolnick 2001*	10	207	0	187	0.3%	18.98 [1.12, 321.69]
Roux 2013*	182	311	71	200	3.0%	1.65 [1.34, 2.03]
Roy 2011	16	70	16	70	2.2%	1.00 [0.54, 1.84]
Ruggiero 2015	102	210	30	172	2.7%	2.78 [1.96, 3.97]
Sidwell 2004	60	193	18	178	2.4%	3.07 [1.89, 5.00]
Streetan 2006	36	53	31	31	3.0%	0.69 [0.57, 0.83]
Tosi 2008	23	359	14	276	2.1%	1.26 [0.66, 2.41]
Vaculík 2017	5	47	3	49	0.9%	1.74 [0.44, 6.87]
Van der Kallen 2014	174	214	124	220	3.1%	1.44 [1.26, 1.65]
Wallace 2011	42	46	26	42	2.9%	1.47 [1.14, 1.90]
Yuksel 2010*	28	129	15	133	2.2%	1.92 [1.08, 3.43]
**Total (95% CI)**		**10515**		**10021**	**100.0%**	**1.62 [1.39, 1.91]**
Total events	3561		1800			

Heterogeneity: $Tau^2 = 0.21$; $Chi^2 = 434.49$, df = 42 ($P < 0.00001$); $I^2 = 90\%$

Test for overall effect: $Z = 5.98$ ($P < 0.00001$)

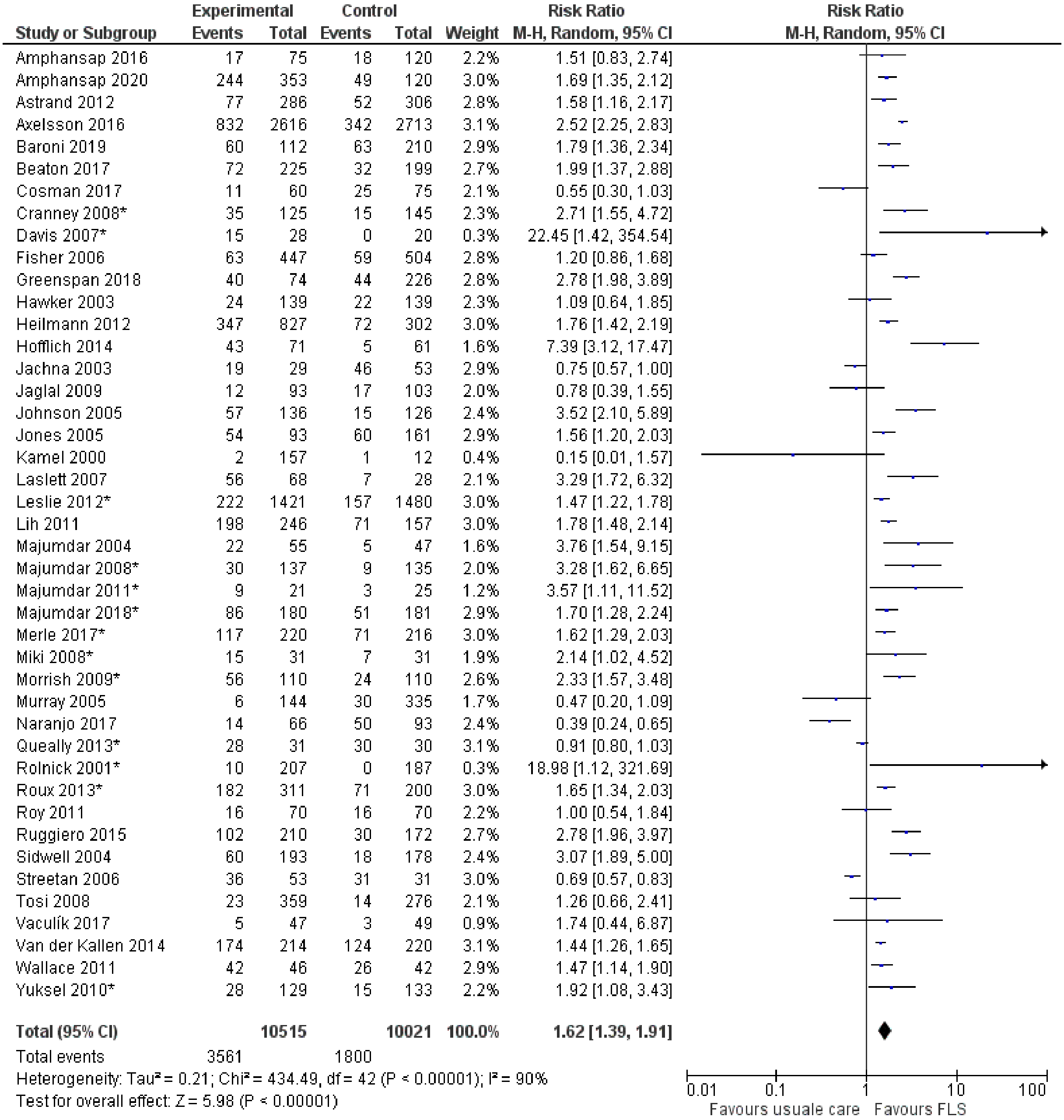

Antiosteoporotic adherence in the FLS group compared to usual care

Study or Subgroup	Experimental Events	Experimental Total	Control Events	Control Total	Weight	Risk Ratio M-H, Random, 95% CI
Amphansap 2016	96	120	31	75	10.7%	1.94 [1.46, 2.57]
Bachour 2017	53	98	26	100	9.0%	2.08 [1.43, 3.03]
Beaton 2017	203	225	151	199	13.6%	1.19 [1.09, 1.30]
Beaton 2017b	15727	39317	11746	24676	14.0%	0.84 [0.83, 0.86]
Ganda 2014*	22	45	23	49	8.3%	1.04 [0.68, 1.59]
Heilmann 2012	134	291	20	71	8.8%	1.63 [1.10, 2.42]
Laslett 2007	65	68	24	28	12.8%	1.12 [0.95, 1.31]
Majumdar 2008*	132	137	121	135	13.8%	1.07 [1.01, 1.15]
Ruggiero 2015	83	210	25	172	8.6%	2.72 [1.82, 4.05]
Streetan 2006	22	34	0	6	0.5%	9.00 [0.62, 131.58]
**Total (95% CI)**		**40545**		**25511**	**100.0%**	**1.35 [1.12, 1.64]**
Total events	16537		12167			

Heterogeneity: Tau² = 0.07; Chi² = 210.81, df = 9 (P < 0.00001); I² = 96%
Test for overall effect: Z = 3.14 (P = 0.002)

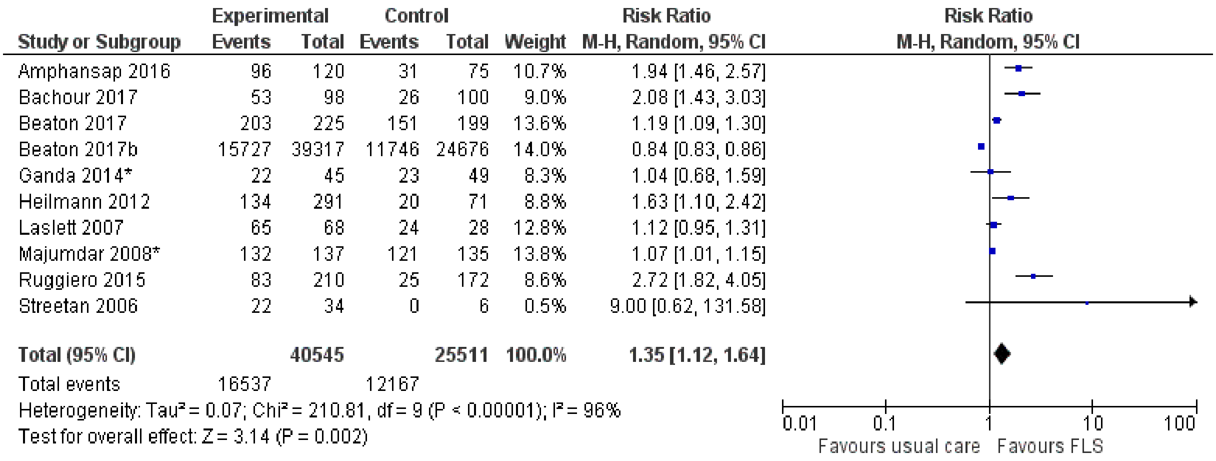

Risk of refracture in the FLS group compared to usual care

Study or Subgroup	Experimental Events	Experimental Total	Control Events	Control Total	Weight	Risk Ratio M-H, Random, 95% CI
Amphansap 2016	0	75	36	120	1.2%	0.02 [0.00, 0.35]
Astrand 2012	39	286	69	306	7.8%	0.60 [0.42, 0.87]
Aubry-Rozier 2018	18	332	11	214	6.0%	1.05 [0.51, 2.19]
Axelsson 2016	217	2616	228	2713	8.4%	0.99 [0.83, 1.18]
Chan 2015	327	123009	94	101649	8.2%	2.87 [2.29, 3.62]
Davidson 2017	34	328	25	131	7.3%	0.54 [0.34, 0.87]
Huntjens 2011	89	1335	191	1920	8.2%	0.67 [0.53, 0.85]
Huntjens 2014	95	1412	130	1910	8.1%	0.99 [0.77, 1.28]
Inderjeeth 2018	17	202	8	45	5.8%	0.47 [0.22, 1.03]
Lih 2011	10	246	31	157	6.2%	0.21 [0.10, 0.41]
Miki 2008*	0	31	1	31	1.0%	0.33 [0.01, 7.88]
Nakayama 2016	57	515	67	416	7.9%	0.69 [0.49, 0.95]
Sanli 2019	23	122	30	160	7.2%	1.01 [0.62, 1.64]
Singh 2019	3	101	1	55	1.7%	1.63 [0.17, 15.33]
Van der Kallen 2014	14	214	41	220	6.8%	0.35 [0.20, 0.62]
Wasfie 2019	78	215	84	150	8.2%	0.65 [0.52, 0.81]
**Total (95% CI)**		**131039**		**110197**	**100.0%**	**0.70 [0.51, 0.97]**
Total events	1021		1047			

Heterogeneity: Tau² = 0.33; Chi² = 165.51, df = 15 (P < 0.00001); I² = 91%
Test for overall effect: Z = 2.11 (P = 0.03)

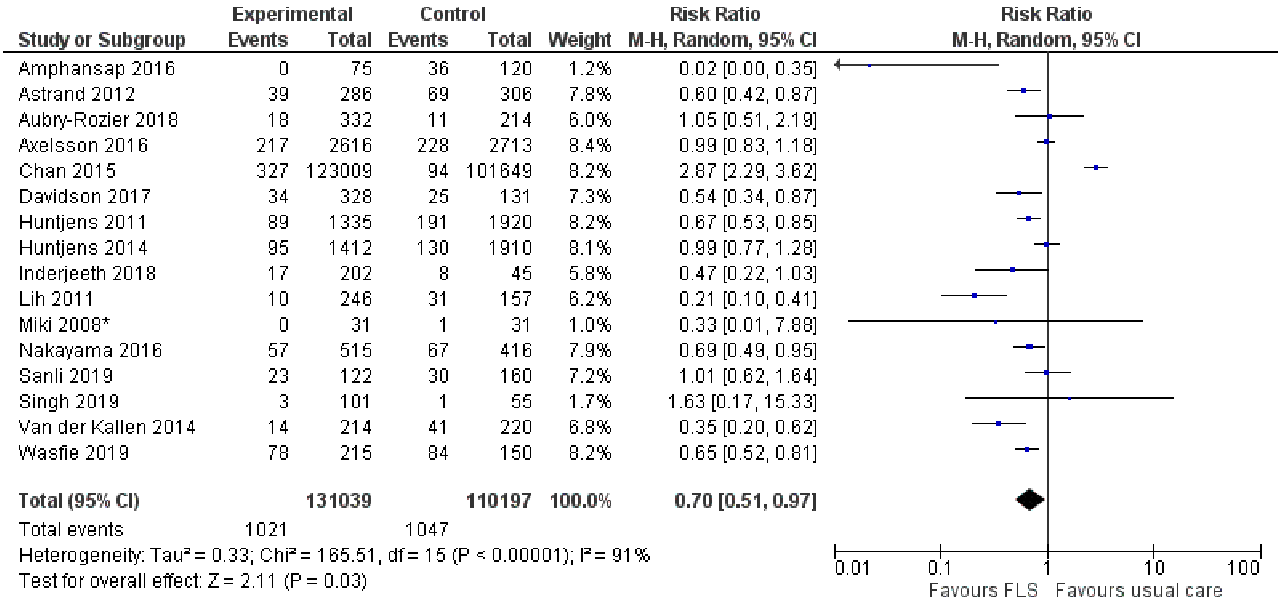

Risk of mortality in the FLS group compared to usual care

Study or Subgroup	Experimental Events	Experimental Total	Control Events	Control Total	Weight	Risk Ratio M-H, Random, 95% CI
Amphansap 2016	8	75	11	120	2.4%	1.16 [0.49, 2.76]
Anderson 2017	0	117	1	154	0.3%	0.44 [0.02, 10.65]
Anighoro 2020	3	147	7	116	1.3%	0.34 [0.09, 1.28]
Astrand 2012	34	286	52	306	4.9%	0.70 [0.47, 1.04]
Aubry-Rozier 2018	7	332	15	214	2.4%	0.30 [0.12, 0.73]
Axelsson 2016	319	2616	361	2713	6.4%	0.92 [0.80, 1.05]
Bachour 2017	16	98	16	100	3.5%	1.02 [0.54, 1.92]
Baroni 2019	16	112	46	210	4.1%	0.65 [0.39, 1.10]
Brañas 2018	29	643	35	578	4.4%	0.74 [0.46, 1.20]
Coventry 2017	6	137	10	183	2.0%	0.80 [0.30, 2.15]
Davidson 2017	67	328	16	131	4.2%	1.67 [1.01, 2.77]
Fisher 2006	21	447	39	504	4.1%	0.61 [0.36, 1.02]
Henderson 2017	20	206	47	248	4.3%	0.51 [0.31, 0.84]
Huntjens 2011	120	1335	384	1920	6.2%	0.45 [0.37, 0.54]
Huntjens 2014	162	1412	235	1910	6.2%	0.93 [0.77, 1.13]
Lamb 2017	4	240	3	196	1.1%	1.09 [0.25, 4.81]
Majumdar 2008*	1	137	0	135	0.3%	2.96 [0.12, 71.94]
Miki 2008*	2	31	5	31	1.0%	0.40 [0.08, 1.91]
Nakayama 2016	167	515	108	416	6.1%	1.25 [1.02, 1.53]
Rotman-Pikielny 2018	37	219	46	218	4.9%	0.80 [0.54, 1.18]
Ruggiero 2015	33	210	22	172	4.2%	1.23 [0.74, 2.03]
Sanli 2019	33	122	58	160	5.2%	0.75 [0.52, 1.07]
Schuijt 2020	153	524	75	282	5.9%	1.10 [0.87, 1.39]
Shigemoto 2018	12	132	12	104	2.9%	0.79 [0.37, 1.68]
Soong 2016	7	331	12	240	2.3%	0.42 [0.17, 1.06]
Streetan 2006	4	53	3	31	1.2%	0.78 [0.19, 3.26]
Svenøy 2020	63	276	43	167	5.3%	0.89 [0.63, 1.24]
Van der Kallen 2014	6	214	34	220	2.5%	0.18 [0.08, 0.42]
Vidan 2005*	1	155	9	164	0.6%	0.12 [0.02, 0.92]
**Total (95% CI)**		**11450**		**11943**	**100.0%**	**0.76 [0.64, 0.90]**
Total events	1351		1705			

Heterogeneity: Tau² = 0.11; Chi² = 108.77, df = 28 (P < 0.00001); I² = 74%

Test for overall effect: Z = 3.18 (P = 0.001)

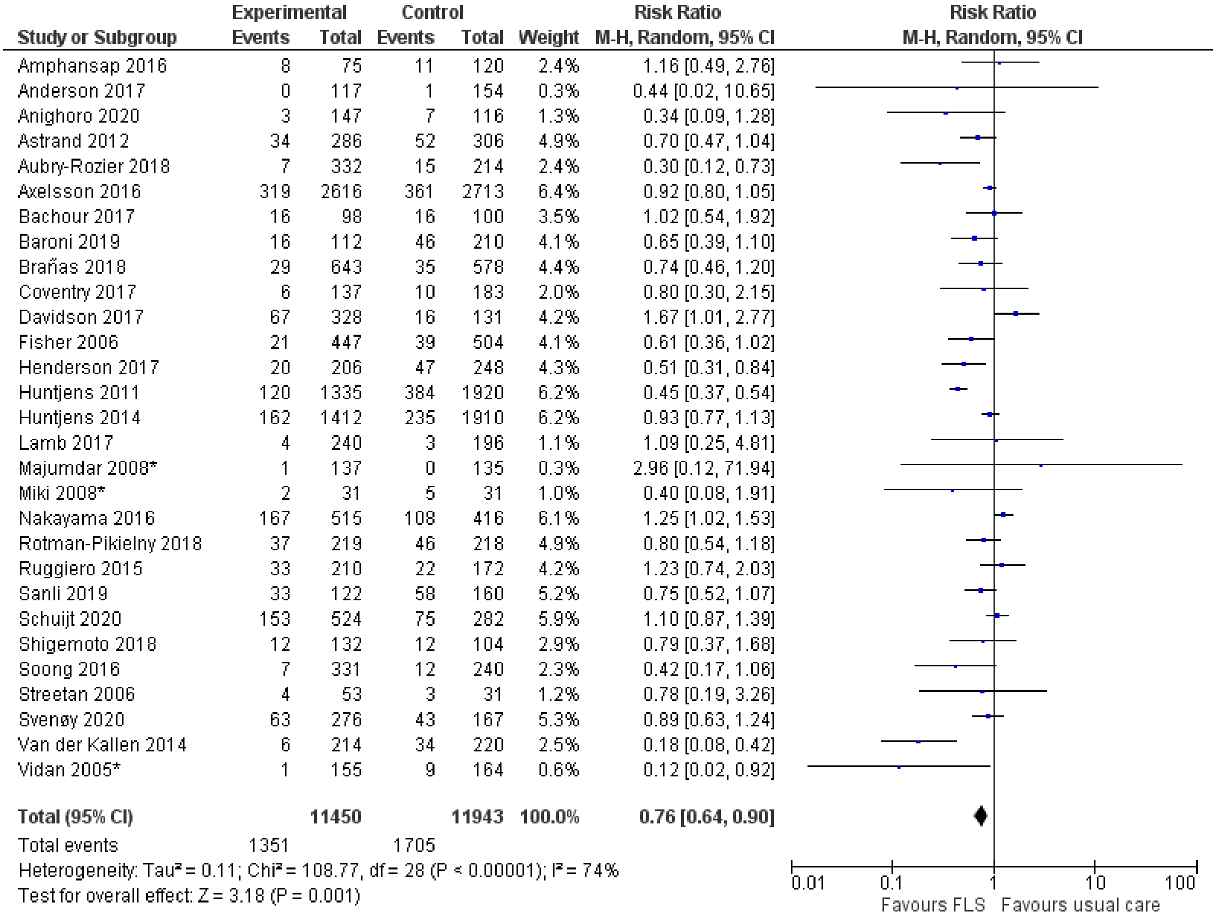

Quality evaluation

Observational studies evaluated with the Newcastle Ottawa Scale

	Selection				Comparability	Outcome			
**Cohort study**	Representativeness of the exposed cohort	Selection of the non exposed cohort	Ascertainment of exposure	Demonstration that outcome of interest was not present at start of study	Comparability of cohorts on the basis of the design or analysis	Assessment of outcome	Was follow-up long enough for outcomes to occur	Adequacy of follow-up of cohorts	tot
Wasfie 2019	1	1	1	1	2	1	1	1	9
Vaculik 2017	1	1	1	1	0	1	1	1	7
Shigemoto 2018	1	1	1	1	0	1	1	1	7
Schuijt 2020	1	1	1	1	2	1	1	1	9
Sanli 2019	1	1	1	1	2	1	1	1	9
Rotman - Pikielny 2018	1	1	1	1	2	1	1	1	9
Naranjo 2017	1	1	1	1	1	1	1	1	8
Lamb 2017	1	1	1	1	0	1	1	1	7
Inderjeeth 2018	1	1	1	1	1	1	1	1	8
Hawley 2016	1	1	1	1	2	1	1	1	9
Greenspan 2018	1	1	1	1	1	1	1	1	8
Davidson 2017	1	1	1	1	1	1	1	1	8

Coventry 2017	1	1	1	1	1	1	1	1	8
Chan 2015	1	1	1	1	0	1	1	1	7
Brañas 2018	1	1	1	1	0	1	1	1	7
Beaupre 2020	1	1	1	1	0	1	1	1	7
Beaton 2017	1	1	1	1	0	1	1	1	7
Baroni 2019	1	1	1	1	2	1	1	1	9
Bachour 2017	1	1	1	1	0	1	1	1	7
Aubry-Rozier 2018	1	1	1	1	1	1	1	1	8
Anighoro 2020	1	1	1	1	0	1	1	1	7
Anderson 2017	1	1	1	1	0	1	1	1	7
Amphansap 2020	1	1	1	1	0	1	1	1	7
Abrahamsen 2019	1	1	1	1	1	1	1	1	8
Sietsema 2018	1	1	1	0	0	1	1	0	5
Singh 2019	1	1	1	0	1	1	1	0	6
Sofie 2016	1	1	1	1	0	1	1	0	6
Soong 2016	1	1	1	1	0	1	1	0	6
Svenøy 2020	1	1	1	0	1	1	1	0	6

Summary of Findings

Randomized controlled trials

DXA TESTING

Certainty assessment							№ of patients		Effect		Certainty	Importance
№ of studies	Study design	Risk of bias	Inconsistency	Indirectness	Imprecision	Other considerations	BMD testing	placebo	Relative (95% CI)	Absolute (95% CI)		
**BMD testing**												
12	randomised trials	not serious	very serious [a]	not serious	not serious	none	933/2744 (34.0%)	438/2706 (16.2%)	**RR 1.97** (1.42 to 2.75)	**157 more per 1.000** (from 68 more to 283 more)	⨁⨁◯◯ LOW	CRITICAL

CI: Confidence interval; **RR:** Risk ratio

Explanations
a. I2>75%

TREATMENT INITIATION

Certainty assessment							№ of patients		Effect		Certainty	Importance
№ of studies	Study design	Risk of bias	Inconsistency	Indirectness	Imprecision	Other considerations	Treatment initiation	placebo	Relative (95% CI)	Absolute (95% CI)		
**Treatment initiation**												
13	randomised trials	not serious	very serious [a]	not serious	not serious	none	833/2951 (28.2%)	453/2893 (15.7%)	**RR 1.94** (1.43 to 2.62)	**147 more per 1.000** (from 67 more to 254 more)	⨁⨁◯◯ LOW	CRITICAL

CI: Confidence interval; **RR:** Risk ratio

Explanations
a. I2>75%

ADHERENCE

Certainty assessment							№ of patients		Effect		Certainty	Importance
№ of studies	Study design	Risk of bias	Inconsistency	Indirectness	Imprecision	Other considerations	Adherence	placebo	Relative (95% CI)	Absolute (95% CI)		
**Adherence**												
2	randomised trials	not serious	not serious	not serious	serious [a]	none	154/182 (84.6%)	144/184 (78.3%)	**RR 1.07** (1.01 to 1.15)	**55 more per 1.000** (from 8 more to 117 more)	⨁⨁⨁◯ MODERATE	CRITICAL

CI: Confidence interval; **RR:** Risk ratio

Explanations
a. Downgraded after the performance of a Post-Hoc Power Analysis.

REFRACTURE

Certainty assessment							№ of patients		Effect		Certainty	Importance
№ of studies	Study design	Risk of bias	Inconsistency	Indirectness	Imprecision	Other considerations	Refracture	placebo	Relative (95% CI)	Absolute (95% CI)		
**Refracture**												
1	randomised trials	serious [a]	not serious	not serious	very serious [b,c]	none	0/31 (0.0%)	1/31 (3.2%)	**RR 0.33** (0.01 to 7.88)	**22 fewer per 1.000** (from 32 fewer to 222 more)	⨁◯◯◯ VERY LOW	CRITICAL

CI: Confidence interval; **RR:** Risk ratio

Explanations
a. Unclear risk of bias for: random sequence generation, allocation concealment, blinding of outcome assessment. High risk of bias for: blinding of participants and personnel.

b. Downgraded after the performance of a Post-Hoc Power Analysis.

c. Confidence intervals crossed the line of no difference with plausible effects in favour to the experimental group.

MORTALITY

Certainty assessment							№ of patients		Effect		Certainty	Importance
№ of studies	Study design	Risk of bias	Inconsistency	Indirectness	Imprecision	Other considerations	Mortality	placebo	Relative (95% CI)	Absolute (95% CI)		
**Mortality**												
3	randomised trials	serious [a]	not serious	not serious	serious [b,c]	none	4/323 (1.2%)	14/330 (4.2%)	**RR 0.37** (0.09 to 1.61)	**27 fewer per 1.000** (from 39 fewer to 26 more)	⊕⊕◯◯ LOW	CRITICAL

CI: Confidence interval; **RR:** Risk ratio

Explanations

a. Miki 2008: unclear risk of bias for three domains (random sequence generation, allocation concealment, blinding of outcome assessment) and high risk of bias for one domain (blinding of participants and personnel). Vidan 2005: unclear risk of bias for four domains (random sequence generation, allocation concealment, blinding of participants and personnel, other bias).

b. Confidence intervals crossed the line of no difference with plausible effects in favour to the experimental group.

c. Downgraded after the performance of a Post-Hoc Power Analysis.
